# PPR-Meta: a tool for identifying phages and plasmids from metagenomic fragments using deep learning

**DOI:** 10.1093/gigascience/giz066

**Published:** 2019-06-20

**Authors:** Zhencheng Fang, Jie Tan, Shufang Wu, Mo Li, Congmin Xu, Zhongjie Xie, Huaiqiu Zhu

**Affiliations:** 1 State Key Laboratory for Turbulence and Complex Systems and Department of Biomedical Engineering, College of Engineering, Peking University, No.5 Yiheyuan Road Haidian District, Beijing 100871, China; 2 Center for Quantitative Biology, Peking University, No.5 Yiheyuan Road Haidian District, Beijing 100871, China; 3 Peking University-Tsinghua University - National Institute of Biological Sciences (PTN) joint PhD program, School of Life Sciences, Peking University, No.5 Yiheyuan Road Haidian District, Beijing 100871, China; 4 Department of Biomedical Engineering, Georgia Institute of Technology and Emory University, 631 Cherry St, Atlanta, Georgia 30332, GA, USA

**Keywords:** metagenome, mobile genetic elements, horizontal gene transfer, phage, plasmid, deep learning

## Abstract

**Background:**

Phages and plasmids are the major components of mobile genetic elements, and fragments from such elements generally co-exist with chromosome-derived fragments in sequenced metagenomic data. However, there is a lack of efficient methods that can simultaneously identify phages and plasmids in metagenomic data, and the existing tools identifying either phages or plasmids have not yet presented satisfactory performance.

**Findings:**

We present PPR-Meta, a 3-class classifier that allows simultaneous identification of both phage and plasmid fragments from metagenomic assemblies. PPR-Meta consists of several modules for predicting sequences of different lengths. Using deep learning, a novel network architecture, referred to as the Bi-path Convolutional Neural Network, is designed to improve the performance for short fragments. PPR-Meta demonstrates much better performance than currently available similar tools individually for phage or plasmid identification, while testing on both artificial contigs and real metagenomic data. PPR-Meta is freely available via http://cqb.pku.edu.cn/ZhuLab/PPR_Meta or https://github.com/zhenchengfang/PPR-Meta.

**Conclusions:**

To the best of our knowledge, PPR-Meta is the first tool that can simultaneously identify phage and plasmid fragments efficiently and reliably. The software is optimized and can be easily run on a local PC by non-computer professionals. We developed PPR-Meta to promote the research on mobile genetic elements and horizontal gene transfer.

## Findings

### Introduction

Phages and plasmids, known as mobile genetic elements (MGEs), are the main participants in horizontal gene transfer (HGT) along with genetic information exchanging among prokaryotes or eukaryotes [1]. Such elements can regulate the microbial community by interacting with the host. One of the important roles of MGEs is their ability to distribute resistance genes among bacteria and facilitate environmental adaptations among microbial communities [2]. In most cases, a substantial number of phage and plasmid genomes are present in the microbial community. For example, reports have shown that the abundance of marine phages even surpasses that of other organisms in marine systems, and more than half of the bacteria isolated from marine systems contain ≥1 plasmid [3, 4]. Thus, the identification of phage and plasmid fragments in metagenomes is a fundamental issue in comprehensive analyses of HGT and the interaction between MGEs and hosts. Although experimental approaches have been developed to enrich phages or plasmids from environment samples [5, 6], the enriched samples lose host information, which may hinder the comprehensiveness of the analyses. Therefore, computational tools for directly identifying phages and plasmids from metagenomes are expected to be developed in the field.

However, the effective identification of such elements remains a considerable challenge. Currently the fragment assembly performance of both plasmid and phage from high-throughput sequencing data is not as good as that of host-derived fragments [7]. This indicates that sequences from phages or plasmids exist as a large number of short fragments, resulting in the difficulty of the identification. In addition, fewer sequenced genomes of phages and plasmids are available compared with bacterial genomes in current databases [1]. Especially, although the abundance of viruses is estimated to exceed that of other organisms on the earth [8], so far the number of phage genomes in the NCBI database is still less than one-thirtieth the number of prokaryotic genomes, and it was estimated that more than half of the sequences from viral metagenomes could not find significant homology to the released database [5]. Therefore, it is essential to develop a tool for identifying novel phages and plasmids from metagenomic data with a large number of mixed short reads.

Despite the difficulty of identification, several tools have recently been developed to detect either phages or plasmids from culture-dependent whole-genome sequencing (WGS) data or metagenomic data. Tools that detect regions from an integrated phage sequence (referred to as prophage) over a sequenced complete bacterial genome have been designed. These tools include Prophinder [9], Phage_Finder [10], PhiSpy [11], PHAST (and its enhanced version PHASTER) [12, 13], VirSorter [14], and ProphET [15]. Such approaches primarily used a scan window to move across the complete bacterial chromosome and extract regions that seem to be phages on the basis of a similarity search against viral databases. Because the scan windows of these tools are often required to be able to cover several genes, such tools are difficult to apply to metagenomic data because the sequences of metagenome are too short to contain even a complete gene [16]. Although VirSorter can also assign metagenomic contigs as phages or bacteria, its sensitivity of identification is quite low. Moreover, lytic phages and some temperate phages do not integrate their genomes into their host chromosomes [17]; thus, these tools may only be able to identify specific phages. The tool MARVEL [18] can assign metagenomic bins as phages or bacteria and demonstrates better performance than previous tools. In the other hand, in order to identify sequences from low-abundance phages, which may not fall into bins, tools that can directly judge each fragment are also needed. In contrast, VirFinder [19] can directly judge each sequence, and it uses a logistic regression as the classifier to detect phage sequences based on *k*-mer frequencies and presents a relatively good performance. In terms of plasmids, most of the current tools for plasmid identification were designed for WGS or even specific species, such as PlasmidFinder [20], PLACNET [21], PlasmidSeeker [22], and mlplasmids [23]. However, the plasmid identification strategy for WGS may not by applicable for metagenomes. For example, PlasmidSeeker considers plasmid contigs to have a higher read coverage because plasmids may have copies in their hosts. In metagenome, however, the difference of read coverage among contigs may result from different abundances of species rather than copy number. The tool cBar [24] is the first tool designed primarily for plasmid identification in metagenomes. This tool applies sequential minimal optimization as a classifier based on *k*-mer frequencies. Similar to cBar, PlasFlow [25] is also a *k-*mer−based tool for identifying plasmids. Compared with cBar, PlasFlow further combines the information of different *k*-mer lengths and uses multiple neural networks as voting devices to determine whether the sequence belongs to the plasmid, and it achieves a better performance than cBar.

Although related tools have been developed, state-of-the-art tools for detecting short fragments have not presented satisfactory performance. Moreover, because these tools can only identify either phages or plasmids, they clearly do not meet the needs of a comprehensive analysis of MGEs and HGT. Considering that poor sequence assembly performance results in a large number of short fragments, it is a practical goal to develop a higher performing tool. In this paper, we present the PPR-Meta (Phage and Plasmid Recognizer for Metagenomes), a 3-class classifier for identifying metagenomic fragments as phages, plasmids, or chromosomes based on the deep learning algorithm. To achieve higher performance on short fragments, we designed a novel neural network architecture, which is referred to as the Bi-path Convolutional Neural Network (BiPathCNN). To the best of our knowledge, PPR-Meta is the first tool that can simultaneously identify phage and plasmid fragments efficiently and reliably.

### Dataset construction

Owing to the fact that no suitable real metagenome datasets with confident annotation are available as a benchmark, we therefore used simulated datasets with artificial contigs generated from sequenced complete genomes. We downloaded the complete genomes of prokaryote chromosomes (total of 10,090 genomes), prokaryote plasmids (total of 8,801 genomes), and phages (total of 2,279 genomes) from the NCBI genome database [26]. The list of the genomes is provided in Additional file 1. To evaluate the ability of PPR-Meta to identify novel species, genomes released before January 2016 were used to build the training set while the remainder were used to build the test set. In general, prokaryote chromosomes may contain regions of integrated phages, referred to as prophages [27]; however, most genomes do not have the prophage annotation. Here, we used ProphET (v0.5.1) to extract prophages from all the prokaryote chromosomes, and a total of 16,393 prophages predicted by ProphET (shown in Additional file 2) were incorporated into the phage dataset. Moving prophages from a chromosome dataset to a phage dataset can help to both expand the phage dataset and remove noise from the chromosome dataset. Because the predicted prophages were generated by ProphET and could not be used as a benchmark, we removed the predicted prophages from the test set. To evaluate the performance of PPR-Meta for prophage identification, we collected 267 manually annotated prophages of 54 prokaryote chromosomes from Casjens [27]. To ensure that the test data were “novel” to PPR-Meta, these prophages and their hosts were removed from the training set.

We used the MetaSim (v0.9.1) simulator [28] to extract artificial contigs from the complete genomes. Four groups of artificial contigs of different lengths were generated: Group A with a length range of 100–400 bp, Group B with a length range of 400–800 bp, Group C with a length range of 800–1,200 bp, and Group D with a length range of 5,000–10,000 bp. Groups A, B, and C were constructed to simulate the length obtained with different sequencing technologies and the average assembly contig length, while Group D was constructed to simulate long contigs in metagenomic data.

We also used real metagenomic data to estimate the reliability of PPR-Meta. The real data included phage metagenomic data of bovine rumen [29], which were downloaded from MG-RAST [30] (accessions: mgm4534202.3 and mgm4534203.3) as raw reads and assembled by SPAdes (v3.11.1) [31]; plasmid metagenomic data of bovine rumen [32], downloaded from MG-RAST (accessions: mgm4460391.3); and 20 samples of healthy human gut [33], downloaded from the NCBI SRA [34] and assembled by SPAdes. The accessions of the human gut samples are shown in Additional file 1. Additional details on the dataset construction are provided in the Methods section.

### Mathematical model of DNA sequences

The method of representing biological sequences is significant for every machine learning–based tool. Although *k*-mer frequencies have been widely used in many studies [19], such frequencies may present serious fluctuations in short sequences [35]. Here, we use a more detailed approach to represent the DNA fragments. Specifically, each sequence is represented by “base one-hot matrix (BOH)” and “codon one-hot matrix (COH).” “One-hot” is one of the most widely used encoding forms for each character in a given string in the field of natural language processing [36], and it is also used to represent bases or amino acids in biological sequences. A “one-hot” vector contains several bits, and the number of bits is equal to the number of character types in a given string. For each character type, the corresponding bit of the “one-hot” vector is 1 and the remaining bits are 0, and there must be a one-to-one correspondence between each character type and each bit. For BOH in PPR-Meta, bases A, C, G, and T are represented by [0,0,0,1], [0,0,1,0], [0,1,0,0], and [1,0,0,0], respectively. Therefore, together with the complementary strand, a sequence of length L can be represented by a BOH matrix of length 2 × L and width 4. For COH, each sequence is first expanded to 6 phases in the form of codons. For example, sequence 5′-ACGTTCGAACG-3′ will be split into the following 6 codon sequences:

1. ACG, TTC, GAA
2. CGT, TCG, AAC
3. GTT, CGA, ACG
4. CGT, TCG, AAC
5. GTT, CGA, ACG
6. TTC, GAA, CGT

Similar to BOH, each codon of COH is represented by a 64-dimensional one-hot vector, namely, one certain position is 1 and the other positions are 0. Therefore, a sequence of length L can be represented by a COH matrix of length 2 × L and width 64. Both BOH and COH will be used as input for the neural networks mentioned below.

### Structure of deep learning neural networks

**Figure 1:**
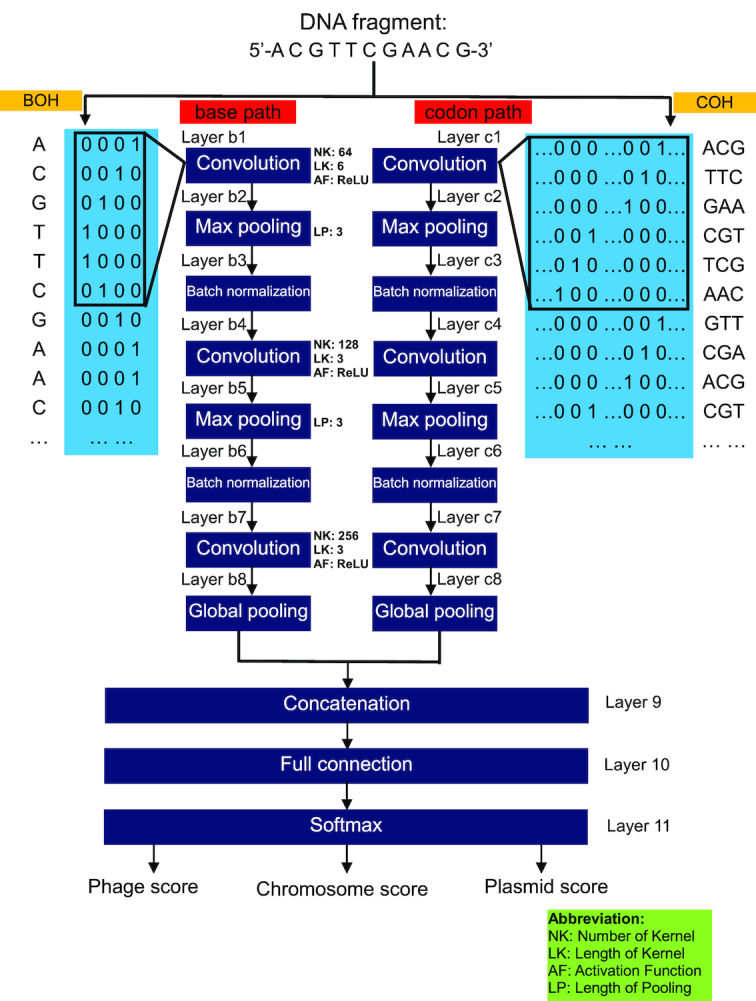
Structure of BiPathCNN. Three BiPathCNNs were trained for sequences from Groups A, B, and C. Each BiPathCNN contains a “base path” and a “codon path,” which take BOH and COH as inputs, respectively.

To ensure that PPR-Meta optimally adapts to sequences of different lengths, we trained 3 neural networks for Groups A, B, and C. To improve the performance, we designed BiPathCNN (Fig. 1), a novel neural network structure, to make reliable predictions. BiPathCNN contains a “base path” and a “codon path,” which take BOH and COH as inputs, respectively. After multiple convolution operations, the data for the 2 paths are combined by a merge layer. The fully connected layers then receive the merged data and finally output 3 scores that reflect the likelihood of the input fragment as a phage, chromosome, or plasmid.

The details of each layer are described as follows.

Layers b1 and c1: 1D convolutional layers with 64 convolution kernels using “ReLU” (Rectified Linear Unit) as the activation function. The ReLU function can be expressed as *y* = max(0, *x*). These layers take BOH or COH as inputs. The length of the convolution kernels is set to 6.

Layers b2 and c2: max pooling layers with a pooling length set to 3.

Layers b3 and c3: batch normalization layers with the dropout operation. Each element of the feature map from the previous layer in each batch will be normalized, which can speed up the convergence and prevent overfitting.

Layers b4-b6 and c4-c6: similar to layers b1–b3 or layers c1–c3, respectively. We set the number of convolution kernels in layers b4 and c4 as 128 and the length of the kernels as 3.

Layers b7 and c7: 1D convolutional layers containing 256 convolution kernels and using ReLU as the activation function. The length of the convolution kernels is set to 3.

Layers b8 and c8: 1D global average pooling layers that output the global average for each feature map of the previous layer.

Layers 9–11: The concatenation layers combine the output of the “base path” and “codon path.” After the full connection layer with the same number of nodes as the previous layer, the softmax layer calculates the probability of the input fragment as a phage, chromosome, or plasmid.

The selection of the related hyperparameters of each path mentioned above was based on LeNet-5 [37] and VGG [38], 2 classic convolutional neural networks (CNNs) in the field of artificial intelligence. Specifically, the distribution of layers was based on LeNet-5, which contained 3 convolution layers, and there was a pooling layer between every 2 convolution layers. Meanwhile, the distribution of the number of convolution kernels was based on VGG, in which the number of convolution kernels in the different layers was increased by doubling. We also referred to VGG to use ReLU as the activation function. All the neural networks used Adam as the optimizer and cross-entropy as the loss function.

In practical applications, PPR-Meta uses BiPathCNN A to predict sequences between 100 and 400 bp, BiPathCNN B to predict sequences between 400 and 800 bp, and BiPathCNN C to predict sequences between 800 and 1,200 bp. For sequences longer than 1,200 bp, such as sequences in Group D, a scan window will move across the sequence without overlapping, and the weighted average of all windows’ predictions is calculated. The length of the window is set to 1,200 bp (or less if the window ends beyond the sequence boundary). For example, given a sequence of length 2,500 bp, the scan window will first cover the bases from the first to 1,200th positions, then the window will move to bases from the 1,201st to 2,400th positions, and finally, the window will move to bases from the 2,401st to 2,500th positions. Then, PPR-Meta uses BiPathCNN C, BiPathCNN C, and BiPathCNN A to predict the subsequences under the first, second, and third windows, respectively. To generate the final score for the whole sequence, PPR-Meta calculates the weighted average of these windows. The weights of these 3 windows are 1,200/2,500, 1,200/2,500, and 100/2,500, respectively.

### Overall performance

**Figure 2:**
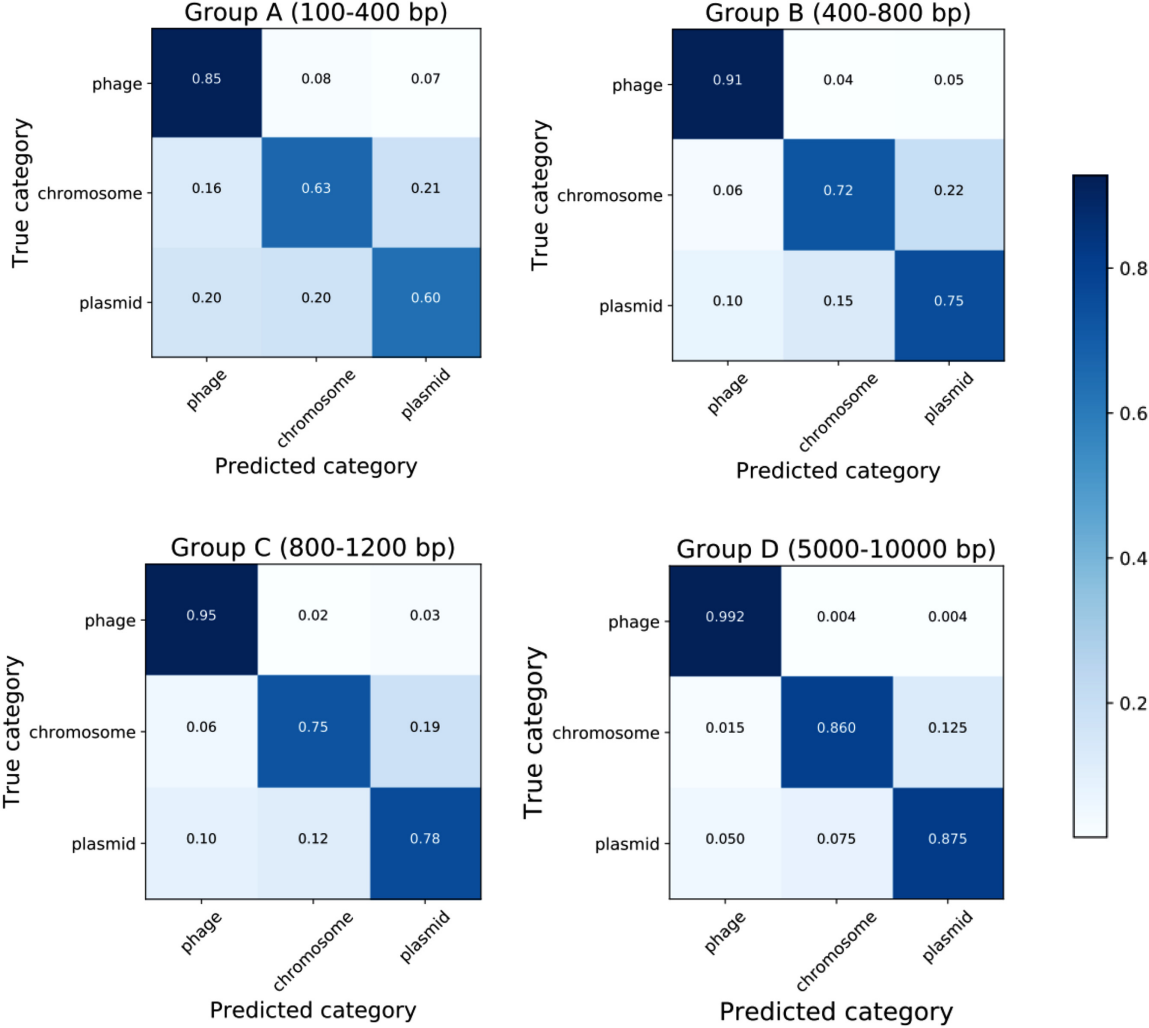
Confusion matrix of PPR-Meta. Three-class confusion matrices were used to evaluate the overall performance of PPR-Meta. Four matrices correspond to the sequences of Group A–D. In each matrix, the rows represent the true category while the columns represent the predicted category of PPR-Meta.

We evaluated PPR-Meta according to 4 groups of test sets with different lengths of short contigs. For each fragment input, the algorithm calculates 3 scores representing the likelihood that the fragment should be identified as a phage, plasmid, or chromosome. Therefore, the category with the highest score is selected as our prediction. We used 3-class confusion matrices (shown in Fig. 2) to evaluate the overall performance of PPR-Meta. In general, PPR-Meta had a better discrimination ability when the sequences were longer, and the phage recognition ability of PPR-Meta was better than the plasmid recognition ability. Plasmid sequences were easily confused with the host chromosomes, which may be because phages and plasmids face different evolutionary pressures. Because plasmids must survive in host cells, they may adapt their sequence signatures, such as the GC content and codon usage, to their hosts. In contrast, phages can assemble their own particles and remain outside of the hosts. Moreover, certain phages may contain their own transfer RNA, which allows them to change their codon usage [39]. Thus, the various similarity of phages and plasmids to their hosts may lead to differences in the identification ability of PPR-Meta. In addition, transposons may carry plasmid DNA fragments to the chromosome [1]. Therefore, the chromosome may contain regions from the plasmid. Sequences shared between the plasmid and the chromosome may also affect the judgment of PPR-Meta. Overall, PPR-Meta can effectively identify the MGEs in the test set.

### Performance comparison

We then compare PPR-Meta with VirFinder (v1.1) and VirSorter (v1.0.3) regarding the ability to identify phages, and with PlasFlow (v1.1) and cBar (v1.2) regarding the ability to identify plasmids. The evaluation criteria were the true-positive rate [TPR = true positives/(true positives + false negatives)], false-positive rate [FPR = false positives/(true negatives + false positives)], and area under the curve (AUC). Note that PlasFlow will filter uncertain predictions according to a default threshold. As a uniform comparison, we turned off this feature by setting the threshold to zero, thus using all the sequences for comparison.

The results are presented in Table 1. In all cases, the AUCs of PPR-Meta were the highest. In terms of phages, VirSorter, which is a gene-based tool, performed poorly, with almost all phages missed. This is probably because there is not a sufficient number of full-length genes present in short DNA fragments for VirSorter's analysis. This also indicates that methods based on homology searches of genetic information are not applicable to species identification in metagenomes. Considering that most contigs of metagenomes are short fragments, especially those of MGEs, VirSorter is not competent for phage identification despite achieving a higher performance for long contigs in Group D. The tool VirFinder outperformed VirSorter. As an alignment-free tool, VirFinder achieved a much higher TPR, especially in short fragments. The TPR of PPR-Meta was approximately 10% higher than that of VirFinder and the FPR was approximately 5−10% lower. The performance improvement on short sequences demonstrates that our sequence representation method is more detailed than the *k*-mer frequencies, and the deep learning algorithm is more capable of extracting sequence features than the logistic regression used by VirFinder. In terms of plasmids, both cBar and PlasFlow did not perform well. cBar appeared to produce random results in most cases, with both the TPR and FPR near 50%. Although the AUC of PlasFlow was slightly higher than that of cBar, PlasFlow tended to categorize most sequences as plasmids, which resulted in an extremely high FPR. For PPR-Meta, the TPR was comparable to that of PlasFlow, while the FPR was approximately 25−40% lower. In some cases, a few assembled sequences from high-abundance species may be much longer, so we also tested PPR-Meta and the related tools using 15 and 30 kbp fragments (shown in Additional File 3, Fig. S1). The results showed that the performance of PPR-Meta was still the best for these long sequences. In addition, we tested the accuracy as well as the running time of each BiPathCNN on test datasets from different groups and found that using a non-corresponding BiPathCNN to predict sequences from specific groups would lead to a lower accuracy and longer running time (shown in Additional File 3, Fig. S2). Overall, PPR-Meta presented a much better performance than other homology-search−based tools such as VirSorter and k-mer−based tools such as VirFinder, PlasFlow, and cBar.

**Table 1:** Evaluation of the performance of PPR-Meta and comparison of the performance of PPR-Meta and related tools

Group	Tool	Evaluation on phage (%)			Evaluation on plasmid (%)		
		TPR	FPR	AUC	TPR	FPR	AUC
Group A 100–400 bp	PPR-Meta	84.96	18.01	91.82	59.91	14.14	83.05
	VirFinder	73.77	25.45	81.30	NA	NA	NA
	VirSorter	0	0	50.00	NA	NA	NA
	PlasFlow	NA	NA	NA	71.89	62.59	56.30
	cBar	NA	NA	NA	52.68	46.07	53.31
Group B 400–800 bp	PPR-Meta	90.75	8.37	97.21	74.56	13.37	89.64
	VirFinder	79.27	18.15	88.64	NA	NA	NA
	VirSorter	0.05	0.002	50.02	NA	NA	NA
	PlasFlow	NA	NA	NA	72.61	55.01	62.50
	cBar	NA	NA	NA	55.00	43.59	55.70
Group C 800–1,200 bp	PPR-Meta	95.24	7.75	98.54	78.09	10.95	91.84
	VirFinder	81.91	15.63	91.09	NA	NA	NA
	VirSorter	0.17	0.002	50.09	NA	NA	NA
	PlasFlow	NA	NA	NA	75.89	50.55	68.01
	cBar	NA	NA	NA	55.54	41.87	56.84
Group D 5–10 kbp	PPR-Meta	99.20	3.25	99.77	87.53	6.45	96.02
	VirFinder	89.26	8.13	97.12	NA	NA	NA
	VirSorter	66.80	2.48	82.66	NA	NA	NA
	PlasFlow	NA	NA	NA	88.50	30.22	88.42
	cBar	NA	NA	NA	63.79	32.61	65.59

NA: not applicable.

### Effectiveness of BiPathCNN

PPR-Meta achieved much higher performance than the other methods as mentioned above. The innovation of PPR-Meta is the design of BiPathCNN, which uses both base and codon information to improve the performance. In BiPathCNN, the “base path” is beneficial to extracting the sequence features of non-coding regions while the “codon path” is beneficial to extracting coding regions. To verify the effectiveness of BiPathCNN, we removed the codon path and base path and retrained PPR-Meta. The newly trained PPR-Meta was tested, and the results showed that the performance of PPR-Meta with either the base path or codon path presented a lower performance only relative to that of BiPathCNN in most cases (Table 2). Moreover, the performance of the codon path−only CNN was better than that of base path−only CNN, which indicates that the features that distinguish phages, chromosomes, and plasmids are more concentrated in the coding region. Compared with other sequence representation methods that ignore the coding or non-coding region, such as methods based on *k*-mer frequencies, PPR-Meta uses a more detailed method of describing a sequence and achieves a higher performance.

**Table 2:** Performance comparison among BiPathCNN, the base path−only CNN, and codon path−only CNN

Group	Tool	Evaluation on phage (%)			Evaluation on plasmid (%)		
		TPR	FPR	AUC	TPR	FPR	AUC
Group A 100–400 bp	BiPathCNN	84.96	18.01	91.82	59.91	14.14	83.05
	Base path−only	81.86	24.58	87.50	56.96	17.60	78.50
	Codon path−only	86.84	20.47	91.85	62.15	16.65	82.26
Group B 400–800 bp	BiPathCNN	90.75	8.37	97.21	74.56	13.37	89.64
	Base path−only	88.76	17.46	93.87	72.37	18.86	85.57
	Codon path−only	84.95	5.93	96.57	82.98	23.10	88.32
Group C 800–1,200 bp	BiPathCNN	95.24	7.75	98.54	78.09	10.95	91.84
	Base path−only	92.09	17.71	95.47	73.31	15.12	88.02
	Codon path−only	94.60	12.44	97.55	73.17	12.41	89.22

### Performance in the presence of sequencing errors

Sequencing errors exist in various sequencing technologies, and tools that handle high-throughput sequencing data should be able to tolerate these errors. In addition, the third-generation sequencing technologies, such as Pacific Biosciences and Nanopore, have much higher rates of sequencing errors. Thus, the compatibility of tools with new sequencing technologies should be considered.

Sequencing errors can be divided into 2 types: base substitutions and base insertions or deletions. We tested the impact of these 2 types of sequencing errors on the identification performance of PPR-Meta and related tools. We used MetaSim to extract modified fragments with 1% substitutions and 1% insertions or deletions separately from the test genomes. We used the same criteria described above to compare the performance of different tools in terms of both types of error. The results are presented in Tables 3 and 4.

**Table 3:** Identification performance of each tool with 1% base substitutions

Group	Tool	Evaluation on phage (%)			Evaluation on plasmid (%)		
		TPR	FPR	AUC	TPR	FPR	AUC
Group A 100–400 bp	PPR-Meta	84.42	17.99	91.57	61.19	15.27	82.76
	VirFinder	72.55	26.20	80.42	NA	NA	NA
	VirSorter	0	0	50.00	NA	NA	NA
	PlasFlow	NA	NA	NA	71.72	62.82	55.86
	cBar	NA	NA	NA	52.98	46.18	53.40
Group B 400–800 bp	PPR-Meta	90.05	8.48	97.02	75.07	14.03	89.39
	VirFinder	78.50	18.75	87.95	NA	NA	NA
	VirSorter	0.02	0	50.01	NA	NA	NA
	PlasFlow	NA	NA	NA	72.31	55.61	61.87
	cBar	NA	NA	NA	54.83	44.63	55.10
Group C 800–1,200 bp	PPR-Meta	94.54	7.72	98.33	79.03	11.99	91.59
	VirFinder	81.29	15.92	90.68	NA	NA	NA
	VirSorter	0.21	0	50.11	NA	NA	NA
	PlasFlow	NA	NA	NA	75.24	50.91	67.15
	cBar	NA	NA	NA	56.57	42.85	56.86
Group D 5–10 kbp	PPR-Meta	98.97	3.15	99.75	87.65	7.20	95.83
	VirFinder	88.90	8.19	97.01	NA	NA	NA
	VirSorter	60.30	1.13	79.80	NA	NA	NA
	PlasFlow	NA	NA	NA	88.57	31.42	87.86
	cBar	NA	NA	NA	64.31	34.63	64.84

NA: not applicable.

**Table 4:** Identification performance of each tool with 1% base insertions or deletions

Group	Tool	Evaluation on phage (%)			Evaluation on plasmid (%)		
		TPR	FPR	AUC	TPR	FPR	AUC
Group A 100–400 bp	PPR-Meta	80.26	18.64	89.28	65.29	19.93	81.62
	VirFinder	72.62	25.96	80.57	NA	NA	NA
	VirSorter	0	0	50.00	NA	NA	NA
	PlasFlow	NA	NA	NA	71.12	62.83	55.81
	cBar	NA	NA	NA	53.63	46.43	53.60
Group B 400–800 bp	PPR-Meta	85.50	9.69	95.26	77.44	17.57	88.48
	VirFinder	79.00	18.76	88.28	NA	NA	NA
	VirSorter	0.24	0	50.12	NA	NA	NA
	PlasFlow	NA	NA	NA	72.74	55.44	62.31
	cBar	NA	NA	NA	55.38	45.22	55.08
Group C 800–1,200 bp	PPR-Meta	92.99	9.12	97.54	79.74	14.29	90.80
	VirFinder	81.98	16.00	90.93	NA	NA	NA
	VirSorter	2.38	0.02	51.18	NA	NA	NA
	PlasFlow	NA	NA	NA	75.23	51.25	66.75
	cBar	NA	NA	NA	56.74	43.45	56.64
Group D 5–10 kbp	PPR-Meta	98.90	3.51	99.73	89.23	8.48	95.84
	VirFinder	88.93	8.40	96.98	NA	NA	NA
	VirSorter	47.25	0.25	73.51	NA	NA	NA
	PlasFlow	NA	NA	NA	88.74	31.70	88.08
	cBar	NA	NA	NA	64.62	35.40	64.61

NA: not applicable.

In most cases, in the presence of 1% base substitutions, a slight decrease in each evaluation criterion was observed for each tool compared with that in the presence of non-sequencing errors, although the decrease was not obvious. PPR-Meta was still the best-performing tool. When 1% of the bases were inserted or deleted, the performance of most tools was slightly reduced with the exception of VirSorter. Base insertions or deletions caused substantial fluctuations in the performance of VirSorter. For sequences of Group D, the AUC of VirSorter decreased by ∼9% compared with sequences with no errors. In our opinion, the reason that VirSorter exhibits great fluctuations in performance with base insertions or deletions is that insertions and deletions disrupt the phase of the open reading frame (ORF). VirSorter identifies phage sequences primarily by observing the distribution of genes, such as the densities of known viral genes or the enrichment of short genes. Disrupting the ORF phase will severely affect gene identification [40], thereby leading to interference in the downstream analysis. Thus, although VirSorter can achieve relatively good performance on long contigs, caution should be taken when applying VirSorter to data generated by third-generation sequencing technology.

Considering that the error rate of the raw data generated from third-generation sequencing technology may be much higher, we also tested PPR-Meta and the related tools using artificial contigs modified with 10% base substitutions and 10% insertions or deletions in Group D, whose lengths were close to those of the raw reads generated from third-generation sequencing technology. The results are shown in Additional File 3 and Fig. S3. The results showed that the AUCs of PPR-Meta remained the highest (>90%), although the performance fluctuated somewhat, especially in the presence of 10% insertions or deletions. Recently, many dedicated tools have been developed to help improve the consensus accuracy for third-generation sequencing technology to >99% [41]; therefore, the extremely high error rate on the raw data should not affect the use of PPR-Meta on assembled third-generation sequences.

### Prophage identification ability

We tested the prophage identification ability of the related tools on the 267 manually annotated prophages. The results in Table 5 showed that although the recognition rate of PPR-Meta for prophages was lower than that for phage contigs generated from the NCBI database, the overall performance of PPR-Meta was still much better than that of VirFinder and VirSorter. We additionally collected 139 manually verified prophages from the PhAnToMe database [42], and most of the hosts of these prophages were not the same as those of the previous 267 prophages. Consistent with the results for the 267 prophages, the prophage recognition rate of PPR-Meta was much higher than that of the comparative tools (shown in Additional File 3, Fig. S4), indicating that PPR-Meta can identify more prophages. The lower recognition rate of prophages compared with that of the phages in the NCBI database may be due to the difference of sequence pattern between prophages and phages in the NCBI database. The complete genomes in the NCBI database tend to come from phages that are easily obtained experimentally, while prophages hide their genomes in the hosts. During co-evolution, prophages may adjust the sequence pattern according to their hosts to eliminate the hosts’ restriction enzymes [39]. In terms of VirFinder, the ability to identify prophages was significantly reduced and more than half of the prophages were missed, which may be because VirFinder ignored prophages that exist in the chromosomes during training. In both the training and test set of VirFinder, all prophages were labelled as chromosomes, which led to the misjudgement of prophages. In microbial communities, temperate phages are dominant and a significant portion of temperate phages exist in the form of prophages [43]. For example, prophages have been shown to represent the main component of phages in healthy human guts [17]. In certain prokaryotes, prophages account for up to 20% of the host chromosome [27]. Thus, compared with VirFinder, PPR-Meta may be more adapted to real microbial communities because it can identify more prophages.

**Table 5:** Recognition rate of prophages

Group	Tool	Recognition rate (%)
Group A 100–400 bp	PPR-Meta	60.79
	VirFinder	43.46
	VirSorter	0
Group B 400–800 bp	PPR-Meta	60.59
	VirFinder	40.77
	VirSorter	0
Group C 800–1,200 bp	PPR-Meta	68.09
	VirFinder	41.94
	VirSorter	0.05
Group D 5–10 kbp	PPR-Meta	75.58
	VirFinder	48.62
	VirSorter	37.75

### Evaluation in real metagenomic data

We also evaluated PPR-Meta and the related tools using real metagenomic data. We first evaluated whether PPR-Meta can identify MGEs using both phage metagenomic and plasmid metagenomic data of bovine rumens, in which either phages or plasmids were enriched before sequencing. The phage metagenomic data were downloaded as raw reads, and a total of 107,529 contigs were generated after assembly. VirSorter, VirFinder, and PPR-Meta were run on the phage metagenome. Consistent with the results for artificial contigs, VirSorter missed nearly all the phages and only 0.02% of the contigs were identified. VirFinder and PPR-Meta were much better than VirSorter and identified 68.86% and 76.90% of the contigs, respectively, showing that PPR-Meta had the highest coverage of this data set.

The plasmid metagenomic data were downloaded as assembled contigs containing 5,771 sequences. It is worth noting that there are a certain number of phages that survive as circular DNA [17], and when enriching plasmids, these circular phages will also be extracted together with the plasmids. Thus, the plasmid metagenome may contain a mixture of phages and plasmids in which the host chromosomes are filtered. From the RefSeq viral database, we collected the genes labelled as “portal,” “spike,” “major capsid protein,” “terminase large subunit,” “tail,” “coat,” or “virion formation,” which were more likely to exist in phages [14]. We found that one of the sequences contained a homologous region of the portal protein by applying the blastx search (e-value ≤ 1e−4), indicating that phages are likely to co-exist with plasmids in this dataset. Therefore, PPR-Meta, VirSorter, VirFinder, cBar, and PlasFlow were all run on this dataset. The results showed that VirSorter did not identify any sequences as phages while VirFinder identified 49.90% as phages. cBar and PlasFlow identified 64.46% and 74.67% of the sequences as plasmids. For PPR-Meta, a total of 81.96% of the sequences were identified as MGEs, in which 49.18% were phages and 32.78% were plasmids. More than half of the sequences (64.73%) predicted as phages by PPR-Meta were also predicted as phages by VirFinder, and most of the sequences (74.74%) predicted as plasmids by PPR-Meta were also predicted by PlasFlow. Furthermore, the sequence containing the homologous region of the portal protein was identified as phages and 8 out of 10 sequences coding plasmid backbone functions listed in Fig. 3 of Kav et al. [32] were also identified as plasmids by PPR-Meta. Thus, the prediction of PPR-Meta may be reliable. Because of the filtering of chromosomes from this dataset, PPR-Meta could identify most of the extrachromosomal elements with the fewest false-negative predictions.

Because we lack samples in which only chromosomes are enriched and all the extrachromosomal elements are filtered, estimating whether related tools will misjudge chromosomes as MGEs directly is difficult using real data. Because 16S ribosomal RNA (rRNA) is more likely to occur in chromosomes, sequences containing the homologous region of 16S rRNA are likely chromosome-derived. We collected 20 metagenome samples from the human gut, which represented mixtures of phages, chromosomes, and plasmids. All contigs of the samples were searched against the 16S rRNA database of Greengenes [44] using blastn, and the contigs containing the homologous region (e-value ≤ 1e−4, hits length ≥ 250) of 16S rRNA were collected. Hits longer than 250 bp could cover ≥1 conserved region of 16S rRNA, so the alignments were reliable. In terms of phage identification, PPR-Meta, VirFinder, and VirSorter predicted an average of 3.43%, 11.32%, and 0% of the 16S-like contigs as phages, respectively, indicating that PPR-Meta likely generated fewer false-positive predictions than VirFinder. Although VirSorter did not cover any of the 16S-like contigs, the low number of false-positive predictions came at the cost of missing almost all phages as shown above. In terms of plasmid identification, PPR-Meta, PlasFlow, and cBar predicted an average of 26.69%, 52.57%, and 63.36% of the 16S-like contigs as plasmids, respectively, indicating that PPR-Meta may generate the lowest number of false-positive predictions. Because individual extrachromosomal elements also contain ribosomal RNA, especially large plasmids [45], the coverage of 16S-like contigs may be higher than the real FPR. Overall, PPR-Meta can identify more MGEs with fewer false-positive results.

Considering that third-generation sequencing technology is more and more widely used to analyse metagenomes, we also used real virome data generated by MinION [46] to test whether PPR-Meta and the related tools can identify phages from third-generation sequencing technology. The virome was downloaded as assembled sequences (accession: GCA_900 491 955.1), containing 1,500 sequences. The results showed that PPR-Meta, VirFinder, and VirSorter could identify 79.20%, 76.27%, and 30.40% of viral sequences, respectively, indicating that PPR-Meta has the highest performance. Therefore PPR-Meta can also handle data from third-generation sequencing technology, although it is designed primarily for second-generation sequencing technology.

### Phages and plasmids in the human digestive tract

**Figure 3:**
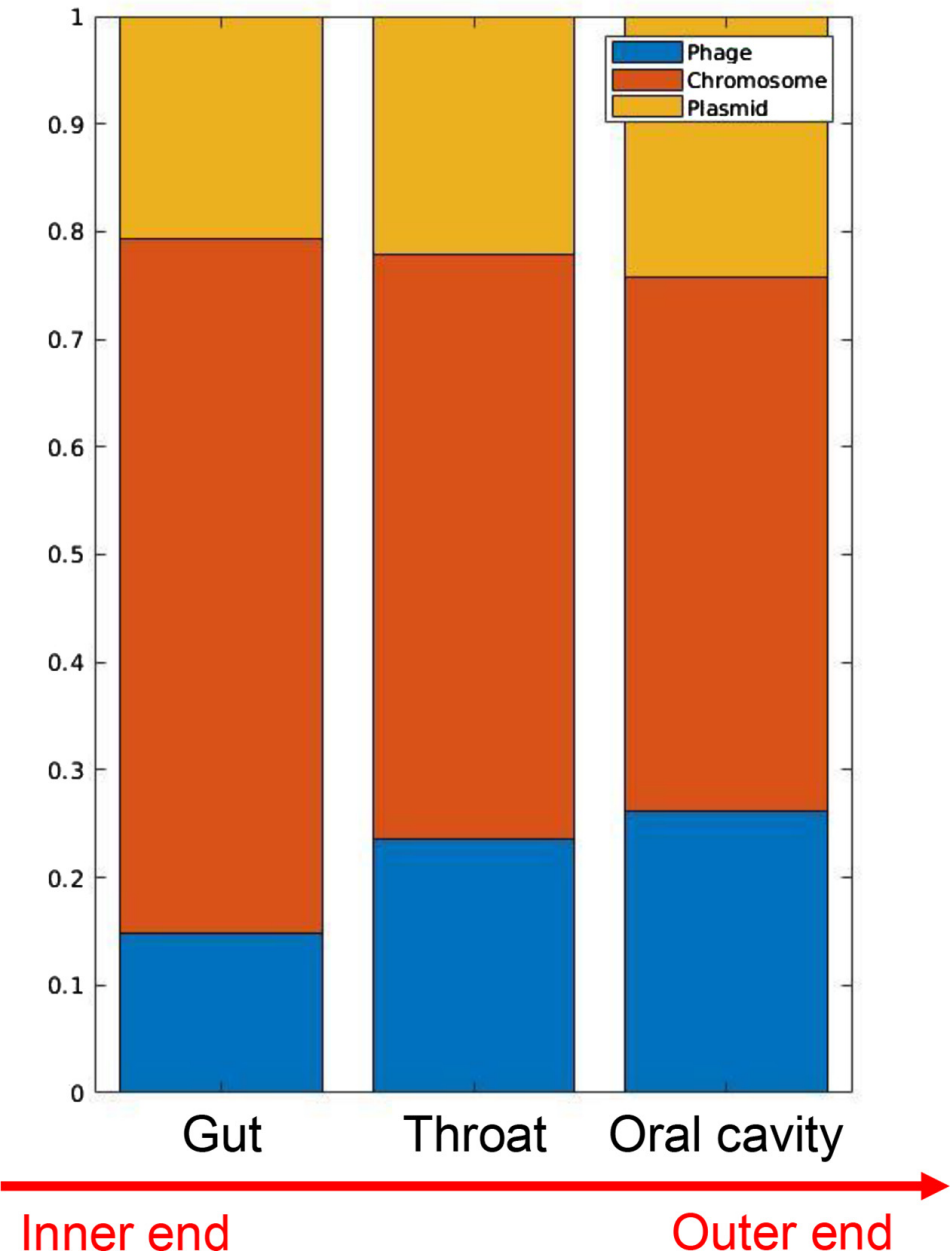
Percentages of phages, chromosomes, and plasmids in the human digestive tract. PPR-Meta was used to predict the sequences of phages, chromosomes, and plasmids in metagenomic assemblies, including samples from the gut, throat, and oral cavity. The sequence percentages of phages, chromosomes, and plasmids were calculated.

As an application example, we used PPR-Meta to analyse the percentages of phages, bacterial chromosomes, and plasmids in microbial communities from the human digestive tract. We collected 10 samples from the gut (sampling from stools), 7 samples from the throat, and 10 samples from the oral cavity (sampling from the tongue dorsum). All samples were downloaded from the Human Microbiome Project [47] as assembled contigs. The accessions of all samples are provided in Additional File 1. PPR-Meta was run on all samples, and the percentages of sequences predicted as phages, chromosomes, and plasmids were calculated. The results are shown in Fig. 3. We found that in the positions closer to the outer end of the digestive tract, the percentages of phages and plasmids tended to be higher. For example, in the gut, the inner end of the digestive tract, the percentages of phages and plasmids were lower, while in the oral cavity, the outer end of the digestive tract, the percentages were higher. Especially, phage sequences occupied ∼14.80% of all sequences in the gut, which was consistent with the estimated viral proportion in the human gut (4−17%) [48]. In the oral cavity, the percentage of phage sequences was obviously higher, occupying ∼26.23% of all sequences. It has been reported that the number of phages in the oral cavity is estimated to be 35 times more than that of bacteria [49], indicating that the high percentage of phages predicted by PPR-Meta may be reliable. Moreover, the high percentages of phages and plasmids means that HGT may be more frequent. Because the outer end of the digestive tract is closer to the changing external environment, HGT seems to be a way for microbial communities at the outer end of the digestive tract to adapt to the external environment.

### Usage of PPR-Meta

PPR-Meta takes the sequence file in fasta format as input and outputs a tabular file. The output file contains 3 scores between 0 and 1 that reflect the likelihood of obtaining phages, chromosomes, and plasmids for each sequence. By default, the final prediction is the category with the highest score. To meet users’ actual requirements, PPR-Meta is designed with the option to adjust the threshold to filter out the uncertain predictions so that the remaining predictions may be more reliable. Given a threshold, a sequence with a highest score lower than the threshold will be labelled as “uncertain.” In this way, the outputs of PPR-Meta contain 6 categories: phage, uncertain phage, chromosome, uncertain chromosome, plasmid, and uncertain plasmid. We evaluated the uncertain prediction rate, accuracy, AUC, TPR, and FPR under different thresholds, and the results are shown in Additional File 3, Fig. S5. In general, with a higher threshold, the accuracy, AUC, and TPR as well as the uncertain prediction rate will be higher, while the FPR will be lower.

PPR-Meta is user friendly, and the program has been optimized in a virtual machine so that users can directly run PPR-Meta without installing any dependency package. We also provided a short video guide to show how to install the virtual machine. If users are analysing large-scale data, running the executable file on the physical host is more suitable. In this way, when the GPU is available, PPR-Meta will run on the GPU automatically to speed up the program. The memory requirements are dependent on the data size. We recommend ≥4−6 GB of available memory when running the virtual machine or ≥16 GB when handling large-scale data on the physical host. We tested the running time of PPR-Meta using 90,000 sequences from 100 to 10,000 bp and found that this tool can handle all sequences in ∼45 minutes on a machine with the following configuration: CPU: Intel Core i7 6700; GPU: NVIDIA GTX1060; and Memory: 64G, DDR4.

## Discussion and Conclusions

In this paper, we proposed an *ab initio* method, PPR-Meta, to identify both phages and plasmids from metagenomic sequences. PPR-Meta uses a novel strategy to improve the MGE identification performance and avoids performing similarity searches to make judgments. Similarity search−based tools, such as VirSorter, provide good results for long sequences. However, such methods do not work effectively for short fragments owing to the insufficient number of genes for the statistical analysis. Compared with other reference-free tools, PPR-Meta uses a more detailed method of characterizing DNA sequences. We use a BOH matrix, which is beneficial for non-coding regions, and a COH matrix, which is beneficial for coding regions, to represent sequences. In contrast, traditional *k*-mer methods do not consider coding or non-coding regions. When the sequence is short, k-mer frequencies will be noisy. On the other hand, *k*-mer−based methods may also be more sensitive to the sequence length than the BiPathCNN method in the present work. The distribution of *k*-mer frequencies may be different between long and short sequences, and the variance of the *k*-mer frequencies for short sequences may be much higher. Thus, the *k*-mer−based classifier constructed using short-sequence data may not be applicable for long sequences, and vice versa. Among the *k*-mer−based tools, cBar was trained with complete genomes and PlasFlow was trained on 10-kbp fragments, which might make it difficult for them to adapt to metagenomic data with a wide range of lengths. In contrast, our BiPathCNN directly extracts sequence features from the raw data represented by the one-hot matrix and may be less sensitive to sequence length. Tests of each BiPathCNN on test datasets from different groups (Additional File 3, Fig. S2) also showed that although the overall accuracy was slightly reduced when testing each group using a non-corresponding BiPathCNN from the other groups, the decrease was not obvious, indicating that our approach is not quite sensitive to the sequence length. Another shortcoming of *k*-mer−based tools may be that mapping sequences of different length for *k*-mer feature vectors with the same dimension will also lose some information. PPR-Meta takes all bases and codons as inputs in the neural network, thereby exploiting all information in the fragments. In the design of the algorithm, we used a deep learning network as the classifier. Deep learning has achieved great success in many fields, such as long non-coding RNA identification [50] and the prediction of sequence specificities of nucleic acid binding proteins [51]. In the construction of PPR-Meta, we designed the BiPathCNN, which contains a “base path” and “codon path” to handle the BOH matrix and COH matrix, respectively. Testing showed that the performance of the CNN with double paths was better than that with a single path.

Furthermore, we were surprised to find that PPR-Meta's output scores were able to describe the interaction between phages or plasmids and their hosts. Specifically, the difference between the phage score and chromosome score reveals the lifestyle of the phages (virulent or temperate), while the difference between the plasmid score and chromosome score reveals the transmissibility of the plasmids (transmissible or non-transmissible). We collected both phage genomes with lifestyle annotations from McNair et al. [8] and plasmid genomes with transmissibility annotations from Shintani et al. [52] and then extracted artificial contigs. PPR-Meta was run on all the contigs, and the correctly predicted contigs were retained. From the results, 2 normalized statistics were constructed:

\begin{equation*}
\mathrm{life}\_\mathrm{score} = \left( {\mathrm{phage}\_\mathrm{score} - \mathrm{chromosome}\_\mathrm{score}} \right)/\mathrm{phage}\_\mathrm{score}
\end{equation*}

and

\begin{equation*}
\mathrm{trans}\_\mathrm{score} = \left( {\mathrm{plasmid}\_\mathrm{score} - \mathrm{chromosome}\_\mathrm{score}} \right)/\mathrm{plasmid}\_\mathrm{score}.
\end{equation*}

**Figure 4:**
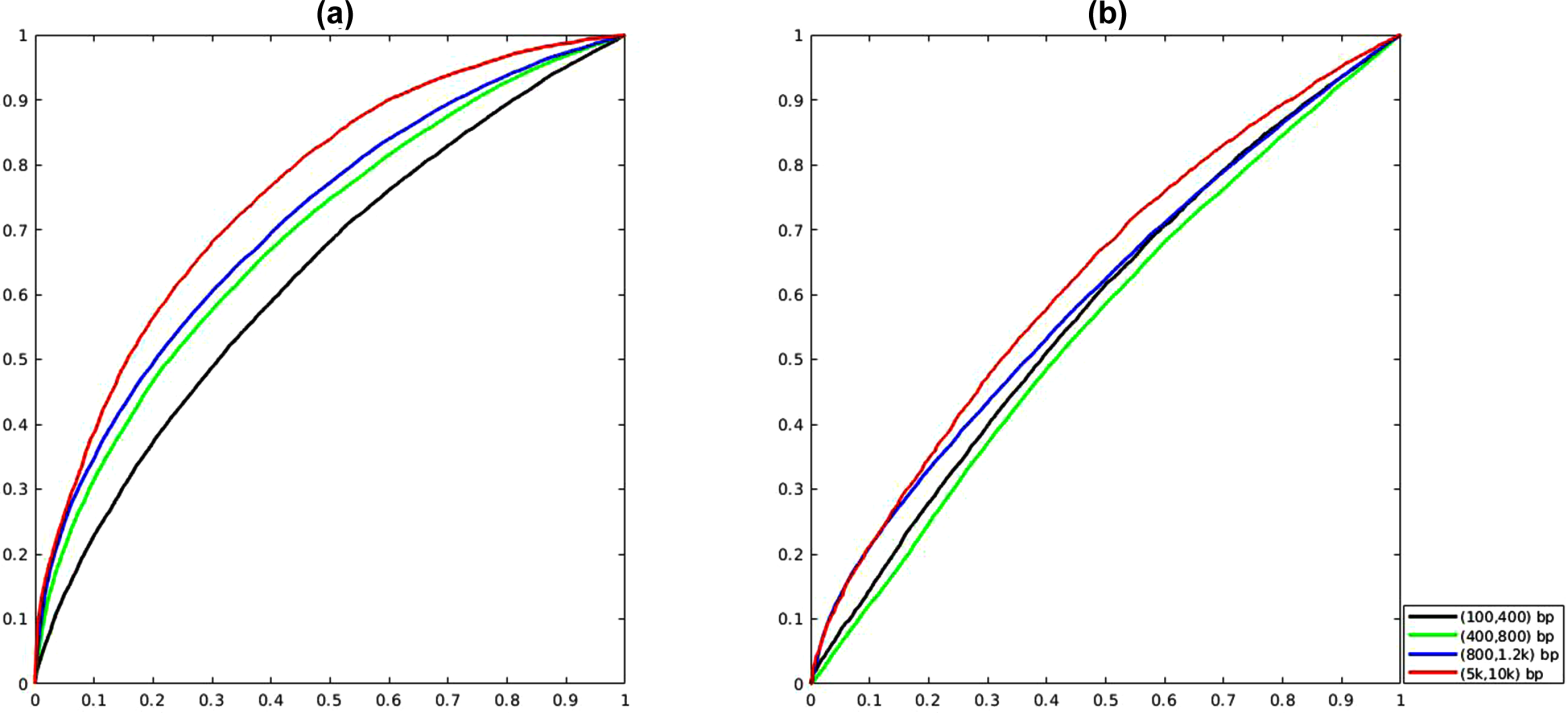
ROC curve of classifying phage lifestyle and plasmid transmissibility. (a) Classifying virulent phages and temperate phages using life_score. In order of sequence length, the AUC is 0.63, 0.69, 0.71, and 0.76. (b) Classifying transmissible plasmid and non-transmissible plasmid using trans_score. In order of sequence length, the AUC is 0.58, 0.55, 0.60, and 0.62.

The receiver operating characteristic curve (ROC) showed that life_score could distinguish the lifestyle of phages while trans_score could distinguish the transmissibility of plasmids with AUC > 0.5 (shown in Fig. 4). Specifically, temperate phages tend to have lower life_score values and non-transmissible plasmids tend to have lower trans_score values. This phenomenon may be due to the genome amelioration of foreign DNA to the host. For example, research has shown that the comparison of the trinucleotide composition between a plasmid and bacterial chromosome can be used to predict the host range of plasmids [53]. Because temperate phages and non-transmissible plasmids experience longer residence times within the host cell, they may adjust the sequence pattern toward the host. Thus, the sequence pattern between temperate phages (or non-transmissible plasmids) and host chromosomes may be more similar than that between virulent phages (or transmissible plasmids) and host chromosomes, thereby resulting in a lower life_score (or trans_score) value. Although tools that can classify phage lifestyle and plasmid transmissibility on metagenomes are lacking as far as we know, the aforementioned phenomena may provide insights into the classification strategy for future studies.

In general, bacteria contain genomic islands, regions of horizontal origin on chromosomes [54]. The formation mechanisms of some genomic islands are caused by phages or plasmids [55]. To see how PPR-Meta and related tools perform on DNA fragments from these regions, we collected genomic island sequences from the Islander database [56]. Upon testing on artificial contigs of between 100 bp and 10 kbp extracted from these genomic islands, the results showed that PPR-Meta could identify 65.25% of them as foreign DNA (either phage or plasmid), while VirFinder, VirSorter, PlasFlow, and cBar could identify 20.46%, 6.72%, 53.11%, and 51.62% of them, respectively, indicating that PPR-Meta can better recognize sequences from regions of horizontal origin on bacterial chromosomes.

PPR-Meta also has some limitations. In addition to prokaryote chromosomes, plasmids, and phages, other organisms of low abundance may exist in the microbial community, such as fungi and protozoans. Such organisms are not included in the training set of PPR-Meta and may have interfered with the judgment of PPR-Meta. To increase the suitability of PPR-Meta for real situation, we will retrain PPR-Meta regularly with expanded datasets. More organisms, as well as newly sequenced genomes, will be added to the dataset so that PPR-Meta will become more powerful and reliable. In addition, due to sequence exchanges among phages, plasmids, and chromosomes, there are a few chimeric sequences from 2 sources (e.g., a prophage and chromosome chimera). PPR-Meta cannot perform detailed judgments about these chimeras, and we are considering how to further identify such sequences. However, because these chimeras do not exist at a large scale, we believe that the presence of chimeras will not have a significant impact on the application of PPR-Meta.

In conclusion, the performance of PPR-Meta has been shown to be much better than that of currently available similar tools, while none of these tools can function as PPR-Meta does. It is thus expected that the PPR-Meta tool will meet the demand of metagenomics analysis when considering the microbial community tangled with phages and plasmids, and certainly qualifies as a powerful tool for the research community.

## Methods

PPR-Meta was trained and tested using artificial contigs. We downloaded the accession list of prokaryote chromosomes, prokaryote plasmids, and phages from the NCBI genome database, and the corresponding genomes were downloaded according to the list. To ensure the quality of the data, we only used the complete genomic molecule with the RefSeq accession prefix. Because chromosomes may contain prophages, we used ProphET to extract the prophage regions of all chromosomes. ProphET requires a genome sequence file (fasta format) and genome annotation file (gff format) as inputs. A few genomes do not contain the annotation information, and these genomes were removed from the dataset. We then used MetaSim to generate 4 groups of artificial contigs with different lengths as mentioned in the main text. To generate artificial contigs with no error for both training and test sets, we used the “exact” preset to return fragments exactly matching reference sequences. In each group, the “DNA Clone Size Distribution Type” was set to “Uniform.” To generate artificial contigs modified with sequencing errors, we used the “Sanger” preset, which allows users to modify sequences according to their settings. Note that because we were not going to generate sequences with technology-specific errors, the following settings do not reflect the real situation of the Sanger technology. For the generation of sequences with 1% base substitutions, the “Read Length Distribution Type” was set to “Uniform” and the “Mate Pair Probability” was set to 0; both the “Error Rate at Read Start” and the “Error Rate at End of Read” were set to 0.01; and both the “Insertion Error Rate” and “Deletion Error Rate” were set to 0. For the generation of sequences with 1% base insertions or deletions, most settings were the same as mentioned above, except that both the “Insertion Error Rate” and “Deletion Error Rate” were set to 0.5. In general, the performance of the algorithm will improve as the amount of training data increases. Considering the memory size, running time, and accuracy, a total of 2,700,000 artificial contigs were generated to train PPR-Meta. The number of training contigs of each phage, chromosome, and plasmid was 300,000 from Group A to C.

We also used real metagenomic data to evaluate PPR-Meta and the related tools. We used SPAdes to assemble the raw reads, as we mentioned in the main text. The phage metagenomic data of the bovine rumen were downloaded from MG-RAST, and we used the command “spades.py –meta –1 file1.fastq –2 file2.fastq –o out_folder” to assemble the paired-end raw reads. In the assembly, the contig number, N50, average length, maximum length, and minimum length were 107,529, 288, 312.06, 75,508, and 56, respectively. To download the 20 samples of the healthy human gut, we used the command “prefetch SRRaccession” from the SRA Toolkit. All samples were downloaded as “.sra” files. We then used the command “fastq-dump –split-files accession.sra” from the SRA Toolkit to convert the sra file into 2 paired-end fastq files and used SPAdes with the aforementioned settings to assemble the raw reads. The information about the contig number, N50, average length, maximum length, and minimum length is provided in Additional File 1.

The artificial contigs are stored at [57].

## Availability of supporting source code and requirements

Project name: PPR-Meta.

Project home page: http://cqb.pku.edu.cn/ZhuLab/PPR_Meta or https://github.com/zhenchengfang/PPR-Meta.

Operating system: The code of PPR-Meta was written on Linux. We optimized the program in a virtual machine; thus, PPR-Meta is platform independent.

Programming language: python, matlab

Other requirements: no other requirements are needed if running in the virtual machine. If not, Python 2.7.12, TensorFlow 1.4.1, Keras 2.0.8, and MATLAB Component Runtime 2018a (for free) are needed. MATLAB is not necessary.

License: GPL-3.0.

RRID:SCR_016915

## Availability of supporting data and materials

The artificial contigs, related scripts, and original results are available at http://cqb.pku.edu.cn/ZhuLab/PPR_Meta/data/. All the other data are available at corresponding references mentioned in the main text. Snapshots of our code and other supporting data are available in the *GigaScience* repository, GigaDB [58].

## Additional files

**Additional file 1:** Accession list of the data used to train and test PPR-Meta.

**Additional file 2:** Prophage coordinates predicted by ProphET.

**Additional file 3:** Figure S1: Comparison of the performance of PPR-Meta and related tools using artificial contigs of 15k bp and 30k bp; Figure S2: Evaluation of the accuracy and running time of each group using BiPathCNNs from different groups; Figure S3: Identification performance of each tool with 10% base substitutions or 10% indels (insertions or deletions) using sequences in Group D. Figure S4: Recognition rate of prophages from PhAnToMe database; Figure S5: Performance of PPR-Meta under different thresholds.

giz066_GIGA-D-18-00464_Original_Submission

giz066_GIGA-D-18-00464_Revision_1

giz066_GIGA-D-18-00464_Revision_2

giz066_Response_to_Reviewer_Comments_Original_Submission

giz066_Response_to_Reviewer_Comments_Revision_1

giz066_Reviewer_1_Report_Original_Submission Pawel Krawczyk -- 12/13/2018 Reviewed

giz066_Reviewer_1_Report_Revision_1 Pawel Krawczyk -- 4/1/2019 Reviewed

giz066_Reviewer_2_Report_Original_Submission Deyvid Amgarten -- 12/23/2018 Reviewed

giz066_Reviewer_2_Report_Revision_1 Deyvid Amgarten -- 4/6/2019 Reviewed

giz066_Reviewer_3_Report_Original_Submission Corey Hudson -- 12/25/2018 Reviewed

giz066_Reviewer_3_Report_Revision_1 Corey Hudson -- 4/5/2019 Reviewed

giz066_Supplemental_Files

## Abbreviations

A: adenine; AUC: area under the curve; BiPathCNN: Bi-path Convolutional Neural Network; BOH: base one-hot matrix; bp: base pairs; C: cytosine; CNN: convolutional neural network; COH: codon one-hot matrix; CPU: central processing unit; FPR: false-positive rate; G: guanine; GB: gigabytes; GPU: graphics processing unit; HGT: horizontal gene transfer; kbp: kilobase pairs; MGE: mobile genetic element; NCBI: National Center for Biotechnology Information; ORF: open reading frame; PhAnToMe: Phage annotation, Tools, and Methods; PHAST: PHAge Search Tool; ProphET: Prophage Estimation Tool; ReLU: Rectified Linear Unit; ROC: receiver operating characteristic; rRNA: ribosomal RNA; SPAdes: St. Petersburg genome assembler; SRA: Sequence Read Archive; T: thymine; TPR: true-positive rate; WGS: whole-genome sequencing.

## Competing interests

The authors declare that they have no competing interests.

## Funding

This work was supported by the National Key Research and Development Program of China (2017YFC1200205), the National Natural Science Foundation of China (31671366), and the Special Research Project of “Clinical Medicine + X” by Peking University.

## Authors’ contributions

H.Q.Z. and Z.C.F. proposed and designed the study. Z.C.F., J.T., and S.F.W. constructed the datasets and wrote and optimized the code. M.L., C.M.X., and Z.J.X. tested the program. Z.C.F. and H.Q.Z. wrote and revised the manuscript, and all authors proofread and improved the manuscript.
