# Supplementary material for: PPR-Meta: a tool for identifying phages and plasmids from metagenomic fragments using deep learning
Source: Gigascience. 2019 Jun 20;8(6):giz066. doi: 10.1093/gigascience/giz066 (PMC6586199; doi:10.1093/gigascience/giz066)
Supplement: giz066_GIGA-D-18-00464_Original_Submission [file giz066_giga-d-18-00464_original_submission.pdf]

GigaScience

PPR-Meta: a tool for identifying phages and plasmids from metagenomic fragments using deep learning

--Manuscript Draft--

**Manuscript Number:**	GIGA-D-18-00464
**Full Title:**	PPR-Meta: a tool for identifying phages and plasmids from metagenomic fragments using deep learning
**Article Type:**	Technical Note
**Funding Information:**	Ministry of Science and Technology of the People's Republic of China (2017YFC1200205) — Dr Huaiqiu Zhu<br>National Natural Science Foundation of China (31671366) — Dr Huaiqiu Zhu<br>Peking University (None) — Dr Huaiqiu Zhu
**Abstract:**	Background: Phages and plasmids are the major components of mobile genetic elements, and fragments from such elements generally co-exist with chromosome-derived fragments in sequenced metagenomic data. However, there is a lack of efficient methods that can simultaneously identify phages and plasmids in metagenomic data, and the existing tools identifying either phages or plasmids have not yet presented satisfactory performances.<br>Findings: We present PPR-Meta, an ab initio tool that allows simultaneously identifying both phage and plasmid fragments from metagenomic sequences. PPR-Meta consists of several modules for predicting sequences of different lengths. Using deep learning, a novel network architecture, referred to as the Bi-path Convolutional Neural Network, is designed to improve the performance for short fragments. PPR-Meta demonstrates much better performance than currently available similar tools individually for phage or plasmid identification, while testing on both artificial contigs and real metagenomic data. PPR-Meta is freely available via http://cqb.pku.edu.cn/ZhuLab/PPR_Meta or https://github.com/zhenchengfang/PPR-Meta.<br>Conclusions: To the best of our knowledge, PPR-Meta is the first tool that can simultaneously identify phage and plasmid fragments efficiently and reliably. The software is optimized and can be easily run on a local PC by non-computer professionals. We developed PPR-Meta to promote the research on mobile genetic elements and horizontal gene transfer.
**Corresponding Author:**	Huaiqiu Zhu<br><br>CHINA
**Corresponding Author Secondary Information:**	
**Corresponding Author's Institution:**	
**Corresponding Author's Secondary Institution:**	
**First Author:**	Zhencheng Fang
**First Author Secondary Information:**	
**Order of Authors:**	Zhencheng Fang<br>Jie Tan<br>Shufang Wu<br>Mo Li<br>Congmin Xu<br>Zhongjie Xie

Powered by Editorial Manager® and ProduXion Manager® from Aries Systems Corporation

	Huaiqiu Zhu
**Order of Authors Secondary Information:**	
**Additional Information:**	
**Question**	**Response**
Are you submitting this manuscript to a special series or article collection?	No
**Experimental design and statistics**<br><br>Full details of the experimental design and statistical methods used should be given in the Methods section, as detailed in our Minimum Standards Reporting Checklist. Information essential to interpreting the data presented should be made available in the figure legends.<br><br>Have you included all the information requested in your manuscript?	Yes
**Resources**<br><br>A description of all resources used, including antibodies, cell lines, animals and software tools, with enough information to allow them to be uniquely identified, should be included in the Methods section. Authors are strongly encouraged to cite Research Resource Identifiers (RRIDs) for antibodies, model organisms and tools, where possible.<br><br>Have you included the information requested as detailed in our Minimum Standards Reporting Checklist?	Yes
**Availability of data and materials**<br><br>All datasets and code on which the conclusions of the paper rely must be either included in your submission or deposited in publicly available repositories (where available and ethically appropriate), referencing such data using a unique identifier in the references and in	Yes

the “Availability of Data and Materials” section of your manuscript.<br><br>Have you have met the above requirement as detailed in our Minimum Standards Reporting Checklist?	

Powered by Editorial Manager® and ProduXion Manager® from Aries Systems Corporation

Manuscript Click here to download Manuscript manuscript.docx

PPR-Meta: a tool for identifying phages and plasmids from metagenomic fragments using deep learning

Zhencheng Fang[1,2], Jie Tan[1,2], Shufang Wu[1,2], Mo Li[1,2,3], Congmin Xu[1,2,4], Zhongjie Xie[1,2] and Huaiqiu Zhu[1,2]*

[1] State Key Laboratory for Turbulence and Complex Systems and Department of Biomedical Engineering, College of Engineering, Peking University, Beijing 100871, China

[2] Center for Quantitative Biology, Peking University, Beijing 100871, China

[3] Peking University-Tsinghua University - National Institute of Biological Sciences (PTN) joint PhD program, School of Life Sciences, Peking University, Beijing 100871, China

[4] Department of Biomedical Engineering, Georgia Institute of Technology and Emory University, Georgia 30332, USA

* To whom correspondence should be addressed.

hqzhu@pku.edu.cn

Abstract

Background: Phages and plasmids are the major components of mobile genetic elements, and fragments from such elements generally co-exist with chromosome-derived fragments in sequenced metagenomic data. However, there is a lack of efficient methods that can simultaneously identify phages and plasmids in metagenomic data, and the existing tools identifying either phages or plasmids have not yet presented satisfactory performances.

Findings: We present PPR-Meta, an ab initio tool that allows simultaneously identifying both phage and plasmid fragments from metagenomic sequences. PPR-Meta consists of several modules for predicting sequences of different lengths. Using deep learning, a novel network architecture, referred to as the Bi-path Convolutional Neural Network, is designed to improve the performance for short fragments. PPR-Meta demonstrates much better performance than currently available similar tools individually for phage or plasmid identification, while testing on both artificial contigs and real metagenomic data. PPR-Meta is freely available via http://cqb.pku.edu.cn/ZhuLab/PPR_Meta or https://github.com/zhenchengfang/PPR-Meta.

Conclusions: To the best of our knowledge, PPR-Meta is the first tool that can simultaneously identify phage and plasmid fragments efficiently and reliably. The software is optimized and can be easily run on a local PC by non-computer professionals. We developed PPR-Meta to promote the research on mobile genetic elements and horizontal gene transfer.

Keywords: metagenome, mobile genetic elements, horizontal gene transfer, phage, plasmid, deep learning

Findings

Introduction

Phages and plasmids, known as mobile genetic elements (MGEs), are the main participants in horizontal gene transfer (HGT) along with genetic information exchanging among prokaryotes or eukaryotes [1]. Such elements can regulate the microbial community by interacting with the host. One of the important roles of MGEs is their ability to distribute resistance genes among bacteria and facilitate environmental adaptations among microbial communities [2]. In most cases, a substantial number of phage and plasmid genomes are present in the microbial community. For example, reports have shown that the abundance of marine phages even surpasses that of other organisms in marine systems, and more than half of the bacteria isolated from marine systems contain at least one plasmid [3][4]. Thus, the identification of phage and plasmid fragments in metagenomes is a fundamental issue in comprehensive analyses of HGT and the interaction between MGEs and hosts. Although experimental approaches have been developed to enrich phages or plasmids from environment samples [5][6], the enriched samples lose host information, which may hinder the comprehensiveness of the analyses. Therefore, computational tools for directly

identifying phages and plasmids from metagenomes are expected to be developed in the field.

However, the effective identification of such elements remains a considerable challenge. Currently the fragments assembly performance of both plasmid and phage from high-throughput sequencing data is not as well as that of host-derived fragments [7]. This indicates that sequences from phages or plasmids exist as a large number of short fragments, resulting in the difficulty of the identification. In addition, fewer sequenced genomes of phages and plasmids are available compared with bacterial genomes in current databases [1]. Especially, although the abundance of viruses is estimated to exceed that of other organisms on the earth [8], so far the number of phage genomes in the NCBI database is still less than one-thirtieth the number of prokaryotic genomes, and it was estimated that more than half of the sequences from viral metagenomes could not find significant homology to the released database [5]. Therefore, it is especially essential to develop a tool for identifying novel phages and plasmids from metagenomic data with a large number of mixed short reads.

Despite the difficulty of identification, several tools have been recently developed to detect either phages or plasmids from culture-dependent Whole Genome Sequencing (WGS) data or metagenomic data. Tools that detect regions from an integrated phage sequence (referred to as prophage) over a sequenced complete bacterial genome have been designed. These tools include Prophinder [9], Phage_Finder [10], PhiSpy [11], PHAST (and its

enhanced version PHASTER) [12] [13], VirSorter [14], and ProphET [15]. Such approaches primarily used a scan window to move across the complete bacterial chromosome and extracted regions that seem to be phages based on a similarity search against viral databases. Because the scan windows of these tools are often required to be able to cover several genes, such tools are difficult to apply to metagenomic data since the sequences of metagenome are too short to contain even a complete gene [16]. Although VirSorter can also assign metagenomic contigs as phages or bacteria, its sensitivity of identification is quite low. Moreover, lytic phages and some temperate phages do not integrate their genomes into their host chromosomes [17], thus these tools may only be able to identify specific phages. The tool MARVEL [18] can assign metagenomic bins as phages or bacteria, and its performance relies on the contig length and binning accuracy. However, research has shown that viral sequences are highly fragmented in the metagenome [19], which may prevent binning, thereby limiting the usage of MARVEL. In contrast, VirFinder [20] can directly judge each sequence, and it uses a logistic regression as the classifier to detect phage sequences based on *k*-mer frequencies and presents a relatively good performance. Compared with the other tools, VirFinder is more suitable for metagenomes. In terms of plasmids, most of the current tools for plasmid identification were designed for WGS or even specific species, such as PlasmidFinder [21], PLACNET [22] and PlasmidSeeker [23]. However, the plasmid identification strategy for WGS may not by applicable for metagenomes.

For example, PlasmidSeeker considers plasmid contigs to have a higher read coverage because plasmids may have copies in their hosts. In metagenome, however, the difference of read coverage among contigs may result from different abundances of species rather than copy number. The tool cBar [24] is the first tool designed primarily for plasmid identification in metagenomes. This tool applies SMO as a classifier based on *k*-mer frequencies. Similar to cBar, PlasFlow [25] is also a *k*-mer-based tool for identifying plasmids. Compared with cBar, PlasFlow further combines the information of different *k*-mer lengths and uses multiple neural networks as voting devices to determine whether the sequence belongs to the plasmid, and it achieves a better performance than cBar.

Although related tools have been developed, state-of-the-art tools for detecting short fragments have not presented satisfactory performances. Moreover, because these tools can only identify either phages or plasmids, they clearly do not meet the needs of a comprehensive analysis of MGEs and HGT. Considering that poor sequence assembly performance results in a large number of short fragments, it is a practical goal to develop a higher performing tool. In this paper, we present the PPR-Meta (**P**hage and **P**lasmid **R**ecognizer for **Meta**genomes) to identify metagenomic fragments as phages, plasmids or chromosomes based on the deep learning algorithm. To achieve higher performance on short fragments, we designed a novel neural network architecture which is referred to as the Bi-path Convolutional Neural Network

(BiPathCNN). To the best of our knowledge, PPR-Meta is the first tool that can simultaneously identify phage and plasmid fragments efficiently and reliably.

Dataset construction

Owing to that no suitable real metagenome datasets with confident annotation are available as a benchmark, we therefore used the simulated datasets with artificial contigs generated from sequenced complete genomes. We downloaded the complete genomes of prokaryote chromosomes (total of 10,090 genomes), prokaryote plasmids (total of 8,801 genomes) and phages (total of 2,279 genomes) from the NCBI genome database [26]. The list of the genomes is provided in Additional file 1. To evaluate the ability of PPR-Meta to identify novel species, genomes released before January 2016 were employed to build the training set while the remainder used to build the test set. In general, prokaryote chromosomes may contain regions of integrated phages, referred to as prophages [27], however most genomes do not have the prophage annotation. Here, we used ProphET to extract prophages from all the prokaryote chromosomes, and a total of 16,393 prophages predicted by ProphET (shown in Additional file 2) were incorporated into the phage dataset. Since the predicted prophages were generated by ProphET and could not be used as a benchmark, we removed the predicted prophages from the test set. To evaluate the performance of PPR-Meta for prophage identification, we collected 267 manually annotated prophages of 54 prokaryote chromosomes

from Casjens [27]. To ensure that the test data were “novel” to PPR-Meta, these prophages and their hosts were removed from the training set.

We used the MetaSim simulator [28] to extract artificial contigs from the complete genomes. Four groups of artificial contigs of different lengths were generated: Group A with a length range of 100-400 bp, Group B with a length range of 400-800 bp, Group C with a length range of 800-1200 bp and Group D with a length range of 5000-10000 bp. Group A, B and C were constructed to simulate the length obtained with different sequencing technology and the average assembly contig length, while Group D was constructed to simulate long contigs in metagenomic data. Additional details on the dataset construction are provided in Methods section.

We also used real metagenomic data to estimate the reliability of PPR-Meta. The real data included phage metagenomic data of bovine rumen [19], which were downloaded from MG-RAST [29] (Accessions: mgm4534202.3 and mgm4534203.3) as raw reads and assembled by SPAdes [30]; plasmid metagenomic data of bovine rumen [31], downloaded from MG-RAST (accessions: mgm4460391.3); and 20 samples of healthy human gut [32], downloaded from the NCBI Short Read Archive [33] and assembled by SPAdes. The accessions of the human gut samples are shown in Additional file 1.

Mathematical model of DNA sequences

The method of representing biological sequence is significant for every machine

learning-based tool. Although *k*-mer frequencies have been widely used in many studies [20], such frequencies may present serious fluctuations in short sequences [34]. Here, we use a more detailed approach to represent the short sequences in Group A, Group B and Group C. Specifically, each sequence is represented by "base one-hot matrix (BOH)" and "codon one-hot matrix (COH)". For BOH, bases A, C, G and T are represented by [0,0,0,1], [0,0,1,0], [0,1,0,0], and [1,0,0,0], respectively. Therefore, together with the complementary strand, a sequence of length L can be represented by a BOH matrix of length 2×L and width 4. For COH, each sequence is first expanded to six phases in the form of codons. For example, sequence 5'-ACGTTCGAACG-3' will be split into the following six codon sequences:

1): ACG, TTC, GAA

2): CGT, TCG, AAC

3): GTT, CGA, ACG

4): CGT, TCG, AAC

5): GTT, CGA, ACG

6): TTC, GAA, CGT

Similar to BOH, each codon of COH is represented by a 64 dimensional one-hot vector, namely one certain position is 1 and the other positions are 0. Therefore, a sequence of length L can be represented by a COH matrix of length 2×L and width 64. Both BOH and COH will be used as input for the neural networks mentioned below.

Since sequences from Group D are long contigs and *k*-mer frequencies have already been shown to generate good representations of long sequences, to save computing resources, we used 6-mer frequencies to represent sequences from Group D.

Structure of deep learning neural networks

To ensure that PPR-Meta optimally adapts to sequences of different lengths, we trained corresponding neural networks for each group. For Group A, B and C, we designed BiPathCNN to improve the performance (Figure 1). BiPathCNN contains a “base path” and a “codon path”, which take BOH and COH as inputs respectively. After multiple convolution operations, the data for the two paths are combined by a merge layer. The fully connected layers then receive the merged data and finally output three scores that reflect the likelihood of the input fragment as a phage, chromosome or plasmid.

Figure 1. Structure of BiPathCNN. Three BiPathCNNs were trained for sequences from Group A, B and C. Each BiPathCNN contains a “base path” and a “codon path”, which take BOH and COH as inputs respectively.

The details of each layer are described as follows.

Layer b1 and layer c1: one-dimensional convolutional layers with 64 convolution kernels using “ReLU” (Rectified Linear Unit) as the activation

function. The ReLU function can be expressed as y=max(0,x). These layers take BOH or COH as inputs. The length of the convolution kernels is set to 6.

Layer b2 and layer c2: max pooling layers with a pooling length set to 3.

Layer b3 and layer c3: batch normalization layers with the dropout operation. Each element of the feature map from previous layer in each batch will be normalized, which can speed up the convergence and prevent overfitting.

Layer b4~b6 and layer c4~c6: similar to layers b1~b3 or layers c1~c3, respectively. We set the number of convolution kernels in layer b4 and c4 as 128 and the length of the kernels as 3.

Layer b7 and layer c7: one-dimensional convolutional layers containing 256 convolution kernels and using ReLU as the activation function. The length of the convolution kernels is set to 3.

Layer b8 and layer c8: one-dimensional global average pooling layers that output the global average for each feature map of the previous layer.

Layer 9 to layer 11: The concatenation layers combine the output of the “base path” and “codon path”. After the full connection layer with the same number of nodes as the previous layer, the softmax layer calculates the probability of the input fragment as a phage, chromosome or plasmid.

For Group D, a Fully Connected Neural Network (FNN) was used (Figure 2). The FNN takes the *k*-mer frequencies as inputs. During the training phase, Gaussian noise was first added to the *k*-mer frequencies to improve the robustness of the network. The modified *k*-mer then goes through a batch

normalization layer and five full connection layers with dropout operations. Finally, the FNN outputs three scores reflecting the likelihood of obtaining a phage, chromosome or plasmid. All the neural networks used Adam as the optimizer and cross-entropy as the loss function.

In practical applications, we use the neural network of Group A to predict sequences shorter than 100 bp and the neural network of Group D to predict sequences longer than 10k bp. For sequences between 1200 and 5000 bp, a scan window will move across the sequence without overlapping and the weighted average of all windows' predictions is calculated. The length of the window is set to 1200 bp (or less if the window is beyond the sequence boundary).

Figure 2. Structure of the FNN. The FNN was trained for sequences from Group D. It takes the 6-mer frequencies as input.

Overall performance

We evaluated PPR-Meta according to four groups of test sets with different lengths of short contigs. For each fragment input, the algorithm calculates three scores representing the likelihood that the fragment should be identified as a phage, plasmid or chromosome. Therefore, the category with the highest score is selected as our prediction. We used three-class confusion matrices (shown in Figure 3) to evaluate the overall performance of PPR-Meta. In general, PPR-

Meta had a better discrimination ability when the sequences were longer, and the phage recognition ability of PPR-Meta was better than the plasmid recognition ability. Plasmid sequences were easily confused with the host chromosomes, which may because phages and plasmids face different evolutionary pressures. Since plasmid must survive in host cells, they may adapt their sequence signatures, such as the GC content and codon usage, to their hosts. In contrast, phages can assemble their own particles and remain outside of the hosts. Moreover, certain phages may contain their own tRNA, which allows them to change their codon usage [35]. Thus, the various similarity of phages and plasmids to their hosts may lead to differences in the identification ability of PPR-Meta. In addition, transposons may carry plasmid DNA fragments to the chromosome [1]. Therefore, the chromosome may contain regions from the plasmid. Sequences shared between the plasmid and the chromosome may also affect the judgment of PPR-Meta. Overall, PPR-Meta can effectively identify the MGEs in the test set.

Figure 3. Confusion matrix of PPR-Meta. Three-class confusion matrices were used to evaluate the overall performance of PPR-Meta. Four matrices correspond to the sequences of Group A to D. In each matrix, the rows represent the true category while the column represent the predicted category of PPR-Meta.

Performance comparison

We then compare PPR-Meta with VirFinder and VirSorter regarding the ability to identify phages, and with PlasFlow and cBar regarding the ability to identify plasmids. The evaluation criteria were the true positive rate (TPR=TP/(TP+FN)), false positive rate (FPR=FP/(TN+FP)) and area under the curve (AUC). Note that PlasFlow will filter uncertain predictions according to a default threshold. As a uniform comparison, we turned off this feature by setting the threshold to zero, thus using all the sequences for comparison.

The results are shown in Table 1. In all cases, the AUCs of PPR-Meta were the highest. In term of phages, VirSorter, which is a gene-based tool, performed poorly with almost all phages missed. It is probably because there is not a sufficient number of full-length genes present in short DNA fragments for VirSorter's analysis. This also indicates that methods based on homology searches of genetic information are not applicable to species identification in metagenomes. Considering that most contigs of metagenomes are short fragments especially those of MGEs, VirSorter is not competent for phage identification despite achieving a higher performance for long contigs in Group D. The tool VirFinder outperformed VirSorter. As an alignment-free tool, VirFinder achieved a much higher TPR, especially in short fragments. The TPR of PPR-Meta was approximately 3%~13% higher than that of VirFinder and the FPR was approximately 6%~9% lower. The performance improvement on short sequences demonstrates that our sequences representation method is more

detailed than the *k*-mer frequencies, and the deep learning algorithm is more capable of extracting sequence features than the logistic regression used by VirFinder. In terms of plasmids, both cBar and PlasFlow did not perform well. The cBar appearing to produce random results in most cases, with both the TPR and FPR near 50%. Although the AUC of PlasFlow was slightly higher than that of cBar, PlasFlow tended to judge most sequences as plasmids, which resulted in an extremely high FPR. For PPR-Meta, our FPR was much lower than that of cBar and PlasFlow. Although PPR-Meta achieved a slightly lower TPR than PlasFlow in Group A, our TPR remained highest in all other cases. For long sequences in Group D, although both PPR-Meta and PlasFlow applied fully connected network as the classifier based on *k*-mer frequencies, the performance of PPR-Meta was obviously better than that of PlasFlow. This is likely because that we have used more layers in the neural network and applied methods of optimizing the network, such as the usage of batch normalization.

Table 1. Evaluation of the performance of PPR-Meta and comparison of the performance of PPR-Meta and related tools.

Effectiveness of BiPathCNN

PPR-Meta achieved a much higher performance than the other methods as mentioned above. The innovation of PPR-Meta is the design of BiPathCNN, which uses both base and codon information to improve the performance. In

BiPathCNN, the “base path” is beneficial to extracting the sequence features of non-coding regions while the “codon path” is beneficial to extracting coding regions. To verify the effectiveness of BiPathCNN, we removed the “codon path” and “base path” and retrained PPR-Meta. The newly trained PPR-Meta was tested, and the results showed that the performance of PPR-Meta with either the “base path” or “codon path” only presented a lower performance relative to that of BiPathCNN in most cases (Table 2). Moreover, the performance of the “codon path” only CNN was better than that of “base path” only CNN, which indicates that the features that distinguish phages, chromosomes and plasmids are more concentrated in the coding region. Compared with other sequence representation methods that ignore the coding or non-coding region, such as method based on *k*-mer frequencies, PPR-Meta uses a more detailed method of describing a sequence and achieves a higher performance.

Table 2. Performance comparison among BiPathCNN, the base path-only CNN and codon path-only CNN.

Performance in the presence of sequencing errors

Sequencing errors exist in various sequencing technologies, and tools that handle high-throughput sequencing data should be able to tolerate these errors. In addition, the third-generation sequencing technology, such as PacBio and Nanopore, have much higher sequencing errors. Thus, the compatibility of tools

with new sequencing technologies should be considered.

Sequencing errors can be divided into two types: base substitutions and base insertions or deletions. We tested the impact of these two types of sequencing errors on the identification performance of PPR-Meta and related tools. We used MetaSim to extract modified fragments with 1% substitutions and 1% insertions or deletions separately from the test genomes. We used the same criteria described above to compare the performance of different tools in terms of both types of error. The results are shown in Table 3 and Table 4.

In most cases, in the presence of 1% base substitutions, a slight decrease in each evaluation criterion was observed for each tool compared with that in the presence of non-sequencing errors, although the decrease was not obvious. PPR-Meta was still the best-performing tool. When 1% of the bases were inserted or deleted, the performance of most tools was slightly reduced with the exception of VirSorter. Base insertions or deletions caused significant fluctuations in the performance of VirSorter. For sequences of Group D, the AUC of VirSorter decreased by approximately 9% compared with sequences with no errors. In our opinion, the reason that VirSorter exhibits great fluctuations in performance with base insertions or deletions is that insertions and deletions disrupt the phase of the open reading frame (ORF). VirSorter identifies phage sequences primarily by observing the distribution of genes, such as the densities of known viral genes or the enrichment of short genes. Disrupting the ORF phase will severely affect gene identification [36], thereby

leading to interference in the downstream analysis. Thus, although VirSorter can achieve a relatively good performance on long contigs, caution should be taken when applying VirSorter to data generated by third-generation sequencing technology.

Table 3. Identification performance of each tool with 1% base substitutions.

Table 4. Identification performance of each tool with 1% base insertions or deletions.

Prophage identification ability

We tested the prophage identification ability of the related tools on the 267 manually annotated prophages. The results in Table 5 showed that although the recognition rate of PPR-Meta for prophages was lower than that for phage contigs generated from the NCBI database, the overall performance of PPR-Meta was still much better than that of VirFinder and VirSorter. The decline in performance may be due to the difference of sequence pattern between prophages and phages in the NCBI database. The complete genomes in the NCBI database tend to come from phages that are easily obtained experimentally while prophages hide their genomes in the hosts. During co-evolution, prophages may adjust the sequence pattern according to their hosts to eliminate the hosts' restriction enzymes [35]. In terms of VirFinder, the ability

of identifying prophages was significantly reduced and more than half of the prophages were missed, which may be because VirFinder ignored prophages that exist in the chromosomes during training. In both the training and test set of VirFinder, all prophages were labelled as chromosomes, which led to the misjudgement of prophages. In microbial communities, temperate phages are dominant and a significant portion of temperate phages exist in the form of prophages [37]. For example, prophage have been shown to represent the main component of phages in healthy human guts [17]. In certain prokaryotes, prophages account for up to 20% of the host chromosome [27]. Thus, compared with VirFinder, PPR-Meta may be more adapted to real microbial communities since it can identify more prophages.

Table 5. Recognition rate of prophages

Evaluation in real metagenomic data

We also evaluated PPR-Meta and the related tools using real metagenomic data. We first evaluated whether PPR-Meta can identify MGEs using both phage metagenomic and plasmid metagenomic data of bovine rumens, in which either phages or plasmids were enriched before sequencing. The phage metagenomic data were downloaded as raw reads, and a total of 107,529 contigs were generated after assembly. VirSorter, VirFinder and PPR-Meta were run on the phage metagenome. Consistent with the results for artificial

contigs, VirSorter missed nearly all the phages and only 0.02% of the contigs were identified. VirFinder and PPR-Meta were much better than VirSorter and identified 68.86% and 76.88% of the contigs, respectively, showing that PPR-Meta had the highest coverage of this data set.

The plasmid metagenomic data were downloaded as assembled contigs containing 5771 sequences. It is worth noting that there are a certain number of phages survive as circular DNA [17], and when enriching plasmids, these circular phages will also be extracted together with the plasmids. Thus, the plasmid metagenome may contain a mixture of phages and plasmids in which the host chromosomes are filtered. From the RefSeq viral database, we collected the genes labelled as “portal”, “spike”, “major capsid protein”, “terminase large subunit”, “tail”, “coat”, or “virion formation”, which were more likely to exist in phages [14]. We found that one of the sequences contained a homologous region of the portal protein by applying the blastx search (e-value<=1e-4), indicating that phages are likely to co-exist with plasmids in this dataset. Therefore, all of PPR-Meta, VirSorter, VirFinder, cBar and PlasFlow were run on this dataset. Results showed that VirSorter did not identify any sequences as phages while VirFinder identified 49.90% as phages. In term of cBar and PlasFlow, they identified 64.46% and 74.67% of the sequences as plasmids. For PPR-Meta, total of 82.00% of the sequences were identified as MGEs, in which 49.16% were phages and 32.84% were plasmids. More than half of the sequences (64.72%) predicted as phages by PPR-Meta were also

predicted as phages by VirFinder, and most of the sequences (74.72%) predicted as plasmids by PPR-Meta were also predicted by PlasFlow. Furthermore, the sequence containing the homologous region of the portal protein was identified as phages and 8 out of 10 sequences coding plasmid backbone functions listed in Figure 3 of [31] were also identified as plasmids by PPR-Meta. Thus, the prediction of PPR-Meta may be reliable. Because of the filtering of chromosomes from this dataset, PPR-Meta could identify most of the extrachromosomal elements with the fewest false negative predictions.

Since we lack samples in which only chromosomes are enriched and all the extrachromosomal elements are filtered, estimating whether related tools will misjudge chromosomes as MGEs directly is difficult using real data. Because 16S rRNA is more likely to occur in chromosomes, sequences containing the homologous region of 16S rRNA are likely chromosome-derived. We collected 20 metagenome samples from the human gut, which represented mixtures of phages, chromosomes and plasmids. All contigs of the samples were searched against the 16S rRNA database of Greengeen [38] using blastn, and the contigs containing the homologous region (e-value<=1e-4, hits length>=250) of 16S rRNA were collected. Hits longer than 250 bp could cover at least one conserved region of 16S rRNA so the alignments were reliable. In terms of phage identification, PPR-Meta, VirFinder and VirSorter predicted an average of 4.18%, 11.03% and 0% of the 16S-like contigs as phages, respectively, indicating that PPR-Meta likely generated fewer false positive predictions than

VirFinder. Although VirSorter did not cover any of the 16S-like contigs, the low number of false positive predictions came at the cost of missing almost all phages as shown above. In terms of plasmid identification, PPR-Meta, PlasFlow and cBar predicted an average of 25.46%, 53.74% and 63.83% of the 16S-like contigs as plasmids, respectively, indicating that the PPR-Meta may generate the lowest number of false positive predictions. Because individual extrachromosomal elements also contain ribosomal RNA, especially large plasmids [39], the coverage of 16S-like contigs may be higher than the real FPR. Overall, PPR-Meta can identify more MGEs with fewer false positives.

Usage of PPR-Meta

PPR-Meta takes the sequence file in fasta format as input and outputs a tabular file. The output file contains three scores between 0 and 1 that reflect the likelihood of obtaining phages, chromosomes and plasmids for each sequence. By default, the final prediction is the category with the highest score. To meet users' actual requirements, PPR-Meta is designed with the option to adjust the default threshold of discriminant criteria. In general, with a higher threshold, both the TPR and FPR will be lower. The performance of PPR-Meta under different thresholds are provided in Additional file 3.

PPR-Meta is user friendly, and the program has been optimized in a virtual machine so that users can directly run PPR-Meta without installing any dependency package. We also provided a short video guide to show how to

install the virtual machine. If users are analysing large-scale data, running the executable file on the physical host is more suitable. In this way, when the GPU is available, PPR-Meta will run on the GPU automatically to speed up the program. The memory requirements are dependent on the data size. We recommend at least 4~6 GB of available memory when running the virtual machine or at least 16 GB when handling large-scale data on the physical host. We tested the running time of PPR-Meta using 90,000 sequences from 100 to 10k bp and found that this tool can handle all sequences in approximately 15 minutes on a machine with the following configuration: CPU: Intel Core i7 6700; GPU: NVIDIA GTX1060; and Memory: 64G, DDR4.

Discussion and conclusions

In this paper, we proposed an ab initio method, PPR-Meta, to identify both phages and plasmids from metagenomic sequences. PPR-Meta employs a novel strategy to improve the MGE identification performance and avoids performing similarity searches to make judgments. Similarity search-based tools, such as VirSorter, provide good results for long sequences. However, such methods do not work effectively for short fragments due to the insufficient number of genes for the statistical analysis. Compared with other reference-free tools, PPR-Meta employs a more detailed method of characterizing DNA sequences. We use a BOH matrix, which is beneficial to non-coding regions, and a COH matrix, which is beneficial to coding regions, to represent

sequences. In contrast, traditional k-mer methods do not consider coding or non-coding regions. When the sequence is short, k-mer frequencies will be noisy. Moreover, mapping sequences of different length for k-mer feature vectors with the same dimension will also lose some information. PPR-Meta takes all bases and codons as inputs in the neural network, thereby exploiting all information in the fragments. In the design of the algorithm, we employed a deep learning network as the classifier. Deep learning has achieved great success in many fields, such as lncRNA identification [40] and the prediction of sequence specificities of nucleic acid binding proteins [41]. In the construction of PPR-Meta, we designed the BiPathCNN, which contains a “base path” and “codon path” to handle the BOH matrix and COH matrix, respectively. Testing showed that the performance of the CNN with double paths was better than that with single path.

Furthermore, we were surprised to find that PPR-Meta’s output scores were able to describe the interaction between phages or plasmids and their hosts. Specifically, the difference between the phage score and chromosome score reveals the lifestyle of the phages (virulent or temperate), while the difference between the plasmid score and chromosome score reveals the transmissibility of the plasmids (transmissible or non-transmissible). We collected both phage genomes with lifestyle annotations from McNair et al. [8] and plasmid genomes with transmissibility annotations from Shintani et al. [42] and then extracted artificial contigs. PPR-Meta was run on all the contigs and

the correctly predicted contigs were retained. From the results, two normalized statistics were constructed:

life_score=(*phage_score*-*chromosome_score*)/*phage_score*

and

trans_score=(*plasmid_score*-*chromosome_score*)/*plasmid_score*

The receiver operating characteristic curve (ROC) showed that life_score could distinguish the lifestyle of phages while trans_score could distinguish the transmissibility of plasmids with AUC values higher than 0.5 (shown in Figure 4). Specifically, temperate phages tend to have lower life_score values and non-transmissible plasmids tend to have lower trans_score values. This phenomenon may be due to the adaptation of foreign DNA to the host. Since temperate phages and non-transmissible plasmids experience longer residence times within the host cell, they may adjust the sequence pattern toward the host. Thus, the sequence pattern between temperate phages (or non-transmissible plasmids) and host chromosomes may be more similar than that between virulent phages (or transmissible plasmids) and host chromosomes, thereby resulting in a lower life_score (or trans_score) value. Although tools that can classify phage lifestyle and plasmid transmissibility on metagenomes are lacking as far as we know, the phenomena mentioned above may provide insights into the classification strategy for future studies.

Figure 4. ROC curve of classifying phage lifestyle and plasmid

transmissibility. (a) Classify virulent phages and temperate phages using life_score. In order of sequence length, the AUC is 0.63, 0.69, 0.71 and 0.82. (b) Classify transmissible plasmid and non-transmissible plasmid using trans_score. In order of sequence length, the AUC is 0.58, 0.55, 0.60 and 0.66.

PPR-Meta also has some limitations. In addition to prokaryote chromosomes, plasmids and phages, other organisms of low-abundance may exist in the microbial community, such as fungi and protozoan. Such organisms are not included in the training set of PPR-Meta and may have interfered with the judgment of PPR-Meta. To increase the suitability of PPR-Meta for real scenes, we will retrain PPR-Meta regularly with expanded datasets. More organisms as well as the newly sequencing genomes will be added to the dataset so that PPR-Meta will be more powerful and reliable. In addition, due to sequence exchanges among phages, plasmids and chromosomes, there are a few chimeric sequences from two sources (e.g., a prophage and chromosome chimera). PPR-Meta cannot perform detailed judgments about these chimeras, and we are considering how to further identify such sequences. However, because these chimeras do not exist at a large scale, we believe that the presence of chimeras will not have a significant impact on the application of PPR-Meta.

In conclusion, the performance of PPR-Meta has shown much better than that of currently available similar tools, while none of these tools can function

as PPR-Meta does. It is thus expected that the PPR-Meta tool would meet the demand of metagenomics analysis when considered the microbial community tangled with phages and plasmids, and certainly qualifies as a powerful tool for the research community.

Methods

PPR-Meta was trained and tested using artificial contigs. We downloaded the accession list of prokaryote chromosomes, prokaryote plasmids and phages from the NCBI genome database, and the corresponding genomes were downloaded according to the list. To ensure the quality of the data, we only used the complete genomic molecule with the RefSeq accession prefix. Since chromosomes may contain prophages, we used ProphET to extract the prophage regions of all chromosomes. ProphET requires a genome sequence file (fasta format) and genome annotation file (gff format) as inputs. A few genomes do not contain the annotation information, and these genomes were removed from the dataset. We then used MetaSim to generate four groups of artificial contigs with different lengths as mentioned in the main text. The sequence length of each group obeyed a uniform distribution. In general, the performance of the algorithm will be better as the amount of training data increases. Considering the memory size, running time and accuracy, a total of 3,060,000 artificial contigs were generated to train PPR-Meta. The number of training contigs of each phage, chromosome and plasmid is 300,000 from

Group A to C and 120,000 from Group D. The artificial contigs are stored at http://cqb.pku.edu.cn/ZhuLab/PPR_Meta/data.zip.

Availability of supporting source code and requirements

Project name: PPR-Meta.

Project home page: http://cqb.pku.edu.cn/ZhuLab/PPR_Meta or https://github.com/zhenchengfang/PPR-Meta.

Operating system: The code of PPR-Meta was written on Linux. We optimized the program in a virtual machine thus PPR-Meta is platform independent.

Programming language: python, matlab

Other requirements: no other requirements are needed if running in the virtual machine. If not, Python 2.7.12, TensorFlow 1.4.1, Keras 2.0.8 and MATLAB Component Runtime 2018a (for free) are needed. MATLAB is not necessary.

License: GPL-3.0.

Availability of supporting data

The artificial contigs are available at http://cqb.pku.edu.cn/ZhuLab/PPR_Meta/data.zip. All the other data are available at corresponding references mentioned in the main text.

Additional file

Additional file 1: accession list of the data used to train and test PPR-Meta.

Additional file 2: prophage coordinate predicted by ProphET.

Additional file 3: PPR-Meta performance under different thresholds.

List of abbreviations

MGEs: mobile genetic elements

HGT: horizontal gene transfer

WGS: Whole Genome Sequencing

BiPathCNN: Bi-path Convolutional Neural Network

BOH: base one-hot matrix

COH: codon one-hot matrix

FNN: Fully Connected Neural Network

TPR: true positive rate

FPR: false positive rate

AUC: area under curve

ROC: receiver operating characteristic

Ethics approved and consent to participate

Not applicable

Consent for publication

Not applicable

Competing interests

The authors declare that they have no competing interests

Funding

This work was supported by the National Key Research and Development Program of China (2017YFC1200205), the National Natural Science Foundation of China (31671366), and the Special Research Project of ‘Clinical Medicine + X’ by PKU.

Authors' contributions

HQZ and ZCF proposed and designed the study. ZCF, JT and SFW constructed the data sets, and wrote and optimized the code. ML, CMX and ZJX tested the program. ZCF and HQZ wrote and revised the manuscript and all authors proofread and improved the manuscript.

Acknowledgements

We thank Dr. Cheng Yang, Dr. Longshu Yang, Dr. Xiaoqing Jiang, Li Qu of Peking University for their helpful discussions.

References

[1] Frost LS, Leplae R, Summers AO, Toussaint A. Mobile genetic elements: the agents of open source evolution. *Nat. Rev. Microbiol.*, 2005;3(9): 722.

[2] Brown-Jaque M, Calero-Cáceres W, Muniesa M. Transfer of antibiotic-resistance genes via phage-related mobile elements. *Plasmid*, 2015;*79*: 1-7.

[3] Paul JH, Sullivan MB. Marine phage genomics: what have we learned?. *Curr. Opin. Biotechnol.*, 2005:16(3): 299-307.

[4] Sobecky PA, Hazen TH. Horizontal gene transfer and mobile genetic elements in marine systems. In *Horizontal Gene Transfer*, 2009; 435-453, Humana Press.

[5] Hayes S, Mahony J, Nauta A, van Sinderen D. Metagenomic approaches to assess bacteriophages in various environmental niches. *Viruses*, 2017; 9(6): 127.

[6] Li LL., Norman A, Hansen LH, Sørensen SJ. Metamobilomics-expanding our knowledge on the pool of plasmid encoded traits in natural environments using high-throughput sequencing. *Clin. Microbiol. Infect.*, 2012: 18, 5-7.

[7] Rozov R, Brown Kav A, Bogumil D, Shterzer N, Halperin E, Mizrahi I, Shamir R. Recycler: an algorithm for detecting plasmids from de novo assembly graphs. *Bioinformatics*, 2017; 33(4): 475-482.

[8] McNair K, Bailey BA, Edwards RA. PHACTS, a computational approach to classifying the lifestyle of phages. *Bioinformatics*, 2012; 28(5): 614-618.

[9] Lima-Mendez G, Van Helden J, Toussaint A, Leplae R. Prophinder: a computational tool for prophage prediction in prokaryotic genomes. *Bioinformatics*, 2008; 24(6): 863-865.

[10] Fouts DE. Phage_Finder: automated identification and classification of

prophage regions in complete bacterial genome sequences. *Nucleic Acids Res.*, 2006; 34(20): 5839-5851.

[11] Akhter S, Aziz RK, Edwards RA. PhiSpy: a novel algorithm for finding prophages in bacterial genomes that combines similarity-and composition-based strategies. *Nucleic Acids Res.*, 2012; 40(16): e126-e126.

[12] Zhou Y, Liang Y, Lynch KH, Dennis JJ, Wishart DS. PHAST: a fast phage search tool. *Nucleic Acids Res.*, 2011; 39(suppl_2): W347-W352.

[13] Arndt D, Grant JR, Marcu A, Sajed T, Pon A, Liang Y, Wishart DS. PHASTER: a better, faster version of the PHAST phage search tool. *Nucleic Acids Res.*, 2016; 44(W1): W16-W21.

[14] Roux S, Enault F, Hurwitz B L, Sullivan MB. VirSorter: mining viral signal from microbial genomic data. *PeerJ*, 2015; 3: e985.

[15] Reis-Cunha JL., Bartholomeu DC, Earl AM, Birren BW, Cerqueira GC. ProphET, Prophage Estimation Tool: a standalone prophage sequence prediction tool with self-updating reference database. 2017; *bioRxiv*, 176750.

[16] Liu Y, Guo J, Hu G, Zhu H. Gene prediction in metagenomic fragments based on the SVM algorithm. In *BMC Bioinformatics*. 2013; 14(5): S12.

[17] Mirzaei MK, Maurice CF. Ménage à trois in the human gut: interactions between host, bacteria and phages. *Nat. Rev. Microbiol.*, 2017; 15(7), 397.

[18] Amgarten DE, Braga LPP, Da Silva AM, Setubal JC. MARVEL, a Tool for Prediction of Bacteriophage Sequences in Metagenomic Bins. *Front. Genet.*, 2018; 9: 304.

[19] Ross EM, Petrovski S, Moate PJ, Hayes BJ. Metagenomics of rumen bacteriophage from thirteen lactating dairy cattle. *BMC Microbiol.*, 2013; 13(1): 242.

[20] Ren J, Ahlgren NA, Lu YY, Fuhrman JA, Sun F. VirFinder: a novel k-mer based tool for identifying viral sequences from assembled metagenomic data. *Microbiome*, 2017; 5(1): 69.

[21] Carattoli A, Zankari E, García-Fernández A, Larsen MV, Lund O, Villa L, Aarestrup FM, Hasman H. In silico detection and typing of plasmids using PlasmidFinder and plasmid multilocus sequence typing. *Antimicrob. Agents Chemother.*, 2014; 58(7): 3895-3903.

[22] Lanza VF, de Toro M, Garcillán-Barcia MP, Mora A, Blanco J, Coque TM, de la Cruz F. Plasmid flux in Escherichia coli ST131 sublineages, analyzed by plasmid constellation network (PLACNET), a new method for plasmid reconstruction from whole genome sequences. *PLoS Genet.*, 2014; 10(12): e1004766.

[23] Roosaare M, Puustusmaa M, Möls M, Vaher M, Remm M. PlasmidSeeker: identification of known plasmids from bacterial whole genome sequencing reads. 2018; *PeerJ*, 6: e4588.

[24] Zhou F, Xu Y. cBar: a computer program to distinguish plasmid-derived from chromosome-derived sequence fragments in metagenomics data. *Bioinformatics*, 2010; 26(16): 2051-2052.

[25] Krawczyk PS, Lipinski L, Dziembowski, A. PlasFlow: predicting plasmid

sequences in metagenomic data using genome signatures. *Nucleic Acids Res.*, 2018; 46(6): e35-e35.

[26] The NCBI genome database. ftp://ftp.ncbi.nlm.nih.gov/genomes/GENOME_REPORTS/. Accessed 12 July 2018.

[27] Casjens S. Prophages and bacterial genomics: what have we learned so far?. *Mol. Microbiol.*, 2003; 49(2): 277-300.

[28] Richter DC, Ott F, Auch AF, Schmid R, Huson DH. MetaSim—a sequencing simulator for genomics and metagenomics. *PLoS One*, 2008; 3(10): e3373.

[29] Meyer F, Paarmann D, D'Souza M, Olson R, Glass EM, Kubal M, Paczian T, Rodriguez A, Stevens R, Wilke A, Wilkening J. The metagenomics RAST server–a public resource for the automatic phylogenetic and functional analysis of metagenomes. *BMC Bioinformatics.* 2008; 9(1): 386.

[30] Bankevich A, Nurk S, Antipov D, Gurevich AA, Dvorkin M, Kulikov AS, Lesin VM, Nikolenko SI, Pham S, Prjibelski AD, Pyshkin AV. SPAdes: a new genome assembly algorithm and its applications to single-cell sequencing. *J. Comput. Biol.*, 2012; 19(5): 455-477.

[31] Kav AB, Sasson G, Jami E, Doron-Faigenboim A, Benhar I, Mizrahi I. Insights into the bovine rumen plasmidome. *Proc. Natl. Acad. Sci. U S A*, 2012; 109(14): 5452-5457.

[32] Qin J, et al. A metagenome-wide association study of gut microbiota in type 2 diabetes. *Nature*, 2012; 490(7418): 55-60.

[33] NCBI Short Read Archive. https://www.ncbi.nlm.nih.gov/sra/. Accessed 12 July 2018.

[34] Galiez C, Siebert M, Enault F, Vincent J, Söding J. WIsH: who is the host? Predicting prokaryotic hosts from metagenomic phage contigs. *Bioinformatics*, 2017; 33(19): 3113-3114.

[35] Edwards RA, McNair K, Faust K, Raes J, Dutilh BE. Computational approaches to predict bacteriophage–host relationships. *FEMS Microbiol. Rev.*, 2015; 40(2): 258-272.

[36] Rho M, Tang H, Ye Y. FragGeneScan: predicting genes in short and error-prone reads. *Nucleic Acids Res.*, 2010; 38(20): e191-e191.

[37] Hurwitz BL, Ponsero A, Thornton J, U'Ren JM. Phage Hunters: computational strategies for finding phages in large-scale 'omics datasets. *Virus Res.*, 2018; 244(15): 110-115.

[38] DeSantis TZ, Hugenholtz P, Larsen N, Rojas M, Brodie EL, Keller K, Huber T, Dalevi D, Hu P, Andersen GL. Greengenes, a chimera-checked 16S rRNA gene database and workbench compatible with ARB. *Appl. Environ. Microbiol.*, 2006; 72(7): 5069-5072.

[39] Smillie C, Garcillán-Barcia MP, Francia MV, Rocha EP, de la Cruz F. Mobility of plasmids. *Microbiol. Mol. Biol. R.*, 2010; 74(3): 434-452.

[40] Yang C, Yang L, Zhou M, Xie H, Zhang C, Wang MD, Zhu H. LncADeep: An ab initio lncRNA identification and functional annotation tool based on deep learning. *Bioinformatics*., 2018; 34(22): 3825-3843.

[41] Alipanahi B, Delong A, Weirauch MT, Frey BJ. Predicting the sequence specificities of DNA-and RNA-binding proteins by deep learning. *Nat. Biotechnol.*, 2015; 33(8): 831-838.

[42] Shintani M, Sanchez ZK, Kimbara K. Genomics of microbial plasmids: classification and identification based on replication and transfer systems and host taxonomy. *Front. Microbiol.*, 2015; 6:242.

Table

Table 1. Evaluation of the performance of PPR-Meta and comparison of the performance of PPR-Meta and related tools.

Group	Tool	Evaluation on phage			Evaluation on plasmid		
		TPR(%)	*FPR*(%)	*AUC*(%)	*TPR*(%)	*FPR*(%)	*AUC*(%)
Group A 100-400 bp	**PPR-Meta**	**84.96**	18.01	**91.82**	59.91	**14.14**	**83.05**
	VirFinder	73.77	25.45	81.30	NA	NA	NA
	VirSorter	0.00	**0.00**	50.00	NA	NA	NA
	PlasFlow	NA	NA	NA	**71.89**	62.59	56.30
	cBar	NA	NA	NA	52.68	46.07	53.31
Group B 400-800 bp	**PPR-Meta**	**90.75**	8.37	**97.21**	**74.56**	**13.37**	**89.64**
	VirFinder	79.27	18.15	88.64	NA	NA	NA
	VirSorter	0.05	**0.002**	50.02	NA	NA	NA
	PlasFlow	NA	NA	NA	72.61	55.01	62.50
	cBar	NA	NA	NA	55.00	43.59	55.70
Group C 800-1200 bp	**PPR-Meta**	**95.24**	7.75	**98.54**	**78.09**	**10.95**	**91.84**
	VirFinder	81.91	15.63	91.09	NA	NA	NA
	VirSorter	0.17	**0.002**	50.09	NA	NA	NA
	PlasFlow	NA	NA	NA	75.89	50.55	68.01
	cBar	NA	NA	NA	55.54	41.87	56.84
Group D 5000-10k bp	**PPR-Meta**	**92.77**	**1.92**	**99.25**	**90.75**	**7.70**	**97.05**
	VirFinder	89.26	8.13	97.12	NA	NA	NA
	VirSorter	66.80	2.48	82.66	NA	NA	NA
	PlasFlow	NA	NA	NA	88.50	30.22	88.42
	cBar	NA	NA	NA	63.79	32.61	65.59

NA: not applicable.

Table 2. Performance comparison among BiPathCNN, the base path-only CNN and codon path-only CNN.

Group	Tool	Evaluation on phage			Evaluation on plasmid		
		TPR(%)	*FPR*(%)	*AUC*(%)	*TPR*(%)	*FPR*(%)	*AUC*(%)
Group A 100-400 bp	**BiPathCNN**	84.96	18.01	91.82	59.91	14.14	**83.05**
	Base path-only	81.86	24.58	87.50	56.96	17.60	78.50
	Codon path-only	86.84	20.47	**91.85**	62.15	16.65	82.26
Group B 400-800 bp	**BiPathCNN**	90.75	8.37	**97.21**	74.56	13.37	**89.64**
	Base path-only	88.76	17.46	93.87	72.37	18.86	85.57
	Codon path-only	84.95	5.93	96.57	82.98	23.10	88.32
Group C 800-1200 bp	**BiPathCNN**	95.24	7.75	**98.54**	78.09	10.95	**91.84**
	Base path-only	92.09	17.71	95.47	73.31	15.12	88.02
	Codon path-only	94.60	12.44	97.55	73.17	12.41	89.22

Table 3. Identification performance of each tool with 1% base substitutions.

Group	Tool	Evaluation on phage			Evaluation on plasmid		
		TPR(%)	*FPR*(%)	*AUC*(%)	*TPR*(%)	*FPR*(%)	*AUC*(%)
Group A 100-400 bp	**PPR-Meta**	**84.42**	17.99	**91.57**	61.19	**15.27**	**82.76**
	VirFinder	72.55	26.20	80.42	NA	NA	NA
	VirSorter	0.00	**0.00**	50.00	NA	NA	NA
	PlasFlow	NA	NA	NA	**71.72**	62.82	55.86
	cBar	NA	NA	NA	52.98	46.18	53.40
Group B 400-800 bp	**PPR-Meta**	**90.05**	8.48	**97.02**	**75.07**	**14.03**	**89.39**
	VirFinder	78.50	18.75	87.95	NA	NA	NA
	VirSorter	0.02	**0.00**	50.01	NA	NA	NA
	PlasFlow	NA	NA	NA	72.31	55.61	61.87
	cBar	NA	NA	NA	54.83	44.63	55.10
Group C 800-1200 bp	**PPR-Meta**	**94.54**	7.72	**98.33**	**79.03**	**11.99**	**91.59**
	VirFinder	81.29	15.92	90.68	NA	NA	NA
	VirSorter	0.21	**0.00**	50.11	NA	NA	NA
	PlasFlow	NA	NA	NA	75.24	50.91	67.15
	cBar	NA	NA	NA	56.57	42.85	56.86
Group D 5000-10k bp	**PPR-Meta**	**91.74**	1.86	**99.06**	**90.93**	**8.59**	**96.91**
	VirFinder	88.90	8.19	97.01	NA	NA	NA
	VirSorter	60.30	**1.13**	79.80	NA	NA	NA
	PlasFlow	NA	NA	NA	88.57	31.42	87.86
	cBar	NA	NA	NA	64.31	34.63	64.84

NA: not applicable.

Table 4. Identification performance of each tool with 1% base insertions or deletions.

Group	Tool	Evaluation on phage			Evaluation on plasmid		
		TPR(%)	*FPR*(%)	*AUC*(%)	*TPR*(%)	*FPR*(%)	*AUC*(%)
Group A 100-400 bp	**PPR-Meta**	**80.26**	18.64	**89.28**	65.29	**19.93**	**81.62**
	VirFinder	72.62	25.96	80.57	NA	NA	NA
	VirSorter	0.00	**0.00**	50.00	NA	NA	NA
	PlasFlow	NA	NA	NA	**71.12**	62.83	55.81
	cBar	NA	NA	NA	53.63	46.43	53.60
Group B 400-800 bp	**PPR-Meta**	**85.50**	9.69	**95.26**	**77.44**	**17.57**	**88.48**
	VirFinder	79.00	18.76	88.28	NA	NA	NA
	VirSorter	0.24	**0.00**	50.12	NA	NA	NA
	PlasFlow	NA	NA	NA	72.74	55.44	62.31
	cBar	NA	NA	NA	55.38	45.22	55.08
Group C 800-1200 bp	**PPR-Meta**	**92.99**	9.12	**97.54**	**79.74**	**14.29**	**90.80**
	VirFinder	81.98	16.00	90.93	NA	NA	NA
	VirSorter	2.38	**0.02**	51.18	NA	NA	NA
	PlasFlow	NA	NA	NA	75.23	51.25	66.75
	cBar	NA	NA	NA	56.74	43.45	56.64
Group D 5000-10k bp	**PPR-Meta**	**92.01**	1.96	**99.15**	**91.10**	**8.56**	**96.90**
	VirFinder	88.93	8.40	96.98	NA	NA	NA
	VirSorter	47.25	**0.25**	73.51	NA	NA	NA
	PlasFlow	NA	NA	NA	88.74	31.70	88.08
	cBar	NA	NA	NA	64.62	35.40	64.61

NA: not applicable.

Table 5. Recognition rate of prophages

Group	Tool	Recognition rate (%)
Group A 100-400 bp	**PPR-Meta**	**60.79**
	VirFinder	43.46
	VirSorter	0.00
Group B 400-800 bp	**PPR-Meta**	**60.59**
	VirFinder	40.77
	VirSorter	0.00
Group C 800-1200 bp	**PPR-Meta**	**68.09**
	VirFinder	41.94
	VirSorter	0.05
Group D 5000-10k bp	**PPR-Meta**	**72.37**
	VirFinder	48.62
	VirSorter	37.75

Figure 1 Click here to download Figure figure_1.pdf

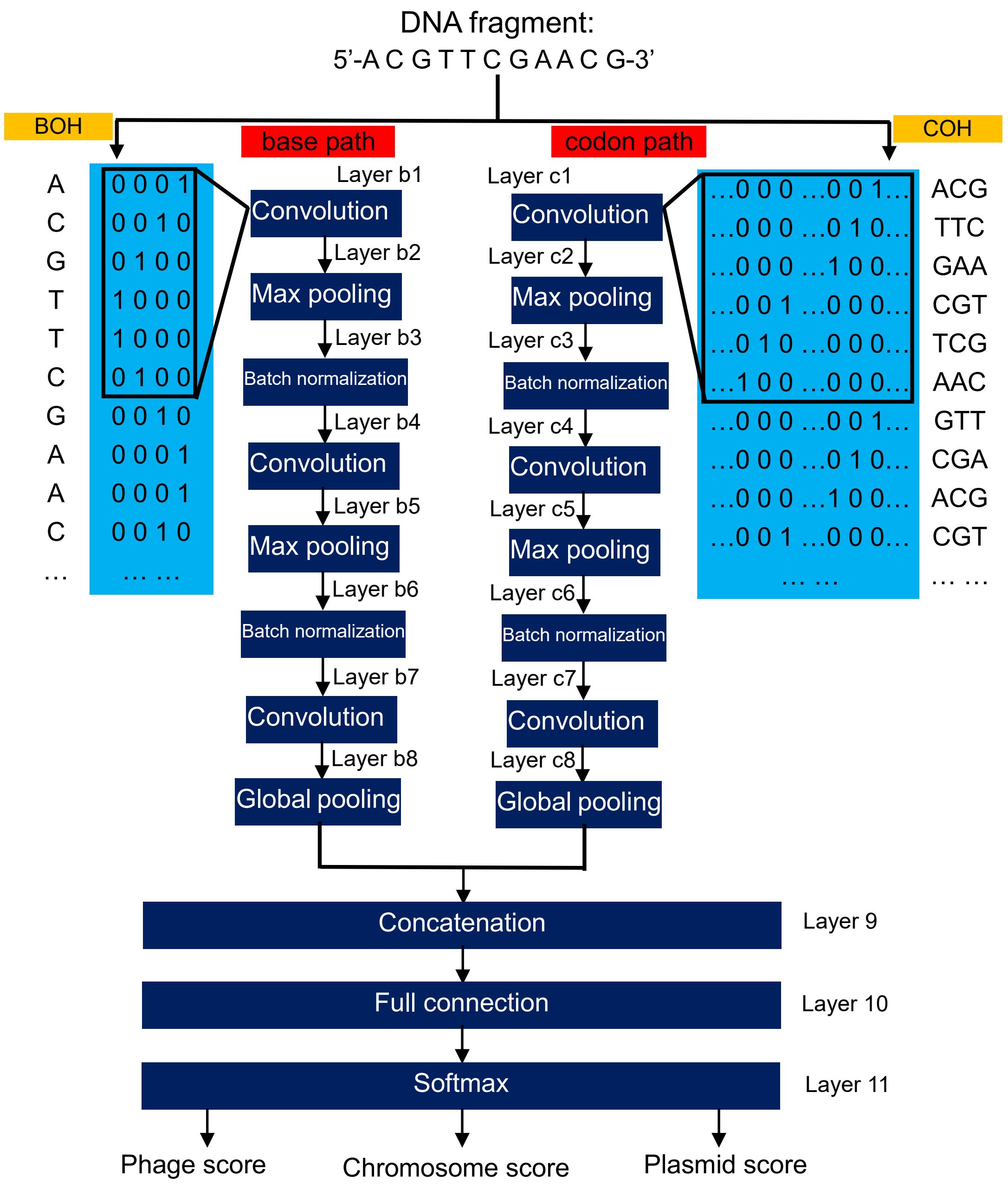

Click here to download Figure figure_2.pdf

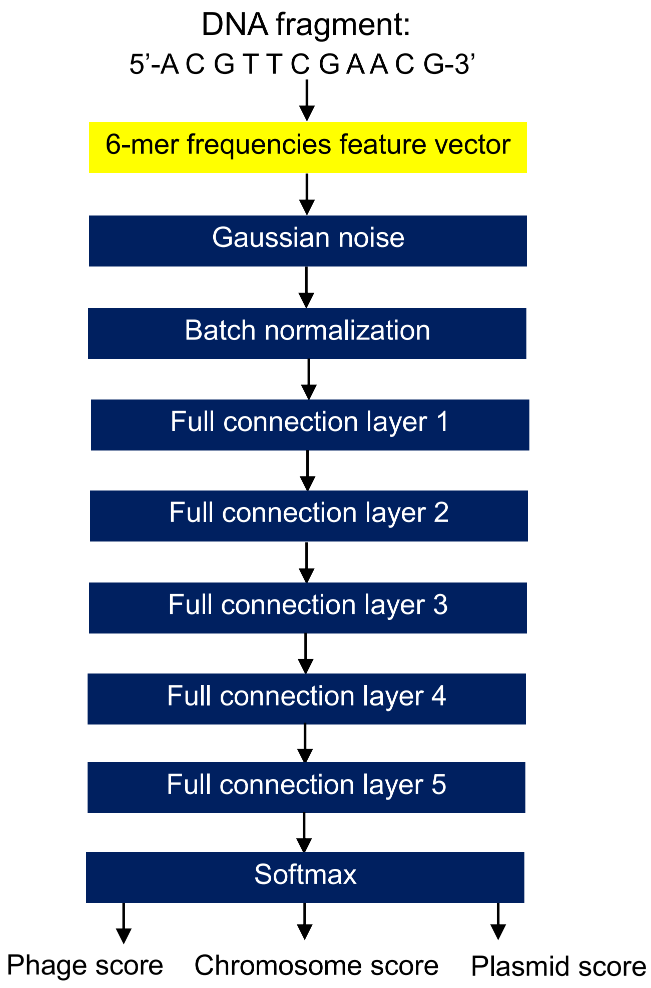

Figure 3

Click here to download Figure figure_3.pdf

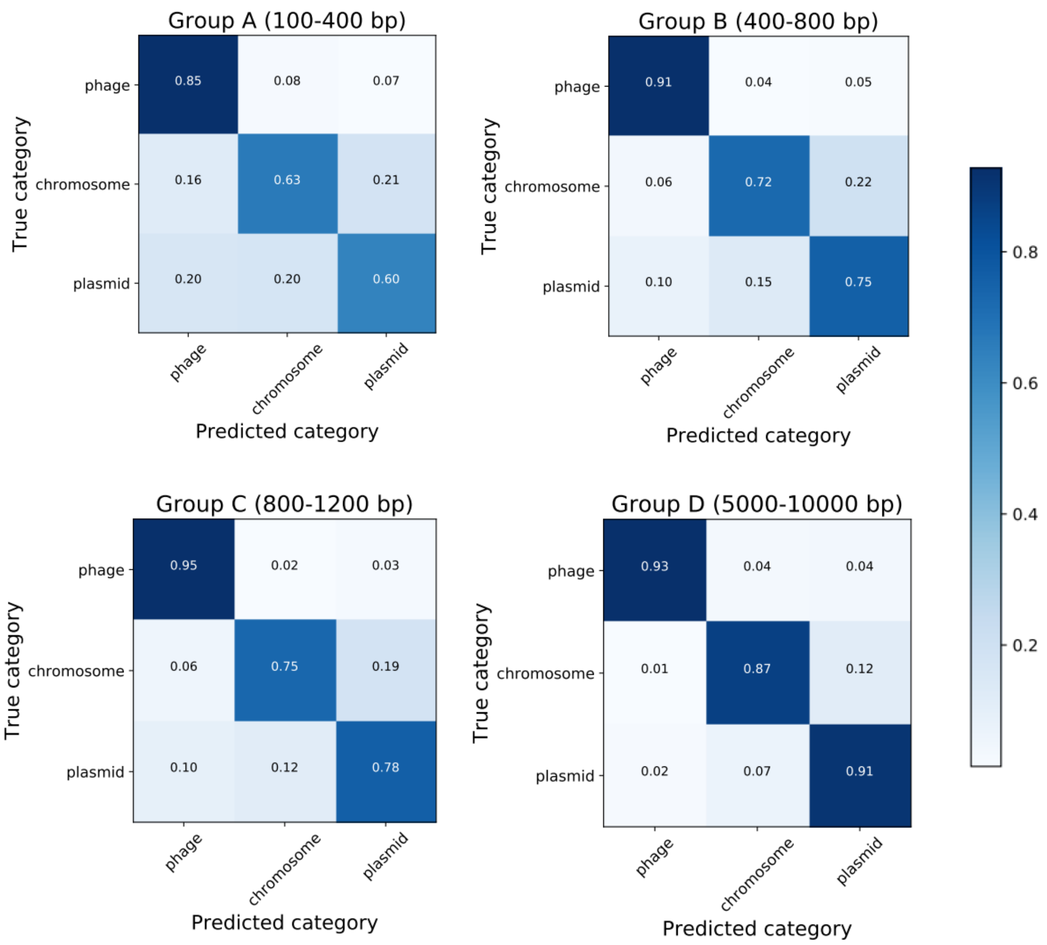

Figure 4 Click here to download Figure figure_4.png

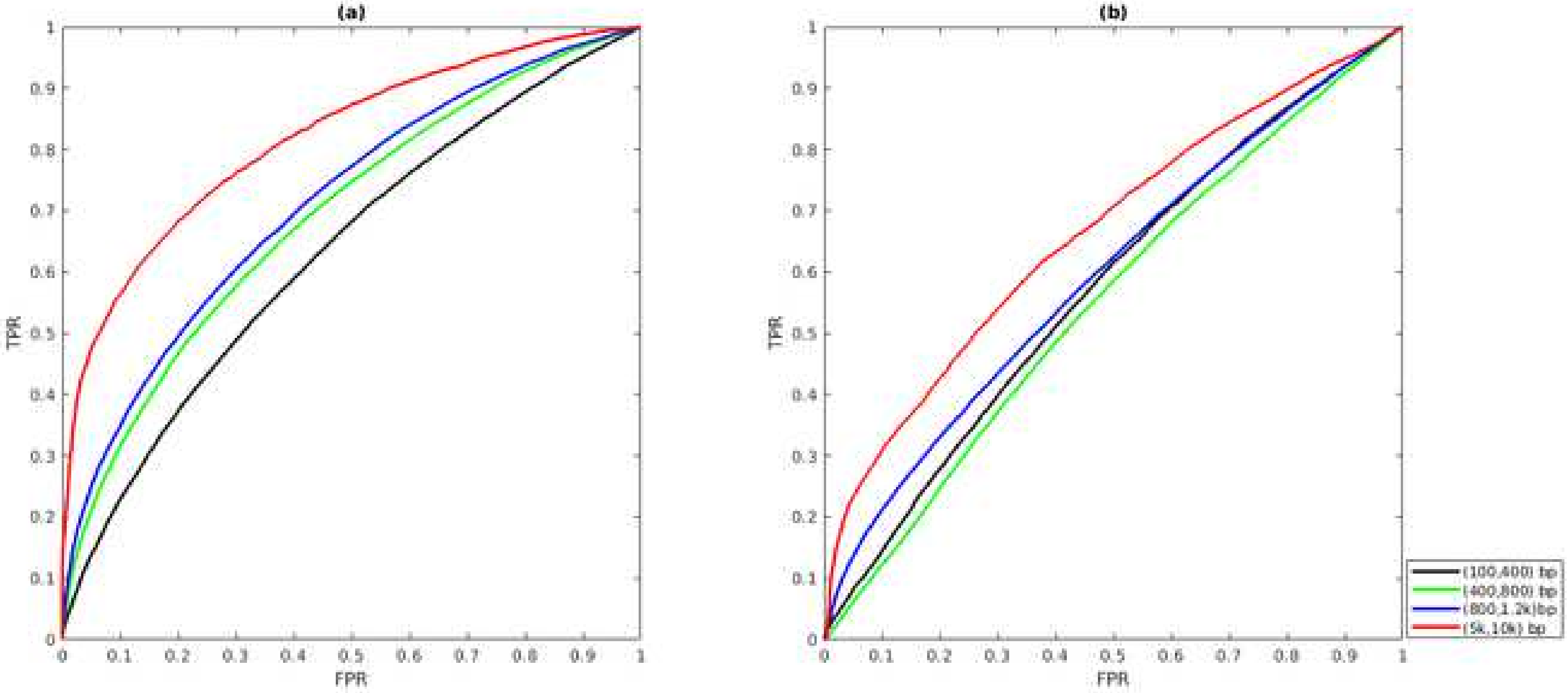

Additional File 1

Click here to access/download
Supplementary Material
Additional file 1.xlsx

Additional File 2

Click here to access/download
Supplementary Material
Additional file 2.xlsx

Click here to access/download
Supplementary Material
Additional file 3.docx
