# Supplementary material for: PPR-Meta: a tool for identifying phages and plasmids from metagenomic fragments using deep learning
Source: Gigascience. 2019 Jun 20;8(6):giz066. doi: 10.1093/gigascience/giz066 (PMC6586199; doi:10.1093/gigascience/giz066)
Supplement: giz066_GIGA-D-18-00464_Revision_1 [file giz066_giga-d-18-00464_revision_1.pdf]

# GigaScience


## PPR-Meta: a tool for identifying phages and plasmids from metagenomic fragments using deep learning

--Manuscript Draft--

| | |
|---|---|
| **Manuscript Number:** | GIGA-D-18-00464R1 |
| **Full Title:** | PPR-Meta: a tool for identifying phages and plasmids from metagenomic fragments using deep learning |
| **Article Type:** | Technical Note |
| **Funding Information:** | National Natural Science Foundation of China (31671366) — Dr Huaiqiu Zhu<br>Ministry of Science and Technology of the People's Republic of China (2017YFC1200205) — Dr Huaiqiu Zhu<br>Peking University (None) — Dr Huaiqiu Zhu |
| **Abstract:** | Background: Phages and plasmids are the major components of mobile genetic elements, and fragments from such elements generally co-exist with chromosome-derived fragments in sequenced metagenomic data. However, there is a lack of efficient methods that can simultaneously identify phages and plasmids in metagenomic data, and the existing tools identifying either phages or plasmids have not yet presented satisfactory performances.<br>Findings: We present PPR-Meta, a three-class classifier that allows simultaneous identification of both phage and plasmid fragments from metagenomic assemblies. PPR-Meta consists of several modules for predicting sequences of different lengths. Using deep learning, a novel network architecture, referred to as the Bi-path Convolutional Neural Network, is designed to improve the performance for short fragments. PPR-Meta demonstrates much better performance than currently available similar tools individually for phage or plasmid identification, while testing on both artificial contigs and real metagenomic data. PPR-Meta is freely available via http://cqb.pku.edu.cn/ZhuLab/PPR_Meta or https://github.com/zhenchengfang/PPR-Meta.<br>Conclusions: To the best of our knowledge, PPR-Meta is the first tool that can simultaneously identify phage and plasmid fragments efficiently and reliably. The software is optimized and can be easily run on a local PC by non-computer professionals. We developed PPR-Meta to promote the research on mobile genetic elements and horizontal gene transfer. |
| **Corresponding Author:** | Huaiqiu Zhu<br><br>CHINA |
| **Corresponding Author Secondary Information:** | |
| **Corresponding Author's Institution:** | |
| **Corresponding Author's Secondary Institution:** | |
| **First Author:** | Zhencheng Fang |
| **First Author Secondary Information:** | |
| **Order of Authors:** | Zhencheng Fang<br>Jie Tan<br>Shufang Wu<br>Mo Li<br>Congmin Xu |

Powered by Editorial Manager® and ProduXion Manager® from Aries Systems Corporation

| | |
|---|---|
| | Zhongjie Xie |
| | Huaiqiu Zhu |
| **Order of Authors Secondary Information:** | |
| **Response to Reviewers:** | Cover Letter<br><br>Dear Editor,<br><br>Thank you very much for your previous E-mail on December 26, 2018 regarding our manuscript ‘‘PPR-Meta: a tool for identifying phages and plasmids from metagenomic fragments using deep learning’’ (Manuscript ID: GIGA-D-18-00464). We are very grateful to your substantial and helpful advices on the manuscript. Herein we first thank three reviewers and are pleasant to know of their overall positive comments about our work as the findings being important to the community with closely related research interests. We thank so many substantial and valuable comments from three reviewers, including their careful reading and check to the manuscript, which clearly helped us improve the paper. In the revised manuscript, we marked all the changed words, sentences or paragraphs in red text.<br><br>Following your instruction and the reviewers’ comments, we have made a conscious effort to revise the manuscript (both the main document and Additional Files) with an essential improvement. Before we describe the revisions and responses to Reviewers’ comments, we would like to first report our revisions following Editor’s instructions. In particular, more appropriate data from prophage and genomic island databases have been used to evaluate PPR-Meta and the comparative tools (Please refer to Line 5-12, Page 20, and Line 5-15, Page 31 in the revised manuscript). We have made some improvements to the PPR-Meta tool to ameliorate the performance, and “whether the tool will automatically decide which model to use for prediction” is described more clearly in the manuscript (Please refer to Line 15, Page 12-Line 8, Page 13, Subsection “Structure of deep learning neural networks” in the revised manuscript.). We have also tested PPR-Meta on a series of metagenomic datasets of the human digestive tract, including the gut, throat and oral cavity, from the Human Microbiome Project (HMP). The findings are interesting and may be significant to the study of human health. In addition, we have registered PPR-Meta in the SciCrunch.org database and have added the RRID (SCR_016915) to Subsection “Availability of supporting source code and requirements” (Please refer to Line 9, Page 35 in the revised manuscript). All the outputs of the new analysis as well as the scripts for data preprocessing and neural network training have also been uploaded to the GigaScience server and our website. This time we uploaded the files separately instead of as one “zip” file in our previous submission, so that it could be updated more easily. Correspondingly, in the revised manuscript the link in Line 18, Page 34 has been changed to http://cqb.pku.edu.cn/ZhuLab/PPR_Meta/data/, and Section “Availability of supporting data” has been revised to: “The artificial contigs, related scripts and original results are available at http://cqb.pku.edu.cn/ZhuLab/PPR_Meta/data/. All the other data are available at corresponding references mentioned in the main text.” (Please refer to Line 12-14, Page 35 in the revised manuscript.) Meanwhile, we found that we missed some information in Section “Acknowledgements” and we have added the sentence “Part of the analysis was performed on the High Performance Computing Platform of the Center for Life Science of Peking University.” to the Section “Acknowledgements”. (Please refer to Line 14-16, Page 37 in the revised manuscript). More details are listed in the following point-by-point-responses to the reviewers’ comments.<br><br>We then report our revisions and responses to three reviewers’ all comments (italic text) one by one as follows:<br><br>To Reviewer #1:<br><br>General Comments:<br>The article by Fang et al describes a novel tool aimed at simultaneous identification of viral and plasmid sequences in metagenomic datasets. Presented tool can be of great value to many researchers analyzing microbiome data, especially for those interested in viral communities and/or mobile elements involved in the AMR spread and may as such also be widely cited. Authors used the novel neural network approach, which |

improves sequence classification for shorter sequences over other existing tools. Especially, usage of both noncoding and coding information is an important improvement over other existing tools, what authors clearly show in their manuscript. Herein we are glad to see Reviewer 1's positive comments on the present work as "… be of great value to many researchers analyzing microbiome data, especially for those interested in viral communities and/or mobile elements involved in the AMR spread and may as such also be widely cited." We especially thank Reviewer 1 for his/her careful reading of our manuscript. The suggestions and comments raised by Reviewer 1 were certainly very helpful for us to improve the manuscript. Below, we itemize the revisions in response to Reviewer 1's points.

1. Authors do not state what is the exact target of their software. Should it be used mainly with the metagenomic assemblies or can it be also easily applied to raw sequencing reads (because one of the models used in the work was trained on relatively short sequences (100-400 bp))? How will it work with isolate genome sequences?
We first thank Reviewer 1 for reminding us with a clear statement about the exact target of PPR-Meta. In the current work, PPR-Meta is primarily designed for metagenomic assemblies generated by next-generation sequencing technology. However, there are many sequences that are assembled poorly or even remain unassembled with short length in most metagenomic assemblies, especially the sequences from low abundance species or in condition of low coverage sequencing. This is the reason why we also trained and tested PPR-Meta using short sequences from 100 to 400 bp. So PPR-Meta can also be easily applied to raw sequencing reads. The only requirement for users is to dispose of all sequences in a "fasta" format file. To make the target clearer for readers, we revised the sentence in Section "Abstract" as follows: "We present PPR-Meta, a three-class classifier that allows simultaneous identification of both phage and plasmid fragments from metagenomic assemblies." (Please refer to Line 8-10, Page 2 in the revised manuscript.)
Of course, PPR-Meta can also work with isolated genome sequences. We collected complete genomes of the Acidianus tailed spindle virus (NC_029316.1), Ichthyobacterium seriolicida (AP014564.1) and Methylobacterium populi plasmid pMPPM01 (AP014810.1) from the test set (shown in the first line in the corresponding sheet in Additional File 1), and we found that PPR-Meta could correctly identify them as a phage, chromosome and plasmid, respectively. However, identifying complete genomes is not the main purpose of PPR-Meta because this is not a difficult issue and has been well addressed by other tools.
In addition, we have added a new section to provide an example application to show how PPR-Meta can be used to analyse metagenomic data. We employed PPR-Meta to identify phage and plasmid sequences on a series of metagenomic datasets of the human digestive tract, including the gut, throat and oral cavity, from the Human Microbiome Project (HMP). The finding is interesting and may be significant to the study of human health. We found that in the position closer to the outer end of the digestive tract, the percentages of phages and plasmids tended to be higher. For example, in the gut, the inner end of the digestive tract, the percentages of phages and plasmids were lower; in the oral cavity, the outer end of the digestive tract, the percentages of phages and plasmids were higher. Please refer to the new section "Phages and plasmids in the human digestive tract" for more details (Please refer to Line 18, Page 24 - Line 21, Page 25 in the revised manuscript.).

2. Although the training procedure and the algorithm itself are quite well described I missed some details regarding data preprocessing and preparation of models. What parameters were used in MetaSim to generate sequences used for training? Authors should note that MetaSim does not produce artificial contigs but synthetic sequencing reads, with technology-specific errors introduced. Did authors use 'exact' preset to return fragments perfectly matching reference sequences?
It would be also great if all the codes and scripts (e.g. those for preprocessing of sequences, and neural network training) are available online or as Supplemental materials.
Herein we realized that we did not provide a clear description of the data pre-processing and the preparation of the models. Actually, we used MetaSim to generate synthetic sequencing reads without technology-specific errors, that means we just used MetaSim to extract DNA sequences with different lengths that perfectly match reference sequences or that be modified with base substitutions or indels that are

evenly distributed over the sequences. To make this process clearer, we have added the description to Section “Methods” as: “To generate artificial contigs with no error for both training and test set, we used the “exact” preset to return fragments exactly matching reference sequences. In each group, the “DNA Clone Size Distribution Type” was set to “Uniform”. To generate artificial contigs modified with sequencing errors, we used the “Sanger” preset, which allowed users to modify sequences according to their settings. Note that as we were not going to generate sequences with technology-specific errors, the following settings do not reflect the real situation of the Sanger technology. For the generation of sequences with 1% base substitutions, the “Read Length Distribution Type” was set to “Uniform”, and the “Mate Pair Probability” was set to 0; both the “Error Rate at Read Star” and the “Error Rate at End of Read” were set to 0.01; and both the “Insertion Error Rate” and “Deletion Error Rate” were set to 0. For the generation of sequences with 1% base insertions or deletions, most settings were the same as mentioned above, except that both the “Insertion Error Rate” and “Deletion Error Rate” were set to 0.5.” (Please refer to Line 5-19, Page 33 in the revised manuscript.)
Also, we have added the following sentences in Subsection “Structure of deep learning neural networks” to describe how we prepared the BiPathCNN and selected hyperparameters: “The selection of the related hyperparameters of each path mentioned above was referred to LeNet-5 [37] and VGG [38], two classic Convolutional Neural Networks in the field of artificial intelligence. Specifically, the distribution of layers was referred to LeNet-5, which contained three convolution layers, and there was a pooling layer between every two convolution layers. Meanwhile, the distribution of the number of convolution kernels was referred to VGG, in which the number of convolution kernels in the different layers was increased by doubling. We also referred to VGG to use ReLU as the activation function.” (Please refer to Line 5-12, Page 12 in the revised manuscript). The citations of LeNet-5 and VGG have also been added to the list of the References.
In addition, we have uploaded the Keras scripts for the construction of the neural network as well as other scripts for data preprocessing to the GigaScience Database and our website (Link: http://cqb.pku.edu.cn/ZhuLab/PPR_Meta/data/). We wish that this is the best way to provide access to readers who want to reproduce or improve PPR-Meta.

3. Have authors tried to build model similar to BiPathCNN for longer sequences? As authors claim that codon path is beneficial for distinguishing plasmids, phages and chromosomes (p. 16, lines 7-10), this additional information should increase kmer-based approach, especially that longer fragments are more likely to contain coding sequences.
I also wonder why authors chose the hexamer frequencies and not any odd-number kmer?
We thank Reviewer 1 for this suggestion of using BiPathCNN for longer sequences. Firstly, we would like to describe the improvements that we made in the revised version of the PPR-Meta tool, which may also be closely related to the other comments. In the original version of PPR-Meta, we built four neural network models for sequences of different lengths. Among these neural networks, we used BiPathCNN, which contains a base path and a codon path, for model A, B and C, and we used a Fully Connected Neural Network (FNN), which takes k-mer frequencies as inputs, for model D. In the revised version of the PPR-Meta tool, we removed model D and kept model A, B and C. In practical applications, PPR-Meta uses model A to predict sequences between 100 and 400 bp, model B to predict sequences between 400 and 800 bp, and model C to predict sequences between 800 and 1200 bp. For sequences longer than 1200 bp, a scan window will move across the sequence without overlapping, and the weighted average of all windows’ predictions is calculated. The length of the window is set to 1200 bp (or less if the window is beyond the sequence boundary). For example, given a sequence of length 2500 bp, the scan window will first cover the bases from the 1st to 1200th positions, then the window will move to the bases from the 1201st to 2400th positions, and finally, the window will move to the bases from the 2401st to 2500th positions. Then, PPR-Meta uses model C, model C and model A to predict the subsequences under the first, second and third windows, respectively. To generate the final score for the whole sequence, PPR-Meta calculates the weighted average of these windows. The weights of these three windows are 1200/2500, 1200/2500 and 100/2500, respectively.
We made this change because we found that the revised version of PPR-Meta could

Powered by Editorial Manager® and ProduXion Manager® from Aries Systems Corporation

achieve a higher performance on long sequences. For example, for sequences with a length of 30k bp, the AUCs of both the phage identification and plasmid identification demonstrate higher performance. In particular, the TPR of phages increases from 93.76% to 99.84%, and almost all phages were identified. Although most sequences in the current metagenomic data are short fragments, a few reads from high-abundance species can be assembled into long contigs containing tens of thousands of bases, and we think that the revised PPR-Meta can be better adapted to these species. Additionally, considering that the third-generation sequencing technology is becoming more and more widely used, we hope that PPR-Meta can also promote studies using long sequencing technology, even though PPR-Meta is designed primarily for the next-generation sequencing technology. In the revised manuscript, we have added comparisons with related tools using sequences longer than 10k bp and sequences from real third-generation sequencing technology, which we will mention in the responses to the below comments.

We then address the questions in this comment. We tried to train BiPathCNN for longer sequences, but we failed to do this because it was very time consuming and had high hardware requirements. Thus, using a scan window to move across a long sequence may be a good alternative. In terms of the reason we used the 6-mer in the original version of PPR-Meta, it seems that the choice of k is not so significant. We tried different k values around 6, such as 5 and 7, and found that the results were comparable. In our opinion, the design of the neural network structure may be more important than the choice of the k value.

Because of the improvements we made in the revised PPR-Meta tool, as we mentioned at the beginning of this response, some of the results in the manuscript have also been updated. Herein, we would like to describe the updated content in our manuscript that is the result of these changes. None of the updated results mentioned below affect any conclusions that we have made in this manuscript. The revised version of the PPR-Meta tool has slight differences only on long sequences, while most of the test data we used in the manuscript are shorter than 5k bp, which is dominant in the current metagenomic sequences, and the revised PPR-Meta generates the same results for sequences shorter than 5k bp. Thus, the magnitude of all of the changes is small, except that the program has a longer running time for sequences longer than 5k bp, as shown in item (15) below.

The changes in the manuscript include the following:

(1). The original Figure 2, which describes the structure of the FNN, was removed. The second paragraph form the last in Subsection “Structure of deep learning neural networks”, which describes the FNN, was also removed.

(2). In Subsection “Mathematical model of DNA sequences”, the sentence “Here, we use a more detailed approach to represent the short sequences in Group A, Group B and Group C.” has been revised to “Here, we use a more detailed approach to represent the DNA fragments.”(Please refer to Line 7-8, Page 9 in the revised manuscript.) Also, the last paragraph of this section, which described using k-mer to represent DNA fragments in Group D, was removed.

(3). In Subsection “Structure of deep learning neural networks”, the sentences “…we trained corresponding neural networks for each group. For Group A, B and C, we designed BiPathCNN to improve the performance (Figure 1).” has been revised to: “…we trained three neural networks for Group A, B and C. To improve the performance, we designed BiPathCNN (Figure 1), a novel neural network structure, to make reliable predictions.”(Please refer to Line 15-17, Page 10 in the revised manuscript.)

(4). In Figure 2 in the revised manuscript (as Figure 3 in the original manuscript), the confusion matrix of Group D was updated. Also, in Subsection “Overall performance”, the phrase “shown in Figure 3” has been revised to “shown in Figure 2”. (Please refer to Line 15, Page 13 in the revised manuscript.)

(5). In Figure 4, the ROCs of Group D, which described the potential of using life_score and trans_score to classify the phage lifestyle and plasmid transmissibility, were updated. Also, the legend of Figure 4 has been revised to: “(a) Classify virulent phages and temperate phages using life_score. In order of sequence length, the AUC is 0.63, 0.69, 0.71 and 0.76. (b) Classify transmissible plasmid and non-transmissible plasmid using trans_score. In order of sequence length, the AUC is 0.58, 0.55, 0.60 and 0.62.

(6). In Section “Methods”, Line 21-22, Page 33, the sentence “Considering the memory size, running time and accuracy, a total of 3,060,000 artificial contigs were generated to train PPR-Meta.” has been revised to “Considering the memory size, running time and accuracy, a total of 2,700,000 artificial contigs were generated to train PPR-Meta.”

Powered by Editorial Manager® and ProduXion Manager® from Aries Systems Corporation

| | |
|---|---|
| | Also, the phrase “and 120,000 from Group D” has been removed from the sentence “The number of training contigs of each phage, chromosome and plasmid is 300,000 from Group A to C and 120,000 from Group D.” (Please refer to Line 22, Page 33-Line 2, Page 34 in the revised manuscript.)<br>(7). In Table 1, Table 3 and Table 4, the performance of PPR-Meta on Group D (the fifth row from the last of each table) was updated.<br>(8). In Table 5, the prophage recognition rate of PPR-Meta on Group D (the third row from the last) was updated.<br>(9). In Subsection “Performance comparison”, the sentence “The TPR of PPR-Meta was approximately 3%~13% higher than that of VirFinder and the FPR was approximately 6%~9% lower” has been revised to “The TPR of PPR-Meta was approximately 10% higher than that of VirFinder, and the FPR was approximately 5~10% lower.” (Refer to Line 13-15, Page 15 in the revised manuscript.)<br>(10). In Subsection “Performance comparison”, the sentences “For PPR-Meta, our FPR was much lower than that of cBar and PlasFlow. Although PPR-Meta achieved a slightly lower TPR than PlasFlow in Group A, our TPR remained highest in all other cases” have been revised to “For PPR-Meta, the TPR was comparable with that of PlasFlow, while the FPR was approximately 25~40% lower.” (Please refer to Line 1-2, Page 16 in the revised manuscript.)<br>(11). In Subsection “Evaluation in real metagenomic data”, the sentence “VirFinder and PPR-Meta were much better than VirSorter and identified 68.86% and 76.88% of the contigs, respectively, showing that PPR-Meta had the highest coverage of this data set” has been revised to “VirFinder and PPR-Meta were much better than VirSorter and identified 68.86% and 76.90% of the contigs, respectively, showing that PPR-Meta had the highest coverage of this data set.” (Refer to Line 21, Page 21 in the revised manuscript.)<br>(12). In Subsection “Evaluation in real metagenomic data”, the sentences “For PPR-Meta, total of 82.00% of the sequences were identified as MGEs, in which 49.16% were phages and 32.84% were plasmids. More than half of the sequences (64.72%) predicted as phages by PPR-Meta were also predicted as phages by VirFinder, and most of the sequences (74.72%) predicted as plasmids by PPR-Meta were also predicted by PlasFlow” have been revised to “For PPR-Meta, total of 81.96% of the sequences were identified as MGEs, in which 49.18% were phages and 32.78% were plasmids. More than half of the sequences (64.73%) predicted as phages by PPR-Meta were also predicted as phages by VirFinder, and most of the sequences (74.74%) predicted as plasmids by PPR-Meta were also predicted by PlasFlow.” (Please refer to Line 16-20, Page 22 in the revised manuscript.)<br>(13). In Subsection “Evaluation in real metagenomic data”, the sentence “In terms of phage identification, PPR-Meta, VirFinder and VirSorter predicted an average of 4.18%, 11.03% and 0% of the 16S-like contigs as phages, respectively, indicating that PPR-Meta likely generated fewer false positive predictions than VirFinder” has been revised to “In terms of phage identification, PPR-Meta, VirFinder and VirSorter predicted an average of 3.43%, 11.32% and 0% of the 16S-like contigs as phages, respectively, indicating that PPR-Meta likely generated fewer false positive predictions than VirFinder.” (Please refer to Line 17, Page 23 in the revised manuscript.)<br>(14). In Subsection “Evaluation in real metagenomic data”, the sentence “In terms of plasmid identification, PPR-Meta, PlasFlow and cBar predicted an average of 25.46%, 53.74% and 63.83% of the 16S-like contigs as plasmids, respectively, indicating that the PPR-Meta may generate the lowest number of false positive predictions” has been revised to “In terms of plasmid identification, PPR-Meta, PlasFlow and cBar predicted an average of 26.69%, 52.57% and 63.36% of the 16S-like contigs as plasmids, respectively, indicating that the PPR-Meta may generate the lowest number of false positive predictions.” (Refer to Line 22, Page 23 in the revised manuscript.)<br>(15). In Subsection “Usage of PPR-Meta”, the sentence “We tested the running time of PPR-Meta using 90,000 sequences from 100 to 10k bp and found that this tool can handle all sequences in approximately 15 minutes on a machine with the following configuration: CPU: Intel Core i7 6700; GPU: NVIDIA GTX1060; and Memory: 64G, DDR4” has been revised to: “… and found that this tool can handle all sequences in approximately 45 minutes on a machine with the following configuration …”(Please refer to Line 10, Page 27 in the revised manuscript.)<br>In items (13) and (14), the results of the comparative tools were also slightly different because we found that one setting in the data pre-processing script may not be the best option. Thus, we re-ran the pre-processing procedure to make the results more precise. The dataset pre-processing procedure is provided in Section “Methods” |

Powered by Editorial Manager® and ProduXion Manager® from Aries Systems Corporation

| | |
|---|---|
| | (Please refer to Line 3-16, Page 34 in the revised manuscript), and the data pre-processing script (file "20_gut.sh") is stored on our website and in the GigaScience Database.<br><br>4. I find really interesting part in which authors used likelihood scores generated by PPR-Meta to predict phage lifestyle or plasmid transmissability. Results shown are really encouraging and may make PPR-Meta an important tool in MGE studies. In the discussion of this phenomenon authors should cite the paper of Suzuki et al. (https://www.ncbi.nlm.nih.gov/pmc/articles/PMC2976448/), which discusses usage of genome signatures to predict the evolutionary host range of plasmids. Additionally, similarity between host and plasmid nucleotide composition is a known phenomenon, and so called genome amelioration is a term properly describing convergence of plasmid and host sequence patterns. Therefore, the statement "Since temperate phages and non-transmissible plasmids experience longer residence times within the host cell, they may adjust the sequence pattern toward the host" seems correct.<br>We appreciate Reviewer 1 for providing a reference that supports our hypothesis. Firstly, to make the description more rigorous, we have revised the sentence "This phenomenon may be due to the adaptation of foreign DNA to the host" to "This phenomenon may be due to the genome amelioration of foreign DNA to the host." (Please refer to Line 7, Page 30 in Section "Discussion and conclusions".) In addition, we have added the following sentence to the revised manuscript as: "For example, research has shown that the comparison of the trinucleotide composition between a plasmid and bacterial chromosome can be used to predict the host range of plasmids [53]." (Please refer to Line 8-10, Page 30 in Section "Discussion and conclusions".) The new citation has also been added to the list of the References.<br><br>5. For me it is really worth emphasizing that authors tested their software in the context of 3rd generation sequencing technologies like PacBio and Nanopore. As there is more and more reports showing applicability of single molecule sequencing technologies for describing microbial communities it would be really great to have tool which which is tested and works well on such datasets. Authors used 1% error rates for their simulated datasets, however, in my opinion they should test also higher error rates, as for aforementioned technologies they can reach up to 10%, error rate, especially for indels. Both technologies are sensitive to homopolymer sequences therefore one would expect more errors in such regions. Additionally I would also like to see if PPR-Meta is able to predict phage/plasmid sequences on real 3rd generation sequencing data. They can use for example recently published virome (https://www.biorxiv.org/content/early/2018/ 11/12/345041.full.pdf+html) or mock microbial community (https://www.biorxiv.org/content/early/ 2018/12/04/487033.full.pdf+html) nanopore data. The former one can also help with "Since we lack samples in which only chromosomes are enriched and all the extrachromosomal elements are filtered, estimating whether related tools will misjudge chromosomes as MGEs directly is difficult using real data" (p. 21, l. 9-11)<br>We would like to thank Reviewer 1 for the concern about testing PPR-Meta using artificial contigs with a higher error rate and real third generation sequencing data. In the revised manuscript, we tested PPR-Meta and related tools using artificial contigs with a high error rate. We used MetaSim to generate artificial contigs modified with 10% base substitutions and 10% indels in Group D, whose lengths were close to the raw reads generated from third generation sequencing data. The two types of errors were tested separately. The results showed that the AUCs of PPR-Meta remained the highest (>90%), although the performance was somewhat fluctuating, especially in the presence of 10% indels. Please refer to Additional File 3, Figure S3, for more details. On the other hand, we consider that a tool that can tolerate a 1% error rate is competent for handling the third generation sequencing data. Although the error rate of third generation sequencing technology can reach up to 10%, many basecalling tools have been developed to help improve the accuracy to over 99%. Thus, we believe that PPR-Meta can generate reliable predictions on the third sequencing data that go through Quality Control (QC). We have added the following sentences to the revised manuscript as: "Considering that the error rate of the raw data generated from the third-generation sequencing technology may be much higher, we also tested PPR-Meta and the related tools using artificial contigs modified with 10% base substitutions and 10% insertions or deletions in Group D, whose lengths were close to the raw reads generated from third-generation sequencing technology. The results are shown in Additional File 3 and Figure S3. The results showed that the AUCs of PPR-Meta |

remained the highest (>90%), although the performance was somewhat fluctuating, especially in the presence of 10% insertions or deletions. Recently, many basecalling tools for the third-generation sequencing technology have been developed to help improve the accuracy over 99% [41], therefore the extremely high error rate on the raw data will not affect the usage of PPR-Meta." (Please refer to Line 4-15, Page 19 in Subsection "Performance in the presence of sequencing errors" in the revised manuscript.) The new citation has also been added to the list of the References.
We also tested whether PPR-Meta and the related tools can predict phage sequences on real third generation sequencing data using virome data from the reference provided by Reviewer 1. The results showed that PPR-Meta could identify more viruses in this dataset. We have added the following sentences to the revised manuscript as: "Considering that the third-generation sequencing technology is more and more widely used to analyse metagenomes, we also used real virome data generated by MinION [46] to test whether PPR-Meta and the related tools can identify phages from third-generation sequencing technology. The virome was downloaded as assembled sequences (accession: GCA_900491955.1), containing 1500 sequences. The results showed that PPR-Meta, VirFinder and VirSorter could identify 79.20%, 76.27% and 30.40% of viral sequences respectively, indicating that PPR-Meta has the highest performance. Therefore PPR-Meta can also handle data from the third-generation sequencing technology, although it is designed primarily for the next-generation sequencing technology." (Please refer to Line 6-16, Page 24 in Subsection "Evaluation in real metagenomic data".) The new citation has also been added to the list of the References.

6. It should be also noted that in case of plasmid prediction PPR-Meta presents different approach than previously published cBar and PlasFlow only for sequences shorter than 10kb, as neural network trained on Group D essentially use the 6-mer frequencies for prediction. Additionally in the PlasFlow manual it is said that such approach does not work well on short sequences (recommended length is > 1kb), therefore comparisons using test datasets from GroupA and GroupB should be done with having this in mind. In my opinion authors should also comment on different lengths of sequences used for training in cBar, PlasFlow and PPR-Meta: cBar was trained with whole chromosome/plasmid sequences whereas PlasFlow on 10kb fragments and PPR-Meta using 4 datasets with differing lengths, what may significantly influence their performance.
We thank Reviewer 1 for reminding us to note that PPR-Meta uses a similar approach to cBar and PlasFlow for long sequences. As we mentioned in the response to General Comment #3, in the revised version of the PPR-Meta tool, we removed the k-mer-based Fully Connected Neural Network (FNN) and instead used BiPathCNN to predict sequences of all lengths, which helped to improve its performance for very long sequences. Indeed, we found that the k-mer-based approach did not work well for short sequences. For example, we used the FNN in the original PPR-Meta tool to predict the sequences in Group A (100~400 bp), and we found that the performance was much poorer: the AUC of phage identification decreased from 91.84% to 72.70%, and the AUC of plasmid identification decreased from 83.05% to 64.79%. One of the innovations of PPR-Meta is that we do not use k-mer frequencies to represent DNA sequences, as we have emphasized in our manuscript. For example, we mentioned that "Although k-mer frequencies have been widely used in many studies, such frequencies may present serious fluctuations in short sequences" and "The performance improvement on short sequences demonstrates that our sequences representation method is more detailed than the k-mer frequencies". To further emphasize the difference between PPR-Meta and the comparative tools, we have added the following sentence to the revised manuscript as: "Overall, PPR-Meta presented a much better performance than other homology-search-based tools such as VirSorter and k-mer-based tools such as VirFinder, PlasFlow and cBar." (Please refer to Line 11-13, Page 16 in Subsection "Performance comparison".)
In our opinion, the k-mer-based methods are more sensitive to sequence length than our BiPathCNN. The distribution of the k-mer frequencies may be different between long sequences and short sequences, and the variance of the k-mer frequencies for short sequences may be much higher. Thus, the k-mer-based classifier constructed using short sequence data may not by applicable for long sequences, and vice versa. We also tested the accuracy of each BiPathCNN on test sets from other groups, as per Reviewer 1's suggestion in Specific Comment #7. We found that although the overall accuracy was slightly reduced when testing each group using the non-corresponding

BiPathCNNs from the other groups, the decrease was not obvious (Please refer to Additional File 3, and Figure S2), indicating that our approach was not quite sensitive to the sequence length. We have added the following sentences to Section “Discussion and conclusions” in the revised manuscript as: “On the other hand, k-mer-based methods may also be more sensitive to the sequence length than the BiPathCNN method in the current work. The distribution of k-mer frequencies may be different between long sequences and short sequences, and the variance of the k-mer frequencies for short sequences may be much higher. Thus, the k-mer-based classifier constructed using short sequence data may not by applicable for long sequences, and vice versa. Among the k-mer-based tools, cBar was trained with complete genomes and PlasFlow was trained on 10k bp fragments, which might make them hard to adapt to metagenomic data with a wide range of lengths. Differently, our BiPathCNN directly extracts sequence features from the raw data represented by the one-hot matrix and may be less sensitive to the sequence length. Tests of each BiPathCNN on test datasets from different groups (Additional File 3, Figure S2) also showed that although the overall accuracy was slightly reduced when testing each group using a non-corresponding BiPathCNN from the other groups, the decrease was not obvious, indicating that our approach is not quite sensitive to the sequence length.” (Please refer to Line 5-20, Page 28 in the revised manuscript.)

Specific Comments:
1. Page 5 lines 2-4: "extracted" should be "extract"
We thank Reviewer 1 for the careful check of our manuscript, and we have corrected this mistake. The sentence has been revised to “Such approaches primarily used a scan window to move across the complete bacterial chromosome and extract regions that seem to be phages based on a similarity search against viral databases.” (Please refer to Line 4, Page 5, in Section “Introduction”.) Meanwhile, after proofreading our manuscript, we have also revised some phrases to improve the language and presentation. Herein we list the revision as follows:
Line 4, Page 15 and Line 14, Page 22, “In term of” have been revised to “In terms of”.
Line 20, Page 15, “appearing” has been revised to “appeared”.
Line 22, Page 31, “sequencing” has been revised to “sequenced”.
Line 16, Page 9, “For BOH” has been revised to “For BOH in PPR-Meta”.
Line 11-12, Page 20, “The decline in performance may be due to…” has been revised to “The lower recognition rate of prophages compared with that of the phages in the NCBI database may be due to…”
Line 20-21, Page 28, we have replaced “Moreover…” with “Another shortcoming of k-mer-based tools may be that…”

2. Page 5 line 21: Authors may also cite following tool: https://www.ncbi.nlm.nih.gov/pubmed/ 30383524.
We thank Reviewer 1 for reminding us that we missed a related tool in our manuscript. Now, the corresponding sentence has been revised to “In terms of plasmids, most of the current tools for plasmid identification were designed for WGS or even specific species, such as PlasmidFinder [20], PLACNET [21], PlasmidSeeker [22] and mlplasmids [23].” (Please refer to Line 21, Page 5, in Section “Introduction”.)

3. Page 6 line 6 "this tool applies SMO" should be changed to: "this tool applies SOM" (Self Organizing Map)
We thank Reviewer 1 for this careful check. According to the reference of cBar, SMO refers to “sequential minimal optimization”. To make it clearer to readers, we have revised the sentence to “This tool applies sequential minimal optimization (SMO) as a classifier based on k-mer frequencies.” (Please refer to Line 6, Page 6, in Section “Introduction”.) In addition, the term “SMO” has also been added to Section “List of abbreviations.” (Please refer to Line 3, Page 36.)

4. page 8 lines 13-19 "phage metagenomic data of bovine rumen [19], which were downloaded from MG-RAST [29] (Accessions: mgm4534202.3 and mgm4534203.3) as raw reads and assembled by SPAdes" and "20 samples of healthy human gut [32], downloaded from the NCBI Short Read Archive [33] and assembled by SPAdes." I miss details on SPAdes assembly. What settings were used and what was the quality of assembly (N50, number of contigs and so on).
We apologize that we missed some details in our manuscript. In the revised manuscript, we have added these details to Section “Methods” as follows: “We also

used real metagenomic data to evaluate PPR-Meta and the related tools. We used SPAdes to assemble the raw reads, as we mentioned in the main text. The phage metagenomic data of the bovine rumen were downloaded from MG-RAST, and we used the command “spades.py --meta -1 file1.fastq -2 file2.fastq -o out_folder” to assemble the pair-end raw reads. In the assembly, the contig number, N50, average length, maximum length and minimum length were 107529, 288, 312.06, 75508 and 56, respectively. To download the 20 samples of the healthy human gut, we used the command “prefetch SRRaccession” from the SRA Toolkit. All samples were downloaded as “.sra” files. We then used the command “fastq-dump --split-files accession.sra” from the SRA Toolkit to convert the sra file into two pair-end fastq files and used SPAdes with the same settings as mentioned above to assemble the raw reads. The information about the contig number, N50, average length, maximum length and minimum length is provided in Additional File 1.” (Please refer to Line 3-16, Page 34 in the revised manuscript.) Also, the sentence “Additional details on the dataset construction are provided in Methods section.” has been moved from the second paragraph from the last to the last paragraph in Section “Dataset construction”. (Please refer to Line 1, Page 9 in the revised manuscript.)
In addition, the scripts used to calculate the quality of the assemblies were provided in the GigaScience Database and on our website.

5. page 12 lines 5-11: Which approach is used for sequences 5-10 kb? FNN or biPath-CNN (groupC model)? If biPath-CNN - what is its performance on this dataset compared to FNN (group D)? It is not clear which approach the software will use for the real datasets of this length.
We are very sorry that we did not provide a clear statement of this issue in our manuscript. As we mentioned in the response to General Comment #3, we removed the FNN from the revised PPR-Meta tool. For sequences longer than 1200 bp, a scan window is used to make a prediction. To make this clearer to the readers, we have added the following sentences to the revised manuscript as: “In practical applications, PPR-Meta uses BiPathCNN A to predict sequences between 100 and 400 bp, BiPathCNN B to predict sequences between 400 and 800 bp, and BiPathCNN C to predict sequences between 800 and 1200 bp. For sequences longer than 1200 bp, such as sequences in Group D, a scan window will move across the sequence without overlapping, and the weighted average of all windows’ predictions is calculated. The length of the window is set to 1200 bp (or less if the window ends beyond the sequence boundary). For example, given a sequence of length 2500 bp, the scan window will first cover the bases from the 1st to 1200th positions, then the window will move to bases from the 1201st to 2400th positions, and finally, the window will move to bases from the 2401st to 2500th positions. Then, PPR-Meta uses BiPathCNN C, BiPathCNN C and BiPathCNN A to predict the subsequences under the first, second and third windows, respectively. To generate the final score for the whole sequence, PPR-Meta calculates the weighted average of these windows. The weights of these three windows are 1200/2500, 1200/2500 and 100/2500, respectively.” (Please refer to Line 15, Page 12-Line 8, Page 13, Subsection “Structure of deep learning neural networks” in the revised manuscript.)

6. I would also like to see the comparison to other software regarding fragments longer than 10kb (which are easily achievable with current metagenomic sequencing techniques).
We would like to thank Reviewer 1 for this concern about making comparisons for fragments longer than 10k bp. We have added comparisons on 15k and 30k bp fragments to the revised manuscript, and the performance of PPR-Meta is still the best. The results are shown in Additional File 3, Figure S1, and we have added the following sentences to the revised manuscript as: “In some cases, a few assembled sequences from high-abundance species may be much longer, so we also tested PPR-Meta and the related tools using 15k bp and 30k bp fragments (shown in Additional File 3, Figure S1). The results showed that the performance of PPR-Meta was still the best for these long sequences.” (Please refer to Line 2-6, Page 16, Subsection “Performance comparison” in the revised manuscript.)

7. Additionally, please test accuracy of each model on testing datasets from other models, e.g. model for group A on test datasets for groups B, C and D, model for group B on test datasets for groups A, C and D, etc. It is not likely that in real datasets sequences will be distributed such uniformly. It is also interesting how biPath-CNN

| | |
|---|---|
| | performs on long sequences, as coding information should significantly increase its performance (like it was shown for shorter fragments). And, maybe, any of the single models is good enough to be used on fragments of all lengths; for me this possibility can not be excluded by looking at presented data.<br>We understand the consideration of Reviewer 1 that using a single model may be good enough for fragments of all lengths. In fact, using different neural networks for sequences of different groups can not only help to improve the accuracy but also speed up the program. As shown in Additional File 3, Figure S2, we tested the accuracy as well as the running time of each neural network on the test datasets from all groups. The results showed that using a non-corresponding neural network from another group to predict sequences from a specific group would lead to a lower accuracy and longer running time (the reason will be described below). Indeed, the sequence lengths in a real dataset may not be distributed uniformly. In many cases, the distribution of the sequence lengths in real metagenomic data is more like a Poisson distribution, with most of the lengths around 0.5k-2k bp and a few of them much longer. Since most of the sequences are short, we considered that it is essential to construct different neural networks for short sequences. As we mentioned in the response to General Comment #3, we also tried to train BiPathCNN for long contigs but failed because it was very time consuming and had high hardware requirements. As an alternative, PPR-Meta uses a scan window for long sequences and predict the subsequence in each window using the corresponding BiPathCNN. The evaluation on long sequences, such as sequences of 15k bp and 30k bp, has shown the effectiveness of this approach.<br>Herein, we would like to explain why using a non-corresponding BiPathCNN would increase the running time. In fact, once the BiPathCNN is constructed, the input size of the neural network is fixed. For example, the BiPathCNN of Group C can handle sequences with a maximum length of 1200 bp. If the BiPathCNN of Group C is used to predict a sequence of 100 bp, the “base one-hot matrix” must be padded with a number of rows of [0,0,0,0], in which all bits are zero, to adapt the input size of the neural network and so does the “codon one-hot matrix”. In general, padding with zeros does not significantly affect the accuracy but will add unnecessary calculations for the neural network, which will also increase the running time. Similarly, the BiPathCNN of Group A can handle sequences with a maximum length of 400 bp. If the BiPathCNN of Group A is used to predict a sequence of 1200 bp, a scan window must be used to split the sequence into 3 subsequences of 400 bp. Each subsequence will be predicted separately, and then an average score for the whole sequence will be calculated. Since the total number of sequences is increased, the running time will also be longer, although the accuracy will not be significantly reduced, as we showed in Figure S2. Overall, we consider that the usage of three BiPathCNNs together with the scan window for long sequences in the revised version of PPR-Meta may be a good choice for handling sequences of different lengths.<br>In the revised manuscript, we have added the following sentence as: “In addition, we tested the accuracy as well as the running time of each BiPathCNN on test datasets from different groups and found that using a non-corresponding BiPathCNN to predict sequences from specific groups would lead to a lower accuracy and longer running time (shown in Additional File 3, Figure S2).” (Please refer to Line 6-10, Page 16, Subsection “Performance comparison” in the revised manuscript.)<br><br>8. Page 15 line 12: I would remove the word "obviously". Please be less advertising and more informative.<br>We are sorry for our inappropriate description. In the revised manuscript, this sentence has been removed because this sentence was part of the discussion of the performance of the FNN that we used in the original version of PPR-Meta, but we removed the FNN in the revised version.<br><br>9. page 16 lines 10-13: "Compared with other sequence representation methods that ignore the coding or non-coding region, such as method based on k-mer frequencies, PPR-Meta uses a more detailed method of describing a sequence and achieves a higher performance." Authors should explicitly note that it relates only to sequences shorter than 5(or 10, see my note above) kb.<br>We thank Reviewer 1 for bringing to our attention this inappropriate description. However, as we mentioned in the response to General Comment #3, the revised version of the PPR-Meta tool employs BiPathCNN for all sequences. Thus, this description seems to be appropriate in the revised manuscript. |

Powered by Editorial Manager® and ProduXion Manager® from Aries Systems Corporation

10. Page 22, lines 16-17: "PPR-Meta is designed with the option to adjust the default threshold of discriminant criteria" It should be described more precisely. Although in the Manual it is noted, that "In this way, sequences with the phage (or plasmid) score higher than the other two categories and the threshold will be regarded as phage (or plasmid)", it is not mentioned that sequences not exceeding threshold for phage or plasmid category will fall into the "chromosome" category, what may not be the best option, increasing False Negative Rate. I also lack more information on the accuracy of PPR-Meta run with different thresholds (Table S1). Can you include also AUC in the table? And compare with PlasFlow, using the same thresholds?
We thank Reviewer 1 for this comment. We quite agree that sequences not exceeding the threshold for the phage or plasmid category will fall into the chromosome category may increase the False Negative Rate. In the revised version of PPR-Meta, we referred to the threshold usage of PlasFlow. Specifically, given a threshold by a user, the sequence with the highest score lower than the threshold will be labelled as “uncertain”. In this way, the outputs of PPR-Meta contain six categories: phage, uncertain phage, chromosome, uncertain chromosome, plasmid and uncertain plasmid. In Additional File 3, Figure S5, we evaluated the uncertain prediction rate, accuracy, AUC, TPR and FPR under different thresholds. The accuracy was defined as the ratio of the number of correctly predicted fragments to the total number of fragments, rather than being calculated on either the phages or plasmids separately. Thus, the accuracy can reflect the overall performance of PPR-Meta. The accuracy, AUC, TPR and FPR were calculated only on the certain predictions. In general, with a higher threshold, the accuracy, AUC, and TPR as well as the uncertain prediction rate will be higher, while the FPR will be lower. We have added the following sentences to the revised manuscript as: “To meet users’ actual requirements, PPR-Meta is designed with the option to adjust the threshold to filter out the uncertain predictions so that the remaining predictions may be more reliable. Given a threshold, a sequence with a highest score lower than the threshold will be labelled as “uncertain”. In this way, the outputs of PPR-Meta contain six categories: phage, uncertain phage, chromosome, uncertain chromosome, plasmid and uncertain plasmid. We evaluated the uncertain prediction rate, accuracy, AUC, TPR and FPR under different thresholds, and the results are shown in Additional File 3, Figure S5. In general, with a higher threshold, the accuracy, AUC, and TPR as well as the uncertain prediction rate will be higher, while the FPR will be lower.” (Please refer to Line 11-21, Page 26, Subsection “Usage of PPR-Meta” in the revised manuscript.)
Herein, we compared the AUC of plasmid identification between PPR-Meta and PlasFlow, both using 0.7 as the threshold (the default threshold in PlasFlow). In Group A (100-400 bp), the AUC of PPR-Meta increased from 83.05% to 90.61%, while 53.36% of sequences were labelled as uncertain; the AUC of PlasFlow increased from 56.30% to 77.05%, while 37.70% of sequences were labelled as uncertain. In Group B (400-800 bp), the AUC of PPR-Meta increased from 89.64% to 93.99%, while 32.71% of sequences were labelled as uncertain; the AUC of PlasFlow increased from 62.50% to 81.85%, while 39.34% of sequences were labelled as uncertain. In Group C (800-1200 bp), the AUC of PPR-Meta increased from 91.84% to 94.68%, while 23.40% of sequences were labelled as uncertain; the AUC of PlasFlow increased from 68.01% to 84.79%, while 38.21% of sequences were labelled as uncertain. In Group D (5k-10k bp), the AUC of PPR-Meta increased from 96.02% to 97.63%, while 25.11% of sequences were labelled as uncertain; the AUC of PlasFlow increased from 88.42% to 93.86%, while 25.46% of sequences were labelled as uncertain.

11. This is only the suggestion, but all the data presenting performance of PPR-Meta in comparison to other tools can be also presented as graphs, what would allow for easy assessment of differences.
We thank Reviewer 1 for this suggestion. In the main text, we presented the results as tables to make the information more precise. However, we quite agree that too many tables will make the manuscript difficult to read. Thus, we presented all of the supplementary results mentioned above as graphs in Additional File 3 to make them easy to assess. Correspondingly, the description of Additional File 3 in the “Additional file” section has been revised to: “Additional file 3: Figure S1 to Figure S5”. (Please refer to Line 19, Page 35.)

Specific Comments Regarding Software Usaibility
1. Using output file extension other than .csv throws an error:

Error using writetable (line 124)
Unrecognized file extension '.tsv'. Use the 'FileType' parameter to specify the file type
Error in PPR-Meta(line 124)
MATLAB:table:write:UnrecognizedFileExtension
This should be better documented, and the user should be warned at the beginning of computation that using custom file extensions will cause that output file cannot be written.
We thank Reviewer 1 for testing our program and discovering the "bug" in PPR-Meta. We have addressed this "bug", and PPR-Meta will now automatically check the extension. If the user does not use ".csv" as an extension, the program will directly add the ".csv" extension to the output file and give a warning. We have added the following sentences to the manual to remind users of this: "Note: the current version of PPR-Meta uses "comma-separated values (CSV)" as the format of the output file. Please use ".csv" as the extension of the output file. PPR-Meta will automatically add the ".csv" extension to the file name if the output file does not take ".csv" as its extension." (Please refer to Section 5, Part I in the manual.)

To Reviewer #2:

General Comments:
Authors present a tool which is able to perform multiclass prediction of phage, plasmid and chromosome sequences in metagenomic data. Using this deep learning approach along with its architecture and the weighted average of all windows is worthy of publication by itself. Really interesting. Tool, source code, virtual machine (along with a video explaining how to use it) and supporting datasets are all publicly available. Also, the discussion section is really interesting. Noting that the differences between chromosome and phage scores may reflect phage lifestyle may be an relevant finding.
Herein we first appreciate Reviewer 2's positive comments on our present work. We would like to especially thank Reviewer 2 for these comments and suggestions, which were certainly helpful for us to improve our work. For Reviewer 2's following concerns on our work, we present our responses and the corresponding improvements or revisions as follows.

1. I am somehow concerned by the biological support of trying to predict these three classes. My concern in mainly about the overlap characteristics that plasmid and chromosomes usually present. And the same thing may be said about phages/prophages, chromosomes and plasmids. An example of this is explored by authors in results reported in page 20.
As a suggestion, authors should state clearly that the tool is aimed to perform a three-class prediction. Perhaps adding sentences to the abstract or even to the title.
We fully understand Reviewer 2's concern about the biological support of trying to predict these three classes. Indeed, genome amelioration is widely observed in foreign DNA, so it is not strange that plasmids (or phages) and chromosomes usually present overlap characteristics. In fact, the similarity of the nucleotide composition between plasmids (or phages) and chromosomes is often used to predict the host of a given plasmid or phage (see Galiez et al., WIsH: who is the host? Predicting prokaryotic hosts from metagenomic phage contigs. Bioinformatics, 2017; 33(19): 3113-3114; Suzuki et al., Predicting plasmid promiscuity based on genomic signature. J. Bacteriol., 2010; 192(22): 6045–6055.) However, the similarity of the nucleotide compositions between plasmids (or phages) and chromosomes is not contradictory with using the characteristics of the nucleotide composition to identify plasmids or phages because the plasmids or phages can still maintain some specific sequence patterns. For example, experiments conducted by Ren et al. showed that a virus and its host shared some similar k-mers, which could help predict the host of a given virus, while viruses would also share more similar k-mers with each other, which could help distinguish them from the hosts (see Ren et al., VirFinder: a novel k-mer based tool for identifying viral sequences from assembled metagenomic data. Microbiome, 2017; 5(1): 69.). Thus, we consider that using the nucleotide composition to identify phages and plasmids is biologically feasible.
To make it clearer to readers that PPR-Meta is intended to perform a three-class prediction, we have revised the sentence in Section "Abstract" as: "We present PPR-Meta, a three-class classifier that allows simultaneous identification of both phage and plasmid fragments from metagenomic assemblies." (Please refer to Line 8-10, Page 2 in the revised manuscript.) We have also revised the sentence in Section "Introduction"

Powered by Editorial Manager® and ProduXion Manager® from Aries Systems Corporation

as: “In this paper, we present the PPR-Meta (Phage and Plasmid Recognizer for Metagenomes), a three-class classifier for identifying metagenomic fragments as phages, plasmids or chromosomes based on the deep learning algorithm.” (Please refer to Line 19, Page 6 in the revised manuscript.) In addition, we have added a new subsection to provide an example application to show how PPR-Meta can be used to analyse metagenomic data. We employed PPR-Meta to identify phage and plasmid sequences on a series of metagenomic datasets of the human digestive tract, including the gut, throat and oral cavity, from the Human Microbiome Project (HMP). The finding is interesting and may be significant to the study of human health. We found that in positions closer to the outer end of the digestive tract, the percentages of phages and plasmids tended to be higher. For example, in the gut, the inner end of the digestive tract, the percentages of phages and plasmids were lower, while in the oral cavity, the outer end of digestive tract, the percentages of phages and plasmids were higher. Please refer to the new subsection “Phages and plasmids in the human digestive tract” for more details (Please refer to Line 18, Page 24 - Line 21, Page 25 in the revised manuscript.).

Minor comments:

1. Page 5, line 13: "However, research has shown that viral sequences are highly fragmented in the metagenome [19], which may prevent binning, thereby limiting the usage of MARVEL." This is an unfair affirmation, since its only supporting reference is a 2013 article and it is safe to say that much has been done to improve metagenomic assemblers since then. Recent publications have been reporting the retrieval of phage complete and/or almost complete genomes by only applying assembly and binning approaches. I refer specially to the IMG-VR database (versions 1.0 and 2.0), which is a repository of thousands of viral sequences retrieved from metagenomic datasets all around the world. Nonetheless, there are many other publications in this line such as:
Paez-Espino, David, et al. "IMG/VR: a database of cultured and uncultured DNA Viruses and retroviruses." Nucleic acids research (2016): gkw1030.
Paez-Espino, David, et al. "IMG/VR v. 2.0: an integrated data management and analysis system for cultivated and environmental viral genomes." Nucleic acids research (2018).
Paez-Espino, David, et al. "Uncovering Earth's virome." Nature 536.7617 (2016): 425.
Sangwan, Naseer, Fangfang Xia, and Jack A. Gilbert. "Recovering complete and draft population genomes from metagenome datasets." Microbiome 4.1 (2016): 8.
Herein, we agree with Reviewer 2 so that we could have an appropriate way to describe this point. We thus have revised these sentences in an appropriate way to “The tool MARVEL can assign metagenomic bins as phages or bacteria and demonstrates better performance than previous tools. In the other hand, in order to identify sequences from low-abundance phages, which may fall into binning, we also need tools that can directly judge each fragment.” (Please refer to Line 12-16, Page 5, Section “Introduction” in the revised manuscript.) Also, we have removed the sentence “Compared with the other tools, VirFinder is more suitable for metagenomes” from Section “Introduction”.

2. Page 27, line 12 and other parts of the article: It is not clear whether authors extracted prophages from chromosomes to train the algorithm with more phages or to remove noise from chromosome datasets. Do the chromosome datasets still contain their prophages?
We realize that we did not provide a clear description of the dataset construction. In fact, both adding more phages and removing noise from the chromosome dataset are the reasons that we extracted the prophages from the chromosomes. On the one hand, the number of phage genomes in the current RefSeq database is much less than that of bacterial chromosomes, while the abundance of viruses in real microbial communities is estimated to be much higher than that of bacteria. At the time we constructed PPR-Meta, we downloaded 10,090 complete prokaryote chromosomes, while only 2,279 completed phages were collected. Thus, extracting prophages from prokaryote chromosomes may be a good approach to expand the phage training set. On the other hand, some bacteria contain several prophages, which may account for up to 20% of the host chromosome. If all of these prophages were labelled as chromosomes when training PPR-Meta, the accuracy would be reduced, especially the sensitivity of phage identification. Thus, in the training set, all of the extracted prophages were directly added to the phage dataset, and the chromosome dataset did not contain prophages. However, all these prophages were predicted by ProphET, a

Powered by Editorial Manager® and ProduXion Manager® from Aries Systems Corporation

software to extract prophages from prokaryote chromosomes based on similarity search, and were not subjected to experimental verification. Thus, these prophages could not be used as a benchmark. Therefore, in the test set, we directly removed the prophages, and neither the phage dataset nor the chromosome dataset contain the prophages, which we have emphasized in the manuscript. To test the prophage identification of PPR-Meta and the related tools, we additionally collected 267 manually annotated prophages. (Please refer to Line 2, Page 8, Subsection “Dataset construction”.) These manually annotated prophages have widely been used as benchmarks for related computational software, such as Prophinder, Phage_Finder, PHAST, PHASTER and VirSorter. To make this clearer to readers, we have added the following sentence to the revised manuscript as: “Moving prophages from a chromosome dataset to a phage dataset can help to both expand the phage dataset and remove noise from the chromosome dataset.” (Please refer to Line 20-22, Page 7, Subsection “Dataset construction” in the revised manuscript.)

3. A more practical question: For each query given by the user, will the tool automatically decide which model to use for prediction? Page 12 states how authors have proceeded by each query size, but my question regards the tool's behavior.
We are sorry that we missed some details about the tool’s behaviour, and then address our response to Reviewer 2’s question as follows.
Firstly, we would like to describe some improvements that we have made in the revised version of the PPR-Meta tool, which is closely related to this comment. In the original version of PPR-Meta, we built four neural network models for sequences of different lengths. Among these neural networks, we used BiPathCNN, which contains a base path and a codon path, for model A, B and C, and we used a Fully Connected Neural Network (FNN), which takes k-mer frequencies as inputs, for model D. In the revised version of the PPR-Meta tool, we removed model D and kept model A, B and C. In practical applications, PPR-Meta uses model A to predict sequences between 100 and 400 bp, model B to predict sequences between 400 and 800 bp, and model C to predict sequences between 800 and 1200 bp. For sequences longer than 1200 bp, a scan window will move across the sequence without overlapping, and the weighted average of all windows’ predictions is calculated. The length of the window is set to 1200 bp (or less if the window is beyond the sequence boundary). For example, given a sequence of length 2500 bp, the scan window will first cover the bases from the 1st to 1200th positions, then the window will move to the bases from the 1201st to 2400th positions, and finally, the window will move to the bases from the 2401st to 2500th positions. Then, PPR-Meta uses model C, model C and model A to predict the subsequences under the first, second and third windows, respectively. To generate the final score for the whole sequence, PPR-Meta calculates the weighted average of these windows. The weights of these three windows are 1200/2500, 1200/2500 and 100/2500, respectively.
We made this change because we found that the revised version of PPR-Meta could achieve a higher performance on long sequences. For example, for sequences with a length of 30k bp, the AUCs of both the phage identification and plasmid identification are higher. In particular, the TPR of phages increases from 93.76% to 99.84%, and almost all phages were identified. Although most sequences in the current metagenomic data are short fragments, a few reads from high-abundance species can be assembled into long contigs containing tens of thousands of bases, and we think that the revised PPR-Meta can be better adapted to these species. Additionally, considering that the third-generation sequencing technology is becoming more and more widely used, we hope that PPR-Meta can also promote studies using long sequencing technology, even though PPR-Meta is designed primarily for the next-generation sequencing technology. In the revised manuscript, we have added comparisons between PPR-Meta and the related tools using 15k bp and 30k bp sequences, which are much longer than the sequences used for the comparisons in the main text. The results showed that PPR-Meta was still the best performing tool. (Please refer to Additional File 3, Figure S1.) In addition, we also used real metagenomic data of viromes generated from third-generation sequencing technology. The results showed that PPR-Meta could identify more sequences as phages compared with the related tools. (Please refer to Line 6-16, Page 24, in the “Evaluation in real metagenomic data” section.)
We now answer the question in this comment. Actually, the PPR-Meta tool’s behaviour is the same as our above description about the practical applications of PPR-Meta. To make the PPR-Meta tool’s behaviour clearer, we have added the following sentences

Powered by Editorial Manager® and ProduXion Manager® from Aries Systems Corporation

to the revised manuscript as: “In practical applications, PPR-Meta uses BiPathCNN A to predict sequences between 100 and 400 bp, BiPathCNN B to predict sequences between 400 and 800 bp, and BiPathCNN C to predict sequences between 800 and 1200 bp. For sequences longer than 1200 bp, such as sequences in Group D, a scan window will move across the sequence without overlapping, and the weighted average of all windows’ predictions is calculated. The length of the window is set to 1200 bp (or less if the window ends beyond the sequence boundary). For example, given a sequence of length 2500 bp, the scan window will first cover the bases from the 1st to 1200th positions, then the window will move to bases from the 1201st to 2400th positions, and finally, the window will move to bases from the 2401st to 2500th positions. Then, PPR-Meta uses BiPathCNN C, BiPathCNN C and BiPathCNN A to predict the subsequences under the first, second and third windows, respectively. To generate the final score for the whole sequence, PPR-Meta calculates the weighted average of these windows. The weights of these three windows are 1200/2500, 1200/2500 and 100/2500, respectively.” (Please refer to Line 15, Page 12-Line 8, Page 13, Subsection “Structure of deep learning neural networks” in the revised manuscript.)

Because of the improvements we made in the revised PPR-Meta tool, as we mentioned at the beginning of this response, some of the results in the manuscript have also been updated. Herein, we would like to describe the updated content in our manuscript that is the result of these changes. None of the updated results mentioned below affect any conclusions that we have made in this manuscript. The revised version of the PPR-Meta tool has slight differences only on long sequences, while most of the test data we used in the manuscript are shorter than 5k bp, which is dominant in the current metagenomic sequences, and the revised PPR-Meta generates the same results for sequences shorter than 5k bp. Thus, the magnitude of all of the changes is small, except that the program has a longer running time for sequences longer than 5k bp, as shown in item (15) below.

The changes in the manuscript include the following:

(1). The original Figure 2, which describes the structure of the FNN, was removed. The second paragraph form the last in Subsection “Structure of deep learning neural networks”, which describes the FNN, was also removed.

(2). In Subsection “Mathematical model of DNA sequences”, the sentence “Here, we use a more detailed approach to represent the short sequences in Group A, Group B and Group C.” has been revised to “Here, we use a more detailed approach to represent the DNA fragments.”(Please refer to Line 7-8, Page 9 in the revised manuscript.) Also, the last paragraph of this section, which described using k-mer to represent DNA fragments in Group D, was removed.

(3). In Subsection “Structure of deep learning neural networks”, the sentences “…we trained corresponding neural networks for each group. For Group A, B and C, we designed BiPathCNN to improve the performance (Figure 1).” has been revised to: “…we trained three neural networks for Group A, B and C. To improve the performance, we designed BiPathCNN (Figure 1), a novel neural network structure, to make reliable predictions.”(Please refer to Line 15-17, Page 10 in the revised manuscript.)

(4). In Figure 2 in the revised manuscript (as Figure 3 in the original manuscript), the confusion matrix of Group D was updated. Also, in Subsection “Overall performance”, the phrase “shown in Figure 3” has been revised to “shown in Figure 2”. (Please refer to Line 15, Page 13 in the revised manuscript.)

(5). In Figure 4, the ROCs of Group D, which described the potential of using life_score and trans_score to classify the phage lifestyle and plasmid transmissibility, were updated. Also, the legend of Figure 4 has been revised to: “(a) Classify virulent phages and temperate phages using life_score. In order of sequence length, the AUC is 0.63, 0.69, 0.71 and 0.76. (b) Classify transmissible plasmid and non-transmissible plasmid using trans_score. In order of sequence length, the AUC is 0.58, 0.55, 0.60 and 0.62.

(6). In Section “Methods”, Line 21-22, Page 33, the sentence “Considering the memory size, running time and accuracy, a total of 3,060,000 artificial contigs were generated to train PPR-Meta.” has been revised to “Considering the memory size, running time and accuracy, a total of 2,700,000 artificial contigs were generated to train PPR-Meta.” Also, the phrase “and 120,000 from Group D” has been removed from the sentence “The number of training contigs of each phage, chromosome and plasmid is 300,000 from Group A to C and 120,000 from Group D.” (Please refer to Line 22, Page 33-Line 2, Page 34 in the revised manuscript.)

(7). In Table 1, Table 3 and Table 4, the performance of PPR-Meta on Group D (the fifth row from the last of each table) was updated.

(8). In Table 5, the prophage recognition rate of PPR-Meta on Group D (the third row from the last) was updated.
(9). In Subsection "Performance comparison", the sentence "The TPR of PPR-Meta was approximately 3%~13% higher than that of VirFinder and the FPR was approximately 6%~9% lower" has been revised to "The TPR of PPR-Meta was approximately 10% higher than that of VirFinder, and the FPR was approximately 5~10% lower." (Refer to Line 13-15, Page 15 in the revised manuscript.)
(10). In Subsection "Performance comparison", the sentences "For PPR-Meta, our FPR was much lower than that of cBar and PlasFlow. Although PPR-Meta achieved a slightly lower TPR than PlasFlow in Group A, our TPR remained highest in all other cases" have been revised to "For PPR-Meta, the TPR was comparable with that of PlasFlow, while the FPR was approximately 25~40% lower." (Please refer to Line 1-2, Page 16 in the revised manuscript.)
(11). In Subsection "Evaluation in real metagenomic data", the sentence "VirFinder and PPR-Meta were much better than VirSorter and identified 68.86% and 76.88% of the contigs, respectively, showing that PPR-Meta had the highest coverage of this data set" has been revised to "VirFinder and PPR-Meta were much better than VirSorter and identified 68.86% and 76.90% of the contigs, respectively, showing that PPR-Meta had the highest coverage of this data set." (Refer to Line 21, Page 21 in the revised manuscript.)
(12). In Subsection "Evaluation in real metagenomic data", the sentences "For PPR-Meta, total of 82.00% of the sequences were identified as MGEs, in which 49.16% were phages and 32.84% were plasmids. More than half of the sequences (64.72%) predicted as phages by PPR-Meta were also predicted as phages by VirFinder, and most of the sequences (74.72%) predicted as plasmids by PPR-Meta were also predicted by PlasFlow" have been revised to "For PPR-Meta, total of 81.96% of the sequences were identified as MGEs, in which 49.18% were phages and 32.78% were plasmids. More than half of the sequences (64.73%) predicted as phages by PPR-Meta were also predicted as phages by VirFinder, and most of the sequences (74.74%) predicted as plasmids by PPR-Meta were also predicted by PlasFlow." (Please refer to Line 16-20, Page 22 in the revised manuscript.)
(13). In Subsection "Evaluation in real metagenomic data", the sentence "In terms of phage identification, PPR-Meta, VirFinder and VirSorter predicted an average of 4.18%, 11.03% and 0% of the 16S-like contigs as phages, respectively, indicating that PPR-Meta likely generated fewer false positive predictions than VirFinder" has been revised to "In terms of phage identification, PPR-Meta, VirFinder and VirSorter predicted an average of 3.43%, 11.32% and 0% of the 16S-like contigs as phages, respectively, indicating that PPR-Meta likely generated fewer false positive predictions than VirFinder." (Please refer to Line 17, Page 23 in the revised manuscript.)
(14). In Subsection "Evaluation in real metagenomic data", the sentence "In terms of plasmid identification, PPR-Meta, PlasFlow and cBar predicted an average of 25.46%, 53.74% and 63.83% of the 16S-like contigs as plasmids, respectively, indicating that the PPR-Meta may generate the lowest number of false positive predictions" has been revised to "In terms of plasmid identification, PPR-Meta, PlasFlow and cBar predicted an average of 26.69%, 52.57% and 63.36% of the 16S-like contigs as plasmids, respectively, indicating that the PPR-Meta may generate the lowest number of false positive predictions." (Refer to Line 22, Page 23 in the revised manuscript.)
(15). In Subsection "Usage of PPR-Meta", the sentence "We tested the running time of PPR-Meta using 90,000 sequences from 100 to 10k bp and found that this tool can handle all sequences in approximately 15 minutes on a machine with the following configuration: CPU: Intel Core i7 6700; GPU: NVIDIA GTX1060; and Memory: 64G, DDR4" has been revised to: "… and found that this tool can handle all sequences in approximately 45 minutes on a machine with the following configuration …"(Please refer to Line 10, Page 27 in the revised manuscript.)
In items (13) and (14), the results of the comparative tools were also slightly different because we found that one setting in the data pre-processing script may not be the best option. Thus, we re-ran the pre-processing procedure to make the results more precise. The dataset pre-processing procedure is provided in Section "Methods" (Please refer to Line 3-16, Page 34 in the revised manuscript), and the data pre-processing script (file "20_gut.sh") is stored on our website and in the GigaScience Database.

To Reviewer #3:

Powered by Editorial Manager® and ProduXion Manager® from Aries Systems Corporation

General Comments:
1. This paper describes PPR-Meta - a tool for identifying phages and plasmids from metagenomic fragments using deep learning. The authors distribute the software through their own site as Virtual Machine and through Github as a set of Matlab and h5 model files. The distribution is very nonintuitive and unconventional. It would be far better for the authors to distribute as a standard git package, rather than as a .zip file. Furthermore, the requirement that the use download a 30gb Virtual Machine image is odd and not at all standard in bioinformatics. The installation of PPR-Meta should be possible without having to instantiate an entire VM. I would greatly prefer a makefile and configure script with the requirement of a Ubuntu OS, over downloading a 30gb over http. This may qualify as a major revision, because I assume switching to this would require a lot of additional work.
Herein we thank Reviewer 3 for this helpful suggestion on the distribution of the PPR-Meta tool. Following this suggestion, we have distributed PPR-Meta as a standard git package, and an archival copy of the PPR-Meta package is also available via our website. Users can directly download PPR-Meta using the command “git clone https://github.com/zhenchengfang/PPR-Meta.git” and the executable file is under the folder. We have also updated the file “README.md” in GitHub.
However, we are sorry that it is hard for us to provide a configure script for a "one click"-type installation. PPR-Meta relies on two Python Deep Learning libraries, TensorFlow and Keras, whose installations also rely on related packages in the operating system. In addition, if users want to speed up the program with a GPU, they need to install additional support software, as we mentioned in the manual. Thus, some details of the PPR-Meta installation may be slightly different under different system environments. Actually for computer professionals, it is not difficult to install the program following the manuals of PPR-Meta and the dependent packages. In order to make PPR-Meta more user-friendly for non-computer professionals, we followed the suggestion of the journal’s Author Guidelines to optimize PPR-Meta in a virtual machine. We also provided a brief step-by-step video guide to show how to run PPR-Meta in the virtual machine. Therefore in this way, any non-computer professional who is not familiar with the command line can easily run PPR-Meta on a local PC.
In the revised version of our work, the virtual machine has also been reconfigured. To minimize the size of the virtual machine, we used the “Minimal installation” mode to configure the virtual machine. We further compressed the virtual machine into a “.7z” file, which can easily be decompressed using a current compressing software such as “WinRAR”. Now, the size of the virtual machine has been reduced from 30 GB to about 2.5 GB, more than 10 times smaller and we stored the virtual machine on our website. In addition, according to the journal’s requirements, copies of the PPR-Meta package and virtual machine are also stored in the GigaScience database. In the future, we will update the packages stored on different websites synchronously.

2. There are some basic issues in the paper in detecting phage vs. prophage vs. plasmid. I think the paper leans heavily on phage and plasmid databases, over which it excels in a metagenomic context, but doesn't address the large corpus of prophage and genomic island databases in the literature. I would prefer these to be addressed and compared. The entire literature on genomic islands, including experimental, feature-based and machine-learning based methods for identification are largely missing. The authors use ProphET to identify the prophages. This is a machine learning based approach, and not a structure or experimental-based gold standard dataset.
Herein we understand the consideration of Reviewer 3 about how PPR-Meta and the related tools perform in the large corpus of prophage and genomic island databases. Indeed, in our original manuscript, we used only 267 manually annotated prophages from Casjens to evaluate PPR-Meta and the related tools.
Firstly, we would like to explain why we used only 267 manually annotated prophages and ignored the other prophage databases. We looked at several prophage databases and found that most prophages in the current prophage databases were predicted by related computational software and had not been experimentally verified. Thus, these prophages may not be appropriate to be used as benchmarks. For example, the prophages in the ACLAME database were predicted by the Prophinder tool; the prophages in the PHASTER database were predicted by the PHASTER tool; the prophages in the PHANTOME database were predicted by several tools, while only a few of them were manually verified; the prophages in ProphageDB were predicted using similarity searches (see Srividhya et al. (2006). Database and comparative

| | |
|---|---|
| | identification of prophages. Lec. Notes Control Informat. Sci.) and we failed to access its website (http://bicmku.in:8082/prophagedb; http://ispc.weizmann.ac.il/prophagedb). In contrast, the 267 manually annotated prophages are of high quality and have widely been used as benchmarks for computational software, such as Prophinder, Phage_Finder, PHAST, PHASTER and VirSorter. Thus, to make the evaluation more reliable, we used only these 267 manually annotated prophages in our original manuscript. During the revision of the manuscript, we found that the PHANTOME database also contains some prophages that were manually verified. Thus, as a supplement, we have used these prophages to evaluate PPR-Meta and the related tools in the revised manuscript. The newly collected dataset contained 139 prophages from 50 bacteria, and most of the hosts of these prophages were not the same as those of the previous 267 prophages. Therefore, the prophages from PHANTOME can be regarded as a new dataset. The results are shown in Additional File 3, Figure S4. Consistent with the results on the 267 prophages, the prophage recognition rate of PPR-Meta on the prophages from PHANTOME was about 20% higher than that of the comparative tools, indicating that PPR-Meta can identify more prophages than the related tools. We have added the following sentences to the revised manuscript as: "We additionally collected 139 manually verified prophages from the PHANTOME database [42], and most of the hosts of these prophages were not the same as those of the previous 267 prophages. Consistent with the results for the 267 prophages, the prophage recognition rate of PPR-Meta was much higher than that of the comparative tools (shown in Additional File 3, Figure S4), indicating that PPR-Meta can identify more prophages." (Please refer to Line 5-12, Page 20, Subsection "Prophage identification ability" in the revised manuscript.) The new citation has also been added to the list of the References.<br>Secondly, we would like to explain why we excluded the genomic island databases in our original manuscript. Compared with the prophages, the mechanisms of genomic islands are more diverse. The formation of genomic islands may be caused by various elements such as phages, plasmids, conjugative transposons, integrative and conjugative elements (ICEs), as well as nonreplicative but excisable elements. Since PPR-Meta is designed primarily for phage and plasmid identification, identifying some types of genomic islands seems to be another new work for this study because not all genomic islands are closely related to phages or plasmids. However, phages and plasmids are two of the most important elements that mediate the formation of genomic islands, and we quite agree with Reviewer 3 that we should discuss how the related tools perform in genomic islands and make a comparison. Therefore, we collected sequences of genomic islands from the Islander database. Upon testing on artificial contigs extracted from these genomic islands, the results showed that PPR-Meta could identify more genomic islands as foreign DNA (either phages or plasmids) than VirFinder, VirSorter, PlasFlow or cBar. This result indicated that PPR-Meta had a better ability to recognize sequences from regions of horizontal origin on bacterial chromosomes. We have added the following sentences to Section "Discussion and conclusions" in the revised manuscript as: "In general, bacteria contain genomic islands, regions of horizontal origin on chromosomes [54]. The formation mechanisms of some genomic islands are caused by phages or plasmids [55]. To see how PPR-Meta and related tools perform on DNA fragments from these regions, we collected genomic island sequences from the Islander database [56]. Upon testing on artificial contigs between 100 bp and 10k bp extracted from these genomic islands, the results showed that PPR-Meta could identify 65.25% of them as foreign DNA (either phage or plasmid), while VirFinder, VirSorter, PlasFlow and cBar could identify 20.46%, 6.72%, 53.11% and 51.62% of them, respectively, indicating that PPR-Meta has a better ability to recognize sequences from regions of horizontal origin on bacterial chromosomes." (Please refer to Line 5-15, Page 31 in the revised manuscript.) The new citations have also been added to the list of the References.<br>For the last point in this comment, we have noted that the prophages predicted by ProphET could not be used as standard dataset. As we mentioned in the manuscript, we used only these predicted prophages as a training set to expand the phage dataset, not as a test set. In the test set, all prophages predicted by ProphET were removed. (Please refer to Line 22, Page 7-Line 2, Page 8, Subsection "Dataset construction".) We evaluated only the prophage identification using manually annotated prophages, as we mentioned above. Thus, the evaluation of the prophages is reliable.<br><br>3. Figures 1 and 2, describing the bi-path convolutional network could be considerably clearer. Specifically the whole process would be clearer if the "base one-hot matrix" |

and "codon one-hot matrix" were better understood. Furthermore, the description of layers should be clear enough in the figure that they are unnecessary in the text. Finally, justification for the NN architecture is not offered. Is this a standard architecture? Was it randomly created? Was it optimized?
Herein we apologize that we did not provide a clear description of the Neural Network construction. We then address our response to Reviewer 3's comment as follows. Firstly, we would like to describe the improvements that we made in the revised version of the PPR-Meta tool, which is closely related to this comment and also is similar to Reviewer 1's comment. In the original version of PPR-Meta, we built four neural network models for sequences of different lengths. Among these neural networks, we used BiPathCNN, which contains a base path and a codon path, for model A, B and C, and we used a Fully Connected Neural Network (FNN), which takes k-mer frequencies as inputs, for model D. In the revised version of the PPR-Meta tool, we removed model D and kept model A, B and C. In practical applications, PPR-Meta uses model A to predict sequences between 100 and 400 bp, model B to predict sequences between 400 and 800 bp, and model C to predict sequences between 800 and 1200 bp. For sequences longer than 1200 bp, a scan window will move across the sequence without overlapping, and the weighted average of all windows' predictions is calculated. The length of the window is set to 1200 bp (or less if the window is beyond the sequence boundary). For example, given a sequence of length 2500 bp, the scan window will first cover the bases from the 1st to 1200th positions, then the window will move to the bases from the 1201st to 2400th positions, and finally, the window will move to the bases from the 2401st to 2500th positions. Then, PPR-Meta uses model C, model C and model A to predict the subsequences under the first, second and third windows, respectively. To generate the final score for the whole sequence, PPR-Meta calculates the weighted average of these windows. The weights of these three windows are 1200/2500, 1200/2500 and 100/2500, respectively.
We made this change because we found that the revised version of PPR-Meta could achieve a higher performance on long sequences. For example, for sequences with a length of 30k bp, the AUCs of both the phage identification and plasmid identification are higher. In particular, the TPR of phages increases from 93.76% to 99.84%, and almost all phages were identified. Although most sequences in the current metagenomic data are short fragments, a few reads from high-abundance species can be assembled into long contigs containing tens of thousands of bases, and we think that the revised PPR-Meta can be better adapted to these species. Additionally, considering that the third-generation sequencing technology is becoming more and more widely used, we hope that PPR-Meta can also promote studies using long sequencing technology, even though PPR-Meta is designed primarily for the next-generation sequencing technology. In the revised manuscript, we have added comparisons between PPR-Meta and the related tools using 15k bp and 30k bp sequences, which are much longer than the sequences used for the comparisons in the main text. The results showed that PPR-Meta was still the best performing tool. (Please refer to Additional File 3, Figure S1.) In addition, we also used real metagenomic data of viromes generated from third-generation sequencing technology. The results showed that PPR-Meta could identify more sequences as phages compared with the related tools. (Please refer to Line 6-16, Page 24, Subsection "Evaluation in real metagenomic data" in the revised manuscript.)
We now answer the questions in this comment. Because of the changes we made, the original Figure 2, which described the FNN, was removed because the FNN was not included in the revised PPR-Meta tool. Following the advice of Reviewer 3, we thus have marked the details of the related hyperparameters of each layer in Figure 1, such as the number of convolution kernels and the length of the kernels. We still maintained the related description in the text to make it easier to understand by readers who are unfamiliar with deep learning.
For "one-hot", we are sorry that we missed some of the details in the manuscript. In fact, "one-hot" is one of the most widely used encoding forms for each character in a given string in the field of Natural Language Processing (NLP), and it is also used to represent bases or amino acids in biological sequences. "One-hot" refers to a vector containing several bits in which only one bit is 1 and the others are 0. For example, given a string containing 10 types of character, the "one-hot" vectors for each character contain 10 bits. For a specific character type, the corresponding bit of the "one-hot" vector is 1, and the remaining 9 bits are 0. It is not important which bit a specific character type corresponds to, but there must be a one-to-one correspondence between each character type and each bit. In PPR-Meta, we used the "one-hot"

Powered by Editorial Manager® and ProduXion Manager® from Aries Systems Corporation

vectors [0,0,0,1], [0,0,1,0], [0,1,0,0] and [1,0,0,0] to represent each base and "one-hot" vectors containing 64 bits to represent each codon. That is, one base corresponded to one bit or one codon corresponded to one bit. To make it clearer for readers, we have added the following sentences to the "Mathematical model of DNA sequences" section of the revised manuscript as: ""One-hot" is one of the most widely used encoding forms for each character in a given string in the field of Natural Language Processing (NLP) [36], and it is also used to represent bases or amino acids in biological sequences. A "one-hot" vector contains several bits, and the number of bits is equal to the number of character types in a given string. For each character type, the corresponding bit of the "one-hot" vector is 1 and the remaining bits are 0, and there must be a one-to-one correspondence between each character type and each bit." (Please refer to Line 9-16, Page 9 in the revised manuscript.) We have also cited a reference that introduces the "one-hot" encoding in the above description so that readers who are interested in the sequence encoding form can learn more about it.

For the last point in this comment about the justification for the NN architecture, we realize that we did not give a clear description of this. In the design of PPR-Meta, the framework of BiPathCNN was proposed by ourselves while the selection of the hyperparameters of each path was referred to LeNet-5 and VGG, two classic Convolutional Neural Networks in the field of artificial intelligence. To let PPR-Meta effectively extract features from both the coding and noncoding regions, we designed the base path and the codon path to handle the "base one-hot matrix" and "codon one-hot matrix", respectively. Our test in Table 2 showed that this structure was effective because the performance of the BiPathCNN was better than that of the single-path CNN. Furthermore, we referred to LeNet-5 and VGG to optimize each path. For the layer distribution, we referred to LeNet-5. LeNet-5 contains 3 convolution layers, and there is a pooling layer between every two convolution layers. In the base path and codon path of PPR-Meta, we followed this layer distribution. However, LeNet-5 only contains 6, 16 and 120 convolution kernels in each convolution layer, which seems to be not enough for PPR-Meta to present reliable predictions. Thus, we referred to VGG for the selection of the number of convolution kernels. The number of convolution kernels in the different layers of VGG is increased by doubling, from 64, 128, 256 to 512. In PPR-Meta, we followed this distribution in the base path and codon path, that is, the first, second and third convolution layers contained 64, 128 and 256 convolution kernels, respectively. In addition, we also referred to VGG to use ReLU as an activation function. To make the construction of the neural network clearer, we have added the following sentences to the "Structure of deep learning neural networks" section of the revised manuscript as: "The selection of the related hyperparameters of each path mentioned above was referred to LeNet-5 [37] and VGG [38], two classic Convolutional Neural Networks in the field of artificial intelligence. Specifically, the distribution of layers was referred to LeNet-5, which contained 3 convolution layers, and there was a pooling layer between every two convolution layers. Meanwhile, the distribution of the number of convolution kernels was referred to VGG, in which the number of convolution kernels in the different layers was increased by doubling. We also referred to VGG to use ReLU as the activation function." (Please refer to Line 5-12, Page 12 in the revised manuscript.) The new citations have also been added to the list of the References. In addition, we have also uploaded the scripts for the neural network construction as well as other data preprocessing scripts on our website and the GigaScience Database. We think that this is the best way to provide access to readers who want to reproduce or improve PPR-Meta.

Because of the improvements we made in the revised PPR-Meta tool, as we mentioned at the beginning of this response, some of the results in the manuscript have also been updated. Herein, we would like to describe the updated content in our manuscript that is the result of these changes. None of the updated results mentioned below affect any conclusions that we have made in this manuscript. The revised version of the PPR-Meta tool has slight differences only on long sequences, while most of the test data we used in the manuscript are shorter than 5k bp, which is dominant in the current metagenomic sequences, and the revised PPR-Meta generates the same results for sequences shorter than 5k bp. Thus, the magnitude of all of the changes is small, except that the program has a longer running time for sequences longer than 5k bp, as shown in item (15) below.

The changes in the manuscript include the following:

(1). The original Figure 2, which describes the structure of the FNN, was removed. The second paragraph form the last in Subsection "Structure of deep learning neural networks", which describes the FNN, was also removed.

| | |
|---|---|
| | (2). In Subsection "Mathematical model of DNA sequences", the sentence "Here, we use a more detailed approach to represent the short sequences in Group A, Group B and Group C." has been revised to "Here, we use a more detailed approach to represent the DNA fragments."(Please refer to Line 7-8, Page 9 in the revised manuscript.) Also, the last paragraph of this section, which described using k-mer to represent DNA fragments in Group D, was removed.<br>(3). In Subsection "Structure of deep learning neural networks", the sentences "…we trained corresponding neural networks for each group. For Group A, B and C, we designed BiPathCNN to improve the performance (Figure 1)." has been revised to: "…we trained three neural networks for Group A, B and C. To improve the performance, we designed BiPathCNN (Figure 1), a novel neural network structure, to make reliable predictions."(Please refer to Line 15-17, Page 10 in the revised manuscript.)<br>(4). In Figure 2 in the revised manuscript (as Figure 3 in the original manuscript), the confusion matrix of Group D was updated. Also, in Subsection "Overall performance", the phrase "shown in Figure 3" has been revised to "shown in Figure 2". (Please refer to Line 15, Page 13 in the revised manuscript.)<br>(5). In Figure 4, the ROCs of Group D, which described the potential of using life_score and trans_score to classify the phage lifestyle and plasmid transmissibility, were updated. Also, the legend of Figure 4 has been revised to: "(a) Classify virulent phages and temperate phages using life_score. In order of sequence length, the AUC is 0.63, 0.69, 0.71 and 0.76. (b) Classify transmissible plasmid and non-transmissible plasmid using trans_score. In order of sequence length, the AUC is 0.58, 0.55, 0.60 and 0.62.<br>(6). In Section "Methods", Line 21-22, Page 33, the sentence "Considering the memory size, running time and accuracy, a total of 3,060,000 artificial contigs were generated to train PPR-Meta." has been revised to "Considering the memory size, running time and accuracy, a total of 2,700,000 artificial contigs were generated to train PPR-Meta." Also, the phrase "and 120,000 from Group D" has been removed from the sentence "The number of training contigs of each phage, chromosome and plasmid is 300,000 from Group A to C and 120,000 from Group D." (Please refer to Line 22, Page 33-Line 2, Page 34 in the revised manuscript.)<br>(7). In Table 1, Table 3 and Table 4, the performance of PPR-Meta on Group D (the fifth row from the last of each table) was updated.<br>(8). In Table 5, the prophage recognition rate of PPR-Meta on Group D (the third row from the last) was updated.<br>(9). In Subsection "Performance comparison", the sentence "The TPR of PPR-Meta was approximately 3%~13% higher than that of VirFinder and the FPR was approximately 6%~9% lower" has been revised to "The TPR of PPR-Meta was approximately 10% higher than that of VirFinder, and the FPR was approximately 5~10% lower." (Refer to Line 13-15, Page 15 in the revised manuscript.)<br>(10). In Subsection "Performance comparison", the sentences "For PPR-Meta, our FPR was much lower than that of cBar and PlasFlow. Although PPR-Meta achieved a slightly lower TPR than PlasFlow in Group A, our TPR remained highest in all other cases" have been revised to "For PPR-Meta, the TPR was comparable with that of PlasFlow, while the FPR was approximately 25~40% lower." (Please refer to Line 1-2, Page 16 in the revised manuscript.)<br>(11). In Subsection "Evaluation in real metagenomic data", the sentence "VirFinder and PPR-Meta were much better than VirSorter and identified 68.86% and 76.88% of the contigs, respectively, showing that PPR-Meta had the highest coverage of this data set" has been revised to "VirFinder and PPR-Meta were much better than VirSorter and identified 68.86% and 76.90% of the contigs, respectively, showing that PPR-Meta had the highest coverage of this data set." (Refer to Line 21, Page 21 in the revised manuscript.)<br>(12). In Subsection "Evaluation in real metagenomic data", the sentences "For PPR-Meta, total of 82.00% of the sequences were identified as MGEs, in which 49.16% were phages and 32.84% were plasmids. More than half of the sequences (64.72%) predicted as phages by PPR-Meta were also predicted as phages by VirFinder, and most of the sequences (74.72%) predicted as plasmids by PPR-Meta were also predicted by PlasFlow" have been revised to "For PPR-Meta, total of 81.96% of the sequences were identified as MGEs, in which 49.18% were phages and 32.78% were plasmids. More than half of the sequences (64.73%) predicted as phages by PPR-Meta were also predicted as phages by VirFinder, and most of the sequences (74.74%) predicted as plasmids by PPR-Meta were also predicted by PlasFlow." (Please refer to Line 16-20, Page 22 in the revised manuscript.) |

*Powered by Editorial Manager® and ProduXion Manager® from Aries Systems Corporation*

| | |
|---|---|
| | (13). In Subsection "Evaluation in real metagenomic data", the sentence "In terms of phage identification, PPR-Meta, VirFinder and VirSorter predicted an average of 4.18%, 11.03% and 0% of the 16S-like contigs as phages, respectively, indicating that PPR-Meta likely generated fewer false positive predictions than VirFinder" has been revised to "In terms of phage identification, PPR-Meta, VirFinder and VirSorter predicted an average of 3.43%, 11.32% and 0% of the 16S-like contigs as phages, respectively, indicating that PPR-Meta likely generated fewer false positive predictions than VirFinder." (Please refer to Line 17, Page 23 in the revised manuscript.)<br>(14). In Subsection "Evaluation in real metagenomic data", the sentence "In terms of plasmid identification, PPR-Meta, PlasFlow and cBar predicted an average of 25.46%, 53.74% and 63.83% of the 16S-like contigs as plasmids, respectively, indicating that the PPR-Meta may generate the lowest number of false positive predictions" has been revised to "In terms of plasmid identification, PPR-Meta, PlasFlow and cBar predicted an average of 26.69%, 52.57% and 63.36% of the 16S-like contigs as plasmids, respectively, indicating that the PPR-Meta may generate the lowest number of false positive predictions." (Refer to Line 22, Page 23 in the revised manuscript.)<br>(15). In Subsection "Usage of PPR-Meta", the sentence "We tested the running time of PPR-Meta using 90,000 sequences from 100 to 10k bp and found that this tool can handle all sequences in approximately 15 minutes on a machine with the following configuration: CPU: Intel Core i7 6700; GPU: NVIDIA GTX1060; and Memory: 64G, DDR4" has been revised to: "… and found that this tool can handle all sequences in approximately 45 minutes on a machine with the following configuration …"(Please refer to Line 10, Page 27 in the revised manuscript.)<br>In items (13) and (14), the results of the comparative tools were also slightly different because we found that one setting in the data pre-processing script may not be the best option. Thus, we re-ran the pre-processing procedure to make the results more precise. The dataset pre-processing procedure is provided in Section "Methods" (Please refer to Line 3-16, Page 34 in the revised manuscript), and the data pre-processing script (file "20_gut.sh") is stored on our website and in the GigaScience Database.<br><br>4. Training on simulated data, rather than on a known set of MGEs was a strange choice, I would like to see this more sufficiently explained. In the prophage identification section, only 267 manually annotated prophages are included, when there are in fact thousands of annotated prophages and genomic islands.<br>We thank Reviewer 3 for this concern about training PPR-Meta on a known set of MGEs, and we would like to explain why we chose to train PPR-Meta using simulated data. The most important reason that we trained PPR-Meta using artificial contigs extracted from sequenced complete genomes is that the source of these artificial contigs is more reliable and of high quality. Although there are some real metagenomic samples in which either phages or circular DNA are enriched, the enriched samples are often contaminated by the host chromosomes (see Roux S. et al. (2013). Assessment of viral community functional potential from viral metagenomes may be hampered by contamination with cellular sequences. Open Biol.). Besides, reagent and laboratory contamination may also affect the quality of real metagenomic data (see Salter SJ. et al. (2014). Reagent and laboratory contamination can critically impact sequence-based microbiome analyses. BMC Biol.). If we use these low-quality data containing contamination to train PPR-Meta, the performance of PPR-Meta may be reduced. Moreover, because there are seldom samples in which only chromosomes are enriched and all the extrachromosomal elements are filtered, we hard to collect negative samples to train PPR-Meta using real metagenomic data. Since no suitable real metagenome datasets with confident annotation are available as a benchmark, training PPR-Meta using artificial contigs extracted from sequenced complete genomes may be the best choice. Actually, many bioinformatic tools to analyse metagenomic data were trained or tested using simulated data, such as tools for gene prediction, sequence binning (see: (1) Lu YY. et al. (2017). COCACOLA: binning metagenomic contigs using sequence COmposition, read CoverAge, CO-alignment and paired-end read LinkAge. Bioinformatics. (2) Alneberg J. et al. (2014). Binning metagenomic contigs by coverage and composition. Nat. Methods.), short read assembly (Reference: Namiki, T. et al. (2012). MetaVelvet: an extension of Velvet assembler to de novo metagenome assembly from short sequence reads. Nucleic Acids Res.) and host prediction for metagenomic phage contigs. In particular, all the four tools that were compared with PPR-Meta in the manuscript were also constructed primarily using sequenced genomes from the NCBI database rather than a known set of MGEs from a |

real metagenomic sample. Because of the high quality of the simulated data, constructing artificial contigs is one of the main approaches to developing bioinformatics tools for metagenomic analysis.
For the prophage identification, we agree with Reviewer 3 that there are thousands of annotated prophages and genomic islands. As we mentioned in the response to General Comment #2, we only used 267 manually annotated prophages because we found that most prophages from the current prophage database were predicted by the related software and had not been verified, while these 267 manually annotated prophages were of high quality and have widely been used as benchmarks for computational software. In the revised manuscript, we additionally collected another 139 manually verified prophages from the PHANTOME database to further support our conclusion. The result shown in Additional File 3, Figure S4, showed that PPR-Meta had the best ability to identify these prophages. Further, because the formation mechanism of certain genomic islands is related to phages or plasmids, we have used genomic islands from the Islander database to evaluate how PPR-Meta and related tools perform on these genomic islands. The results showed that PPR-Meta could identify more genomic islands as foreign DNA (either phages or plasmids) than other tools, indicating that PPR-Meta had a better ability to recognize sequences from regions of horizontal origin on bacterial chromosomes. For more details, please refer to our response to General Comment #2 as well as Line 5-15, Page 31, Section "Discussion and conclusions" of our revised manuscript.

5. Finally, I would like to see the analysis of this software performed on a large metagenomic dataset. What percentage of the an ocean, fecal or soil sample is made up of phages/plasmids? Does the tool simply key in on odd codon/base pair coding, or is it keying in on larger gene-level issues? With a neural network, this is not always easy to tell, but could have fundamental impacts on how we view the metagenomics of mobile elements.
We thank Reviewer 3 for this helpful advice on using PPR-Meta to analyse a large metagenomic dataset. In the revised manuscript, we have employed PPR-Meta to identify phage and plasmid sequences on a series of metagenomic datasets of the human digestive tract from the Human Microbiome Project (HMP), including the gut, throat and oral cavity. The results are interesting and may be significant to the study of human health. In the revised manuscript, we have added a new subsection "Phages and plasmids in the human digestive tract", to present the results. (Please refer to Line 18, Page 24 - Line 21, Page 25 in the revised manuscript.)
The title and legend of Figure 3 are presented in Line 1, Page 26 as: "Figure 3. Percentages of phages, chromosomes and plasmids in the human digestive tract. PPR-Meta was used to predict the sequences of phages, chromosomes and plasmids in metagenomic assemblies, including samples from the gut, throat and oral cavity. The sequence percentages of phages, chromosomes and plasmids were calculated." The new citations have also been added to the list of the References, and the term HMP has also been add to the "List of abbreviations" section. (Please refer to Line 11, Page 36 in the revised manuscript.)
For the last point of this comment, we understand the consideration of Reviewer 3 about how PPR-Meta works. In our opinion, PPR-Meta primarily works by observing the sequence signatures such as odd codon/base pair coding, as Reviewer 3 mentioned, rather than by using information on larger gene-level issues. PPR-Meta is mainly designed for identifying phages or plasmids from metagenomic short fragments, and the results have shown that PPR-Meta can present satisfactory performance. In general, fragments shorter than 2k bp are hard to contain more than one gene or even a complete gene. If PPR-Meta works by using information on larger gene-level issues, it could not present satisfactory performance due to the insufficient number of genes for its analysis. As an example for comparison, the tool VirSorter, one of the software compared with PPR-Meta in the manuscript, is a gene-based tool, and it performs poorly. In our evaluation, VirSorter missed most phages for sequences shorter than 1.2k bp. Since VirSorter identifies phage sequences primarily by observing the distribution of genes, such as the densities of known viral genes or the enrichment of short genes, the lack of genes in short fragments may lead to the poor performance of VirSorter. Therefore, PPR-Meta does not seem to be a gene-based tool but a tool based on sequence patterns. In fact, it has been reported that plasmids and phages contain different sequence signatures from their host, such as the GC content, codon usage or k-mer frequencies, which make it possible to identify them through sequence signatures. In addition, the information of sequence signatures has also been widely

| | |
|---|---|
| | used in many sequence classification software, such as the binning algorithm for metagenomic fragments. (see Alneberg et al. (2014). Binning metagenomic contigs by coverage and composition. Nat. Methods.). Overall, in our opinion, we consider that PPR-Meta makes the judgment primarily through sequence signatures rather than larger gene-level issues.<br><br>In hoping that the above revision has clarified all the points by three reviewers and given a point-by-point response to all the concerns, we hereby resubmit our manuscript to GigaScience. We thank you for your kind consideration.<br><br>Sincerely yours,<br>Huaiqiu Zhu, Ph. D., Professor<br>Peking University |
| Additional Information: | |
| Question | Response |
| Are you submitting this manuscript to a special series or article collection? | No |
| **Experimental design and statistics**<br><br>Full details of the experimental design and statistical methods used should be given in the Methods section, as detailed in our Minimum Standards Reporting Checklist. Information essential to interpreting the data presented should be made available in the figure legends.<br><br>Have you included all the information requested in your manuscript? | Yes |
| **Resources**<br><br>A description of all resources used, including antibodies, cell lines, animals and software tools, with enough information to allow them to be uniquely identified, should be included in the Methods section. Authors are strongly encouraged to cite Research Resource Identifiers (RRIDs) for antibodies, model organisms and tools, where possible.<br><br>Have you included the information requested as detailed in our Minimum Standards Reporting Checklist? | Yes |
| **Availability of data and materials** | Yes |

Powered by Editorial Manager® and ProduXion Manager® from Aries Systems Corporation

| | |
|---|---|
| All datasets and code on which the conclusions of the paper rely must be either included in your submission or deposited in publicly available repositories (where available and ethically appropriate), referencing such data using a unique identifier in the references and in the "Availability of Data and Materials" section of your manuscript.<br><br>Have you have met the above requirement as detailed in our Minimum Standards Reporting Checklist? | |

Manuscript_revision

Click here to access/download;Manuscript;Manuscript_revision1.docx

Click here to view linked References

# PPR-Meta: a tool for identifying phages and plasmids from metagenomic fragments using deep learning

Zhencheng Fang[1,2], Jie Tan[1,2], Shufang Wu[1,2], Mo Li[1,2,3], Congmin Xu[1,2,4], Zhongjie Xie[1,2] and Huaiqiu Zhu[1,2]*

[1] State Key Laboratory for Turbulence and Complex Systems and Department of Biomedical Engineering, College of Engineering, Peking University, Beijing 100871, China

[2] Center for Quantitative Biology, Peking University, Beijing 100871, China

[3] Peking University-Tsinghua University - National Institute of Biological Sciences (PTN) joint PhD program, School of Life Sciences, Peking University, Beijing 100871, China

[4] Department of Biomedical Engineering, Georgia Institute of Technology and Emory University, Georgia 30332, USA

* To whom correspondence should be addressed.

hqzhu@pku.edu.cn

## Abstract

**Background**: Phages and plasmids are the major components of mobile genetic elements, and fragments from such elements generally co-exist with chromosome-derived fragments in sequenced metagenomic data. However, there is a lack of efficient methods that can simultaneously identify phages and plasmids in metagenomic data, and the existing tools identifying either phages or plasmids have not yet presented satisfactory performances.

**Findings**: We present PPR-Meta, a three-class classifier that allows simultaneous identification of both phage and plasmid fragments from metagenomic assemblies. PPR-Meta consists of several modules for predicting sequences of different lengths. Using deep learning, a novel network architecture, referred to as the Bi-path Convolutional Neural Network, is designed to improve the performance for short fragments. PPR-Meta demonstrates much better performance than currently available similar tools individually for phage or plasmid identification, while testing on both artificial contigs and real metagenomic data. PPR-Meta is freely available via http://cqb.pku.edu.cn/ZhuLab/PPR_Meta or https://github.com/zhenchengfang/PPR-Meta.

**Conclusions:** To the best of our knowledge, PPR-Meta is the first tool that can simultaneously identify phage and plasmid fragments efficiently and reliably. The software is optimized and can be easily run on a local PC by non-computer professionals. We developed PPR-Meta to promote the research on mobile

genetic elements and horizontal gene transfer.

**Keywords:** metagenome, mobile genetic elements, horizontal gene transfer, phage, plasmid, deep learning

## Findings

### Introduction

Phages and plasmids, known as mobile genetic elements (MGEs), are the main participants in horizontal gene transfer (HGT) along with genetic information exchanging among prokaryotes or eukaryotes [1]. Such elements can regulate the microbial community by interacting with the host. One of the important roles of MGEs is their ability to distribute resistance genes among bacteria and facilitate environmental adaptations among microbial communities [2]. In most cases, a substantial number of phage and plasmid genomes are present in the microbial community. For example, reports have shown that the abundance of marine phages even surpasses that of other organisms in marine systems, and more than half of the bacteria isolated from marine systems contain at least one plasmid [3][4]. Thus, the identification of phage and plasmid fragments in metagenomes is a fundamental issue in comprehensive analyses of HGT and the interaction between MGEs and hosts. Although experimental approaches have been developed to enrich phages or plasmids from environment samples [5][6], the enriched samples lose host information, which may hinder the

comprehensiveness of the analyses. Therefore, computational tools for directly identifying phages and plasmids from metagenomes are expected to be developed in the field.

However, the effective identification of such elements remains a considerable challenge. Currently the fragments assembly performance of both plasmid and phage from high-throughput sequencing data is not as well as that of host-derived fragments [7]. This indicates that sequences from phages or plasmids exist as a large number of short fragments, resulting in the difficulty of the identification. In addition, fewer sequenced genomes of phages and plasmids are available compared with bacterial genomes in current databases [1]. Especially, although the abundance of viruses is estimated to exceed that of other organisms on the earth [8], so far the number of phage genomes in the NCBI database is still less than one-thirtieth the number of prokaryotic genomes, and it was estimated that more than half of the sequences from viral metagenomes could not find significant homology to the released database [5]. Therefore, it is especially essential to develop a tool for identifying novel phages and plasmids from metagenomic data with a large number of mixed short reads.

Despite the difficulty of identification, several tools have been recently developed to detect either phages or plasmids from culture-dependent Whole Genome Sequencing (WGS) data or metagenomic data. Tools that detect regions from an integrated phage sequence (referred to as prophage) over a sequenced complete bacterial genome have been designed. These tools

include Prophinder [9], Phage_Finder [10], PhiSpy [11], PHAST (and its enhanced version PHASTER) [12] [13], VirSorter [14], and ProphET [15]. Such approaches primarily used a scan window to move across the complete bacterial chromosome and extract regions that seem to be phages based on a similarity search against viral databases. Because the scan windows of these tools are often required to be able to cover several genes, such tools are difficult to apply to metagenomic data since the sequences of metagenome are too short to contain even a complete gene [16]. Although VirSorter can also assign metagenomic contigs as phages or bacteria, its sensitivity of identification is quite low. Moreover, lytic phages and some temperate phages do not integrate their genomes into their host chromosomes [17], thus these tools may only be able to identify specific phages. The tool MARVEL [18] can assign metagenomic bins as phages or bacteria and demonstrates better performance than previous tools. In the other hand, in order to identify sequences from low-abundance phages, which may fall into binning, we also need tools that can directly judge each fragment. In contrast, VirFinder [19] can directly judge each sequence, and it uses a logistic regression as the classifier to detect phage sequences based on *k*-mer frequencies and presents a relatively good performance. In terms of plasmids, most of the current tools for plasmid identification were designed for WGS or even specific species, such as PlasmidFinder [20], PLACNET [21], PlasmidSeeker [22] and mlplasmids [23]. However, the plasmid identification strategy for WGS may not by applicable for

metagenomes. For example, PlasmidSeeker considers plasmid contigs to have a higher read coverage because plasmids may have copies in their hosts. In metagenome, however, the difference of read coverage among contigs may result from different abundances of species rather than copy number. The tool cBar [24] is the first tool designed primarily for plasmid identification in metagenomes. This tool applies sequential minimal optimization (SMO) as a classifier based on *k*-mer frequencies. Similar to cBar, PlasFlow [25] is also a *k*-mer-based tool for identifying plasmids. Compared with cBar, PlasFlow further combines the information of different *k*-mer lengths and uses multiple neural networks as voting devices to determine whether the sequence belongs to the plasmid, and it achieves a better performance than cBar.

Although related tools have been developed, state-of-the-art tools for detecting short fragments have not presented satisfactory performances. Moreover, because these tools can only identify either phages or plasmids, they clearly do not meet the needs of a comprehensive analysis of MGEs and HGT. Considering that poor sequence assembly performance results in a large number of short fragments, it is a practical goal to develop a higher performing tool. In this paper, we present the PPR-Meta (**P**hage and **P**lasmid **R**ecognizer for **Meta**genomes), a three-class classifier for identifying metagenomic fragments as phages, plasmids or chromosomes based on the deep learning algorithm. To achieve higher performance on short fragments, we designed a novel neural network architecture which is referred to as the Bi-path

Convolutional Neural Network (BiPathCNN). To the best of our knowledge, PPR-Meta is the first tool that can simultaneously identify phage and plasmid fragments efficiently and reliably.

**Dataset construction**

Owing to that no suitable real metagenome datasets with confident annotation are available as a benchmark, we therefore used the simulated datasets with artificial contigs generated from sequenced complete genomes. We downloaded the complete genomes of prokaryote chromosomes (total of 10,090 genomes), prokaryote plasmids (total of 8,801 genomes) and phages (total of 2,279 genomes) from the NCBI genome database [26]. The list of the genomes is provided in Additional file 1. To evaluate the ability of PPR-Meta to identify novel species, genomes released before January 2016 were employed to build the training set while the remainder used to build the test set. In general, prokaryote chromosomes may contain regions of integrated phages, referred to as prophages [27], however most genomes do not have the prophage annotation. Here, we used ProphET to extract prophages from all the prokaryote chromosomes, and a total of 16,393 prophages predicted by ProphET (shown in Additional file 2) were incorporated into the phage dataset. Moving prophages from a chromosome dataset to a phage dataset can help to both expand the phage dataset and remove noise from the chromosome dataset. Since the predicted prophages were generated by ProphET and could

not be used as a benchmark, we removed the predicted prophages from the test set. To evaluate the performance of PPR-Meta for prophage identification, we collected 267 manually annotated prophages of 54 prokaryote chromosomes from Casjens [27]. To ensure that the test data were “novel” to PPR-Meta, these prophages and their hosts were removed from the training set.

We used the MetaSim simulator [28] to extract artificial contigs from the complete genomes. Four groups of artificial contigs of different lengths were generated: Group A with a length range of 100-400 bp, Group B with a length range of 400-800 bp, Group C with a length range of 800-1200 bp and Group D with a length range of 5000-10000 bp. Group A, B and C were constructed to simulate the length obtained with different sequencing technology and the average assembly contig length, while Group D was constructed to simulate long contigs in metagenomic data.

We also used real metagenomic data to estimate the reliability of PPR-Meta. The real data included phage metagenomic data of bovine rumen [29], which were downloaded from MG-RAST [30] (Accessions: mgm4534202.3 and mgm4534203.3) as raw reads and assembled by SPAdes [31]; plasmid metagenomic data of bovine rumen [32], downloaded from MG-RAST (accessions: mgm4460391.3); and 20 samples of healthy human gut [33], downloaded from the NCBI Short Read Archive [34] and assembled by SPAdes. The accessions of the human gut samples are shown in Additional file 1.

Additional details on the dataset construction are provided in Methods section.

**Mathematical model of DNA sequences**

The method of representing biological sequence is significant for every machine learning-based tool. Although *k*-mer frequencies have been widely used in many studies [19], such frequencies may present serious fluctuations in short sequences [35]. Here, we use a more detailed approach to represent the DNA fragments. Specifically, each sequence is represented by "base one-hot matrix (BOH)" and "codon one-hot matrix (COH)". "One-hot" is one of the most widely used encoding forms for each character in a given string in the field of Natural Language Processing (NLP) [36], and it is also used to represent bases or amino acids in biological sequences. A "one-hot" vector contains several bits, and the number of bits is equal to the number of character types in a given string. For each character type, the corresponding bit of the "one-hot" vector is 1 and the remaining bits are 0, and there must be a one-to-one correspondence between each character type and each bit. For BOH in PPR-Meta, bases A, C, G and T are represented by [0,0,0,1], [0,0,1,0], [0,1,0,0], and [1,0,0,0], respectively. Therefore, together with the complementary strand, a sequence of length L can be represented by a BOH matrix of length 2×L and width 4. For COH, each sequence is first expanded to six phases in the form of codons. For example, sequence 5'-ACGTTCGAACG-3' will be split into the following six codon sequences:

1): ACG, TTC, GAA

2): CGT, TCG, AAC

3): GTT, CGA, ACG

4): CGT, TCG, AAC

5): GTT, CGA, ACG

6): TTC, GAA, CGT

Similar to BOH, each codon of COH is represented by a 64 dimensional one-hot vector, namely one certain position is 1 and the other positions are 0. Therefore, a sequence of length L can be represented by a COH matrix of length 2×L and width 64. Both BOH and COH will be used as input for the neural networks mentioned below.

**Structure of deep learning neural networks**

To ensure that PPR-Meta optimally adapts to sequences of different lengths, we trained three neural networks for Group A, B and C. To improve the performance, we designed BiPathCNN (Figure 1), a novel neural network structure, to make reliable predictions. BiPathCNN contains a “base path” and a “codon path”, which take BOH and COH as inputs respectively. After multiple convolution operations, the data for the two paths are combined by a merge layer. The fully connected layers then receive the merged data and finally output three scores that reflect the likelihood of the input fragment as a phage, chromosome or plasmid.

**Figure 1. Structure of BiPathCNN.** Three BiPathCNNs were trained for sequences from Group A, B and C. Each BiPathCNN contains a “base path” and a “codon path”, which take BOH and COH as inputs respectively.

The details of each layer are described as follows.

Layer b1 and layer c1: one-dimensional convolutional layers with 64 convolution kernels using “ReLU” (Rectified Linear Unit) as the activation function. The ReLU function can be expressed as y=max(0,x). These layers take BOH or COH as inputs. The length of the convolution kernels is set to 6.

Layer b2 and layer c2: max pooling layers with a pooling length set to 3.

Layer b3 and layer c3: batch normalization layers with the dropout operation. Each element of the feature map from previous layer in each batch will be normalized, which can speed up the convergence and prevent overfitting.

Layer b4~b6 and layer c4~c6: similar to layers b1~b3 or layers c1~c3, respectively. We set the number of convolution kernels in layer b4 and c4 as 128 and the length of the kernels as 3.

Layer b7 and layer c7: one-dimensional convolutional layers containing 256 convolution kernels and using ReLU as the activation function. The length of the convolution kernels is set to 3.

Layer b8 and layer c8: one-dimensional global average pooling layers that output the global average for each feature map of the previous layer.

Layer 9 to layer 11: The concatenation layers combine the output of the "base path" and "codon path". After the full connection layer with the same number of nodes as the previous layer, the softmax layer calculates the probability of the input fragment as a phage, chromosome or plasmid.

The selection of the related hyperparameters of each path mentioned above was referred to LeNet-5 [37] and VGG [38], two classic Convolutional Neural Networks in the field of artificial intelligence. Specifically, the distribution of layers was referred to LeNet-5, which contained three convolution layers, and there was a pooling layer between every two convolution layers. Meanwhile, the distribution of the number of convolution kernels was referred to VGG, in which the number of convolution kernels in the different layers was increased by doubling. We also referred to VGG to use ReLU as the activation function. All the neural networks used Adam as the optimizer and cross-entropy as the loss function.

In practical applications, PPR-Meta uses BiPathCNN A to predict sequences between 100 and 400 bp, BiPathCNN B to predict sequences between 400 and 800 bp, and BiPathCNN C to predict sequences between 800 and 1200 bp. For sequences longer than 1200 bp, such as sequences in Group D, a scan window will move across the sequence without overlapping, and the weighted average of all windows' predictions is calculated. The length of the window is set to 1200 bp (or less if the window ends beyond the sequence boundary). For example, given a sequence of length 2500 bp, the scan window

will first cover the bases from the 1st to 1200th positions, then the window will move to bases from the 1201st to 2400th positions, and finally, the window will move to bases from the 2401st to 2500th positions. Then, PPR-Meta uses BiPathCNN C, BiPathCNN C and BiPathCNN A to predict the subsequences under the first, second and third windows, respectively. To generate the final score for the whole sequence, PPR-Meta calculates the weighted average of these windows. The weights of these three windows are 1200/2500, 1200/2500 and 100/2500, respectively.

**Overall performance**

We evaluated PPR-Meta according to four groups of test sets with different lengths of short contigs. For each fragment input, the algorithm calculates three scores representing the likelihood that the fragment should be identified as a phage, plasmid or chromosome. Therefore, the category with the highest score is selected as our prediction. We used three-class confusion matrices (shown in Figure 2) to evaluate the overall performance of PPR-Meta. In general, PPR-Meta had a better discrimination ability when the sequences were longer, and the phage recognition ability of PPR-Meta was better than the plasmid recognition ability. Plasmid sequences were easily confused with the host chromosomes, which may because phages and plasmids face different evolutionary pressures. Since plasmid must survive in host cells, they may adapt their sequence signatures, such as the GC content and codon usage, to

their hosts. In contrast, phages can assemble their own particles and remain outside of the hosts. Moreover, certain phages may contain their own tRNA, which allows them to change their codon usage [39]. Thus, the various similarity of phages and plasmids to their hosts may lead to differences in the identification ability of PPR-Meta. In addition, transposons may carry plasmid DNA fragments to the chromosome [1]. Therefore, the chromosome may contain regions from the plasmid. Sequences shared between the plasmid and the chromosome may also affect the judgment of PPR-Meta. Overall, PPR-Meta can effectively identify the MGEs in the test set.

**Figure 2. Confusion matrix of PPR-Meta.** Three-class confusion matrices were used to evaluate the overall performance of PPR-Meta. Four matrices correspond to the sequences of Group A to D. In each matrix, the rows represent the true category while the column represent the predicted category of PPR-Meta.

**Performance comparison**

We then compare PPR-Meta with VirFinder and VirSorter regarding the ability to identify phages, and with PlasFlow and cBar regarding the ability to identify plasmids. The evaluation criteria were the true positive rate (TPR=TP/(TP+FN)), false positive rate (FPR=FP/(TN+FP)) and area under the curve (AUC). Note that PlasFlow will filter uncertain predictions according to a default threshold.

As a uniform comparison, we turned off this feature by setting the threshold to zero, thus using all the sequences for comparison.

The results are shown in Table 1. In all cases, the AUCs of PPR-Meta were the highest. In terms of phages, VirSorter, which is a gene-based tool, performed poorly with almost all phages missed. It is probably because there is not a sufficient number of full-length genes present in short DNA fragments for VirSorter's analysis. This also indicates that methods based on homology searches of genetic information are not applicable to species identification in metagenomes. Considering that most contigs of metagenomes are short fragments especially those of MGEs, VirSorter is not competent for phage identification despite achieving a higher performance for long contigs in Group D. The tool VirFinder outperformed VirSorter. As an alignment-free tool, VirFinder achieved a much higher TPR, especially in short fragments. The TPR of PPR-Meta was approximately 10% higher than that of VirFinder and the FPR was approximately 5~10% lower. The performance improvement on short sequences demonstrates that our sequences representation method is more detailed than the *k*-mer frequencies, and the deep learning algorithm is more capable of extracting sequence features than the logistic regression used by VirFinder. In terms of plasmids, both cBar and PlasFlow did not perform well. The cBar appeared to produce random results in most cases, with both the TPR and FPR near 50%. Although the AUC of PlasFlow was slightly higher than that of cBar, PlasFlow tended to judge most sequences as plasmids, which resulted

in an extremely high FPR. For PPR-Meta, the TPR was comparable with that of PlasFlow, while the FPR was approximately 25~40% lower. In some cases, a few assembled sequences from high-abundance species may be much longer, so we also tested PPR-Meta and the related tools using 15k bp and 30k bp fragments (shown in Additional File 3, Figure S1). The results showed that the performance of PPR-Meta was still the best for these long sequences. In addition, we tested the accuracy as well as the running time of each BiPathCNN on test datasets from different groups and found that using a non-corresponding BiPathCNN to predict sequences from specific groups would lead to a lower accuracy and longer running time (shown in Additional File 3, Figure S2). Overall, PPR-Meta presented a much better performance than other homology-search-based tools such as VirSorter and k-mer-based tools such as VirFinder, PlasFlow and cBar.

**Table 1**. Evaluation of the performance of PPR-Meta and comparison of the performance of PPR-Meta and related tools.

**Effectiveness of BiPathCNN**

PPR-Meta achieved a much higher performance than the other methods as mentioned above. The innovation of PPR-Meta is the design of BiPathCNN, which uses both base and codon information to improve the performance. In BiPathCNN, the “base path” is beneficial to extracting the sequence features of

non-coding regions while the "codon path" is beneficial to extracting coding regions. To verify the effectiveness of BiPathCNN, we removed the "codon path" and "base path" and retrained PPR-Meta. The newly trained PPR-Meta was tested, and the results showed that the performance of PPR-Meta with either the "base path" or "codon path" only presented a lower performance relative to that of BiPathCNN in most cases (Table 2). Moreover, the performance of the "codon path" only CNN was better than that of "base path" only CNN, which indicates that the features that distinguish phages, chromosomes and plasmids are more concentrated in the coding region. Compared with other sequence representation methods that ignore the coding or non-coding region, such as method based on *k*-mer frequencies, PPR-Meta uses a more detailed method of describing a sequence and achieves a higher performance.

**Table 2**. Performance comparison among BiPathCNN, the base path-only CNN and codon path-only CNN.

**Performance in the presence of sequencing errors**

Sequencing errors exist in various sequencing technologies, and tools that handle high-throughput sequencing data should be able to tolerate these errors. In addition, the third-generation sequencing technology, such as PacBio and Nanopore, have much higher sequencing errors. Thus, the compatibility of tools with new sequencing technologies should be considered.

Sequencing errors can be divided into two types: base substitutions and base insertions or deletions. We tested the impact of these two types of sequencing errors on the identification performance of PPR-Meta and related tools. We used MetaSim to extract modified fragments with 1% substitutions and 1% insertions or deletions separately from the test genomes. We used the same criteria described above to compare the performance of different tools in terms of both types of error. The results are shown in Table 3 and Table 4.

In most cases, in the presence of 1% base substitutions, a slight decrease in each evaluation criterion was observed for each tool compared with that in the presence of non-sequencing errors, although the decrease was not obvious. PPR-Meta was still the best-performing tool. When 1% of the bases were inserted or deleted, the performance of most tools was slightly reduced with the exception of VirSorter. Base insertions or deletions caused significant fluctuations in the performance of VirSorter. For sequences of Group D, the AUC of VirSorter decreased by approximately 9% compared with sequences with no errors. In our opinion, the reason that VirSorter exhibits great fluctuations in performance with base insertions or deletions is that insertions and deletions disrupt the phase of the open reading frame (ORF). VirSorter identifies phage sequences primarily by observing the distribution of genes, such as the densities of known viral genes or the enrichment of short genes. Disrupting the ORF phase will severely affect gene identification [40], thereby leading to interference in the downstream analysis. Thus, although VirSorter

can achieve a relatively good performance on long contigs, caution should be taken when applying VirSorter to data generated by the third-generation sequencing technology.

Considering that the error rate of the raw data generated from the third-generation sequencing technology may be much higher, we also tested PPR-Meta and the related tools using artificial contigs modified with 10% base substitutions and 10% insertions or deletions in Group D, whose lengths were close to the raw reads generated from third-generation sequencing technology. The results are shown in Additional File 3 and Figure S3. The results showed that the AUCs of PPR-Meta remained the highest (>90%), although the performance was somewhat fluctuating, especially in the presence of 10% insertions or deletions. Recently, many basecalling tools for the third-generation sequencing technology have been developed to help improve the accuracy over 99% [41], therefore the extremely high error rate on the raw data will not affect the usage of PPR-Meta.

**Table 3**. Identification performance of each tool with 1% base substitutions.

**Table 4**. Identification performance of each tool with 1% base insertions or deletions.

**Prophage identification ability**

We tested the prophage identification ability of the related tools on the 267 manually annotated prophages. The results in Table 5 showed that although the recognition rate of PPR-Meta for prophages was lower than that for phage contigs generated from the NCBI database, the overall performance of PPR-Meta was still much better than that of VirFinder and VirSorter. We additionally collected 139 manually verified prophages from the PHANTOME database [42], and most of the hosts of these prophages were not the same as those of the previous 267 prophages. Consistent with the results for the 267 prophages, the prophage recognition rate of PPR-Meta was much higher than that of the comparative tools (shown in Additional File 3, Figure S4), indicating that PPR-Meta can identify more prophages. The lower recognition rate of prophages compared with that of the phages in the NCBI database may be due to the difference of sequence pattern between prophages and phages in the NCBI database. The complete genomes in the NCBI database tend to come from phages that are easily obtained experimentally while prophages hide their genomes in the hosts. During co-evolution, prophages may adjust the sequence pattern according to their hosts to eliminate the hosts' restriction enzymes [39]. In terms of VirFinder, the ability of identifying prophages was significantly reduced and more than half of the prophages were missed, which may be because VirFinder ignored prophages that exist in the chromosomes during training. In both the training and test set of VirFinder, all prophages were labelled as chromosomes, which led to the misjudgement of prophages. In

microbial communities, temperate phages are dominant and a significant portion of temperate phages exist in the form of prophages [43]. For example, prophage have been shown to represent the main component of phages in healthy human guts [17]. In certain prokaryotes, prophages account for up to 20% of the host chromosome [27]. Thus, compared with VirFinder, PPR-Meta may be more adapted to real microbial communities since it can identify more prophages.

**Table 5**. Recognition rate of prophages

## Evaluation in real metagenomic data

We also evaluated PPR-Meta and the related tools using real metagenomic data. We first evaluated whether PPR-Meta can identify MGEs using both phage metagenomic and plasmid metagenomic data of bovine rumens, in which either phages or plasmids were enriched before sequencing. The phage metagenomic data were downloaded as raw reads, and a total of 107,529 contigs were generated after assembly. VirSorter, VirFinder and PPR-Meta were run on the phage metagenome. Consistent with the results for artificial contigs, VirSorter missed nearly all the phages and only 0.02% of the contigs were identified. VirFinder and PPR-Meta were much better than VirSorter and identified 68.86% and 76.90% of the contigs, respectively, showing that PPR-Meta had the highest coverage of this data set.

The plasmid metagenomic data were downloaded as assembled contigs containing 5771 sequences. It is worth noting that there are a certain number of phages survive as circular DNA [17], and when enriching plasmids, these circular phages will also be extracted together with the plasmids. Thus, the plasmid metagenome may contain a mixture of phages and plasmids in which the host chromosomes are filtered. From the RefSeq viral database, we collected the genes labelled as “portal”, “spike”, “major capsid protein”, “terminase large subunit”, “tail”, “coat”, or “virion formation”, which were more likely to exist in phages [14]. We found that one of the sequences contained a homologous region of the portal protein by applying the blastx search (e-value<=1e-4), indicating that phages are likely to co-exist with plasmids in this dataset. Therefore, all of PPR-Meta, VirSorter, VirFinder, cBar and PlasFlow were run on this dataset. Results showed that VirSorter did not identify any sequences as phages while VirFinder identified 49.90% as phages. In terms of cBar and PlasFlow, they identified 64.46% and 74.67% of the sequences as plasmids. For PPR-Meta, total of 81.96% of the sequences were identified as MGEs, in which 49.18% were phages and 32.78% were plasmids. More than half of the sequences (64.73%) predicted as phages by PPR-Meta were also predicted as phages by VirFinder, and most of the sequences (74.74%) predicted as plasmids by PPR-Meta were also predicted by PlasFlow. Furthermore, the sequence containing the homologous region of the portal protein was identified as phages and 8 out of 10 sequences coding plasmid

backbone functions listed in Figure 3 of [32] were also identified as plasmids by PPR-Meta. Thus, the prediction of PPR-Meta may be reliable. Because of the filtering of chromosomes from this dataset, PPR-Meta could identify most of the extrachromosomal elements with the fewest false negative predictions.

Since we lack samples in which only chromosomes are enriched and all the extrachromosomal elements are filtered, estimating whether related tools will misjudge chromosomes as MGEs directly is difficult using real data. Because 16S rRNA is more likely to occur in chromosomes, sequences containing the homologous region of 16S rRNA are likely chromosome-derived. We collected 20 metagenome samples from the human gut, which represented mixtures of phages, chromosomes and plasmids. All contigs of the samples were searched against the 16S rRNA database of Greengeen [44] using blastn, and the contigs containing the homologous region (e-value<=1e-4, hits length>=250) of 16S rRNA were collected. Hits longer than 250 bp could cover at least one conserved region of 16S rRNA so the alignments were reliable. In terms of phage identification, PPR-Meta, VirFinder and VirSorter predicted an average of 3.43%, 11.32% and 0% of the 16S-like contigs as phages, respectively, indicating that PPR-Meta likely generated fewer false positive predictions than VirFinder. Although VirSorter did not cover any of the 16S-like contigs, the low number of false positive predictions came at the cost of missing almost all phages as shown above. In terms of plasmid identification, PPR-Meta, PlasFlow and cBar predicted an average of 26.69%, 52.57% and 63.36% of the

16S-like contigs as plasmids, respectively, indicating that the PPR-Meta may generate the lowest number of false positive predictions. Because individual extrachromosomal elements also contain ribosomal RNA, especially large plasmids [45], the coverage of 16S-like contigs may be higher than the real FPR. Overall, PPR-Meta can identify more MGEs with fewer false positives.

Considering that the third-generation sequencing technology is more and more widely used to analyse metagenomes, we also used real virome data generated by MinION [46] to test whether PPR-Meta and the related tools can identify phages from third-generation sequencing technology. The virome was downloaded as assembled sequences (accession: GCA_900491955.1), containing 1500 sequences. The results showed that PPR-Meta, VirFinder and VirSorter could identify 79.20%, 76.27% and 30.40% of viral sequences respectively, indicating that PPR-Meta has the highest performance. Therefore PPR-Meta can also handle data from the third-generation sequencing technology, although it is designed primarily for the next-generation sequencing technology.

**Phages and plasmids in the human digestive tract**

As an application example, we employed PPR-Meta to analyse the percentages of phages, bacterial chromosomes and plasmids in microbial communities from the human digestive tract. We collected 10 samples from the gut (sampling from stools), 7 samples from the throat and 10 samples from the oral cavity

(sampling from the tongue dorsum). All samples were downloaded from the Human Microbiome Project (HMP) [47] as assembled contigs. The accessions of all samples are provided in Additional File 1. PPR-Meta was run on all samples, and the percentages of sequences predicted as phages, chromosomes and plasmids were calculated. The results are shown in Figure 3. We found that in the positions closer to the outer end of the digestive tract, the percentages of phages and plasmids tended to be higher. For example, in the gut, the inner end of the digestive tract, the percentages of phages and plasmids were lower, while in the oral cavity, the outer end of the digestive tract, the percentages were higher. Especially, phage sequences occupied approximately 14.80% of all sequences in the gut, which was consistent with the viral proportion in the human gut (4~17%) estimated earlier [48]. In the oral cavity, the percentage of phage sequences was obviously higher, occupying approximately 26.23% of all sequences. It has been reported that the number of phages in the oral cavity is estimated to be 35 times more than that of bacteria [49], indicating that the high percentage of phages predicted by PPR-Meta may be reliable. Moreover, the high percentages of phages and plasmids means that HGT may be more frequent. Since the outer end of the digestive tract is closer to the changing external environment, HGT seems to be a way for microbial communities at the outer end of the digestive tract to adapt to the external environment.

**Figure 3. Percentages of phages, chromosomes and plasmids in the human digestive tract.** PPR-Meta was used to predict the sequences of phages, chromosomes and plasmids in metagenomic assemblies, including samples from the gut, throat and oral cavity. The sequence percentages of phages, chromosomes and plasmids were calculated.

## Usage of PPR-Meta

PPR-Meta takes the sequence file in fasta format as input and outputs a tabular file. The output file contains three scores between 0 and 1 that reflect the likelihood of obtaining phages, chromosomes and plasmids for each sequence. By default, the final prediction is the category with the highest score. To meet users' actual requirements, PPR-Meta is designed with the option to adjust the threshold to filter out the uncertain predictions so that the remaining predictions may be more reliable. Given a threshold, a sequence with a highest score lower than the threshold will be labelled as "uncertain". In this way, the outputs of PPR-Meta contain six categories: phage, uncertain phage, chromosome, uncertain chromosome, plasmid and uncertain plasmid. We evaluated the uncertain prediction rate, accuracy, AUC, TPR and FPR under different thresholds, and the results are shown in Additional File 3, Figure S5. In general, with a higher threshold, the accuracy, AUC, and TPR as well as the uncertain prediction rate will be higher, while the FPR will be lower.

PPR-Meta is user friendly, and the program has been optimized in a virtual

machine so that users can directly run PPR-Meta without installing any dependency package. We also provided a short video guide to show how to install the virtual machine. If users are analysing large-scale data, running the executable file on the physical host is more suitable. In this way, when the GPU is available, PPR-Meta will run on the GPU automatically to speed up the program. The memory requirements are dependent on the data size. We recommend at least 4~6 GB of available memory when running the virtual machine or at least 16 GB when handling large-scale data on the physical host. We tested the running time of PPR-Meta using 90,000 sequences from 100 to 10k bp and found that this tool can handle all sequences in approximately 45 minutes on a machine with the following configuration: CPU: Intel Core i7 6700; GPU: NVIDIA GTX1060; and Memory: 64G, DDR4.

## Discussion and conclusions

In this paper, we proposed an ab initio method, PPR-Meta, to identify both phages and plasmids from metagenomic sequences. PPR-Meta employs a novel strategy to improve the MGE identification performance and avoids performing similarity searches to make judgments. Similarity search-based tools, such as VirSorter, provide good results for long sequences. However, such methods do not work effectively for short fragments due to the insufficient number of genes for the statistical analysis. Compared with other reference-free tools, PPR-Meta employs a more detailed method of characterizing DNA

sequences. We use a BOH matrix, which is beneficial to non-coding regions, and a COH matrix, which is beneficial to coding regions, to represent sequences. In contrast, traditional k-mer methods do not consider coding or non-coding regions. When the sequence is short, k-mer frequencies will be noisy. On the other hand, k-mer-based methods may also be more sensitive to the sequence length than the BiPathCNN method in the current work. The distribution of k-mer frequencies may be different between long sequences and short sequences, and the variance of the k-mer frequencies for short sequences may be much higher. Thus, the k-mer-based classifier constructed using short sequence data may not by applicable for long sequences, and vice versa. Among the k-mer-based tools, cBar was trained with complete genomes and PlasFlow was trained on 10k bp fragments, which might make them hard to adapt to metagenomic data with a wide range of lengths. Differently, our BiPathCNN directly extracts sequence features from the raw data represented by the one-hot matrix and may be less sensitive to the sequence length. Tests of each BiPathCNN on test datasets from different groups (Additional File 3, Figure S2) also showed that although the overall accuracy was slightly reduced when testing each group using a non-corresponding BiPathCNN from the other groups, the decrease was not obvious, indicating that our approach is not quite sensitive to the sequence length. Another shortcoming of k-mer-based tools may be that mapping sequences of different length for k-mer feature vectors with the same dimension will also lose some information. PPR-Meta takes all

bases and codons as inputs in the neural network, thereby exploiting all information in the fragments. In the design of the algorithm, we employed a deep learning network as the classifier. Deep learning has achieved great success in many fields, such as lncRNA identification [50] and the prediction of sequence specificities of nucleic acid binding proteins [51]. In the construction of PPR-Meta, we designed the BiPathCNN, which contains a “base path” and “codon path” to handle the BOH matrix and COH matrix, respectively. Testing showed that the performance of the CNN with double paths was better than that with single path.

Furthermore, we were surprised to find that PPR-Meta’s output scores were able to describe the interaction between phages or plasmids and their hosts. Specifically, the difference between the phage score and chromosome score reveals the lifestyle of the phages (virulent or temperate), while the difference between the plasmid score and chromosome score reveals the transmissibility of the plasmids (transmissible or non-transmissible). We collected both phage genomes with lifestyle annotations from McNair et al. [8] and plasmid genomes with transmissibility annotations from Shintani et al. [52] and then extracted artificial contigs. PPR-Meta was run on all the contigs and the correctly predicted contigs were retained. From the results, two normalized statistics were constructed:

$$life\_score=(phage\_score-chromosome\_score)/phage\_score$$

and

$$trans\_score = (plasmid\_score - chromosome\_score)/plasmid\_score$$

The receiver operating characteristic curve (ROC) showed that life_score could distinguish the lifestyle of phages while trans_score could distinguish the transmissibility of plasmids with AUC values higher than 0.5 (shown in Figure 4). Specifically, temperate phages tend to have lower life_score values and non-transmissible plasmids tend to have lower trans_score values. This phenomenon may be due to the genome amelioration of foreign DNA to the host. For example, research has shown that the comparison of the trinucleotide composition between a plasmid and bacterial chromosome can be used to predict the host range of plasmids [53]. Since temperate phages and non-transmissible plasmids experience longer residence times within the host cell, they may adjust the sequence pattern toward the host. Thus, the sequence pattern between temperate phages (or non-transmissible plasmids) and host chromosomes may be more similar than that between virulent phages (or transmissible plasmids) and host chromosomes, thereby resulting in a lower life_score (or trans_score) value. Although tools that can classify phage lifestyle and plasmid transmissibility on metagenomes are lacking as far as we know, the phenomena mentioned above may provide insights into the classification strategy for future studies.

**Figure 4. ROC curve of classifying phage lifestyle and plasmid transmissibility.** (a) Classify virulent phages and temperate phages using

life_score. In order of sequence length, the AUC is 0.63, 0.69, 0.71 and 0.76. (b) Classify transmissible plasmid and non-transmissible plasmid using trans_score. In order of sequence length, the AUC is 0.58, 0.55, 0.60 and 0.62.

In general, bacteria contain genomic islands, regions of horizontal origin on chromosomes [54]. The formation mechanisms of some genomic islands are caused by phages or plasmids [55]. To see how PPR-Meta and related tools perform on DNA fragments from these regions, we collected genomic island sequences from the Islander database [56]. Upon testing on artificial contigs between 100 bp and 10k bp extracted from these genomic islands, the results showed that PPR-Meta could identify 65.25% of them as foreign DNA (either phage or plasmid), while VirFinder, VirSorter, PlasFlow and cBar could identify 20.46%, 6.72%, 53.11% and 51.62% of them, respectively, indicating that PPR-Meta has a better ability to recognize sequences from regions of horizontal origin on bacterial chromosomes.

PPR-Meta also has some limitations. In addition to prokaryote chromosomes, plasmids and phages, other organisms of low-abundance may exist in the microbial community, such as fungi and protozoan. Such organisms are not included in the training set of PPR-Meta and may have interfered with the judgment of PPR-Meta. To increase the suitability of PPR-Meta for real scenes, we will retrain PPR-Meta regularly with expanded datasets. More organisms as well as the newly sequenced genomes will be added to the

dataset so that PPR-Meta will be more powerful and reliable. In addition, due to sequence exchanges among phages, plasmids and chromosomes, there are a few chimeric sequences from two sources (e.g., a prophage and chromosome chimera). PPR-Meta cannot perform detailed judgments about these chimeras, and we are considering how to further identify such sequences. However, because these chimeras do not exist at a large scale, we believe that the presence of chimeras will not have a significant impact on the application of PPR-Meta.

In conclusion, the performance of PPR-Meta has shown much better than that of currently available similar tools, while none of these tools can function as PPR-Meta does. It is thus expected that the PPR-Meta tool would meet the demand of metagenomics analysis when considered the microbial community tangled with phages and plasmids, and certainly qualifies as a powerful tool for the research community.

## Methods

PPR-Meta was trained and tested using artificial contigs. We downloaded the accession list of prokaryote chromosomes, prokaryote plasmids and phages from the NCBI genome database, and the corresponding genomes were downloaded according to the list. To ensure the quality of the data, we only used the complete genomic molecule with the RefSeq accession prefix. Since chromosomes may contain prophages, we used ProphET to extract the

prophage regions of all chromosomes. ProphET requires a genome sequence file (fasta format) and genome annotation file (gff format) as inputs. A few genomes do not contain the annotation information, and these genomes were removed from the dataset. We then used MetaSim to generate four groups of artificial contigs with different lengths as mentioned in the main text. To generate artificial contigs with no error for both training and test set, we used the “exact” preset to return fragments exactly matching reference sequences. In each group, the “DNA Clone Size Distribution Type” was set to “Uniform”. To generate artificial contigs modified with sequencing errors, we used the “Sanger” preset, which allowed users to modify sequences according to their settings. Note that as we were not going to generate sequences with technology-specific errors, the following settings do not reflect the real situation of the Sanger technology. For the generation of sequences with 1% base substitutions, the “Read Length Distribution Type” was set to “Uniform”, and the “Mate Pair Probability” was set to 0; both the “Error Rate at Read Star” and the “Error Rate at End of Read” were set to 0.01; and both the “Insertion Error Rate” and “Deletion Error Rate” were set to 0. For the generation of sequences with 1% base insertions or deletions, most settings were the same as mentioned above, except that both the “Insertion Error Rate” and “Deletion Error Rate” were set to 0.5. In general, the performance of the algorithm will be better as the amount of training data increases. Considering the memory size, running time and accuracy, a total of 2,700,000 artificial contigs were generated to train PPR-

Meta. The number of training contigs of each phage, chromosome and plasmid is 300,000 from Group A to C.

We also used real metagenomic data to evaluate PPR-Meta and the related tools. We used SPAdes to assemble the raw reads, as we mentioned in the main text. The phage metagenomic data of the bovine rumen were downloaded from MG-RAST, and we used the command “spades.py --meta -1 file1.fastq -2 file2.fastq -o out_folder” to assemble the pair-end raw reads. In the assembly, the contig number, N50, average length, maximum length and minimum length were 107529, 288, 312.06, 75508 and 56, respectively. To download the 20 samples of the healthy human gut, we used the command “prefetch SRRaccession” from the SRA Toolkit. All samples were downloaded as “.sra” files. We then used the command “fastq-dump --split-files accession.sra” from the SRA Toolkit to convert the sra file into two pair-end fastq files and used SPAdes with the same settings as mentioned above to assemble the raw reads. The information about the contig number, N50, average length, maximum length and minimum length is provided in Additional File 1.

The artificial contigs are stored at http://cqb.pku.edu.cn/ZhuLab/PPR_Meta/data/.

**Availability of supporting source code and requirements**

**Project name:** PPR-Meta.

**Project home page:** http://cqb.pku.edu.cn/ZhuLab/PPR_Meta or

https://github.com/zhenchengfang/PPR-Meta.

**Operating system:** The code of PPR-Meta was written on Linux. We optimized the program in a virtual machine thus PPR-Meta is platform independent.

**Programming language:** python, matlab

**Other requirements:** no other requirements are needed if running in the virtual machine. If not, Python 2.7.12, TensorFlow 1.4.1, Keras 2.0.8 and MATLAB Component Runtime 2018a (for free) are needed. MATLAB is not necessary.

**License:** GPL-3.0.

**RRID:** SCR_016915

## Availability of supporting data

The artificial contigs, related scripts and original results are available at http://cqb.pku.edu.cn/ZhuLab/PPR_Meta/data/. All the other data are available at corresponding references mentioned in the main text.

## Additional file

Additional file 1: accession list of the data used to train and test PPR-Meta.

Additional file 2: prophage coordinate predicted by ProphET.

Additional file 3: Figure S1 to Figure S5.

## List of abbreviations

MGEs: mobile genetic elements

HGT: horizontal gene transfer

WGS: Whole Genome Sequencing

SMO: sequential minimal optimization

BiPathCNN: Bi-path Convolutional Neural Network

BOH: base one-hot matrix

COH: codon one-hot matrix

TPR: true positive rate

FPR: false positive rate

AUC: area under curve

ROC: receiver operating characteristic

HMP: Human Microbiome Project

**Ethics approved and consent to participate**

Not applicable

**Consent for publication**

Not applicable

**Competing interests**

The authors declare that they have no competing interests

**Funding**

This work was supported by the National Key Research and Development Program of China (2017YFC1200205), the National Natural Science Foundation of China (31671366), and the Special Research Project of 'Clinical Medicine + X' by PKU.

## Authors' contributions

HQZ and ZCF proposed and designed the study. ZCF, JT and SFW constructed the data sets, and wrote and optimized the code. ML, CMX and ZJX tested the program. ZCF and HQZ wrote and revised the manuscript and all authors proofread and improved the manuscript.

## Acknowledgements

We thank Dr. Cheng Yang, Dr. Longshu Yang, Dr. Xiaoqing Jiang, Li Qu of Peking University for their helpful discussions. Part of the analysis was performed on the High Performance Computing Platform of the Center for Life Science of Peking University.

## References

[1] Frost LS, Leplae R, Summers AO, Toussaint A. Mobile genetic elements: the agents of open source evolution. *Nat. Rev. Microbiol.*, 2005;3(9): 722.

[2] Brown-Jaque M, Calero-Cáceres W, Muniesa M. Transfer of antibiotic-resistance genes via phage-related mobile elements. *Plasmid*, 2015;*79*: 1-7.

[3] Paul JH, Sullivan MB. Marine phage genomics: what have we learned?. *Curr. Opin. Biotechnol.*, 2005:16(3): 299-307.

[4] Sobecky PA, Hazen TH. Horizontal gene transfer and mobile genetic elements in marine systems. In *Horizontal Gene Transfer*, 2009; 435-453, Humana Press.

[5] Hayes S, Mahony J, Nauta A, van Sinderen D. Metagenomic approaches to assess bacteriophages in various environmental niches. *Viruses*, 2017; 9(6): 127.

[6] Li LL., Norman A, Hansen LH, Sørensen SJ. Metamobilomics-expanding our knowledge on the pool of plasmid encoded traits in natural environments using high-throughput sequencing. *Clin. Microbiol. Infect.*, 2012: 18, 5-7.

[7] Rozov R, Brown Kav A, Bogumil D, Shterzer N, Halperin E, Mizrahi I, Shamir R. Recycler: an algorithm for detecting plasmids from de novo assembly graphs. *Bioinformatics*, 2017; 33(4): 475-482.

[8] McNair K, Bailey BA, Edwards RA. PHACTS, a computational approach to classifying the lifestyle of phages. *Bioinformatics*, 2012; 28(5): 614-618.

[9] Lima-Mendez G, Van Helden J, Toussaint A, Leplae R. Prophinder: a computational tool for prophage prediction in prokaryotic genomes. *Bioinformatics*, 2008; 24(6): 863-865.

[10] Fouts DE. Phage_Finder: automated identification and classification of prophage regions in complete bacterial genome sequences. *Nucleic Acids Res.*, 2006; 34(20): 5839-5851.

[11] Akhter S, Aziz RK, Edwards RA. PhiSpy: a novel algorithm for finding prophages in bacterial genomes that combines similarity-and composition-based strategies. *Nucleic Acids Res.*, 2012; 40(16): e126-e126.

[12] Zhou Y, Liang Y, Lynch KH, Dennis JJ, Wishart DS. PHAST: a fast phage search tool. *Nucleic Acids Res.*, 2011; 39(suppl_2): W347-W352.

[13] Arndt D, Grant JR, Marcu A, Sajed T, Pon A, Liang Y, Wishart DS. PHASTER: a better, faster version of the PHAST phage search tool. *Nucleic Acids Res.*, 2016; 44(W1): W16-W21.

[14] Roux S, Enault F, Hurwitz B L, Sullivan MB. VirSorter: mining viral signal from microbial genomic data. *PeerJ*, 2015; 3: e985.

[15] Reis-Cunha JL., Bartholomeu DC, Earl AM, Birren BW, Cerqueira GC. ProphET, Prophage Estimation Tool: a standalone prophage sequence prediction tool with self-updating reference database. 2017; *bioRxiv*, 176750.

[16] Liu Y, Guo J, Hu G, Zhu H. Gene prediction in metagenomic fragments based on the SVM algorithm. *BMC Bioinformatics*, 2013; 14(5): S12.

[17] Mirzaei MK, Maurice CF. Ménage à trois in the human gut: interactions between host, bacteria and phages. *Nat. Rev. Microbiol.*, 2017; 15(7), 397.

[18] Amgarten DE, Braga LPP, Da Silva AM, Setubal JC. MARVEL, a Tool for Prediction of Bacteriophage Sequences in Metagenomic Bins. *Front. Genet.*, 2018; 9: 304.

[19] Ren J, Ahlgren NA, Lu YY, Fuhrman JA, Sun F. VirFinder: a novel k-mer based tool for identifying viral sequences from assembled metagenomic data.

*Microbiome*, 2017; 5(1): 69.

[20] Carattoli A, Zankari E, García-Fernández A, Larsen MV, Lund O, Villa L, Aarestrup FM, Hasman H. In silico detection and typing of plasmids using PlasmidFinder and plasmid multilocus sequence typing. *Antimicrob. Agents Chemother.*, 2014; 58(7): 3895-3903.

[21] Lanza VF, de Toro M, Garcillán-Barcia MP, Mora A, Blanco J, Coque TM, de la Cruz F. Plasmid flux in Escherichia coli ST131 sublineages, analyzed by plasmid constellation network (PLACNET), a new method for plasmid reconstruction from whole genome sequences. *PLoS Genet.*, 2014; 10(12): e1004766.

[22] Roosaare M, Puustusmaa M, Möls M, Vaher M, Remm M. PlasmidSeeker: identification of known plasmids from bacterial whole genome sequencing reads. 2018; *PeerJ*, 6: e4588.

[23] Arredondo-Alonso S, Rogers MRC, Braat JC, Verschuuren TD, Top J, Corander J, Willems RJL, Schürch AC. mlplasmids: a user-friendly tool to predict plasmid- and chromosome-derived sequences for single species. *Microb. Genom.*, 2018; 4(11).

[24] Zhou F, Xu Y. cBar: a computer program to distinguish plasmid-derived from chromosome-derived sequence fragments in metagenomics data. *Bioinformatics*, 2010; 26(16): 2051-2052.

[25] Krawczyk PS, Lipinski L, Dziembowski, A. PlasFlow: predicting plasmid sequences in metagenomic data using genome signatures. *Nucleic Acids Res.*,

2018; 46(6): e35-e35.

[26] The NCBI genome database. ftp://ftp.ncbi.nlm.nih.gov/genomes/GENOME_REPORTS/. Accessed 12 July 2018.

[27] Casjens S. Prophages and bacterial genomics: what have we learned so far?. *Mol. Microbiol.*, 2003; 49(2): 277-300.

[28] Richter DC, Ott F, Auch AF, Schmid R, Huson DH. MetaSim—a sequencing simulator for genomics and metagenomics. *PLoS One*, 2008; 3(10): e3373.

[29] Ross EM, Petrovski S, Moate PJ, Hayes BJ. Metagenomics of rumen bacteriophage from thirteen lactating dairy cattle. BMC Microbiol., 2013; 13(1): 242.

[30] Meyer F, Paarmann D, D'Souza M, Olson R, Glass EM, Kubal M, Paczian T, Rodriguez A, Stevens R, Wilke A, Wilkening J. The metagenomics RAST server–a public resource for the automatic phylogenetic and functional analysis of metagenomes. *BMC Bioinformatics.* 2008; 9(1): 386.

[31] Bankevich A, Nurk S, Antipov D, Gurevich AA, Dvorkin M, Kulikov AS, Lesin VM, Nikolenko SI, Pham S, Prjibelski AD, Pyshkin AV. SPAdes: a new genome assembly algorithm and its applications to single-cell sequencing. *J. Comput. Biol.*, 2012; 19(5): 455-477.

[32] Kav AB, Sasson G, Jami E, Doron-Faigenboim A, Benhar I, Mizrahi I. Insights into the bovine rumen plasmidome. *Proc. Natl. Acad. Sci. U S A*, 2012; 109(14): 5452-5457.

[33] Qin J, et al. A metagenome-wide association study of gut microbiota in type 2 diabetes. *Nature*, 2012; 490(7418): 55-60.

[34] NCBI Short Read Archive. https://www.ncbi.nlm.nih.gov/sra/. Accessed 12 July 2018.

[35] Galiez C, Siebert M, Enault F, Vincent J, Söding J. WIsH: who is the host? Predicting prokaryotic hosts from metagenomic phage contigs. *Bioinformatics*, 2017; 33(19): 3113-3114.

[36] Braud C, Denis P. (2015). Comparing word representations for implicit discourse relation classification. *In Proceedings of the 2015 Conference on Empirical Methods in Natural Language Processing*, (2015); 2201-2211.

[37] LeCun Y, Bottou L, Bengio Y, Haffner P. Gradient-based learning applied to document recognition. *Proceedings of the IEEE*, 1998; 86(11): 2278-2324.

[38] Simonyan K, Zisserman A. Very deep convolutional networks for large-scale image recognition. *arXiv preprint arXiv*, 2014: 1409.1556.

[39] Edwards RA, McNair K, Faust K, Raes J, Dutilh BE. Computational approaches to predict bacteriophage–host relationships. *FEMS Microbiol. Rev.*, 2015; 40(2): 258-272.

[40] Rho M, Tang H, Ye Y. FragGeneScan: predicting genes in short and error-prone reads. *Nucleic Acids Res.*, 2010; 38(20): e191-e191.

[41] Wick RR, Judd LM, Holt KE. Comparison of Oxford Nanopore Basecalling Tools. *URL https://doi.org/10.5281/zenodo.1082696*, 2017.

[42] Aziz, Ramy, Akhter, Sajia, Schmieder, Robert, Edwards, Robert A.

PhAnToMe. Phage annotation, tools, and Methods. http://www.phantome.org/Downloads/Prophages/PhiSpy/. Accessed 9 January 2019.

[43] Hurwitz BL, Ponsero A, Thornton J, U'Ren JM. Phage Hunters: computational strategies for finding phages in large-scale 'omics datasets. *Virus Res.*, 2018; 244(15): 110-115.

[44] DeSantis TZ, Hugenholtz P, Larsen N, Rojas M, Brodie EL, Keller K, Huber T, Dalevi D, Hu P, Andersen GL. Greengenes, a chimera-checked 16S rRNA gene database and workbench compatible with ARB. *Appl. Environ. Microbiol.*, 2006; 72(7): 5069-5072.

[45] Smillie C, Garcillán-Barcia MP, Francia MV, Rocha EP, de la Cruz F. Mobility of plasmids. *Microbiol. Mol. Biol. R.*, 2010; 74(3): 434-452.

[46] Warwick-Dugdale J, Solonenko N, Moore K, Chittick L, Gregory AC, Allen MJ, Sullivan MB, Temperton B. Long-read metagenomics reveals cryptic and abundant marine viruses. *bioRxiv*, 2018; 345041.

[47] Turnbaugh PJ, Ley RE, Hamady M, Fraser-Liggett CM, Knight R, Gordon JI. The human microbiome project. *Nature*, 2007; 449(7164): 804-810.

[48] Minot S, Sinha R, Chen J, Li H, Keilbaugh SA, Wu GD, Lewis JD, Bushman FD. The human gut virome: inter-individual variation and dynamic response to diet. *Genome Res.*, 2011; 21:1616–25.

[49] Edlund A, Santiago-Rodriguez TM, Boehm TK, Pride DT. Bacteriophage and their potential roles in the human oral cavity. *J. Oral Microbiol.*, 2015; 7(1):

27423.

[50] Yang C, Yang L, Zhou M, Xie H, Zhang C, Wang MD, Zhu H. LncADeep: An ab initio lncRNA identification and functional annotation tool based on deep learning. *Bioinformatics*, 2018; 34(22): 3825-3843.

[51] Alipanahi B, Delong A, Weirauch MT, Frey BJ. Predicting the sequence specificities of DNA-and RNA-binding proteins by deep learning. *Nat. Biotechnol.*, 2015; 33(8): 831-838.

[52] Shintani M, Sanchez ZK, Kimbara K. Genomics of microbial plasmids: classification and identification based on replication and transfer systems and host taxonomy. *Front. Microbiol.*, 2015; 6:242.

[53] Suzuki H, Yano H, Brown CJ, Top EM. Predicting plasmid promiscuity based on genomic signature. *J. Bacteriol.*, 2010; 192(22): 6045–6055.

[54] Bertelli C, Laird MR, Williams KP, Simon Fraser University Research Computing Group, Lau BY, Hoad G, Winsor GL, Brinkman FSL. IslandViewer 4: expanded prediction of genomic islands for larger-scale datasets. *Nucleic Acids Res.*, 2017; 45(W1): W30-W35.

[55] Juhas M, Van Der Meer JR, Gaillard M, Harding RM, Hood DW, Crook DW. Genomic islands: tools of bacterial horizontal gene transfer and evolution. *FEMS Microbiol. Rev.*, 2009; 33(2):376-93.

[56] Hudson CM, Lau BY, Williams KP. Islander: a database of precisely mapped genomic islands in tRNA and tmRNA genes. *Nucleic Acids Res.*, 2015; 43: D48-53.

## Table

**Table 1**. Evaluation of the performance of PPR-Meta and comparison of the performance of PPR-Meta and related tools.

| Group | Tool | Evaluation on phage | | | Evaluation on plasmid | | |
|---|---|---|---|---|---|---|---|
| | | *TPR*(%) | *FPR*(%) | *AUC*(%) | *TPR*(%) | *FPR*(%) | *AUC*(%) |
| Group A 100-400 bp | **PPR-Meta** | **84.96** | 18.01 | **91.82** | 59.91 | **14.14** | **83.05** |
| | VirFinder | 73.77 | 25.45 | 81.30 | NA | NA | NA |
| | VirSorter | 0.00 | **0.00** | 50.00 | NA | NA | NA |
| | PlasFlow | NA | NA | NA | **71.89** | 62.59 | 56.30 |
| | cBar | NA | NA | NA | 52.68 | 46.07 | 53.31 |
| Group B 400-800 bp | **PPR-Meta** | **90.75** | 8.37 | **97.21** | **74.56** | **13.37** | **89.64** |
| | VirFinder | 79.27 | 18.15 | 88.64 | NA | NA | NA |
| | VirSorter | 0.05 | **0.002** | 50.02 | NA | NA | NA |
| | PlasFlow | NA | NA | NA | 72.61 | 55.01 | 62.50 |
| | cBar | NA | NA | NA | 55.00 | 43.59 | 55.70 |
| Group C 800-1200 bp | **PPR-Meta** | **95.24** | 7.75 | **98.54** | **78.09** | **10.95** | **91.84** |
| | VirFinder | 81.91 | 15.63 | 91.09 | NA | NA | NA |
| | VirSorter | 0.17 | **0.002** | 50.09 | NA | NA | NA |
| | PlasFlow | NA | NA | NA | 75.89 | 50.55 | 68.01 |
| | cBar | NA | NA | NA | 55.54 | 41.87 | 56.84 |
| Group D 5000-10k bp | **PPR-Meta** | **99.20** | 3.25 | **99.77** | 87.53 | **6.45** | **96.02** |
| | VirFinder | 89.26 | 8.13 | 97.12 | NA | NA | NA |
| | VirSorter | 66.80 | **2.48** | 82.66 | NA | NA | NA |
| | PlasFlow | NA | NA | NA | **88.50** | 30.22 | 88.42 |
| | cBar | NA | NA | NA | 63.79 | 32.61 | 65.59 |

NA: not applicable.

**Table 2**. Performance comparison among BiPathCNN, the base path-only CNN and codon path-only CNN.

| Group | Tool | Evaluation on phage | | | Evaluation on plasmid | | |
|---|---|---|---|---|---|---|---|
| | | *TPR*(%) | *FPR*(%) | *AUC*(%) | *TPR*(%) | *FPR*(%) | *AUC*(%) |
| Group A 100-400 bp | **BiPathCNN** | 84.96 | 18.01 | 91.82 | 59.91 | 14.14 | **83.05** |
| | Base path-only | 81.86 | 24.58 | 87.50 | 56.96 | 17.60 | 78.50 |
| | Codon path-only | 86.84 | 20.47 | **91.85** | 62.15 | 16.65 | 82.26 |
| Group B 400-800 bp | **BiPathCNN** | 90.75 | 8.37 | **97.21** | 74.56 | 13.37 | **89.64** |
| | Base path-only | 88.76 | 17.46 | 93.87 | 72.37 | 18.86 | 85.57 |
| | Codon path-only | 84.95 | 5.93 | 96.57 | 82.98 | 23.10 | 88.32 |
| Group C 800-1200 bp | **BiPathCNN** | 95.24 | 7.75 | **98.54** | 78.09 | 10.95 | **91.84** |
| | Base path-only | 92.09 | 17.71 | 95.47 | 73.31 | 15.12 | 88.02 |
| | Codon path-only | 94.60 | 12.44 | 97.55 | 73.17 | 12.41 | 89.22 |

**Table 3**. Identification performance of each tool with 1% base substitutions.

| Group | Tool | Evaluation on phage | | | Evaluation on plasmid | | |
|---|---|---|---|---|---|---|---|
| | | *TPR*(%) | *FPR*(%) | *AUC*(%) | *TPR*(%) | *FPR*(%) | *AUC*(%) |
| Group A 100-400 bp | **PPR-Meta** | **84.42** | 17.99 | **91.57** | 61.19 | **15.27** | **82.76** |
| | VirFinder | 72.55 | 26.20 | 80.42 | NA | NA | NA |
| | VirSorter | 0.00 | **0.00** | 50.00 | NA | NA | NA |
| | PlasFlow | NA | NA | NA | **71.72** | 62.82 | 55.86 |
| | cBar | NA | NA | NA | 52.98 | 46.18 | 53.40 |
| Group B 400-800 bp | **PPR-Meta** | **90.05** | 8.48 | **97.02** | **75.07** | **14.03** | **89.39** |
| | VirFinder | 78.50 | 18.75 | 87.95 | NA | NA | NA |
| | VirSorter | 0.02 | **0.00** | 50.01 | NA | NA | NA |
| | PlasFlow | NA | NA | NA | 72.31 | 55.61 | 61.87 |
| | cBar | NA | NA | NA | 54.83 | 44.63 | 55.10 |
| Group C 800-1200 bp | **PPR-Meta** | **94.54** | 7.72 | **98.33** | **79.03** | **11.99** | **91.59** |
| | VirFinder | 81.29 | 15.92 | 90.68 | NA | NA | NA |
| | VirSorter | 0.21 | **0.00** | 50.11 | NA | NA | NA |
| | PlasFlow | NA | NA | NA | 75.24 | 50.91 | 67.15 |
| | cBar | NA | NA | NA | 56.57 | 42.85 | 56.86 |
| Group D 5000-10k bp | **PPR-Meta** | **98.97** | 3.15 | **99.75** | 87.65 | **7.20** | **95.83** |
| | VirFinder | 88.90 | 8.19 | 97.01 | NA | NA | NA |
| | VirSorter | 60.30 | **1.13** | 79.80 | NA | NA | NA |
| | PlasFlow | NA | NA | NA | **88.57** | 31.42 | 87.86 |
| | cBar | NA | NA | NA | 64.31 | 34.63 | 64.84 |

NA: not applicable.

**Table 4**. Identification performance of each tool with 1% base insertions or deletions.

| Group | Tool | Evaluation on phage | | | Evaluation on plasmid | | |
|---|---|---|---|---|---|---|---|
| | | *TPR*(%) | *FPR*(%) | *AUC*(%) | *TPR*(%) | *FPR*(%) | *AUC*(%) |
| Group A 100-400 bp | **PPR-Meta** | **80.26** | 18.64 | **89.28** | 65.29 | **19.93** | **81.62** |
| | VirFinder | 72.62 | 25.96 | 80.57 | NA | NA | NA |
| | VirSorter | 0.00 | **0.00** | 50.00 | NA | NA | NA |
| | PlasFlow | NA | NA | NA | **71.12** | 62.83 | 55.81 |
| | cBar | NA | NA | NA | 53.63 | 46.43 | 53.60 |
| Group B 400-800 bp | **PPR-Meta** | **85.50** | 9.69 | **95.26** | **77.44** | **17.57** | **88.48** |
| | VirFinder | 79.00 | 18.76 | 88.28 | NA | NA | NA |
| | VirSorter | 0.24 | **0.00** | 50.12 | NA | NA | NA |
| | PlasFlow | NA | NA | NA | 72.74 | 55.44 | 62.31 |
| | cBar | NA | NA | NA | 55.38 | 45.22 | 55.08 |
| Group C 800-1200 bp | **PPR-Meta** | **92.99** | 9.12 | **97.54** | **79.74** | **14.29** | **90.80** |
| | VirFinder | 81.98 | 16.00 | 90.93 | NA | NA | NA |
| | VirSorter | 2.38 | **0.02** | 51.18 | NA | NA | NA |
| | PlasFlow | NA | NA | NA | 75.23 | 51.25 | 66.75 |
| | cBar | NA | NA | NA | 56.74 | 43.45 | 56.64 |
| Group D 5000-10k bp | **PPR-Meta** | **98.90** | 3.51 | **99.73** | **89.23** | **8.48** | **95.84** |
| | VirFinder | 88.93 | 8.40 | 96.98 | NA | NA | NA |
| | VirSorter | 47.25 | **0.25** | 73.51 | NA | NA | NA |
| | PlasFlow | NA | NA | NA | 88.74 | 31.70 | 88.08 |
| | cBar | NA | NA | NA | 64.62 | 35.40 | 64.61 |

NA: not applicable.

**Table 5**. Recognition rate of prophages

| Group | Tool | Recognition rate (%) |
|---|---|---|
| Group A 100-400 bp | **PPR-Meta** | **60.79** |
| | VirFinder | 43.46 |
| | VirSorter | 0.00 |
| Group B 400-800 bp | **PPR-Meta** | **60.59** |
| | VirFinder | 40.77 |
| | VirSorter | 0.00 |
| Group C 800-1200 bp | **PPR-Meta** | **68.09** |
| | VirFinder | 41.94 |
| | VirSorter | 0.05 |
| Group D 5000-10k bp | **PPR-Meta** | **75.58** |
| | VirFinder | 48.62 |

| | | |
|---|---|---|
| | VirSorter | 37.75 |

1

Figure 4

Click here to access/download;Figure;figure_4.jpg

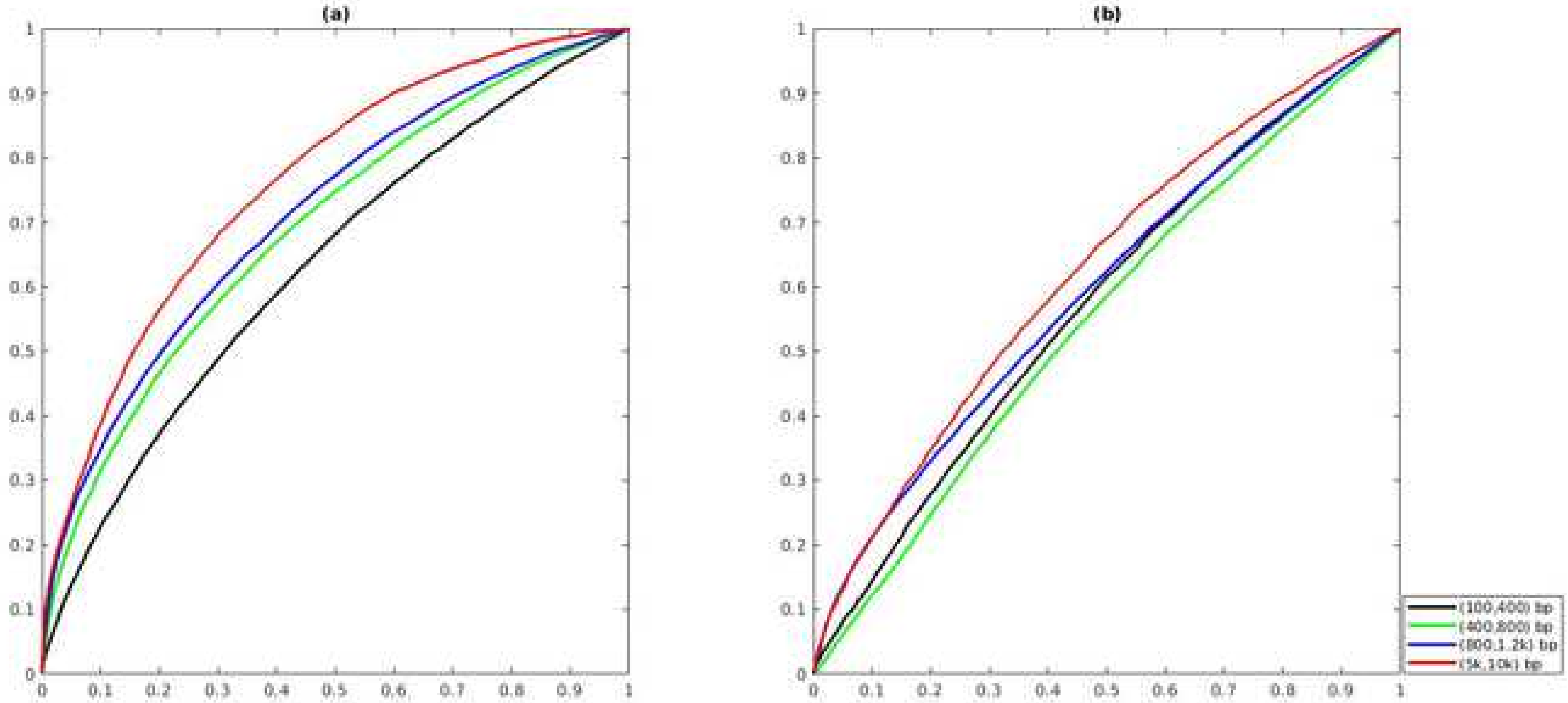

Figure 3

Click here to access/download;Figure;figure_3.pdf

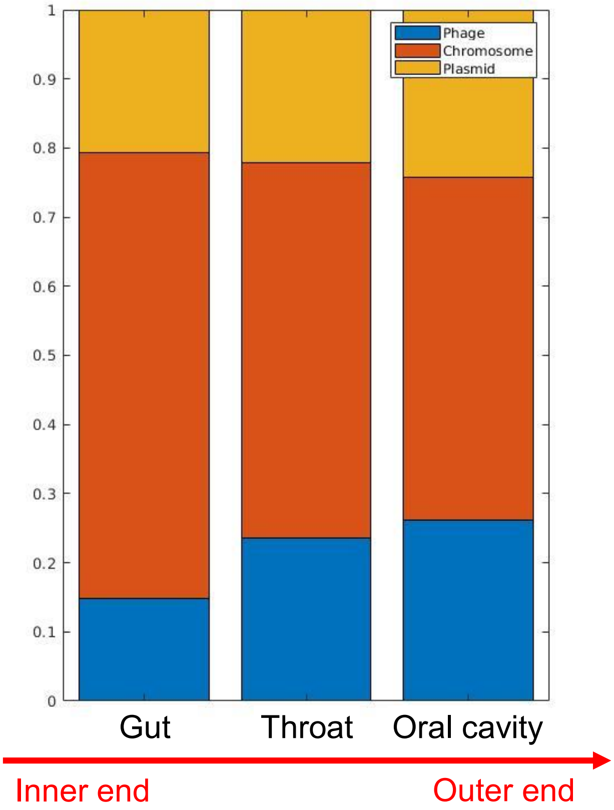

Figure 2

Click here to access/download;Figure;figure_2.pdf

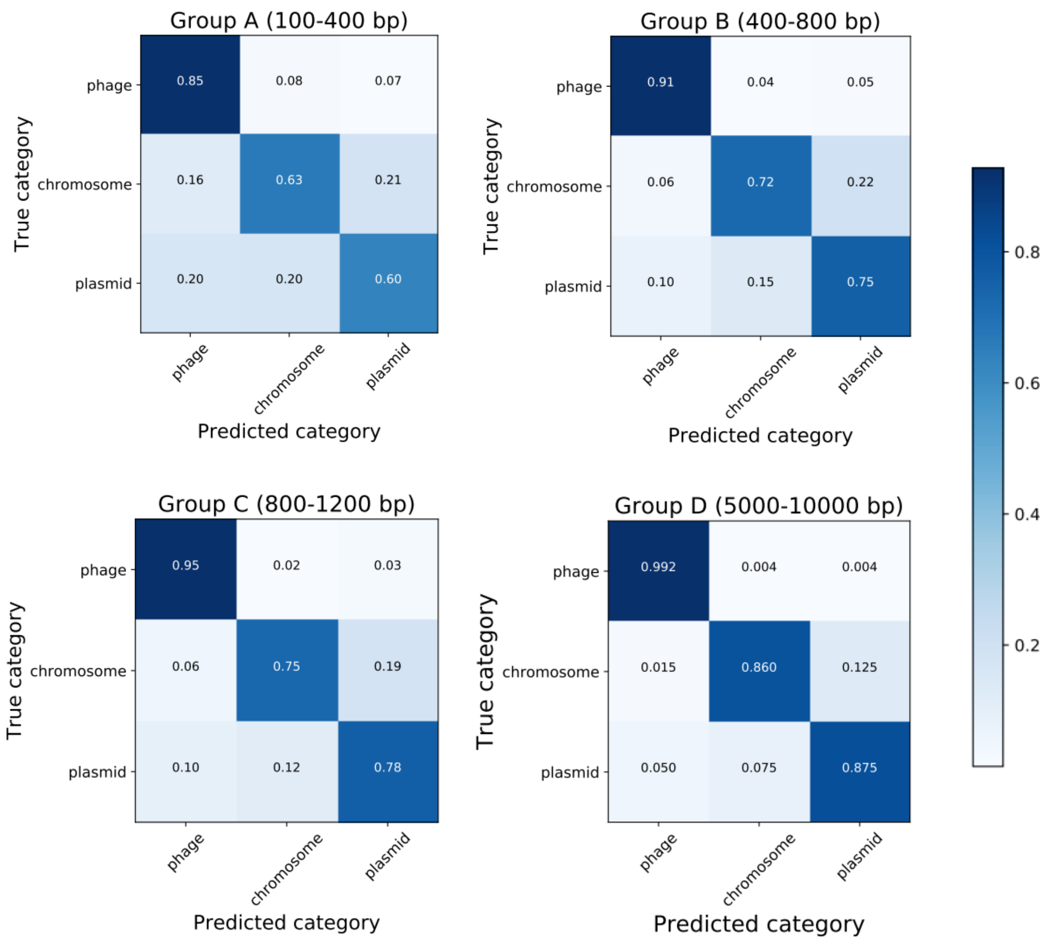

Figure 1

Click here to access/download;Figure;figure_1.pdf

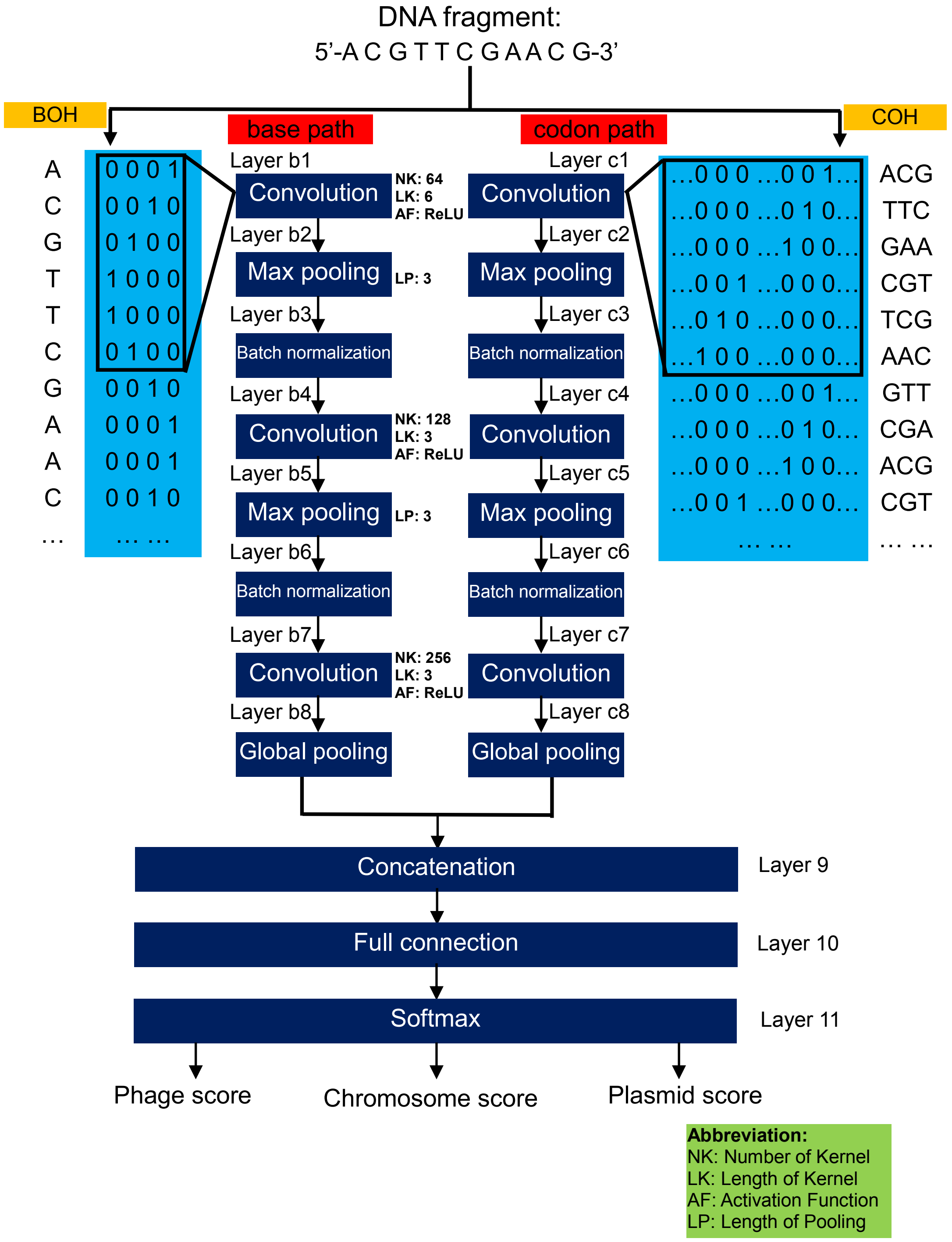

Additional File 2

Click here to access/download
**Supplementary Material**
Additional file 2.xlsx

Additional File 3

Click here to access/download
**Supplementary Material**
Additional file 3.docx

Additional File 1

Click here to access/download
**Supplementary Material**
Additional file 1.xlsx

Cover letter

Click here to access/download;Personal Cover;Cover_Letter_revision1.docx

# Cover Letter

Dear Editor,

Thank you very much for your previous E-mail on December 26, 2018 regarding our manuscript "PPR-Meta: a tool for identifying phages and plasmids from metagenomic fragments using deep learning" (Manuscript ID: GIGA-D-18-00464). We are very grateful to your substantial and helpful advices on the manuscript. Herein we first thank three reviewers and are pleasant to know of their overall positive comments about our work as the findings being important to the community with closely related research interests. We thank so many substantial and valuable comments from three reviewers, including their careful reading and check to the manuscript, which clearly helped us improve the paper. In the revised manuscript, we marked all the changed words, sentences or paragraphs in red text.

Following your instruction and the reviewers' comments, we have made a conscious effort to revise the manuscript (both the main document and Additional Files) with an essential improvement. Before we describe the revisions and responses to Reviewers' comments, we would like to first report our revisions following Editor's instructions. In particular, more appropriate data from prophage and genomic island databases have been used to evaluate PPR-Meta and the comparative tools (Please refer to Line 5-12, Page 20, and Line 5-15, Page 31 in the revised manuscript). We have made some improvements to the PPR-Meta tool to ameliorate the performance, and "*whether the tool will automatically decide which model to use for prediction*" is described more clearly in the manuscript (Please refer to Line 15, Page 12-Line 8, Page 13, Subsection "Structure of deep learning neural networks" in the revised manuscript.). We have also tested PPR-Meta on a series of metagenomic datasets of the human digestive tract, including the gut, throat and oral cavity, from the Human Microbiome Project (HMP). The findings are interesting and may be significant to the study of human health. In addition, we have registered PPR-Meta in the SciCrunch.org database and have added the RRID (SCR_016915) to Subsection "Availability of supporting source code and requirements" (Please refer to Line 9, Page 35 in the revised manuscript). All the outputs of the new analysis as well as the scripts for data preprocessing and neural network training have also been uploaded to the GigaScience server and our website. This time we uploaded the files separately instead of as one "zip" file in our previous submission, so that it could be updated more easily. Correspondingly, in the revised manuscript the link in Line 18, Page 34 has been changed to http://cqb.pku.edu.cn/ZhuLab/PPR_Meta/data/, and Section "Availability of supporting data" has been revised to: "The artificial contigs, related scripts and original results are available at http://cqb.pku.edu.cn/ZhuLab/PPR_Meta/data/. All the other data are available at corresponding references mentioned in the main text." (Please refer to Line 12-14, Page 35 in the revised

manuscript.) Meanwhile, we found that we missed some information in Section "Acknowledgements" and we have added the sentence "Part of the analysis was performed on the High Performance Computing Platform of the Center for Life Science of Peking University." to the Section "Acknowledgements". (Please refer to Line 14-16, Page 37 in the revised manuscript). More details are listed in the following point-by-point-responses to the reviewers' comments.

We then report our revisions and responses to three reviewers' all comments (*italic text*) one by one as follows:

## *To Reviewer #1:*

**General Comments:**

*The article by Fang et al describes a novel tool aimed at simultaneous identification of viral and plasmid sequences in metagenomic datasets. Presented tool can be of great value to many researchers analyzing microbiome data, especially for those interested in viral communities and/or mobile elements involved in the AMR spread and may as such also be widely cited. Authors used the novel neural network approach, which improves sequence classification for shorter sequences over other existing tools. Especially, usage of both noncoding and coding information is an important improvement over other existing tools, what authors clearly show in their manuscript.*

Herein we are glad to see Reviewer 1's positive comments on the present work as "*... be of great value to many researchers analyzing microbiome data, especially for those interested in viral communities and/or mobile elements involved in the AMR spread and may as such also be widely cited.*" We especially thank Reviewer 1 for his/her careful reading of our manuscript. The suggestions and comments raised by Reviewer 1 were certainly very helpful for us to improve the manuscript. Below, we itemize the revisions in response to Reviewer 1's points.

*1. Authors do not state what is the exact target of their software. Should it be used mainly with the metagenomic assemblies or can it be also easily applied to raw sequencing reads (because one of the models used in the work was trained on relatively short sequences (100-400 bp))? How will it work with isolate genome sequences?*

We first thank Reviewer 1 for reminding us with a clear statement about the exact target of PPR-Meta. In the current work, PPR-Meta is primarily designed for metagenomic assemblies generated by next-generation sequencing technology. However, there are many sequences that are assembled poorly or even remain unassembled with short length in most metagenomic assemblies, especially the sequences from low abundance species or in condition of low coverage sequencing. This is the reason why we also trained and tested PPR-Meta using short sequences from 100 to 400 bp. So PPR-Meta can also be easily applied to raw sequencing reads. The only requirement for users is to dispose of all sequences in a "fasta" format file. To make the target clearer for readers, we

revised the sentence in Section "Abstract" as follows: "We present PPR-Meta, a three-class classifier that allows simultaneous identification of both phage and plasmid fragments from metagenomic assemblies." (Please refer to Line 8-10, Page 2 in the revised manuscript.)

Of course, PPR-Meta can also work with isolated genome sequences. We collected complete genomes of the Acidianus tailed spindle virus (NC_029316.1), Ichthyobacterium seriolicida (AP014564.1) and Methylobacterium populi plasmid pMPPM01 (AP014810.1) from the test set (shown in the first line in the corresponding sheet in Additional File 1), and we found that PPR-Meta could correctly identify them as a phage, chromosome and plasmid, respectively. However, identifying complete genomes is not the main purpose of PPR-Meta because this is not a difficult issue and has been well addressed by other tools.

In addition, we have added a new section to provide an example application to show how PPR-Meta can be used to analyse metagenomic data. We employed PPR-Meta to identify phage and plasmid sequences on a series of metagenomic datasets of the human digestive tract, including the gut, throat and oral cavity, from the Human Microbiome Project (HMP). The finding is interesting and may be significant to the study of human health. We found that in the position closer to the outer end of the digestive tract, the percentages of phages and plasmids tended to be higher. For example, in the gut, the inner end of the digestive tract, the percentages of phages and plasmids were lower; in the oral cavity, the outer end of the digestive tract, the percentages of phages and plasmids were higher. Please refer to the new section "Phages and plasmids in the human digestive tract" for more details (Please refer to Line 18, Page 24 - Line 21, Page 25 in the revised manuscript.).

*2. Although the training procedure and the algorithm itself are quite well described I missed some details regarding data preprocessing and preparation of models. What parameters were used in MetaSim to generate sequences used for training? Authors should note that MetaSim does not produce artificial contigs but synthetic sequencing reads, with technology-specific errors introduced. Did authors use 'exact' preset to return fragments perfectly matching reference sequences?*

*It would be also great if all the codes and scripts (e.g. those for preprocessing of sequences, and neural network training) are available online or as Supplemental materials.*

Herein we realized that we did not provide a clear description of the data pre-processing and the preparation of the models. Actually, we used MetaSim to generate synthetic sequencing reads without technology-specific errors, that means we just used MetaSim to extract DNA sequences with different lengths that perfectly match reference sequences or that be modified with base substitutions or indels that are evenly distributed over the sequences. To make this process clearer, we have added the description to Section "Methods" as: "To generate artificial contigs with no error for both training and test set, we used the "exact" preset to return fragments exactly matching reference sequences. In each group, the "DNA Clone Size Distribution Type" was set to "Uniform". To generate artificial contigs modified with sequencing errors, we used the "Sanger" preset, which

allowed users to modify sequences according to their settings. Note that as we were not going to generate sequences with technology-specific errors, the following settings do not reflect the real situation of the Sanger technology. For the generation of sequences with 1% base substitutions, the "Read Length Distribution Type" was set to "Uniform", and the "Mate Pair Probability" was set to 0; both the "Error Rate at Read Star" and the "Error Rate at End of Read" were set to 0.01; and both the "Insertion Error Rate" and "Deletion Error Rate" were set to 0. For the generation of sequences with 1% base insertions or deletions, most settings were the same as mentioned above, except that both the "Insertion Error Rate" and "Deletion Error Rate" were set to 0.5." (Please refer to Line 5-19, Page 33 in the revised manuscript.)

Also, we have added the following sentences in Subsection "Structure of deep learning neural networks" to describe how we prepared the BiPathCNN and selected hyperparameters: "The selection of the related hyperparameters of each path mentioned above was referred to LeNet-5 [37] and VGG [38], two classic Convolutional Neural Networks in the field of artificial intelligence. Specifically, the distribution of layers was referred to LeNet-5, which contained three convolution layers, and there was a pooling layer between every two convolution layers. Meanwhile, the distribution of the number of convolution kernels was referred to VGG, in which the number of convolution kernels in the different layers was increased by doubling. We also referred to VGG to use ReLU as the activation function." (Please refer to Line 5-12, Page 12 in the revised manuscript). The citations of LeNet-5 and VGG have also been added to the list of the References.

In addition, we have uploaded the Keras scripts for the construction of the neural network as well as other scripts for data preprocessing to the GigaScience Database and our website (Link: http://cqb.pku.edu.cn/ZhuLab/PPR_Meta/data/). We wish that this is the best way to provide access to readers who want to reproduce or improve PPR-Meta.

*3. Have authors tried to build model similar to BiPathCNN for longer sequences? As authors claim that codon path is beneficial for distinguishing plasmids, phages and chromosomes (p. 16, lines 7-10), this additional information should increase kmer-based approach, especially that longer fragments are more likely to contain coding sequences.*

*I also wonder why authors chose the hexamer frequencies and not any odd-number kmer?*

We thank Reviewer 1 for this suggestion of using BiPathCNN for longer sequences.

Firstly, we would like to describe the improvements that we made in the revised version of the PPR-Meta tool, which may also be closely related to the other comments. In the original version of PPR-Meta, we built four neural network models for sequences of different lengths. Among these neural networks, we used BiPathCNN, which contains a base path and a codon path, for model A, B and C, and we used a Fully Connected Neural Network (FNN), which takes *k*-mer frequencies as inputs, for model D. In the revised version of the PPR-Meta tool, we removed model D and kept model A, B and C. In practical applications, PPR-Meta uses model A to predict sequences between 100 and 400 bp, model B to predict sequences between 400 and 800 bp, and model C to predict

sequences between 800 and 1200 bp. For sequences longer than 1200 bp, a scan window will move across the sequence without overlapping, and the weighted average of all windows' predictions is calculated. The length of the window is set to 1200 bp (or less if the window is beyond the sequence boundary). For example, given a sequence of length 2500 bp, the scan window will first cover the bases from the 1st to 1200th positions, then the window will move to the bases from the 1201st to 2400th positions, and finally, the window will move to the bases from the 2401st to 2500th positions. Then, PPR-Meta uses model C, model C and model A to predict the subsequences under the first, second and third windows, respectively. To generate the final score for the whole sequence, PPR-Meta calculates the weighted average of these windows. The weights of these three windows are 1200/2500, 1200/2500 and 100/2500, respectively.

We made this change because we found that the revised version of PPR-Meta could achieve a higher performance on long sequences. For example, for sequences with a length of 30k bp, the AUCs of both the phage identification and plasmid identification demonstrate higher performance. In particular, the TPR of phages increases from 93.76% to 99.84%, and almost all phages were identified. Although most sequences in the current metagenomic data are short fragments, a few reads from high-abundance species can be assembled into long contigs containing tens of thousands of bases, and we think that the revised PPR-Meta can be better adapted to these species. Additionally, considering that the third-generation sequencing technology is becoming more and more widely used, we hope that PPR-Meta can also promote studies using long sequencing technology, even though PPR-Meta is designed primarily for the next-generation sequencing technology. In the revised manuscript, we have added comparisons with related tools using sequences longer than 10k bp and sequences from real third-generation sequencing technology, which we will mention in the responses to the below comments.

We then address the questions in this comment. We tried to train BiPathCNN for longer sequences, but we failed to do this because it was very time consuming and had high hardware requirements. Thus, using a scan window to move across a long sequence may be a good alternative. In terms of the reason we used the 6-mer in the original version of PPR-Meta, it seems that the choice of k is not so significant. We tried different k values around 6, such as 5 and 7, and found that the results were comparable. In our opinion, the design of the neural network structure may be more important than the choice of the k value.

Because of the improvements we made in the revised PPR-Meta tool, as we mentioned at the beginning of this response, some of the results in the manuscript have also been updated. Herein, we would like to describe the updated content in our manuscript that is the result of these changes. None of the updated results mentioned below affect any conclusions that we have made in this manuscript. The revised version of the PPR-Meta tool has slight differences only on long sequences, while most of the test data we used in the manuscript are shorter than 5k bp, which is dominant in the current metagenomic sequences, and the revised PPR-Meta generates the same results for sequences shorter than 5k bp. Thus, the magnitude of all of the changes is small, except that the

program has a longer running time for sequences longer than 5k bp, as shown in item (15) below.

The changes in the manuscript include the following:

(1). The original Figure 2, which describes the structure of the FNN, was removed. The second paragraph form the last in Subsection “Structure of deep learning neural networks”, which describes the FNN, was also removed.

(2). In Subsection “Mathematical model of DNA sequences”, the sentence “Here, we use a more detailed approach to represent the short sequences in Group A, Group B and Group C.” has been revised to “Here, we use a more detailed approach to represent the DNA fragments.”(Please refer to Line 7-8, Page 9 in the revised manuscript.) Also, the last paragraph of this section, which described using k-mer to represent DNA fragments in Group D, was removed.

(3). In Subsection “Structure of deep learning neural networks”, the sentences “…we trained corresponding neural networks for each group. For Group A, B and C, we designed BiPathCNN to improve the performance (Figure 1).” has been revised to: “…we trained three neural networks for Group A, B and C. To improve the performance, we designed BiPathCNN (Figure 1), a novel neural network structure, to make reliable predictions.”(Please refer to Line 15-17, Page 10 in the revised manuscript.)

(4). In Figure 2 in the revised manuscript (as Figure 3 in the original manuscript), the confusion matrix of Group D was updated. Also, in Subsection “Overall performance”, the phrase “shown in Figure 3” has been revised to “shown in Figure 2”. (Please refer to Line 15, Page 13 in the revised manuscript.)

(5). In Figure 4, the ROCs of Group D, which described the potential of using life_score and trans_score to classify the phage lifestyle and plasmid transmissibility, were updated. Also, the legend of Figure 4 has been revised to: “(a) Classify virulent phages and temperate phages using life_score. In order of sequence length, the AUC is 0.63, 0.69, 0.71 and 0.76. (b) Classify transmissible plasmid and non-transmissible plasmid using trans_score. In order of sequence length, the AUC is 0.58, 0.55, 0.60 and 0.62.

(6). In Section “Methods”, Line 21-22, Page 33, the sentence “Considering the memory size, running time and accuracy, a total of 3,060,000 artificial contigs were generated to train PPR-Meta.” has been revised to “Considering the memory size, running time and accuracy, a total of 2,700,000 artificial contigs were generated to train PPR-Meta.” Also, the phrase “and 120,000 from Group D” has been removed from the sentence “The number of training contigs of each phage, chromosome and plasmid is 300,000 from Group A to C and 120,000 from Group D.” (Please refer to Line 22, Page 33-Line 2, Page 34 in the revised manuscript.)

(7). In Table 1, Table 3 and Table 4, the performance of PPR-Meta on Group D (the fifth row from the last of each table) was updated.

(8). In Table 5, the prophage recognition rate of PPR-Meta on Group D (the third row from the last) was updated.

(9). In Subsection “Performance comparison”, the sentence “The TPR of PPR-Meta was

approximately 3%~13% higher than that of VirFinder and the FPR was approximately 6%~9% lower" has been revised to "The TPR of PPR-Meta was approximately 10% higher than that of VirFinder, and the FPR was approximately 5~10% lower." (Refer to Line 13-15, Page 15 in the revised manuscript.)

(10). In Subsection "Performance comparison", the sentences "For PPR-Meta, our FPR was much lower than that of cBar and PlasFlow. Although PPR-Meta achieved a slightly lower TPR than PlasFlow in Group A, our TPR remained highest in all other cases" have been revised to "For PPR-Meta, the TPR was comparable with that of PlasFlow, while the FPR was approximately 25~40% lower." (Please refer to Line 1-2, Page 16 in the revised manuscript.)

(11). In Subsection "Evaluation in real metagenomic data", the sentence "VirFinder and PPR-Meta were much better than VirSorter and identified 68.86% and 76.88% of the contigs, respectively, showing that PPR-Meta had the highest coverage of this data set" has been revised to "VirFinder and PPR-Meta were much better than VirSorter and identified 68.86% and 76.90% of the contigs, respectively, showing that PPR-Meta had the highest coverage of this data set." (Refer to Line 21, Page 21 in the revised manuscript.)

(12). In Subsection "Evaluation in real metagenomic data", the sentences "For PPR-Meta, total of 82.00% of the sequences were identified as MGEs, in which 49.16% were phages and 32.84% were plasmids. More than half of the sequences (64.72%) predicted as phages by PPR-Meta were also predicted as phages by VirFinder, and most of the sequences (74.72%) predicted as plasmids by PPR-Meta were also predicted by PlasFlow" have been revised to "For PPR-Meta, total of 81.96% of the sequences were identified as MGEs, in which 49.18% were phages and 32.78% were plasmids. More than half of the sequences (64.73%) predicted as phages by PPR-Meta were also predicted as phages by VirFinder, and most of the sequences (74.74%) predicted as plasmids by PPR-Meta were also predicted by PlasFlow." (Please refer to Line 16-20, Page 22 in the revised manuscript.)

(13). In Subsection "Evaluation in real metagenomic data", the sentence "In terms of phage identification, PPR-Meta, VirFinder and VirSorter predicted an average of 4.18%, 11.03% and 0% of the 16S-like contigs as phages, respectively, indicating that PPR-Meta likely generated fewer false positive predictions than VirFinder" has been revised to "In terms of phage identification, PPR-Meta, VirFinder and VirSorter predicted an average of 3.43%, 11.32% and 0% of the 16S-like contigs as phages, respectively, indicating that PPR-Meta likely generated fewer false positive predictions than VirFinder." (Please refer to Line 17, Page 23 in the revised manuscript.)

(14). In Subsection "Evaluation in real metagenomic data", the sentence "In terms of plasmid identification, PPR-Meta, PlasFlow and cBar predicted an average of 25.46%, 53.74% and 63.83% of the 16S-like contigs as plasmids, respectively, indicating that the PPR-Meta may generate the lowest number of false positive predictions" has been revised to "In terms of plasmid identification, PPR-Meta, PlasFlow and cBar predicted an average of 26.69%, 52.57% and 63.36% of the 16S-like contigs as plasmids, respectively, indicating that the PPR-Meta may generate the lowest number of false positive predictions." (Refer to Line 22, Page 23 in the revised manuscript.)

(15). In Subsection "Usage of PPR-Meta", the sentence "We tested the running time of PPR-Meta using 90,000 sequences from 100 to 10k bp and found that this tool can handle all sequences in approximately 15 minutes on a machine with the following configuration: CPU: Intel Core i7 6700; GPU: NVIDIA GTX1060; and Memory: 64G, DDR4" has been revised to: "… and found that this tool can handle all sequences in approximately 45 minutes on a machine with the following configuration …"(Please refer to Line 10, Page 27 in the revised manuscript.)

In items (13) and (14), the results of the comparative tools were also slightly different because we found that one setting in the data pre-processing script may not be the best option. Thus, we re-ran the pre-processing procedure to make the results more precise. The dataset pre-processing procedure is provided in Section "Methods" (Please refer to Line 3-16, Page 34 in the revised manuscript), and the data pre-processing script (file "20_gut.sh") is stored on our website and in the GigaScience Database.

*4. I find really interesting part in which authors used likelihood scores generated by PPR-Meta to predict phage lifestyle or plasmid transmissability. Results shown are really encouraging and may make PPR-Meta an important tool in MGE studies. In the discussion of this phenomenon authors should cite the paper of Suzuki et al. (https://www.ncbi.nlm.nih.gov/pmc/articles/PMC2976448/), which discusses usage of genome signatures to predict the evolutionary host range of plasmids. Additionally, similarity between host and plasmid nucleotide composition is a known phenomenon, and so called genome amelioration is a term properly describing convergence of plasmid and host sequence patterns. Therefore, the statement "Since temperate phages and non-transmissible plasmids experience longer residence times within the host cell, they may adjust the sequence pattern toward the host" seems correct.*

We appreciate Reviewer 1 for providing a reference that supports our hypothesis. Firstly, to make the description more rigorous, we have revised the sentence "This phenomenon may be due to the adaptation of foreign DNA to the host" to "This phenomenon may be due to the genome amelioration of foreign DNA to the host." (Please refer to Line 7, Page 30 in Section "Discussion and conclusions".) In addition, we have added the following sentence to the revised manuscript as: "For example, research has shown that the comparison of the trinucleotide composition between a plasmid and bacterial chromosome can be used to predict the host range of plasmids [53]." (Please refer to Line 8-10, Page 30 in Section "Discussion and conclusions".) The new citation has also been added to the list of the References.

*5. For me it is really worth emphasizing that authors tested their software in the context of 3rd generation sequencing technologies like PacBio and Nanopore. As there is more and more reports showing applicability of single molecule sequencing technologies for describing microbial communities it would be really great to have tool which which is tested and works well on such datasets. Authors used 1% error rates for their simulated datasets, however, in my opinion they*

*should test also higher error rates, as for aforementioned technologies they can reach up to 10%, error rate, especially for indels. Both technologies are sensitive to homopolymer sequences therefore one would expect more errors in such regions. Additionally I would also like to see if PPR-Meta is able to predict phage/plasmid sequences on real 3rd generation sequencing data. They can use for example recently published virome (https://www.biorxiv.org/content/early/2018/11/12/345041.full.pdf+html) or mock microbial community (https://www.biorxiv.org/content/early/2018/12/04/487033.full.pdf+html) nanopore data. The former one can also help with "Since we lack samples in which only chromosomes are enriched and all the extrachromosomal elements are filtered, estimating whether related tools will misjudge chromosomes as MGEs directly is difficult using real data" (p. 21, l. 9-11)*

We would like to thank Reviewer 1 for the concern about testing PPR-Meta using artificial contigs with a higher error rate and real third generation sequencing data. In the revised manuscript, we tested PPR-Meta and related tools using artificial contigs with a high error rate. We used MetaSim to generate artificial contigs modified with 10% base substitutions and 10% indels in Group D, whose lengths were close to the raw reads generated from third generation sequencing data. The two types of errors were tested separately. The results showed that the AUCs of PPR-Meta remained the highest (>90%), although the performance was somewhat fluctuating, especially in the presence of 10% indels. Please refer to Additional File 3, Figure S3, for more details. On the other hand, we consider that a tool that can tolerate a 1% error rate is competent for handling the third generation sequencing data. Although the error rate of third generation sequencing technology can reach up to 10%, many basecalling tools have been developed to help improve the accuracy to over 99%. Thus, we believe that PPR-Meta can generate reliable predictions on the third sequencing data that go through Quality Control (QC). We have added the following sentences to the revised manuscript as: "Considering that the error rate of the raw data generated from the third-generation sequencing technology may be much higher, we also tested PPR-Meta and the related tools using artificial contigs modified with 10% base substitutions and 10% insertions or deletions in Group D, whose lengths were close to the raw reads generated from third-generation sequencing technology. The results are shown in Additional File 3 and Figure S3. The results showed that the AUCs of PPR-Meta remained the highest (>90%), although the performance was somewhat fluctuating, especially in the presence of 10% insertions or deletions. Recently, many basecalling tools for the third-generation sequencing technology have been developed to help improve the accuracy over 99% [41], therefore the extremely high error rate on the raw data will not affect the usage of PPR-Meta." (Please refer to Line 4-15, Page 19 in Subsection "Performance in the presence of sequencing errors" in the revised manuscript.) The new citation has also been added to the list of the References.

We also tested whether PPR-Meta and the related tools can predict phage sequences on real third generation sequencing data using virome data from the reference provided by Reviewer 1. The results showed that PPR-Meta could identify more viruses in this dataset. We have added the following sentences to the revised manuscript as: "Considering that the third-generation sequencing

technology is more and more widely used to analyse metagenomes, we also used real virome data generated by MinION [46] to test whether PPR-Meta and the related tools can identify phages from third-generation sequencing technology. The virome was downloaded as assembled sequences (accession: GCA_900491955.1), containing 1500 sequences. The results showed that PPR-Meta, VirFinder and VirSorter could identify 79.20%, 76.27% and 30.40% of viral sequences respectively, indicating that PPR-Meta has the highest performance. Therefore PPR-Meta can also handle data from the third-generation sequencing technology, although it is designed primarily for the next-generation sequencing technology." (Please refer to Line 6-16, Page 24 in Subsection "Evaluation in real metagenomic data".) The new citation has also been added to the list of the References.

*6. It should be also noted that in case of plasmid prediction PPR-Meta presents different approach than previously published cBar and PlasFlow only for sequences shorter than 10kb, as neural network trained on Group D essentially use the 6-mer frequencies for prediction. Additionally in the PlasFlow manual it is said that such approach does not work well on short sequences (recommended length is > 1kb), therefore comparisons using test datasets from GroupA and GroupB should be done with having this in mind. In my opinion authors should also comment on different lengths of sequences used for training in cBar, PlasFlow and PPR-Meta: cBar was trained with whole chromosome/plasmid sequences whereas PlasFlow on 10kb fragments and PPR-Meta using 4 datasets with differing lengths, what may significantly influence their performance.*

We thank Reviewer 1 for reminding us to note that PPR-Meta uses a similar approach to cBar and PlasFlow for long sequences. As we mentioned in the response to General Comment #3, in the revised version of the PPR-Meta tool, we removed the k-mer-based Fully Connected Neural Network (FNN) and instead used BiPathCNN to predict sequences of all lengths, which helped to improve its performance for very long sequences. Indeed, we found that the k-mer-based approach did not work well for short sequences. For example, we used the FNN in the original PPR-Meta tool to predict the sequences in Group A (100~400 bp), and we found that the performance was much poorer: the AUC of phage identification decreased from 91.84% to 72.70%, and the AUC of plasmid identification decreased from 83.05% to 64.79%. One of the innovations of PPR-Meta is that we do not use k-mer frequencies to represent DNA sequences, as we have emphasized in our manuscript. For example, we mentioned that "**Although k-mer frequencies have been widely used in many studies, such frequencies may present serious fluctuations in short sequences**" and "**The performance improvement on short sequences demonstrates that our sequences representation method is more detailed than the k-mer frequencies**". To further emphasize the difference between PPR-Meta and the comparative tools, we have added the following sentence to the revised manuscript as: "Overall, PPR-Meta presented a much better performance than other homology-search-based tools such as VirSorter and k-mer-based tools such as VirFinder, PlasFlow and cBar." (Please refer to Line 11-13, Page 16 in Subsection "Performance comparison".)

In our opinion, the k-mer-based methods are more sensitive to sequence length than our

BiPathCNN. The distribution of the k-mer frequencies may be different between long sequences and short sequences, and the variance of the k-mer frequencies for short sequences may be much higher. Thus, the k-mer-based classifier constructed using short sequence data may not by applicable for long sequences, and vice versa. We also tested the accuracy of each BiPathCNN on test sets from other groups, as per Reviewer 1's suggestion in Specific Comment #7. We found that although the overall accuracy was slightly reduced when testing each group using the non-corresponding BiPathCNNs from the other groups, the decrease was not obvious (Please refer to Additional File 3, and Figure S2), indicating that our approach was not quite sensitive to the sequence length. We have added the following sentences to Section "Discussion and conclusions" in the revised manuscript as: "On the other hand, k-mer-based methods may also be more sensitive to the sequence length than the BiPathCNN method in the current work. The distribution of k-mer frequencies may be different between long sequences and short sequences, and the variance of the k-mer frequencies for short sequences may be much higher. Thus, the k-mer-based classifier constructed using short sequence data may not by applicable for long sequences, and vice versa. Among the k-mer-based tools, cBar was trained with complete genomes and PlasFlow was trained on 10k bp fragments, which might make them hard to adapt to metagenomic data with a wide range of lengths. Differently, our BiPathCNN directly extracts sequence features from the raw data represented by the one-hot matrix and may be less sensitive to the sequence length. Tests of each BiPathCNN on test datasets from different groups (Additional File 3, Figure S2) also showed that although the overall accuracy was slightly reduced when testing each group using a non-corresponding BiPathCNN from the other groups, the decrease was not obvious, indicating that our approach is not quite sensitive to the sequence length." (Please refer to Line 5-20, Page 28 in the revised manuscript.)

**Specific Comments:**

*1. Page 5 lines 2-4: "extracted" should be "extract"*

We thank Reviewer 1 for the careful check of our manuscript, and we have corrected this mistake. The sentence has been revised to "Such approaches primarily used a scan window to move across the complete bacterial chromosome and extract regions that seem to be phages based on a similarity search against viral databases." (Please refer to Line 4, Page 5, in Section "Introduction".) Meanwhile, after proofreading our manuscript, we have also revised some phrases to improve the language and presentation. Herein we list the revision as follows:

Line 4, Page 15 and Line 14, Page 22, "In term of" have been revised to "In terms of".

Line 20, Page 15, "appearing" has been revised to "appeared".

Line 22, Page 31, "sequencing" has been revised to "sequenced".

Line 16, Page 9, "For BOH" has been revised to "For BOH in PPR-Meta".

Line 11-12, Page 20, "The decline in performance may be due to…" has been revised to "The lower recognition rate of prophages compared with that of the phages in the NCBI database may be due to…"

Line 20-21, Page 28, we have replaced "Moreover…" with "Another shortcoming of k-mer-based tools may be that…"

2. *Page 5 line 21: Authors may also cite following tool: https://www.ncbi.nlm.nih.gov/pubmed/30383524*.

We thank Reviewer 1 for reminding us that we missed a related tool in our manuscript. Now, the corresponding sentence has been revised to "In terms of plasmids, most of the current tools for plasmid identification were designed for WGS or even specific species, such as PlasmidFinder [20], PLACNET [21], PlasmidSeeker [22] and mlplasmids [23]." (Please refer to Line 21, Page 5, in Section "Introduction".)

3. *Page 6 line 6 "this tool applies SMO" should be changed to: "this tool applies SOM" (Self Organizing Map)*

We thank Reviewer 1 for this careful check. According to the reference of cBar, SMO refers to "sequential minimal optimization". To make it clearer to readers, we have revised the sentence to "This tool applies sequential minimal optimization (SMO) as a classifier based on k-mer frequencies." (Please refer to Line 6, Page 6, in Section "Introduction".) In addition, the term "SMO" has also been added to Section "List of abbreviations." (Please refer to Line 3, Page 36.)

4. *page 8 lines 13-19 "phage metagenomic data of bovine rumen [19], which were downloaded from MG-RAST [29] (Accessions: mgm4534202.3 and mgm4534203.3) as raw reads and assembled by SPAdes" and "20 samples of healthy human gut [32], downloaded from the NCBI Short Read Archive [33] and assembled by SPAdes." I miss details on SPAdes assembly. What settings were used and what was the quality of assembly (N50, number of contigs and so on).*

We apologize that we missed some details in our manuscript. In the revised manuscript, we have added these details to Section "Methods" as follows: "We also used real metagenomic data to evaluate PPR-Meta and the related tools. We used SPAdes to assemble the raw reads, as we mentioned in the main text. The phage metagenomic data of the bovine rumen were downloaded from MG-RAST, and we used the command "spades.py --meta -1 file1.fastq -2 file2.fastq -o out_folder" to assemble the pair-end raw reads. In the assembly, the contig number, N50, average length, maximum length and minimum length were 107529, 288, 312.06, 75508 and 56, respectively. To download the 20 samples of the healthy human gut, we used the command "prefetch SRRaccession" from the SRA Toolkit. All samples were downloaded as ".sra" files. We then used the command "fastq-dump --split-files accession.sra" from the SRA Toolkit to convert the sra file into two pair-end fastq files and used SPAdes with the same settings as mentioned above to assemble the raw reads. The information about the contig number, N50, average length, maximum length and minimum length is provided in Additional File 1." (Please refer to Line 3-16, Page 34 in the revised manuscript.) Also, the sentence "Additional details on the dataset construction are provided in

Methods section." has been moved from the second paragraph from the last to the last paragraph in Section "Dataset construction". (Please refer to Line 1, Page 9 in the revised manuscript.)

In addition, the scripts used to calculate the quality of the assemblies were provided in the GigaScience Database and on our website.

*5. page 12 lines 5-11: Which approach is used for sequences 5-10 kb? FNN or biPath-CNN (groupC model)? If biPath-CNN - what is its performance on this dataset compared to FNN (group D)? It is not clear which approach the software will use for the real datasets of this length.*

We are very sorry that we did not provide a clear statement of this issue in our manuscript. As we mentioned in the response to General Comment #3, we removed the FNN from the revised PPR-Meta tool. For sequences longer than 1200 bp, a scan window is used to make a prediction. To make this clearer to the readers, we have added the following sentences to the revised manuscript as: "In practical applications, PPR-Meta uses BiPathCNN A to predict sequences between 100 and 400 bp, BiPathCNN B to predict sequences between 400 and 800 bp, and BiPathCNN C to predict sequences between 800 and 1200 bp. For sequences longer than 1200 bp, such as sequences in Group D, a scan window will move across the sequence without overlapping, and the weighted average of all windows' predictions is calculated. The length of the window is set to 1200 bp (or less if the window ends beyond the sequence boundary). For example, given a sequence of length 2500 bp, the scan window will first cover the bases from the 1st to 1200th positions, then the window will move to bases from the 1201st to 2400th positions, and finally, the window will move to bases from the 2401st to 2500th positions. Then, PPR-Meta uses BiPathCNN C, BiPathCNN C and BiPathCNN A to predict the subsequences under the first, second and third windows, respectively. To generate the final score for the whole sequence, PPR-Meta calculates the weighted average of these windows. The weights of these three windows are 1200/2500, 1200/2500 and 100/2500, respectively." (Please refer to Line 15, Page 12-Line 8, Page 13, Subsection "Structure of deep learning neural networks" in the revised manuscript.)

*6. I would also like to see the comparison to other software regarding fragments longer than 10kb (which are easily achievable with current metagenomic sequencing techniques).*

We would like to thank Reviewer 1 for this concern about making comparisons for fragments longer than 10k bp. We have added comparisons on 15k and 30k bp fragments to the revised manuscript, and the performance of PPR-Meta is still the best. The results are shown in Additional File 3, Figure S1, and we have added the following sentences to the revised manuscript as: "In some cases, a few assembled sequences from high-abundance species may be much longer, so we also tested PPR-Meta and the related tools using 15k bp and 30k bp fragments (shown in Additional File 3, Figure S1). The results showed that the performance of PPR-Meta was still the best for these long sequences." (Please refer to Line 2-6, Page 16, Subsection "Performance comparison" in the revised manuscript.)

*7. Additionally, please test accuracy of each model on testing datasets from other models, e.g. model for group A on test datasets for groups B, C and D, model for group B on test datasets for groups A, C and D, etc. It is not likely that in real datasets sequences will be distributed such uniformly. It is also interesting how biPath-CNN performs on long sequences, as coding information should significantly increase its performance (like it was shown for shorter fragments). And, maybe, any of the single models is good enough to be used on fragments of all lengths; for me this possibility can not be excluded by looking at presented data.*

We understand the consideration of Reviewer 1 that using a single model may be good enough for fragments of all lengths. In fact, using different neural networks for sequences of different groups can not only help to improve the accuracy but also speed up the program. As shown in Additional File 3, Figure S2, we tested the accuracy as well as the running time of each neural network on the test datasets from all groups. The results showed that using a non-corresponding neural network from another group to predict sequences from a specific group would lead to a lower accuracy and longer running time (the reason will be described below). Indeed, the sequence lengths in a real dataset may not be distributed uniformly. In many cases, the distribution of the sequence lengths in real metagenomic data is more like a Poisson distribution, with most of the lengths around 0.5k-2k bp and a few of them much longer. Since most of the sequences are short, we considered that it is essential to construct different neural networks for short sequences. As we mentioned in the response to General Comment #3, we also tried to train BiPathCNN for long contigs but failed because it was very time consuming and had high hardware requirements. As an alternative, PPR-Meta uses a scan window for long sequences and predict the subsequence in each window using the corresponding BiPathCNN. The evaluation on long sequences, such as sequences of 15k bp and 30k bp, has shown the effectiveness of this approach.

Herein, we would like to explain why using a non-corresponding BiPathCNN would increase the running time. In fact, once the BiPathCNN is constructed, the input size of the neural network is fixed. For example, the BiPathCNN of Group C can handle sequences with a maximum length of 1200 bp. If the BiPathCNN of Group C is used to predict a sequence of 100 bp, the "base one-hot matrix" must be padded with a number of rows of [0,0,0,0], in which all bits are zero, to adapt the input size of the neural network and so does the "codon one-hot matrix". In general, padding with zeros does not significantly affect the accuracy but will add unnecessary calculations for the neural network, which will also increase the running time. Similarly, the BiPathCNN of Group A can handle sequences with a maximum length of 400 bp. If the BiPathCNN of Group A is used to predict a sequence of 1200 bp, a scan window must be used to split the sequence into 3 subsequences of 400 bp. Each subsequence will be predicted separately, and then an average score for the whole sequence will be calculated. Since the total number of sequences is increased, the running time will also be longer, although the accuracy will not be significantly reduced, as we showed in Figure S2. Overall, we consider that the usage of three BiPathCNNs together with the scan window for long

sequences in the revised version of PPR-Meta may be a good choice for handling sequences of different lengths.

In the revised manuscript, we have added the following sentence as: "In addition, we tested the accuracy as well as the running time of each BiPathCNN on test datasets from different groups and found that using a non-corresponding BiPathCNN to predict sequences from specific groups would lead to a lower accuracy and longer running time (shown in Additional File 3, Figure S2)." (Please refer to Line 6-10, Page 16, Subsection "Performance comparison" in the revised manuscript.)

*8. Page 15 line 12: I would remove the word "obviously". Please be less advertising and more informative.*

We are sorry for our inappropriate description. In the revised manuscript, this sentence has been removed because this sentence was part of the discussion of the performance of the FNN that we used in the original version of PPR-Meta, but we removed the FNN in the revised version.

*9. page 16 lines 10-13: "Compared with other sequence representation methods that ignore the coding or non-coding region, such as method based on k-mer frequencies, PPR-Meta uses a more detailed method of describing a sequence and achieves a higher performance." Authors should explicitly note that it relates only to sequences shorter than 5(or 10, see my note above) kb.*

We thank Reviewer 1 for bringing to our attention this inappropriate description. However, as we mentioned in the response to General Comment #3, the revised version of the PPR-Meta tool employs BiPathCNN for all sequences. Thus, this description seems to be appropriate in the revised manuscript.

*10. Page 22, lines 16-17: "PPR-Meta is designed with the option to adjust the default threshold of discriminant criteria" It should be described more precisely. Although in the Manual it is noted, that "In this way, sequences with the phage (or plasmid) score higher than the other two categories and the threshold will be regarded as phage (or plasmid)", it is not mentioned that sequences not exceeding threshold for phage or plasmid category will fall into the "chromosome" category, what may not be the best option, increasing False Negative Rate. I also lack more information on the accuracy of PPR-Meta run with different thresholds (Table S1). Can you include also AUC in the table? And compare with PlasFlow, using the same thresholds?*

We thank Reviewer 1 for this comment. We quite agree that sequences not exceeding the threshold for the phage or plasmid category will fall into the chromosome category may increase the False Negative Rate. In the revised version of PPR-Meta, we referred to the threshold usage of PlasFlow. Specifically, given a threshold by a user, the sequence with the highest score lower than the threshold will be labelled as "uncertain". In this way, the outputs of PPR-Meta contain six categories: phage, uncertain phage, chromosome, uncertain chromosome, plasmid and uncertain plasmid. In Additional File 3, Figure S5, we evaluated the uncertain prediction rate, accuracy, AUC,

TPR and FPR under different thresholds. The accuracy was defined as the ratio of the number of correctly predicted fragments to the total number of fragments, rather than being calculated on either the phages or plasmids separately. Thus, the accuracy can reflect the overall performance of PPR-Meta. The accuracy, AUC, TPR and FPR were calculated only on the certain predictions. In general, with a higher threshold, the accuracy, AUC, and TPR as well as the uncertain prediction rate will be higher, while the FPR will be lower. We have added the following sentences to the revised manuscript as: "To meet users' actual requirements, PPR-Meta is designed with the option to adjust the threshold to filter out the uncertain predictions so that the remaining predictions may be more reliable. Given a threshold, a sequence with a highest score lower than the threshold will be labelled as "uncertain". In this way, the outputs of PPR-Meta contain six categories: phage, uncertain phage, chromosome, uncertain chromosome, plasmid and uncertain plasmid. We evaluated the uncertain prediction rate, accuracy, AUC, TPR and FPR under different thresholds, and the results are shown in Additional File 3, Figure S5. In general, with a higher threshold, the accuracy, AUC, and TPR as well as the uncertain prediction rate will be higher, while the FPR will be lower." (Please refer to Line 11-21, Page 26, Subsection "Usage of PPR-Meta" in the revised manuscript.)

Herein, we compared the AUC of plasmid identification between PPR-Meta and PlasFlow, both using 0.7 as the threshold (the default threshold in PlasFlow). In Group A (100-400 bp), the AUC of PPR-Meta increased from 83.05% to 90.61%, while 53.36% of sequences were labelled as uncertain; the AUC of PlasFlow increased from 56.30% to 77.05%, while 37.70% of sequences were labelled as uncertain. In Group B (400-800 bp), the AUC of PPR-Meta increased from 89.64% to 93.99%, while 32.71% of sequences were labelled as uncertain; the AUC of PlasFlow increased from 62.50% to 81.85%, while 39.34% of sequences were labelled as uncertain. In Group C (800-1200 bp), the AUC of PPR-Meta increased from 91.84% to 94.68%, while 23.40% of sequences were labelled as uncertain; the AUC of PlasFlow increased from 68.01% to 84.79%, while 38.21% of sequences were labelled as uncertain. In Group D (5k-10k bp), the AUC of PPR-Meta increased from 96.02% to 97.63%, while 25.11% of sequences were labelled as uncertain; the AUC of PlasFlow increased from 88.42% to 93.86%, while 25.46% of sequences were labelled as uncertain.

*11. This is only the suggestion, but all the data presenting performance of PPR-Meta in comparison to other tools can be also presented as graphs, what would allow for easy assessment of differences.*

We thank Reviewer 1 for this suggestion. In the main text, we presented the results as tables to make the information more precise. However, we quite agree that too many tables will make the manuscript difficult to read. Thus, we presented all of the supplementary results mentioned above as graphs in Additional File 3 to make them easy to assess. Correspondingly, the description of Additional File 3 in the "Additional file" section has been revised to: "Additional file 3: Figure S1 to Figure S5". (Please refer to Line 19, Page 35.)

**Specific Comments Regarding Software Usability**

*1. Using output file extension other than .csv throws an error:*
*Error using writetable (line 124)*
*Unrecognized file extension '.tsv'.  Use the 'FileType' parameter to specify the file type*
*Error in PPR-Meta(line 124)*
*MATLAB:table:write:UnrecognizedFileExtension*
*This should be better documented, and the user should be warned at the beginning of computation that using custom file extensions will cause that output file cannot be written.*

We thank Reviewer 1 for testing our program and discovering the "bug" in PPR-Meta. We have addressed this "bug", and PPR-Meta will now automatically check the extension. If the user does not use ".csv" as an extension, the program will directly add the ".csv" extension to the output file and give a warning. We have added the following sentences to the manual to remind users of this: "Note: the current version of PPR-Meta uses "comma-separated values (CSV)" as the format of the output file. Please use ".csv" as the extension of the output file. PPR-Meta will automatically add the ".csv" extension to the file name if the output file does not take ".csv" as its extension." (Please refer to Section 5, Part □ in the manual.)

***To Reviewer #2:***

**General Comments:**

*Authors present a tool which is able to perform multiclass prediction of phage, plasmid and chromosome sequences in metagenomic data. Using this deep learning approach along with its architecture and the weighted average of all windows is worthy of publication by itself. Really interesting. Tool, source code, virtual machine (along with a video explaining how to use it) and supporting datasets are all publicly available. Also, the discussion section is really interesting. Noting that the differences between chromosome and phage scores may reflect phage lifestyle may be an relevant finding.*

Herein we first appreciate Reviewer 2's positive comments on our present work. We would like to especially thank Reviewer 2 for these comments and suggestions, which were certainly helpful for us to improve our work. For Reviewer 2's following concerns on our work, we present our responses and the corresponding improvements or revisions as follows.

*1. I am somehow concerned by the biological support of trying to predict these three classes. My concern in mainly about the overlap characteristics that plasmid and chromosomes usually present. And the same thing may be said about phages/prophages, chromosomes and plasmids. An example of this is explored by authors in results reported in page 20.*

*As a suggestion, authors should state clearly that the tool is aimed to perform a three-class prediction. Perhaps adding sentences to the abstract or even to the title.*

We fully understand Reviewer 2's concern about the biological support of trying to predict

these three classes. Indeed, genome amelioration is widely observed in foreign DNA, so it is not strange that plasmids (or phages) and chromosomes usually present overlap characteristics. In fact, the similarity of the nucleotide composition between plasmids (or phages) and chromosomes is often used to predict the host of a given plasmid or phage (see Galiez et al., WIsH: who is the host? Predicting prokaryotic hosts from metagenomic phage contigs. Bioinformatics, 2017; 33(19): 3113-3114; Suzuki et al., Predicting plasmid promiscuity based on genomic signature. J. Bacteriol., 2010; 192(22): 6045–6055.) However, the similarity of the nucleotide compositions between plasmids (or phages) and chromosomes is not contradictory with using the characteristics of the nucleotide composition to identify plasmids or phages because the plasmids or phages can still maintain some specific sequence patterns. For example, experiments conducted by Ren et al. showed that a virus and its host shared some similar k-mers, which could help predict the host of a given virus, while viruses would also share more similar k-mers with each other, which could help distinguish them from the hosts (see Ren et al., VirFinder: a novel k-mer based tool for identifying viral sequences from assembled metagenomic data. Microbiome, 2017; 5(1): 69.). Thus, we consider that using the nucleotide composition to identify phages and plasmids is biologically feasible.

To make it clearer to readers that PPR-Meta is intended to perform a three-class prediction, we have revised the sentence in Section "Abstract" as: "We present PPR-Meta, a three-class classifier that allows simultaneous identification of both phage and plasmid fragments from metagenomic assemblies." (Please refer to Line 8-10, Page 2 in the revised manuscript.) We have also revised the sentence in Section "Introduction" as: "In this paper, we present the PPR-Meta (Phage and Plasmid Recognizer for Metagenomes), a three-class classifier for identifying metagenomic fragments as phages, plasmids or chromosomes based on the deep learning algorithm." (Please refer to Line 19, Page 6 in the revised manuscript.) In addition, we have added a new subsection to provide an example application to show how PPR-Meta can be used to analyse metagenomic data. We employed PPR-Meta to identify phage and plasmid sequences on a series of metagenomic datasets of the human digestive tract, including the gut, throat and oral cavity, from the Human Microbiome Project (HMP). The finding is interesting and may be significant to the study of human health. We found that in positions closer to the outer end of the digestive tract, the percentages of phages and plasmids tended to be higher. For example, in the gut, the inner end of the digestive tract, the percentages of phages and plasmids were lower, while in the oral cavity, the outer end of digestive tract, the percentages of phages and plasmids were higher. Please refer to the new subsection "Phages and plasmids in the human digestive tract" for more details (Please refer to Line 18, Page 24 - Line 21, Page 25 in the revised manuscript.).

**Minor comments:**

*1. Page 5, line 13: "However, research has shown that viral sequences are highly fragmented in the metagenome [19], which may prevent binning, thereby limiting the usage of MARVEL." This is an unfair affirmation, since its only supporting reference is a 2013 article and it is safe to say that*

*much has been done to improve metagenomic assemblers since then. Recent publications have been reporting the retrieval of phage complete and/or almost complete genomes by only applying assembly and binning approaches. I refer specially to the IMG-VR database (versions 1.0 and 2.0), which is a repository of thousands of viral sequences retrieved from metagenomic datasets all around the world. Nonetheless, there are many other publications in this line such as:*

*Paez-Espino, David, et al. "IMG/VR: a database of cultured and uncultured DNA Viruses and retroviruses." Nucleic acids research (2016): gkw1030.*

*Paez-Espino, David, et al. "IMG/VR v. 2.0: an integrated data management and analysis system for cultivated and environmental viral genomes." Nucleic acids research (2018).*

*Paez-Espino, David, et al. "Uncovering Earth's virome." Nature 536.7617 (2016): 425.*

*Sangwan, Naseer, Fangfang Xia, and Jack A. Gilbert. "Recovering complete and draft population genomes from metagenome datasets." Microbiome 4.1 (2016): 8.*

Herein, we agree with Reviewer 2 so that we could have an appropriate way to describe this point. We thus have revised these sentences in an appropriate way to "The tool MARVEL can assign metagenomic bins as phages or bacteria and demonstrates better performance than previous tools. In the other hand, in order to identify sequences from low-abundance phages, which may fall into binning, we also need tools that can directly judge each fragment." (Please refer to Line 12-16, Page 5, Section "Introduction" in the revised manuscript.) Also, we have removed the sentence "Compared with the other tools, VirFinder is more suitable for metagenomes" from Section "Introduction".

*2. Page 27, line 12 and other parts of the article: It is not clear whether authors extracted prophages from chromosomes to train the algorithm with more phages or to remove noise from chromosome datasets. Do the chromosome datasets still contain their prophages?*

We realize that we did not provide a clear description of the dataset construction. In fact, both adding more phages and removing noise from the chromosome dataset are the reasons that we extracted the prophages from the chromosomes. On the one hand, the number of phage genomes in the current RefSeq database is much less than that of bacterial chromosomes, while the abundance of viruses in real microbial communities is estimated to be much higher than that of bacteria. At the time we constructed PPR-Meta, we downloaded 10,090 complete prokaryote chromosomes, while only 2,279 completed phages were collected. Thus, extracting prophages from prokaryote chromosomes may be a good approach to expand the phage training set. On the other hand, some bacteria contain several prophages, which may account for up to 20% of the host chromosome. If all of these prophages were labelled as chromosomes when training PPR-Meta, the accuracy would be reduced, especially the sensitivity of phage identification. Thus, in the training set, all of the extracted prophages were directly added to the phage dataset, and the chromosome dataset did not contain prophages. However, all these prophages were predicted by ProphET, a software to extract prophages from prokaryote chromosomes based on similarity search, and were not subjected to

experimental verification. Thus, these prophages could not be used as a benchmark. Therefore, in the test set, we directly removed the prophages, and neither the phage dataset nor the chromosome dataset contain the prophages, which we have emphasized in the manuscript. To test the prophage identification of PPR-Meta and the related tools, we additionally collected 267 manually annotated prophages. (Please refer to Line 2, Page 8, Subsection "Dataset construction".) These manually annotated prophages have widely been used as benchmarks for related computational software, such as Prophinder, Phage_Finder, PHAST, PHASTER and VirSorter. To make this clearer to readers, we have added the following sentence to the revised manuscript as: "Moving prophages from a chromosome dataset to a phage dataset can help to both expand the phage dataset and remove noise from the chromosome dataset." (Please refer to Line 20-22, Page 7, Subsection "Dataset construction" in the revised manuscript.)

*3. A more practical question: For each query given by the user, will the tool automatically decide which model to use for prediction? Page 12 states how authors have proceeded by each query size, but my question regards the tool's behavior.*

We are sorry that we missed some details about the tool's behaviour, and then address our response to Reviewer 2's question as follows.

Firstly, we would like to describe some improvements that we have made in the revised version of the PPR-Meta tool, which is closely related to this comment. In the original version of PPR-Meta, we built four neural network models for sequences of different lengths. Among these neural networks, we used BiPathCNN, which contains a base path and a codon path, for model A, B and C, and we used a Fully Connected Neural Network (FNN), which takes k-mer frequencies as inputs, for model D. In the revised version of the PPR-Meta tool, we removed model D and kept model A, B and C. In practical applications, PPR-Meta uses model A to predict sequences between 100 and 400 bp, model B to predict sequences between 400 and 800 bp, and model C to predict sequences between 800 and 1200 bp. For sequences longer than 1200 bp, a scan window will move across the sequence without overlapping, and the weighted average of all windows' predictions is calculated. The length of the window is set to 1200 bp (or less if the window is beyond the sequence boundary). For example, given a sequence of length 2500 bp, the scan window will first cover the bases from the 1st to 1200th positions, then the window will move to the bases from the 1201st to 2400th positions, and finally, the window will move to the bases from the 2401st to 2500th positions. Then, PPR-Meta uses model C, model C and model A to predict the subsequences under the first, second and third windows, respectively. To generate the final score for the whole sequence, PPR-Meta calculates the weighted average of these windows. The weights of these three windows are 1200/2500, 1200/2500 and 100/2500, respectively.

We made this change because we found that the revised version of PPR-Meta could achieve a higher performance on long sequences. For example, for sequences with a length of 30k bp, the AUCs of both the phage identification and plasmid identification are higher. In particular, the TPR

of phages increases from 93.76% to 99.84%, and almost all phages were identified. Although most sequences in the current metagenomic data are short fragments, a few reads from high-abundance species can be assembled into long contigs containing tens of thousands of bases, and we think that the revised PPR-Meta can be better adapted to these species. Additionally, considering that the third-generation sequencing technology is becoming more and more widely used, we hope that PPR-Meta can also promote studies using long sequencing technology, even though PPR-Meta is designed primarily for the next-generation sequencing technology. In the revised manuscript, we have added comparisons between PPR-Meta and the related tools using 15k bp and 30k bp sequences, which are much longer than the sequences used for the comparisons in the main text. The results showed that PPR-Meta was still the best performing tool. (Please refer to Additional File 3, Figure S1.) In addition, we also used real metagenomic data of viromes generated from third-generation sequencing technology. The results showed that PPR-Meta could identify more sequences as phages compared with the related tools. (Please refer to Line 6-16, Page 24, in the "Evaluation in real metagenomic data" section.)

We now answer the question in this comment. Actually, the PPR-Meta tool's behaviour is the same as our above description about the practical applications of PPR-Meta. To make the PPR-Meta tool's behaviour clearer, we have added the following sentences to the revised manuscript as: "In practical applications, PPR-Meta uses BiPathCNN A to predict sequences between 100 and 400 bp, BiPathCNN B to predict sequences between 400 and 800 bp, and BiPathCNN C to predict sequences between 800 and 1200 bp. For sequences longer than 1200 bp, such as sequences in Group D, a scan window will move across the sequence without overlapping, and the weighted average of all windows' predictions is calculated. The length of the window is set to 1200 bp (or less if the window ends beyond the sequence boundary). For example, given a sequence of length 2500 bp, the scan window will first cover the bases from the 1st to 1200th positions, then the window will move to bases from the 1201st to 2400th positions, and finally, the window will move to bases from the 2401st to 2500th positions. Then, PPR-Meta uses BiPathCNN C, BiPathCNN C and BiPathCNN A to predict the subsequences under the first, second and third windows, respectively. To generate the final score for the whole sequence, PPR-Meta calculates the weighted average of these windows. The weights of these three windows are 1200/2500, 1200/2500 and 100/2500, respectively." (Please refer to Line 15, Page 12-Line 8, Page 13, Subsection "Structure of deep learning neural networks" in the revised manuscript.)

Because of the improvements we made in the revised PPR-Meta tool, as we mentioned at the beginning of this response, some of the results in the manuscript have also been updated. Herein, we would like to describe the updated content in our manuscript that is the result of these changes. None of the updated results mentioned below affect any conclusions that we have made in this manuscript. The revised version of the PPR-Meta tool has slight differences only on long sequences, while most of the test data we used in the manuscript are shorter than 5k bp, which is dominant in the current metagenomic sequences, and the revised PPR-Meta generates the same results for

sequences shorter than 5k bp. Thus, the magnitude of all of the changes is small, except that the program has a longer running time for sequences longer than 5k bp, as shown in item (15) below.

The changes in the manuscript include the following:

(1). The original Figure 2, which describes the structure of the FNN, was removed. The second paragraph form the last in Subsection "Structure of deep learning neural networks", which describes the FNN, was also removed.

(2). In Subsection "Mathematical model of DNA sequences", the sentence "Here, we use a more detailed approach to represent the short sequences in Group A, Group B and Group C." has been revised to "Here, we use a more detailed approach to represent the DNA fragments."(Please refer to Line 7-8, Page 9 in the revised manuscript.) Also, the last paragraph of this section, which described using k-mer to represent DNA fragments in Group D, was removed.

(3). In Subsection "Structure of deep learning neural networks", the sentences "…we trained corresponding neural networks for each group. For Group A, B and C, we designed BiPathCNN to improve the performance (Figure 1)." has been revised to: "…we trained three neural networks for Group A, B and C. To improve the performance, we designed BiPathCNN (Figure 1), a novel neural network structure, to make reliable predictions."(Please refer to Line 15-17, Page 10 in the revised manuscript.)

(4). In Figure 2 in the revised manuscript (as Figure 3 in the original manuscript), the confusion matrix of Group D was updated. Also, in Subsection "Overall performance", the phrase "shown in Figure 3" has been revised to "shown in Figure 2". (Please refer to Line 15, Page 13 in the revised manuscript.)

(5). In Figure 4, the ROCs of Group D, which described the potential of using life_score and trans_score to classify the phage lifestyle and plasmid transmissibility, were updated. Also, the legend of Figure 4 has been revised to: "(a) Classify virulent phages and temperate phages using life_score. In order of sequence length, the AUC is 0.63, 0.69, 0.71 and 0.76. (b) Classify transmissible plasmid and non-transmissible plasmid using trans_score. In order of sequence length, the AUC is 0.58, 0.55, 0.60 and 0.62.

(6). In Section "Methods", Line 21-22, Page 33, the sentence "Considering the memory size, running time and accuracy, a total of 3,060,000 artificial contigs were generated to train PPR-Meta." has been revised to "Considering the memory size, running time and accuracy, a total of 2,700,000 artificial contigs were generated to train PPR-Meta." Also, the phrase "and 120,000 from Group D" has been removed from the sentence "The number of training contigs of each phage, chromosome and plasmid is 300,000 from Group A to C and 120,000 from Group D." (Please refer to Line 22, Page 33-Line 2, Page 34 in the revised manuscript.)

(7). In Table 1, Table 3 and Table 4, the performance of PPR-Meta on Group D (the fifth row from the last of each table) was updated.

(8). In Table 5, the prophage recognition rate of PPR-Meta on Group D (the third row from the last) was updated.

(9). In Subsection "Performance comparison", the sentence "The TPR of PPR-Meta was approximately 3%~13% higher than that of VirFinder and the FPR was approximately 6%~9% lower" has been revised to "The TPR of PPR-Meta was approximately 10% higher than that of VirFinder, and the FPR was approximately 5~10% lower." (Refer to Line 13-15, Page 15 in the revised manuscript.)

(10). In Subsection "Performance comparison", the sentences "For PPR-Meta, our FPR was much lower than that of cBar and PlasFlow. Although PPR-Meta achieved a slightly lower TPR than PlasFlow in Group A, our TPR remained highest in all other cases" have been revised to "For PPR-Meta, the TPR was comparable with that of PlasFlow, while the FPR was approximately 25~40% lower." (Please refer to Line 1-2, Page 16 in the revised manuscript.)

(11). In Subsection "Evaluation in real metagenomic data", the sentence "VirFinder and PPR-Meta were much better than VirSorter and identified 68.86% and 76.88% of the contigs, respectively, showing that PPR-Meta had the highest coverage of this data set" has been revised to "VirFinder and PPR-Meta were much better than VirSorter and identified 68.86% and 76.90% of the contigs, respectively, showing that PPR-Meta had the highest coverage of this data set." (Refer to Line 21, Page 21 in the revised manuscript.)

(12). In Subsection "Evaluation in real metagenomic data", the sentences "For PPR-Meta, total of 82.00% of the sequences were identified as MGEs, in which 49.16% were phages and 32.84% were plasmids. More than half of the sequences (64.72%) predicted as phages by PPR-Meta were also predicted as phages by VirFinder, and most of the sequences (74.72%) predicted as plasmids by PPR-Meta were also predicted by PlasFlow" have been revised to "For PPR-Meta, total of 81.96% of the sequences were identified as MGEs, in which 49.18% were phages and 32.78% were plasmids. More than half of the sequences (64.73%) predicted as phages by PPR-Meta were also predicted as phages by VirFinder, and most of the sequences (74.74%) predicted as plasmids by PPR-Meta were also predicted by PlasFlow." (Please refer to Line 16-20, Page 22 in the revised manuscript.)

(13). In Subsection "Evaluation in real metagenomic data", the sentence "In terms of phage identification, PPR-Meta, VirFinder and VirSorter predicted an average of 4.18%, 11.03% and 0% of the 16S-like contigs as phages, respectively, indicating that PPR-Meta likely generated fewer false positive predictions than VirFinder" has been revised to "In terms of phage identification, PPR-Meta, VirFinder and VirSorter predicted an average of 3.43%, 11.32% and 0% of the 16S-like contigs as phages, respectively, indicating that PPR-Meta likely generated fewer false positive predictions than VirFinder." (Please refer to Line 17, Page 23 in the revised manuscript.)

(14). In Subsection "Evaluation in real metagenomic data", the sentence "In terms of plasmid identification, PPR-Meta, PlasFlow and cBar predicted an average of 25.46%, 53.74% and 63.83% of the 16S-like contigs as plasmids, respectively, indicating that the PPR-Meta may generate the lowest number of false positive predictions" has been revised to "In terms of plasmid identification, PPR-Meta, PlasFlow and cBar predicted an average of 26.69%, 52.57% and 63.36% of the 16S-like contigs as plasmids, respectively, indicating that the PPR-Meta may generate the lowest number of

false positive predictions.” (Refer to Line 22, Page 23 in the revised manuscript.)

(15). In Subsection “Usage of PPR-Meta”, the sentence “We tested the running time of PPR-Meta using 90,000 sequences from 100 to 10k bp and found that this tool can handle all sequences in approximately 15 minutes on a machine with the following configuration: CPU: Intel Core i7 6700; GPU: NVIDIA GTX1060; and Memory: 64G, DDR4” has been revised to: “… and found that this tool can handle all sequences in approximately 45 minutes on a machine with the following configuration …”(Please refer to Line 10, Page 27 in the revised manuscript.)

In items (13) and (14), the results of the comparative tools were also slightly different because we found that one setting in the data pre-processing script may not be the best option. Thus, we re-ran the pre-processing procedure to make the results more precise. The dataset pre-processing procedure is provided in Section “Methods” (Please refer to Line 3-16, Page 34 in the revised manuscript), and the data pre-processing script (file “20_gut.sh”) is stored on our website and in the GigaScience Database.

***To Reviewer #3:***

**General Comments:**

*1. This paper describes PPR-Meta - a tool for identifying phages and plasmids from metagenomic fragments using deep learning. The authors distribute the software through their own site as Virtual Machine and through Github as a set of Matlab and h5 model files. The distribution is very nonintuitive and unconventional. It would be far better for the authors to distribute as a standard git package, rather than as a .zip file. Furthermore, the requirement that the use download a 30gb Virtual Machine image is odd and not at all standard in bioinformatics. The installation of PPR-Meta should be possible without having to instantiate an entire VM. I would greatly prefer a makefile and configure script with the requirement of a Ubuntu OS, over downloading a 30gb over http. This may qualify as a major revision, because I assume switching to this would require a lot of additional work.*

Herein we thank Reviewer 3 for this helpful suggestion on the distribution of the PPR-Meta tool. Following this suggestion, we have distributed PPR-Meta as a standard git package, and an archival copy of the PPR-Meta package is also available via our website. Users can directly download PPR-Meta using the command “git clone https://github.com/zhenchengfang/PPR-Meta.git” and the executable file is under the folder. We have also updated the file “README.md” in GitHub.

However, we are sorry that it is hard for us to provide a configure script for a "one click"-type installation. PPR-Meta relies on two Python Deep Learning libraries, TensorFlow and Keras, whose installations also rely on related packages in the operating system. In addition, if users want to speed up the program with a GPU, they need to install additional support software, as we mentioned in the manual. Thus, some details of the PPR-Meta installation may be slightly different under different

system environments. Actually for computer professionals, it is not difficult to install the program following the manuals of PPR-Meta and the dependent packages. In order to make PPR-Meta more user-friendly for non-computer professionals, we followed the suggestion of the journal's Author Guidelines to optimize PPR-Meta in a virtual machine. We also provided a brief step-by-step video guide to show how to run PPR-Meta in the virtual machine. Therefore in this way, any non-computer professional who is not familiar with the command line can easily run PPR-Meta on a local PC.

In the revised version of our work, the virtual machine has also been reconfigured. To minimize the size of the virtual machine, we used the "Minimal installation" mode to configure the virtual machine. We further compressed the virtual machine into a ".7z" file, which can easily be decompressed using a current compressing software such as "WinRAR". Now, the size of the virtual machine has been reduced from 30 GB to about 2.5 GB, more than 10 times smaller and we stored the virtual machine on our website. In addition, according to the journal's requirements, copies of the PPR-Meta package and virtual machine are also stored in the GigaScience database. In the future, we will update the packages stored on different websites synchronously.

*2. There are some basic issues in the paper in detecting phage vs. prophage vs. plasmid. I think the paper leans heavily on phage and plasmid databases, over which it excels in a metagenomic context, but doesn't address the large corpus of prophage and genomic island databases in the literature. I would prefer these to be addressed and compared. The entire literature on genomic islands, including experimental, feature-based and machine-learning based methods for identification are largely missing. The authors use ProphET to identify the prophages. This is a machine learning based approach, and not a structure or experimental-based gold standard dataset.*

Herein we understand the consideration of Reviewer 3 about how PPR-Meta and the related tools perform in the large corpus of prophage and genomic island databases. Indeed, in our original manuscript, we used only 267 manually annotated prophages from Casjens to evaluate PPR-Meta and the related tools.

Firstly, we would like to explain why we used only 267 manually annotated prophages and ignored the other prophage databases. We looked at several prophage databases and found that most prophages in the current prophage databases were predicted by related computational software and had not been experimentally verified. Thus, these prophages may not be appropriate to be used as benchmarks. For example, the prophages in the ACLAME database were predicted by the Prophinder tool; the prophages in the PHASTER database were predicted by the PHASTER tool; the prophages in the PHANTOME database were predicted by several tools, while only a few of them were manually verified; the prophages in ProphageDB were predicted using similarity searches (see Srividhya et al. (2006). Database and comparative identification of prophages. *Lec. Notes Control Informat. Sci.*) and we failed to access its website (http://bicmku.in:8082/prophagedb; http://ispc.weizmann.ac.il/prophagedb). In contrast, the 267 manually annotated prophages are of high quality and have widely been used as benchmarks for computational software, such as

Prophinder, Phage_Finder, PHAST, PHASTER and VirSorter. Thus, to make the evaluation more reliable, we used only these 267 manually annotated prophages in our original manuscript. During the revision of the manuscript, we found that the PHANTOME database also contains some prophages that were manually verified. Thus, as a supplement, we have used these prophages to evaluate PPR-Meta and the related tools in the revised manuscript. The newly collected dataset contained 139 prophages from 50 bacteria, and most of the hosts of these prophages were not the same as those of the previous 267 prophages. Therefore, the prophages from PHANTOME can be regarded as a new dataset. The results are shown in Additional File 3, Figure S4. Consistent with the results on the 267 prophages, the prophage recognition rate of PPR-Meta on the prophages from PHANTOME was about 20% higher than that of the comparative tools, indicating that PPR-Meta can identify more prophages than the related tools. We have added the following sentences to the revised manuscript as: "We additionally collected 139 manually verified prophages from the PHANTOME database [42], and most of the hosts of these prophages were not the same as those of the previous 267 prophages. Consistent with the results for the 267 prophages, the prophage recognition rate of PPR-Meta was much higher than that of the comparative tools (shown in Additional File 3, Figure S4), indicating that PPR-Meta can identify more prophages." (Please refer to Line 5-12, Page 20, Subsection "Prophage identification ability" in the revised manuscript.) The new citation has also been added to the list of the References.

Secondly, we would like to explain why we excluded the genomic island databases in our original manuscript. Compared with the prophages, the mechanisms of genomic islands are more diverse. The formation of genomic islands may be caused by various elements such as phages, plasmids, conjugative transposons, integrative and conjugative elements (ICEs), as well as nonreplicative but excisable elements. Since PPR-Meta is designed primarily for phage and plasmid identification, identifying some types of genomic islands seems to be another new work for this study because not all genomic islands are closely related to phages or plasmids. However, phages and plasmids are two of the most important elements that mediate the formation of genomic islands, and we quite agree with Reviewer 3 that we should discuss how the related tools perform in genomic islands and make a comparison. Therefore, we collected sequences of genomic islands from the Islander database. Upon testing on artificial contigs extracted from these genomic islands, the results showed that PPR-Meta could identify more genomic islands as foreign DNA (either phages or plasmids) than VirFinder, VirSorter, PlasFlow or cBar. This result indicated that PPR-Meta had a better ability to recognize sequences from regions of horizontal origin on bacterial chromosomes. We have added the following sentences to Section "Discussion and conclusions" in the revised manuscript as: "In general, bacteria contain genomic islands, regions of horizontal origin on chromosomes [54]. The formation mechanisms of some genomic islands are caused by phages or plasmids [55]. To see how PPR-Meta and related tools perform on DNA fragments from these regions, we collected genomic island sequences from the Islander database [56]. Upon testing on artificial contigs between 100 bp and 10k bp extracted from these genomic islands, the results

showed that PPR-Meta could identify 65.25% of them as foreign DNA (either phage or plasmid), while VirFinder, VirSorter, PlasFlow and cBar could identify 20.46%, 6.72%, 53.11% and 51.62% of them, respectively, indicating that PPR-Meta has a better ability to recognize sequences from regions of horizontal origin on bacterial chromosomes." (Please refer to Line 5-15, Page 31 in the revised manuscript.) The new citations have also been added to the list of the References.

For the last point in this comment, we have noted that the prophages predicted by ProphET could not be used as standard dataset. As we mentioned in the manuscript, we used only these predicted prophages as a training set to expand the phage dataset, not as a test set. In the test set, all prophages predicted by ProphET were removed. (Please refer to Line 22, Page 7-Line 2, Page 8, Subsection "Dataset construction".) We evaluated only the prophage identification using manually annotated prophages, as we mentioned above. Thus, the evaluation of the prophages is reliable.

*3. Figures 1 and 2, describing the bi-path convolutional network could be considerably clearer. Specifically the whole process would be clearer if the "base one-hot matrix" and "codon one-hot matrix" were better understood. Furthermore, the description of layers should be clear enough in the figure that they are unnecessary in the text. Finally, justification for the NN architecture is not offered. Is this a standard architecture? Was it randomly created? Was it optimized?*

Herein we apologize that we did not provide a clear description of the Neural Network construction. We then address our response to Reviewer 3's comment as follows.

Firstly, we would like to describe the improvements that we made in the revised version of the PPR-Meta tool, which is closely related to this comment and also is similar to Reviewer 1's comment. In the original version of PPR-Meta, we built four neural network models for sequences of different lengths. Among these neural networks, we used BiPathCNN, which contains a base path and a codon path, for model A, B and C, and we used a Fully Connected Neural Network (FNN), which takes k-mer frequencies as inputs, for model D. In the revised version of the PPR-Meta tool, we removed model D and kept model A, B and C. In practical applications, PPR-Meta uses model A to predict sequences between 100 and 400 bp, model B to predict sequences between 400 and 800 bp, and model C to predict sequences between 800 and 1200 bp. For sequences longer than 1200 bp, a scan window will move across the sequence without overlapping, and the weighted average of all windows' predictions is calculated. The length of the window is set to 1200 bp (or less if the window is beyond the sequence boundary). For example, given a sequence of length 2500 bp, the scan window will first cover the bases from the 1st to 1200th positions, then the window will move to the bases from the 1201st to 2400th positions, and finally, the window will move to the bases from the 2401st to 2500th positions. Then, PPR-Meta uses model C, model C and model A to predict the subsequences under the first, second and third windows, respectively. To generate the final score for the whole sequence, PPR-Meta calculates the weighted average of these windows. The weights of these three windows are 1200/2500, 1200/2500 and 100/2500, respectively.

We made this change because we found that the revised version of PPR-Meta could achieve a

higher performance on long sequences. For example, for sequences with a length of 30k bp, the AUCs of both the phage identification and plasmid identification are higher. In particular, the TPR of phages increases from 93.76% to 99.84%, and almost all phages were identified. Although most sequences in the current metagenomic data are short fragments, a few reads from high-abundance species can be assembled into long contigs containing tens of thousands of bases, and we think that the revised PPR-Meta can be better adapted to these species. Additionally, considering that the third-generation sequencing technology is becoming more and more widely used, we hope that PPR-Meta can also promote studies using long sequencing technology, even though PPR-Meta is designed primarily for the next-generation sequencing technology. In the revised manuscript, we have added comparisons between PPR-Meta and the related tools using 15k bp and 30k bp sequences, which are much longer than the sequences used for the comparisons in the main text. The results showed that PPR-Meta was still the best performing tool. (Please refer to Additional File 3, Figure S1.) In addition, we also used real metagenomic data of viromes generated from third-generation sequencing technology. The results showed that PPR-Meta could identify more sequences as phages compared with the related tools. (Please refer to Line 6-16, Page 24, Subsection "Evaluation in real metagenomic data" in the revised manuscript.)

We now answer the questions in this comment. Because of the changes we made, the original Figure 2, which described the FNN, was removed because the FNN was not included in the revised PPR-Meta tool. Following the advice of Reviewer 3, we thus have marked the details of the related hyperparameters of each layer in Figure 1, such as the number of convolution kernels and the length of the kernels. We still maintained the related description in the text to make it easier to understand by readers who are unfamiliar with deep learning.

For "one-hot", we are sorry that we missed some of the details in the manuscript. In fact, "one-hot" is one of the most widely used encoding forms for each character in a given string in the field of Natural Language Processing (NLP), and it is also used to represent bases or amino acids in biological sequences. "One-hot" refers to a vector containing several bits in which only one bit is 1 and the others are 0. For example, given a string containing 10 types of character, the "one-hot" vectors for each character contain 10 bits. For a specific character type, the corresponding bit of the "one-hot" vector is 1, and the remaining 9 bits are 0. It is not important which bit a specific character type corresponds to, but there must be a one-to-one correspondence between each character type and each bit. In PPR-Meta, we used the "one-hot" vectors [0,0,0,1], [0,0,1,0], [0,1,0,0] and [1,0,0,0] to represent each base and "one-hot" vectors containing 64 bits to represent each codon. That is, one base corresponded to one bit or one codon corresponded to one bit. To make it clearer for readers, we have added the following sentences to the "Mathematical model of DNA sequences" section of the revised manuscript as: ""One-hot" is one of the most widely used encoding forms for each character in a given string in the field of Natural Language Processing (NLP) [36], and it is also used to represent bases or amino acids in biological sequences. A "one-hot" vector contains several bits, and the number of bits is equal to the number of character types in a given string. For each

character type, the corresponding bit of the "one-hot" vector is 1 and the remaining bits are 0, and there must be a one-to-one correspondence between each character type and each bit." (Please refer to Line 9-16, Page 9 in the revised manuscript.) We have also cited a reference that introduces the "one-hot" encoding in the above description so that readers who are interested in the sequence encoding form can learn more about it.

For the last point in this comment about the justification for the NN architecture, we realize that we did not give a clear description of this. In the design of PPR-Meta, the framework of BiPathCNN was proposed by ourselves while the selection of the hyperparameters of each path was referred to LeNet-5 and VGG, two classic Convolutional Neural Networks in the field of artificial intelligence. To let PPR-Meta effectively extract features from both the coding and noncoding regions, we designed the base path and the codon path to handle the "base one-hot matrix" and "codon one-hot matrix", respectively. Our test in Table 2 showed that this structure was effective because the performance of the BiPathCNN was better than that of the single-path CNN. Furthermore, we referred to LeNet-5 and VGG to optimize each path. For the layer distribution, we referred to LeNet-5. LeNet-5 contains 3 convolution layers, and there is a pooling layer between every two convolution layers. In the base path and codon path of PPR-Meta, we followed this layer distribution. However, LeNet-5 only contains 6, 16 and 120 convolution kernels in each convolution layer, which seems to be not enough for PPR-Meta to present reliable predictions. Thus, we referred to VGG for the selection of the number of convolution kernels. The number of convolution kernels in the different layers of VGG is increased by doubling, from 64, 128, 256 to 512. In PPR-Meta, we followed this distribution in the base path and codon path, that is, the first, second and third convolution layers contained 64, 128 and 256 convolution kernels, respectively. In addition, we also referred to VGG to use ReLU as an activation function. To make the construction of the neural network clearer, we have added the following sentences to the "Structure of deep learning neural networks" section of the revised manuscript as: "The selection of the related hyperparameters of each path mentioned above was referred to LeNet-5 [37] and VGG [38], two classic Convolutional Neural Networks in the field of artificial intelligence. Specifically, the distribution of layers was referred to LeNet-5, which contained 3 convolution layers, and there was a pooling layer between every two convolution layers. Meanwhile, the distribution of the number of convolution kernels was referred to VGG, in which the number of convolution kernels in the different layers was increased by doubling. We also referred to VGG to use ReLU as the activation function." (Please refer to Line 5-12, Page 12 in the revised manuscript.) The new citations have also been added to the list of the References. In addition, we have also uploaded the scripts for the neural network construction as well as other data preprocessing scripts on our website and the GigaScience Database. We think that this is the best way to provide access to readers who want to reproduce or improve PPR-Meta.

Because of the improvements we made in the revised PPR-Meta tool, as we mentioned at the beginning of this response, some of the results in the manuscript have also been updated. Herein, we would like to describe the updated content in our manuscript that is the result of these changes.

None of the updated results mentioned below affect any conclusions that we have made in this manuscript. The revised version of the PPR-Meta tool has slight differences only on long sequences, while most of the test data we used in the manuscript are shorter than 5k bp, which is dominant in the current metagenomic sequences, and the revised PPR-Meta generates the same results for sequences shorter than 5k bp. Thus, the magnitude of all of the changes is small, except that the program has a longer running time for sequences longer than 5k bp, as shown in item (15) below.

The changes in the manuscript include the following:

(1). The original Figure 2, which describes the structure of the FNN, was removed. The second paragraph form the last in Subsection “Structure of deep learning neural networks”, which describes the FNN, was also removed.

(2). In Subsection “Mathematical model of DNA sequences”, the sentence “Here, we use a more detailed approach to represent the short sequences in Group A, Group B and Group C.” has been revised to “Here, we use a more detailed approach to represent the DNA fragments.”(Please refer to Line 7-8, Page 9 in the revised manuscript.) Also, the last paragraph of this section, which described using k-mer to represent DNA fragments in Group D, was removed.

(3). In Subsection “Structure of deep learning neural networks”, the sentences “…we trained corresponding neural networks for each group. For Group A, B and C, we designed BiPathCNN to improve the performance (Figure 1).” has been revised to: “…we trained three neural networks for Group A, B and C. To improve the performance, we designed BiPathCNN (Figure 1), a novel neural network structure, to make reliable predictions.”(Please refer to Line 15-17, Page 10 in the revised manuscript.)

(4). In Figure 2 in the revised manuscript (as Figure 3 in the original manuscript), the confusion matrix of Group D was updated. Also, in Subsection “Overall performance”, the phrase “shown in Figure 3” has been revised to “shown in Figure 2”. (Please refer to Line 15, Page 13 in the revised manuscript.)

(5). In Figure 4, the ROCs of Group D, which described the potential of using life_score and trans_score to classify the phage lifestyle and plasmid transmissibility, were updated. Also, the legend of Figure 4 has been revised to: “(a) Classify virulent phages and temperate phages using life_score. In order of sequence length, the AUC is 0.63, 0.69, 0.71 and 0.76. (b) Classify transmissible plasmid and non-transmissible plasmid using trans_score. In order of sequence length, the AUC is 0.58, 0.55, 0.60 and 0.62.

(6). In Section “Methods”, Line 21-22, Page 33, the sentence “Considering the memory size, running time and accuracy, a total of 3,060,000 artificial contigs were generated to train PPR-Meta.” has been revised to “Considering the memory size, running time and accuracy, a total of 2,700,000 artificial contigs were generated to train PPR-Meta.” Also, the phrase “and 120,000 from Group D” has been removed from the sentence “The number of training contigs of each phage, chromosome and plasmid is 300,000 from Group A to C and 120,000 from Group D.” (Please refer to Line 22, Page 33-Line 2, Page 34 in the revised manuscript.)

(7). In Table 1, Table 3 and Table 4, the performance of PPR-Meta on Group D (the fifth row from the last of each table) was updated.

(8). In Table 5, the prophage recognition rate of PPR-Meta on Group D (the third row from the last) was updated.

(9). In Subsection "Performance comparison", the sentence "The TPR of PPR-Meta was approximately 3%~13% higher than that of VirFinder and the FPR was approximately 6%~9% lower" has been revised to "The TPR of PPR-Meta was approximately 10% higher than that of VirFinder, and the FPR was approximately 5~10% lower." (Refer to Line 13-15, Page 15 in the revised manuscript.)

(10). In Subsection "Performance comparison", the sentences "For PPR-Meta, our FPR was much lower than that of cBar and PlasFlow. Although PPR-Meta achieved a slightly lower TPR than PlasFlow in Group A, our TPR remained highest in all other cases" have been revised to "For PPR-Meta, the TPR was comparable with that of PlasFlow, while the FPR was approximately 25~40% lower." (Please refer to Line 1-2, Page 16 in the revised manuscript.)

(11). In Subsection "Evaluation in real metagenomic data", the sentence "VirFinder and PPR-Meta were much better than VirSorter and identified 68.86% and 76.88% of the contigs, respectively, showing that PPR-Meta had the highest coverage of this data set" has been revised to "VirFinder and PPR-Meta were much better than VirSorter and identified 68.86% and 76.90% of the contigs, respectively, showing that PPR-Meta had the highest coverage of this data set." (Refer to Line 21, Page 21 in the revised manuscript.)

(12). In Subsection "Evaluation in real metagenomic data", the sentences "For PPR-Meta, total of 82.00% of the sequences were identified as MGEs, in which 49.16% were phages and 32.84% were plasmids. More than half of the sequences (64.72%) predicted as phages by PPR-Meta were also predicted as phages by VirFinder, and most of the sequences (74.72%) predicted as plasmids by PPR-Meta were also predicted by PlasFlow" have been revised to "For PPR-Meta, total of 81.96% of the sequences were identified as MGEs, in which 49.18% were phages and 32.78% were plasmids. More than half of the sequences (64.73%) predicted as phages by PPR-Meta were also predicted as phages by VirFinder, and most of the sequences (74.74%) predicted as plasmids by PPR-Meta were also predicted by PlasFlow." (Please refer to Line 16-20, Page 22 in the revised manuscript.)

(13). In Subsection "Evaluation in real metagenomic data", the sentence "In terms of phage identification, PPR-Meta, VirFinder and VirSorter predicted an average of 4.18%, 11.03% and 0% of the 16S-like contigs as phages, respectively, indicating that PPR-Meta likely generated fewer false positive predictions than VirFinder" has been revised to "In terms of phage identification, PPR-Meta, VirFinder and VirSorter predicted an average of 3.43%, 11.32% and 0% of the 16S-like contigs as phages, respectively, indicating that PPR-Meta likely generated fewer false positive predictions than VirFinder." (Please refer to Line 17, Page 23 in the revised manuscript.)

(14). In Subsection "Evaluation in real metagenomic data", the sentence "In terms of plasmid identification, PPR-Meta, PlasFlow and cBar predicted an average of 25.46%, 53.74% and 63.83%

of the 16S-like contigs as plasmids, respectively, indicating that the PPR-Meta may generate the lowest number of false positive predictions" has been revised to "In terms of plasmid identification, PPR-Meta, PlasFlow and cBar predicted an average of 26.69%, 52.57% and 63.36% of the 16S-like contigs as plasmids, respectively, indicating that the PPR-Meta may generate the lowest number of false positive predictions." (Refer to Line 22, Page 23 in the revised manuscript.)

(15). In Subsection "Usage of PPR-Meta", the sentence "We tested the running time of PPR-Meta using 90,000 sequences from 100 to 10k bp and found that this tool can handle all sequences in approximately 15 minutes on a machine with the following configuration: CPU: Intel Core i7 6700; GPU: NVIDIA GTX1060; and Memory: 64G, DDR4" has been revised to: "… and found that this tool can handle all sequences in approximately 45 minutes on a machine with the following configuration …"(Please refer to Line 10, Page 27 in the revised manuscript.)

In items (13) and (14), the results of the comparative tools were also slightly different because we found that one setting in the data pre-processing script may not be the best option. Thus, we re-ran the pre-processing procedure to make the results more precise. The dataset pre-processing procedure is provided in Section "Methods" (Please refer to Line 3-16, Page 34 in the revised manuscript), and the data pre-processing script (file "20_gut.sh") is stored on our website and in the GigaScience Database.

*4. Training on simulated data, rather than on a known set of MGEs was a strange choice, I would like to see this more sufficiently explained. In the prophage identification section, only 267 manually annotated prophages are included, when there are in fact thousands of annotated prophages and genomic islands.*

We thank Reviewer 3 for this concern about training PPR-Meta on a known set of MGEs, and we would like to explain why we chose to train PPR-Meta using simulated data. The most important reason that we trained PPR-Meta using artificial contigs extracted from sequenced complete genomes is that the source of these artificial contigs is more reliable and of high quality. Although there are some real metagenomic samples in which either phages or circular DNA are enriched, the enriched samples are often contaminated by the host chromosomes (see Roux S. et al. (2013). Assessment of viral community functional potential from viral metagenomes may be hampered by contamination with cellular sequences. *Open Biol.*). Besides, reagent and laboratory contamination may also affect the quality of real metagenomic data (see Salter SJ. et al. (2014). Reagent and laboratory contamination can critically impact sequence-based microbiome analyses. *BMC Biol.*). If we use these low-quality data containing contamination to train PPR-Meta, the performance of PPR-Meta may be reduced. Moreover, because there are seldom samples in which only chromosomes are enriched and all the extrachromosomal elements are filtered, we hard to collect negative samples to train PPR-Meta using real metagenomic data. Since no suitable real metagenome datasets with confident annotation are available as a benchmark, training PPR-Meta using artificial contigs extracted from sequenced complete genomes may be the best choice.

Actually, many bioinformatic tools to analyse metagenomic data were trained or tested using simulated data, such as tools for gene prediction, sequence binning (see: (1) Lu YY. et al. (2017). COCACOLA: binning metagenomic contigs using sequence COmposition, read CoverAge, CO-alignment and paired-end read LinkAge. *Bioinformatics.* (2) Alneberg J. et al. (2014). Binning metagenomic contigs by coverage and composition. *Nat. Methods.*), short read assembly (Reference: Namiki, T. et al. (2012). MetaVelvet: an extension of Velvet assembler to de novo metagenome assembly from short sequence reads. *Nucleic Acids Res.*) and host prediction for metagenomic phage contigs. In particular, all the four tools that were compared with PPR-Meta in the manuscript were also constructed primarily using sequenced genomes from the NCBI database rather than a known set of MGEs from a real metagenomic sample. Because of the high quality of the simulated data, constructing artificial contigs is one of the main approaches to developing bioinformatics tools for metagenomic analysis.

For the prophage identification, we agree with Reviewer 3 that there are thousands of annotated prophages and genomic islands. As we mentioned in the response to General Comment #2, we only used 267 manually annotated prophages because we found that most prophages from the current prophage database were predicted by the related software and had not been verified, while these 267 manually annotated prophages were of high quality and have widely been used as benchmarks for computational software. In the revised manuscript, we additionally collected another 139 manually verified prophages from the PHANTOME database to further support our conclusion. The result shown in Additional File 3, Figure S4, showed that PPR-Meta had the best ability to identify these prophages. Further, because the formation mechanism of certain genomic islands is related to phages or plasmids, we have used genomic islands from the Islander database to evaluate how PPR-Meta and related tools perform on these genomic islands. The results showed that PPR-Meta could identify more genomic islands as foreign DNA (either phages or plasmids) than other tools, indicating that PPR-Meta had a better ability to recognize sequences from regions of horizontal origin on bacterial chromosomes. For more details, please refer to our response to General Comment #2 as well as Line 5-15, Page 31, Section "Discussion and conclusions" of our revised manuscript.

*5. Finally, I would like to see the analysis of this software performed on a large metagenomic dataset. What percentage of the an ocean, fecal or soil sample is made up of phages/plasmids? Does the tool simply key in on odd codon/base pair coding, or is it keying in on larger gene-level issues? With a neural network, this is not always easy to tell, but could have fundamental impacts on how we view the metagenomics of mobile elements.*

We thank Reviewer 3 for this helpful advice on using PPR-Meta to analyse a large metagenomic dataset. **In the revised manuscript, we have employed PPR-Meta to identify phage and plasmid sequences on a series of metagenomic datasets of the human digestive tract from the Human Microbiome Project (HMP), including the gut, throat and oral cavity.** The results are interesting and may be significant to the study of human health. In the revised manuscript,

we have added a new subsection "Phages and plasmids in the human digestive tract", to present the results. (Please refer to Line 18, Page 24 - Line 21, Page 25 in the revised manuscript.)

The title and legend of Figure 3 are presented in Line 1, Page 26 as: "**Figure 3. Percentages of phages, chromosomes and plasmids in the human digestive tract.** PPR-Meta was used to predict the sequences of phages, chromosomes and plasmids in metagenomic assemblies, including samples from the gut, throat and oral cavity. The sequence percentages of phages, chromosomes and plasmids were calculated." The new citations have also been added to the list of the References, and the term HMP has also been add to the "List of abbreviations" section. (Please refer to Line 11, Page 36 in the revised manuscript.)

For the last point of this comment, we understand the consideration of Reviewer 3 about how PPR-Meta works. In our opinion, PPR-Meta primarily works by observing the sequence signatures such as odd codon/base pair coding, as Reviewer 3 mentioned, rather than by using information on larger gene-level issues. PPR-Meta is mainly designed for identifying phages or plasmids from metagenomic short fragments, and the results have shown that PPR-Meta can present satisfactory performance. In general, fragments shorter than 2k bp are hard to contain more than one gene or even a complete gene. If PPR-Meta works by using information on larger gene-level issues, it could not present satisfactory performance due to the insufficient number of genes for its analysis. As an example for comparison, the tool VirSorter, one of the software compared with PPR-Meta in the manuscript, is a gene-based tool, and it performs poorly. In our evaluation, VirSorter missed most phages for sequences shorter than 1.2k bp. Since VirSorter identifies phage sequences primarily by observing the distribution of genes, such as the densities of known viral genes or the enrichment of short genes, the lack of genes in short fragments may lead to the poor performance of VirSorter. Therefore, PPR-Meta does not seem to be a gene-based tool but a tool based on sequence patterns. In fact, it has been reported that plasmids and phages contain different sequence signatures from their host, such as the GC content, codon usage or k-mer frequencies, which make it possible to identify them through sequence signatures. In addition, the information of sequence signatures has also been widely used in many sequence classification software, such as the binning algorithm for metagenomic fragments. (see Alneberg et al. (2014). Binning metagenomic contigs by coverage and composition. *Nat. Methods.*). Overall, in our opinion, we consider that PPR-Meta makes the judgment primarily through sequence signatures rather than larger gene-level issues.

In hoping that the above revision has clarified all the points by three reviewers and given a point-by-point response to all the concerns, we hereby resubmit our manuscript to GigaScience. We thank you for your kind consideration.

Sincerely yours,
Huaiqiu Zhu, Ph. D., Professor
Peking University
