# Supplementary material for: PPR-Meta: a tool for identifying phages and plasmids from metagenomic fragments using deep learning
Source: Gigascience. 2019 Jun 20;8(6):giz066. doi: 10.1093/gigascience/giz066 (PMC6586199; doi:10.1093/gigascience/giz066)
Supplement: giz066_GIGA-D-18-00464_Revision_2 [file giz066_giga-d-18-00464_revision_2.pdf]

GigaScience

PPR-Meta: a tool for identifying phages and plasmids from metagenomic fragments using deep learning

--Manuscript Draft--

**Manuscript Number:**	GIGA-D-18-00464R2
**Full Title:**	PPR-Meta: a tool for identifying phages and plasmids from metagenomic fragments using deep learning
**Article Type:**	Technical Note
**Funding Information:**	National Natural Science Foundation of China (31671366) — Dr Huaiqiu Zhu<br>Ministry of Science and Technology of the People's Republic of China (2017YFC1200205) — Dr Huaiqiu Zhu<br>Peking University (None) — Dr Huaiqiu Zhu
**Abstract:**	Background: Phages and plasmids are the major components of mobile genetic elements, and fragments from such elements generally co-exist with chromosome-derived fragments in sequenced metagenomic data. However, there is a lack of efficient methods that can simultaneously identify phages and plasmids in metagenomic data, and the existing tools identifying either phages or plasmids have not yet presented satisfactory performances.<br>Findings: We present PPR-Meta, a three-class classifier that allows simultaneous identification of both phage and plasmid fragments from metagenomic assemblies. PPR-Meta consists of several modules for predicting sequences of different lengths. Using deep learning, a novel network architecture, referred to as the Bi-path Convolutional Neural Network, is designed to improve the performance for short fragments. PPR-Meta demonstrates much better performance than currently available similar tools individually for phage or plasmid identification, while testing on both artificial contigs and real metagenomic data. PPR-Meta is freely available via http://cqb.pku.edu.cn/ZhuLab/PPR_Meta or https://github.com/zhenchengfang/PPR-Meta.<br>Conclusions: To the best of our knowledge, PPR-Meta is the first tool that can simultaneously identify phage and plasmid fragments efficiently and reliably. The software is optimized and can be easily run on a local PC by non-computer professionals. We developed PPR-Meta to promote the research on mobile genetic elements and horizontal gene transfer.
**Corresponding Author:**	Huaiqiu Zhu<br><br>CHINA
**Corresponding Author Secondary Information:**	
**Corresponding Author's Institution:**	
**Corresponding Author's Secondary Institution:**	
**First Author:**	Zhencheng Fang
**First Author Secondary Information:**	
**Order of Authors:**	Zhencheng Fang<br>Jie Tan<br>Shufang Wu<br>Mo Li<br>Congmin Xu

Powered by Editorial Manager® and ProduXion Manager® from Aries Systems Corporation

	Zhongjie Xie
	Huaiqiu Zhu
**Order of Authors Secondary Information:**	
**Response to Reviewers:**	Cover Letter<br><br>Dear Editor,<br><br>Thank you very much for your previous E-mail on April 6, 2019 regarding our manuscript ''PPR-Meta: a tool for identifying phages and plasmids from metagenomic fragments using deep learning'' (Manuscript ID: GIGA-D-18-00464R1). We are very pleased to know that our manuscript is potentially acceptable for publication in the journal, subject to the suggested further revision following the reviewers. Herein we would like to thank three reviewers and are pleased to know that our previous responses were addressed all of their concerns. Additionally, we are grateful for their careful reading of the previous version of our manuscript, and the further comments raised by three reviewers helped us make further improvements. In the revised manuscript, all the changed words, sentences and paragraphs are marked in red text.<br><br>Following your instruction and Reviewers' comments, this time we have made a conscious effort to revise the manuscript with the further improvement. Before we report the revisions and responses to Reviewers' comments, we would like to first present our responses to Editor's instructions. With respect to the website, because the server is regularly maintained, users might be occasionally unable to access it when the server is under maintenance. Therefore we have double-checked our website (http://cqb.pku.edu.cn/ZhuLab/PPR_Meta/), and we confirm that the website is working. Additionally, our tool is stored on our website, as well as GitHub and the GigaScience database, and thus, users can also download the software if our website is under maintenance. The links to our website and the GitHub page are provided in Subsection "Availability of supporting source code and requirements". (Please refer to Lines 2-3, Page 35 in the revised manuscript.) Also, we have already registered PPR-Meta in the SciCrunch.org database, and the RRID (SCR_016915) is provided in Subsection "Availability of supporting source code and requirements". (Please refer to Line 11, Page 35 in the revised manuscript.) In addition, Reviewer 1 pointed out that the resolution of Figure 4 was poor. However, we had uploaded Figure 4 to the submission system as a high-resolution "jpg" file, but it appears that the resolution decreased when the "jpg" file was converted into a "pdf" file by the submission system. So, we would like to ask for your help to solve this problem when the manuscript is published. If the file needs to be reformatted, please kindly let us know.<br><br>We then report our revisions and responses to three reviewers' all comments (italic text) one by one as follows:<br><br>To Reviewer #1:<br><br>General Comments:<br>In the revised version of their manuscript, the Authors have adequately addressed all points that I had raised on the previous version. The manuscript is now clearer and easier to read. The change to the algorithm, mostly removal of FNN part makes the software and idea clearer. Moving in scan window across longer sequences is also a great idea and I see great possibilities with that approach for identification of prophages or chromosome-derived fragments on plasmids and phage genomes.<br>An interesting addition is also a part describing virome and plasmidome of the digestive tract, and such analysis can be further expanded into a separate manuscript.<br>We are glad to see that our previous responses were adequately addressed all the points raised by Reviewer 1. The positive comment "Moving in scan window across longer sequences is also a great idea and I see great possibilities with that approach for identification of prophages or chromosome-derived fragments on plasmids and phage genomes" is really encouraging. Additionally, the further comments raised by Reviewer 1 have helped us further improve the quality of the manuscript. We would like to greatly thank Reviewer 1 for his/her careful reading of our manuscript. Below, we itemize our revisions in response to Reviewer 1's points.

Powered by Editorial Manager® and ProduXion Manager® from Aries Systems Corporation

	1. On page. 33, line 15: should be Error Rate at Read Start.<br>We thank Reviewer 1 for noting this spelling mistake. The phrase “Error Rate at Read Star” has been revised to “Error Rate at Read Start”. (Please refer to the Section “Methods”, Line 17, Page 33 in the revised manuscript.)<br><br>2. I think Figure 4 should be presented in better resolution.<br>Herein we apologize for the low resolution of Figure 4. We had uploaded Figure 4 to the submission system as a high-resolution “jpg” file, but it appears that the resolution decreased when the “jpg” file was converted into a “pdf” file by the submission system. We believe that Editor can help us solve this problem when the manuscript is published.<br><br>3. Please provide versions of software used, if possible (missing for most of the programs).<br>We thank Reviewer 1 for reminding us to add the versions of the software programs used in the manuscript. In the revised manuscript, all of this information has been provided now. Specifically, in Subsection “Dataset construction”, Line 17, Page 7, we have added the version of ProphET to the sentence: “Here, we used ProphET (v0.5.1) to extract prophages from all the prokaryote chromosomes…”; in Subsection “Dataset construction”, Line 7, Page 8, we have added the version of MetaSim to the sentence: “We used the MetaSim (v0.9.1) simulator to extract artificial contigs from the complete genomes”; in Subsection “Dataset construction”, Line 18, Page 8, we have added the version of SPAdes to the sentence: “…which were downloaded from MG-RAST (Accessions: mgm4534202.3 and mgm4534203.3) as raw reads and assembled by SPAdes (v3.11.1)”; and in Subsection “Performance comparison”, Lines 18-19, Page 14, we have added the versions of VirFinder, VirSorter, PlasFlow and cBar to the sentence: “We then compare PPR-Meta with VirFinder (v1.1) and VirSorter (v1.0.3) regarding the ability to identify phages, and with PlasFlow (v1.1) and cBar (v1.2) regarding the ability to identify plasmids”.<br><br>4. Unfortunately, I can't agree with the statement: “Recently, many basecalling tools for the third-generation sequencing technology have been developed to help improve the accuracy over 99% [41], therefore the extremely high error rate on the raw data will not affect the usage of PPR-Meta" (page 19). Authors correctly refer to work by Wick et al, recently published as a preprint (https://doi.org/10.1101/543439), but accuracy over 99% is not a raw read accuracy, but rather consensus accuracy, where reads are first assembled into contigs, then polished using dedicated software. This sentence should be changed to something like (this is only the suggestion): Recently, many dedicated tools have been developed to help improve the consensus accuracy for the third-generation sequencing technology over 99% [41], therefore the extremely high error rate on the raw data should not affect the usage of PPR-Meta on assembled 3rd generation sequences.<br>We are grateful to Reviewer 1 for noting this incorrect statement, which helped us greatly improve the rigour of the manuscript. We have revised this statement according to the suggestion of Reviewer 1 as follows: “Recently, many dedicated tools have been developed to help improve the consensus accuracy for the third-generation sequencing technology over 99%, therefore the extremely high error rate on the raw data should not affect the usage of PPR-Meta on assembled 3rd generation sequences.” (Please refer to Subsection “Performance in the presence of sequencing errors”, Lines 13-17, Page 19, in the revised manuscript.)<br><br>5. In the case of the sentence: "PPR-Meta can also handle data from the third-generation sequencing technology, although it is designed primarily for the next-generation sequencing technology" (page 24): Isn't 3rd generation also the next-generation? Authors should consider changing “next-generation" to “2nd generation" or 3rd generation to single-molecule<br>We appreciate Reviewer 1 for noting that we have confused certain definitions related to the sequencing technology. Following the suggestion of Reviewer 1, this sentence has been revised as follows: “Therefore, PPR-Meta can also handle data from the third-generation sequencing technology, although it is designed primarily for the second-generation sequencing technology.” (Please refer to Subsection “Evaluation in real metagenomic data”, Line 17, Page 24, in the revised manuscript.)<br><br>6. The sentence "In the other hand, in order to identify sequences from low-abundance

Powered by Editorial Manager® and ProduXion Manager® from Aries Systems Corporation

phages, which may fall into binning, we also need tools that can directly judge each fragment." (page 5) needs rephrasing.
Herein, we thank Reviewer 1 for reminding us to rephrase the sentence in a more appropriate way. To clarify the sentence, we have revised it as follows: “In the other hand, in order to identify sequences from low-abundance phages, which may not fall into bins, tools that can directly judge each fragment are also needed.” (Please refer to Section “Introduction”, Line 14-16, Page 5 in the revised manuscript.) We confirm that “not fall into bins” is the meaning that we want to express and we apologize for missing an important word in this sentence.

To Reviewer #2:

We are pleased to hear that we have properly addressed all of the concerns raised by Reviewer 2. We especially thank Reviewer 2 for the extremely careful reading of our manuscript, and the new comments raised by Reviewer 2 are worthy of attention. Our responses to Reviewer 2’s comments are as follows.

General Comments:
1. Regarding this statement in authors' response and similar statements in the manuscript (pg 24 and 25): "We found that in the position closer to the outer end of the digestive tract, the percentages of phages and plasmids tended to be higher. For example, in the gut, the inner end of the digestive tract, the percentages of phages and plasmids were lower; in the oral cavity, the outer end of the digestive tract, the percentages of phages and plasmids were higher."
It seems that authors' conclusion that "percentage of phage and plasmids are higher in the oral cavity" is only based in counts and proportions of contigs found in each one of these environments. However, abundance estimates would be more appropriate if based on raw reads counts. There are several factors which may affect assembly efficiency, and some (if not most) are not dependent on the total abundance of an organism in the sample. Presence of repeats are one good example which may prevent assembly. All of that said to suggest an approach like mapping raw reads to contigs predicted as phages and plasmids and calculating proportions of the total. Or counts based in total number of bases instead of absolute sequence counts.
Herein, we understand the consideration of Reviewer 2 that estimating the abundance by contig counts may has bias. Indeed, in addition to organism abundance, other factors might also affect the assembly efficiency. When the assembly performance is poor, there may be a large number of short fragments, and therefore, the higher contig percentage of phages and plasmids in the throat and oral cavity might result from the poor assembly rather than the higher abundance of phages or plasmids. To test whether the higher contig percentage of phages and plasmids in the throat and oral cavity results from a poor assembly, we calculated the average length of phage and plasmid contigs predicted by PPR-Meta in each sample from the gut, throat and oral cavity. The results show that the average length of phage contigs from the gut, throat and oral cavity was 732.4, 701.1 and 747.0 bp, respectively, and the average length of plasmid contigs from the gut, throat and oral cavity was 838.7, 959.6 and 962.4 bp, respectively. As shown, the average length of phage and plasmid contigs in the oral cavity was not shorter than that in the gut and throat, which indicated that the sequence assembly performance was comparable among the gut, throat and oral cavity samples. These results also show that the higher percentage of phage and plasmid contigs in the outer end of the digestive tract was likely caused by the higher abundance of phages and plasmids rather than a poor sequence assembly.
Furthermore, the results mentioned above also show that in the outer end of the digestive tract, the average lengths of phage and plasmid contigs were generally longer than those in the inner end, and these results were particularly notable for plasmid contigs. This finding further supports our statement that “in the position closer to the outer end of the digestive tract, the percentage of phages and plasmids tended to be higher” because reads from highly abundant organisms are more easily assembled into long contigs. Moreover, all the data from the Human Microbiome Project (HMP) were generated using a similar protocol, such as a similar sequencing depth, which had significant influences on the sequence assembly; thus, the quality of the data from different samples might be similar. We believe that this uniformity can also ensure the reliability of our analysis and conclusion.
Of course, if we want to learn more about the functions of phages and plasmids in the human digestive tract, we need more detailed analyses that can be expanded into

future work. In the current study, the main purpose of the analysis was to illustrate how PPR-Meta can promote research on phages and plasmids.

2. I tried to access the website provided in the manuscript and it did not work for me. Please, be careful about links: http://cqb.pku.edu.cn/ZhuLab/PPR_Meta/
We thank Reviewer 2 for visiting the website of our tool. We would like to apologize for that the website could not be accessed then, which might be the server maintenance at that time. We have double-checked the website and will keep trying our best to make it accessible. Besides, our tool is stored on our website, GitHub and the GigaScience database, and thus, users can also download the software if our website is under maintenance. The links to our website and GitHub page are provided in the Subsection "Availability of supporting data". (Please refer to Lines 2-3, Page 35 in the revised manuscript.)

3. I respectfully disagree with review #3 regarding virtual machines being an "odd choice and not at all standard in bioinformatics". I would say that this kind of approach has become more and more common in bioinformatics, specially for very complex software that require lots of dependencies. I work with keras and Tensor Flow, and as a matter of fact they are not easy to set up libraries. Virtual machines or docker containers are very practical way of running tools by people without specialized knowledge. Moreover, Virsorter (which is one of the tools that predict phage and prophages in contigs) also has a docker container, which is very useful. Nonetheless, I also appreciate the gold standard github availability, and that authors decreased VM size.
Herein we thank Reviewer 2 for this helpful discussion about how to release a bioinformatic tool. Our PPR-Meta can run on both a physical host and a virtual machine. The advantage of running PPR-Meta on virtual machine is that the virtual machine is very easy to install by non-computer professionals because it does not require the installation of any dependent packages. The PPR-Meta manual provides detailed explanation on how both versions of the tool can be run. Therefore, users can conveniently choose which version to use according to their situation.

To Reviewer #3:

We are very pleased to note that Reviewer 3 recommends that this paper be accepted for publication. We think that the revisions made according to Reviewer 3's previous comments have made the manuscript more convincing. Below, we provide our responses to Reviewer 3's further comments.

General Comments:
1. I brought up that I thought the files should be distributed over a more conventional means, like GitHub. I still have concerns about the requirement to download an entire VM. However, given that the journal's author guidelines specify allowing a virtual machine, I think the authors have acted in the best possible way. I appreciate the authors willingness to further compress the download, from 30 to 2.5 Gb in size. While the program is still quite large, it is much less unwieldy.
Herein we understand the concern of Reviewer 3 regarding the downloading of an entire VM. Our PPR-Meta can run on both a physical host and a virtual machine. The advantage of running PPR-Meta on virtual machine is that the virtual machine is very easy to install by non-computer professionals because it does not require the installation of any dependent packages. If users choose to run PPR-Meta on a physical host, they do not need to download the virtual machine; instead, they only need to download a small package from our website or GitHub. The PPR-Meta manual provides detailed explanations on running both versions of the tool. Therefore, users can conveniently select which version to use according to their own situation.
With respect to the size of the virtual machine, it appears that 2.5 GB is the minimum size that we can achieve, and we apologize for being unable to further reduce the size. Although downloading the virtual machine might take some time, running PPR-Meta on a virtual machine might still be the best approach for non-computer professionals because it can save more time when installing the tool.

2. In my comments, I addressed issues with the detection of phage vs. prophage vs. plasmid - in particular the reliance on phage and plasmid databases and the lack of experimentally verified data, particularly, the lack of genomic islands. The authors

Powered by Editorial Manager® and ProduXion Manager® from Aries Systems Corporation

	updated their analysis, including more phage/plasmid manually curated data, and data on genomic islands. They also showed the performance of these in comparison. This reanalysis and discussion assuages my concerns about the limitations in the analysis.<br>We are glad to see that our new analysis and discussion have been addressed Reviewer 3's concerns. We believe that this analysis indicates that PPR-Meta can also identify genomic islands, which might be caused by phages or plasmids, and this information makes our manuscript more convincing.<br><br>3. The justification for Figure 1 and the elimination of the original Figure 2 sufficiently addressed my concerns that the motivation and description of the FNN were insufficient. I appreciate that the authors show all the changes that resulted from these changes.<br>Again, we are pleased to hear that our new description sufficiently addressed Reviewer 3's concerns regarding the motivation of the design of the neural network. Additionally, we believe that the new description provides insights into the neural network design strategy for readers.<br><br>4. Many of the issues from this comment were taken into account in comment #2 and I appreciate the full description in the authors' responses.<br>We thank Reviewer 3 for this comment from the last report, and we are happy to note that the issues raised in this comment have been addressed.<br><br>5. The authors went above-and-beyond what was required by including the HMP data. This is a major improvement to the generalizability of the analysis. I still believe that the second part hasn't been sufficiently addressed in the manuscript, but has in the comments. Having a robust justification for why the authors believe PPR-Meta is keying on sequence signatures and what the implications of that are, may warrant subsequent work.<br>Herein, we are grateful for the positive comment provided by Reviewer 3 noting that the identification of phage and plasmid sequences from HMP data is a major improvement to the generalizability of the analysis. Additionally, we agree with Reviewer 3 that subsequent work is needed to prove why PPR-Meta is keying on sequence signatures. In the previous response, we explained this issue intuitively, primarily by comparing the performances of PPR-Meta and VirSorter, which is not a sequence-signatures-based tool. Related statements about tool keying on larger gene-level issues may not work well in metagenomic fragments were also emphasized in our manuscript. For example, in Section "Discussion and conclusions", Line 20, Page 27 to Line 1, Page 28, we mentioned that "Similarity search-based tools, such as VirSorter, provide good results for long sequences. However, such methods do not work effectively for short fragments due to the insufficient number of genes for the statistical analysis." Although we cannot provide a more quantitative interpretation at this time, we would like to state that neural networks have been widely used to analyse biological sequence and that neural networks have shown a strong ability to extract sequence patterns or sequence motifs (for more details, see the following reviews: [1] Jones W. et al. (2017). Computational biology: deep learning. Emerging Top. Life Sci. [2] Min S. et al. (2017). Deep learning in bioinformatics. Briefings Bioinf.). Therefore, we think that it is not strange that PPR-Meta can bypass the genetic information of larger gene-level issues and directly identify phage and plasmid sequences by observing the sequence signatures.<br><br>In hoping that the above revision has clarified all the points by three reviewers and given a point-by-point response to all the concerns, we hereby resubmit our manuscript to the journal. We thank you for your kind consideration.<br><br>Sincerely yours,<br>Huaiqiu Zhu, Ph. D., Professor<br>Peking University
**Additional Information:**	
**Question**	**Response**
Are you submitting this manuscript to a special series or article collection?	No

**Experimental design and statistics**<br><br>Full details of the experimental design and statistical methods used should be given in the Methods section, as detailed in our Minimum Standards Reporting Checklist. Information essential to interpreting the data presented should be made available in the figure legends.<br><br>Have you included all the information requested in your manuscript?	Yes
**Resources**<br><br>A description of all resources used, including antibodies, cell lines, animals and software tools, with enough information to allow them to be uniquely identified, should be included in the Methods section. Authors are strongly encouraged to cite Research Resource Identifiers (RRIDs) for antibodies, model organisms and tools, where possible.<br><br>Have you included the information requested as detailed in our Minimum Standards Reporting Checklist?	Yes
**Availability of data and materials**<br><br>All datasets and code on which the conclusions of the paper rely must be either included in your submission or deposited in publicly available repositories (where available and ethically appropriate), referencing such data using a unique identifier in the references and in the "Availability of Data and Materials" section of your manuscript.<br><br>Have you have met the above requirement as detailed in our Minimum Standards Reporting Checklist?	Yes

Manuscript R2 with DOI

Click here to access/download;Manuscript;Revision version 2 WITH DOI.docx

Click here to view linked References

PPR-Meta: a tool for identifying phages and plasmids from metagenomic fragments using deep learning

Zhencheng Fang[1,2], Jie Tan[1,2], Shufang Wu[1,2], Mo Li[1,2,3], Congmin Xu[1,2,4], Zhongjie Xie[1,2] and Huaiqiu Zhu[1,2]*

[1] State Key Laboratory for Turbulence and Complex Systems and Department of Biomedical Engineering, College of Engineering, Peking University, Beijing 100871, China

[2] Center for Quantitative Biology, Peking University, Beijing 100871, China

[3] Peking University-Tsinghua University - National Institute of Biological Sciences (PTN) joint PhD program, School of Life Sciences, Peking University, Beijing 100871, China

[4] Department of Biomedical Engineering, Georgia Institute of Technology and Emory University, Georgia 30332, USA

* To whom correspondence should be addressed.

hqzhu@pku.edu.cn

Abstract

Background: Phages and plasmids are the major components of mobile genetic elements, and fragments from such elements generally co-exist with chromosome-derived fragments in sequenced metagenomic data. However, there is a lack of efficient methods that can simultaneously identify phages and plasmids in metagenomic data, and the existing tools identifying either phages or plasmids have not yet presented satisfactory performances.

Findings: We present PPR-Meta, a three-class classifier that allows simultaneous identification of both phage and plasmid fragments from metagenomic assemblies. PPR-Meta consists of several modules for predicting sequences of different lengths. Using deep learning, a novel network architecture, referred to as the Bi-path Convolutional Neural Network, is designed to improve the performance for short fragments. PPR-Meta demonstrates much better performance than currently available similar tools individually for phage or plasmid identification, while testing on both artificial contigs and real metagenomic data. PPR-Meta is freely available via http://cqb.pku.edu.cn/ZhuLab/PPR_Meta or https://github.com/zhenchengfang/PPR-Meta.

Conclusions: To the best of our knowledge, PPR-Meta is the first tool that can simultaneously identify phage and plasmid fragments efficiently and reliably. The software is optimized and can be easily run on a local PC by non-computer professionals. We developed PPR-Meta to promote the research on mobile

genetic elements and horizontal gene transfer.

Keywords: metagenome, mobile genetic elements, horizontal gene transfer, phage, plasmid, deep learning

Findings

Introduction

Phages and plasmids, known as mobile genetic elements (MGEs), are the main participants in horizontal gene transfer (HGT) along with genetic information exchanging among prokaryotes or eukaryotes [1]. Such elements can regulate the microbial community by interacting with the host. One of the important roles of MGEs is their ability to distribute resistance genes among bacteria and facilitate environmental adaptations among microbial communities [2]. In most cases, a substantial number of phage and plasmid genomes are present in the microbial community. For example, reports have shown that the abundance of marine phages even surpasses that of other organisms in marine systems, and more than half of the bacteria isolated from marine systems contain at least one plasmid [3][4]. Thus, the identification of phage and plasmid fragments in metagenomes is a fundamental issue in comprehensive analyses of HGT and the interaction between MGEs and hosts. Although experimental approaches have been developed to enrich phages or plasmids from environment samples [5][6], the enriched samples lose host information, which may hinder the

comprehensiveness of the analyses. Therefore, computational tools for directly identifying phages and plasmids from metagenomes are expected to be developed in the field.

However, the effective identification of such elements remains a considerable challenge. Currently the fragments assembly performance of both plasmid and phage from high-throughput sequencing data is not as well as that of host-derived fragments [7]. This indicates that sequences from phages or plasmids exist as a large number of short fragments, resulting in the difficulty of the identification. In addition, fewer sequenced genomes of phages and plasmids are available compared with bacterial genomes in current databases [1]. Especially, although the abundance of viruses is estimated to exceed that of other organisms on the earth [8], so far the number of phage genomes in the NCBI database is still less than one-thirtieth the number of prokaryotic genomes, and it was estimated that more than half of the sequences from viral metagenomes could not find significant homology to the released database [5]. Therefore, it is especially essential to develop a tool for identifying novel phages and plasmids from metagenomic data with a large number of mixed short reads.

Despite the difficulty of identification, several tools have been recently developed to detect either phages or plasmids from culture-dependent Whole Genome Sequencing (WGS) data or metagenomic data. Tools that detect regions from an integrated phage sequence (referred to as prophage) over a sequenced complete bacterial genome have been designed. These tools

include Prophinder [9], Phage_Finder [10], PhiSpy [11], PHAST (and its enhanced version PHASTER) [12] [13], VirSorter [14], and ProphET [15]. Such approaches primarily used a scan window to move across the complete bacterial chromosome and extract regions that seem to be phages based on a similarity search against viral databases. Because the scan windows of these tools are often required to be able to cover several genes, such tools are difficult to apply to metagenomic data since the sequences of metagenome are too short to contain even a complete gene [16]. Although VirSorter can also assign metagenomic contigs as phages or bacteria, its sensitivity of identification is quite low. Moreover, lytic phages and some temperate phages do not integrate their genomes into their host chromosomes [17], thus these tools may only be able to identify specific phages. The tool MARVEL [18] can assign metagenomic bins as phages or bacteria and demonstrates better performance than previous tools. In the other hand, in order to identify sequences from low-abundance phages, which may not fall into bins, tools that can directly judge each fragment are also needed. In contrast, VirFinder [19] can directly judge each sequence, and it uses a logistic regression as the classifier to detect phage sequences based on *k*-mer frequencies and presents a relatively good performance. In terms of plasmids, most of the current tools for plasmid identification were designed for WGS or even specific species, such as PlasmidFinder [20], PLACNET [21], PlasmidSeeker [22] and mlplasmids [23]. However, the plasmid identification strategy for WGS may not by applicable for

metagenomes. For example, PlasmidSeeker considers plasmid contigs to have a higher read coverage because plasmids may have copies in their hosts. In metagenome, however, the difference of read coverage among contigs may result from different abundances of species rather than copy number. The tool cBar [24] is the first tool designed primarily for plasmid identification in metagenomes. This tool applies sequential minimal optimization (SMO) as a classifier based on *k*-mer frequencies. Similar to cBar, PlasFlow [25] is also a *k*-mer-based tool for identifying plasmids. Compared with cBar, PlasFlow further combines the information of different *k*-mer lengths and uses multiple neural networks as voting devices to determine whether the sequence belongs to the plasmid, and it achieves a better performance than cBar.

Although related tools have been developed, state-of-the-art tools for detecting short fragments have not presented satisfactory performances. Moreover, because these tools can only identify either phages or plasmids, they clearly do not meet the needs of a comprehensive analysis of MGEs and HGT. Considering that poor sequence assembly performance results in a large number of short fragments, it is a practical goal to develop a higher performing tool. In this paper, we present the PPR-Meta (**P**hage and **P**lasmid **R**ecognizer for **Meta**genomes), a three-class classifier for identifying metagenomic fragments as phages, plasmids or chromosomes based on the deep learning algorithm. To achieve higher performance on short fragments, we designed a novel neural network architecture which is referred to as the Bi-path

Convolutional Neural Network (BiPathCNN). To the best of our knowledge, PPR-Meta is the first tool that can simultaneously identify phage and plasmid fragments efficiently and reliably.

Dataset construction

Owing to that no suitable real metagenome datasets with confident annotation are available as a benchmark, we therefore used the simulated datasets with artificial contigs generated from sequenced complete genomes. We downloaded the complete genomes of prokaryote chromosomes (total of 10,090 genomes), prokaryote plasmids (total of 8,801 genomes) and phages (total of 2,279 genomes) from the NCBI genome database [26]. The list of the genomes is provided in Additional file 1. To evaluate the ability of PPR-Meta to identify novel species, genomes released before January 2016 were employed to build the training set while the remainder used to build the test set. In general, prokaryote chromosomes may contain regions of integrated phages, referred to as prophages [27], however most genomes do not have the prophage annotation. Here, we used ProphET (v0.5.1) to extract prophages from all the prokaryote chromosomes, and a total of 16,393 prophages predicted by ProphET (shown in Additional file 2) were incorporated into the phage dataset. Moving prophages from a chromosome dataset to a phage dataset can help to both expand the phage dataset and remove noise from the chromosome dataset. Since the predicted prophages were generated by ProphET and could

not be used as a benchmark, we removed the predicted prophages from the test set. To evaluate the performance of PPR-Meta for prophage identification, we collected 267 manually annotated prophages of 54 prokaryote chromosomes from Casjens [27]. To ensure that the test data were “novel” to PPR-Meta, these prophages and their hosts were removed from the training set.

We used the MetaSim (v0.9.1) simulator [28] to extract artificial contigs from the complete genomes. Four groups of artificial contigs of different lengths were generated: Group A with a length range of 100-400 bp, Group B with a length range of 400-800 bp, Group C with a length range of 800-1200 bp and Group D with a length range of 5000-10000 bp. Group A, B and C were constructed to simulate the length obtained with different sequencing technology and the average assembly contig length, while Group D was constructed to simulate long contigs in metagenomic data.

We also used real metagenomic data to estimate the reliability of PPR-Meta. The real data included phage metagenomic data of bovine rumen [29], which were downloaded from MG-RAST [30] (Accessions: mgm4534202.3 and mgm4534203.3) as raw reads and assembled by SPAdes (v3.11.1) [31]; plasmid metagenomic data of bovine rumen [32], downloaded from MG-RAST (accessions: mgm4460391.3); and 20 samples of healthy human gut [33], downloaded from the NCBI Short Read Archive [34] and assembled by SPAdes. The accessions of the human gut samples are shown in Additional file 1.

Additional details on the dataset construction are provided in Methods section.

Mathematical model of DNA sequences

The method of representing biological sequence is significant for every machine learning-based tool. Although *k*-mer frequencies have been widely used in many studies [19], such frequencies may present serious fluctuations in short sequences [35]. Here, we use a more detailed approach to represent the DNA fragments. Specifically, each sequence is represented by “base one-hot matrix (BOH)” and “codon one-hot matrix (COH)”. “One-hot” is one of the most widely used encoding forms for each character in a given string in the field of Natural Language Processing (NLP) [36], and it is also used to represent bases or amino acids in biological sequences. A “one-hot” vector contains several bits, and the number of bits is equal to the number of character types in a given string. For each character type, the corresponding bit of the “one-hot” vector is 1 and the remaining bits are 0, and there must be a one-to-one correspondence between each character type and each bit. For BOH in PPR-Meta, bases A, C, G and T are represented by [0,0,0,1], [0,0,1,0], [0,1,0,0], and [1,0,0,0], respectively. Therefore, together with the complementary strand, a sequence of length L can be represented by a BOH matrix of length 2×L and width 4. For COH, each sequence is first expanded to six phases in the form of codons. For example, sequence 5’-ACGTTCGAACG-3’ will be split into the following six codon sequences:

1): ACG, TTC, GAA

2): CGT, TCG, AAC

3): GTT, CGA, ACG

4): CGT, TCG, AAC

5): GTT, CGA, ACG

6): TTC, GAA, CGT

Similar to BOH, each codon of COH is represented by a 64 dimensional one-hot vector, namely one certain position is 1 and the other positions are 0. Therefore, a sequence of length L can be represented by a COH matrix of length $2\times L$ and width 64. Both BOH and COH will be used as input for the neural networks mentioned below.

Structure of deep learning neural networks

To ensure that PPR-Meta optimally adapts to sequences of different lengths, we trained three neural networks for Group A, B and C. To improve the performance, we designed BiPathCNN (Figure 1), a novel neural network structure, to make reliable predictions. BiPathCNN contains a “base path” and a “codon path”, which take BOH and COH as inputs respectively. After multiple convolution operations, the data for the two paths are combined by a merge layer. The fully connected layers then receive the merged data and finally output three scores that reflect the likelihood of the input fragment as a phage, chromosome or plasmid.

Figure 1. Structure of BiPathCNN. Three BiPathCNNs were trained for sequences from Group A, B and C. Each BiPathCNN contains a “base path” and a “codon path”, which take BOH and COH as inputs respectively.

The details of each layer are described as follows.

Layer b1 and layer c1: one-dimensional convolutional layers with 64 convolution kernels using “ReLU” (Rectified Linear Unit) as the activation function. The ReLU function can be expressed as y=max(0,x). These layers take BOH or COH as inputs. The length of the convolution kernels is set to 6.

Layer b2 and layer c2: max pooling layers with a pooling length set to 3.

Layer b3 and layer c3: batch normalization layers with the dropout operation. Each element of the feature map from previous layer in each batch will be normalized, which can speed up the convergence and prevent overfitting.

Layer b4~b6 and layer c4~c6: similar to layers b1~b3 or layers c1~c3, respectively. We set the number of convolution kernels in layer b4 and c4 as 128 and the length of the kernels as 3.

Layer b7 and layer c7: one-dimensional convolutional layers containing 256 convolution kernels and using ReLU as the activation function. The length of the convolution kernels is set to 3.

Layer b8 and layer c8: one-dimensional global average pooling layers that output the global average for each feature map of the previous layer.

Layer 9 to layer 11: The concatenation layers combine the output of the "base path" and "codon path". After the full connection layer with the same number of nodes as the previous layer, the softmax layer calculates the probability of the input fragment as a phage, chromosome or plasmid.

The selection of the related hyperparameters of each path mentioned above was referred to LeNet-5 [37] and VGG [38], two classic Convolutional Neural Networks in the field of artificial intelligence. Specifically, the distribution of layers was referred to LeNet-5, which contained three convolution layers, and there was a pooling layer between every two convolution layers. Meanwhile, the distribution of the number of convolution kernels was referred to VGG, in which the number of convolution kernels in the different layers was increased by doubling. We also referred to VGG to use ReLU as the activation function. All the neural networks used Adam as the optimizer and cross-entropy as the loss function.

In practical applications, PPR-Meta uses BiPathCNN A to predict sequences between 100 and 400 bp, BiPathCNN B to predict sequences between 400 and 800 bp, and BiPathCNN C to predict sequences between 800 and 1200 bp. For sequences longer than 1200 bp, such as sequences in Group D, a scan window will move across the sequence without overlapping, and the weighted average of all windows' predictions is calculated. The length of the window is set to 1200 bp (or less if the window ends beyond the sequence boundary). For example, given a sequence of length 2500 bp, the scan window

will first cover the bases from the 1st to 1200th positions, then the window will move to bases from the 1201st to 2400th positions, and finally, the window will move to bases from the 2401st to 2500th positions. Then, PPR-Meta uses BiPathCNN C, BiPathCNN C and BiPathCNN A to predict the subsequences under the first, second and third windows, respectively. To generate the final score for the whole sequence, PPR-Meta calculates the weighted average of these windows. The weights of these three windows are 1200/2500, 1200/2500 and 100/2500, respectively.

Overall performance

We evaluated PPR-Meta according to four groups of test sets with different lengths of short contigs. For each fragment input, the algorithm calculates three scores representing the likelihood that the fragment should be identified as a phage, plasmid or chromosome. Therefore, the category with the highest score is selected as our prediction. We used three-class confusion matrices (shown in Figure 2) to evaluate the overall performance of PPR-Meta. In general, PPR-Meta had a better discrimination ability when the sequences were longer, and the phage recognition ability of PPR-Meta was better than the plasmid recognition ability. Plasmid sequences were easily confused with the host chromosomes, which may because phages and plasmids face different evolutionary pressures. Since plasmid must survive in host cells, they may adapt their sequence signatures, such as the GC content and codon usage, to

their hosts. In contrast, phages can assemble their own particles and remain outside of the hosts. Moreover, certain phages may contain their own tRNA, which allows them to change their codon usage [39]. Thus, the various similarity of phages and plasmids to their hosts may lead to differences in the identification ability of PPR-Meta. In addition, transposons may carry plasmid DNA fragments to the chromosome [1]. Therefore, the chromosome may contain regions from the plasmid. Sequences shared between the plasmid and the chromosome may also affect the judgment of PPR-Meta. Overall, PPR-Meta can effectively identify the MGEs in the test set.

Figure 2. Confusion matrix of PPR-Meta. Three-class confusion matrices were used to evaluate the overall performance of PPR-Meta. Four matrices correspond to the sequences of Group A to D. In each matrix, the rows represent the true category while the column represent the predicted category of PPR-Meta.

Performance comparison

We then compare PPR-Meta with VirFinder (v1.1) and VirSorter (v1.0.3) regarding the ability to identify phages, and with PlasFlow (v1.1) and cBar (v1.2) regarding the ability to identify plasmids. The evaluation criteria were the true positive rate (TPR=TP/(TP+FN)), false positive rate (FPR=FP/(TN+FP)) and area under the curve (AUC). Note that PlasFlow will filter uncertain predictions

according to a default threshold. As a uniform comparison, we turned off this feature by setting the threshold to zero, thus using all the sequences for comparison.

The results are shown in Table 1. In all cases, the AUCs of PPR-Meta were the highest. In terms of phages, VirSorter, which is a gene-based tool, performed poorly with almost all phages missed. It is probably because there is not a sufficient number of full-length genes present in short DNA fragments for VirSorter's analysis. This also indicates that methods based on homology searches of genetic information are not applicable to species identification in metagenomes. Considering that most contigs of metagenomes are short fragments especially those of MGEs, VirSorter is not competent for phage identification despite achieving a higher performance for long contigs in Group D. The tool VirFinder outperformed VirSorter. As an alignment-free tool, VirFinder achieved a much higher TPR, especially in short fragments. The TPR of PPR-Meta was approximately 10% higher than that of VirFinder and the FPR was approximately 5~10% lower. The performance improvement on short sequences demonstrates that our sequences representation method is more detailed than the *k*-mer frequencies, and the deep learning algorithm is more capable of extracting sequence features than the logistic regression used by VirFinder. In terms of plasmids, both cBar and PlasFlow did not perform well. The cBar appeared to produce random results in most cases, with both the TPR and FPR near 50%. Although the AUC of PlasFlow was slightly higher than that

of cBar, PlasFlow tended to judge most sequences as plasmids, which resulted in an extremely high FPR. For PPR-Meta, the TPR was comparable with that of PlasFlow, while the FPR was approximately 25~40% lower. In some cases, a few assembled sequences from high-abundance species may be much longer, so we also tested PPR-Meta and the related tools using 15k bp and 30k bp fragments (shown in Additional File 3, Figure S1). The results showed that the performance of PPR-Meta was still the best for these long sequences. In addition, we tested the accuracy as well as the running time of each BiPathCNN on test datasets from different groups and found that using a non-corresponding BiPathCNN to predict sequences from specific groups would lead to a lower accuracy and longer running time (shown in Additional File 3, Figure S2). Overall, PPR-Meta presented a much better performance than other homology-search-based tools such as VirSorter and k-mer-based tools such as VirFinder, PlasFlow and cBar.

Table 1. Evaluation of the performance of PPR-Meta and comparison of the performance of PPR-Meta and related tools.

Effectiveness of BiPathCNN

PPR-Meta achieved a much higher performance than the other methods as mentioned above. The innovation of PPR-Meta is the design of BiPathCNN, which uses both base and codon information to improve the performance. In

BiPathCNN, the “base path” is beneficial to extracting the sequence features of non-coding regions while the “codon path” is beneficial to extracting coding regions. To verify the effectiveness of BiPathCNN, we removed the “codon path” and “base path” and retrained PPR-Meta. The newly trained PPR-Meta was tested, and the results showed that the performance of PPR-Meta with either the “base path” or “codon path” only presented a lower performance relative to that of BiPathCNN in most cases (Table 2). Moreover, the performance of the “codon path” only CNN was better than that of “base path” only CNN, which indicates that the features that distinguish phages, chromosomes and plasmids are more concentrated in the coding region. Compared with other sequence representation methods that ignore the coding or non-coding region, such as method based on *k*-mer frequencies, PPR-Meta uses a more detailed method of describing a sequence and achieves a higher performance.

Table 2. Performance comparison among BiPathCNN, the base path-only CNN and codon path-only CNN.

Performance in the presence of sequencing errors

Sequencing errors exist in various sequencing technologies, and tools that handle high-throughput sequencing data should be able to tolerate these errors. In addition, the third-generation sequencing technology, such as PacBio and Nanopore, have much higher sequencing errors. Thus, the compatibility of tools

with new sequencing technologies should be considered.

Sequencing errors can be divided into two types: base substitutions and base insertions or deletions. We tested the impact of these two types of sequencing errors on the identification performance of PPR-Meta and related tools. We used MetaSim to extract modified fragments with 1% substitutions and 1% insertions or deletions separately from the test genomes. We used the same criteria described above to compare the performance of different tools in terms of both types of error. The results are shown in Table 3 and Table 4.

In most cases, in the presence of 1% base substitutions, a slight decrease in each evaluation criterion was observed for each tool compared with that in the presence of non-sequencing errors, although the decrease was not obvious. PPR-Meta was still the best-performing tool. When 1% of the bases were inserted or deleted, the performance of most tools was slightly reduced with the exception of VirSorter. Base insertions or deletions caused significant fluctuations in the performance of VirSorter. For sequences of Group D, the AUC of VirSorter decreased by approximately 9% compared with sequences with no errors. In our opinion, the reason that VirSorter exhibits great fluctuations in performance with base insertions or deletions is that insertions and deletions disrupt the phase of the open reading frame (ORF). VirSorter identifies phage sequences primarily by observing the distribution of genes, such as the densities of known viral genes or the enrichment of short genes. Disrupting the ORF phase will severely affect gene identification [40], thereby

leading to interference in the downstream analysis. Thus, although VirSorter can achieve a relatively good performance on long contigs, caution should be taken when applying VirSorter to data generated by the third-generation sequencing technology.

Considering that the error rate of the raw data generated from the third-generation sequencing technology may be much higher, we also tested PPR-Meta and the related tools using artificial contigs modified with 10% base substitutions and 10% insertions or deletions in Group D, whose lengths were close to the raw reads generated from third-generation sequencing technology. The results are shown in Additional File 3 and Figure S3. The results showed that the AUCs of PPR-Meta remained the highest (>90%), although the performance was somewhat fluctuating, especially in the presence of 10% insertions or deletions. Recently, many dedicated tools have been developed to help improve the consensus accuracy for the third-generation sequencing technology over 99% [41], therefore the extremely high error rate on the raw data should not affect the usage of PPR-Meta on assembled 3rd generation sequences.

Table 3. Identification performance of each tool with 1% base substitutions.

Table 4. Identification performance of each tool with 1% base insertions or deletions.

Prophage identification ability

We tested the prophage identification ability of the related tools on the 267 manually annotated prophages. The results in Table 5 showed that although the recognition rate of PPR-Meta for prophages was lower than that for phage contigs generated from the NCBI database, the overall performance of PPR-Meta was still much better than that of VirFinder and VirSorter. We additionally collected 139 manually verified prophages from the PHANTOME database [42], and most of the hosts of these prophages were not the same as those of the previous 267 prophages. Consistent with the results for the 267 prophages, the prophage recognition rate of PPR-Meta was much higher than that of the comparative tools (shown in Additional File 3, Figure S4), indicating that PPR-Meta can identify more prophages. The lower recognition rate of prophages compared with that of the phages in the NCBI database may be due to the difference of sequence pattern between prophages and phages in the NCBI database. The complete genomes in the NCBI database tend to come from phages that are easily obtained experimentally while prophages hide their genomes in the hosts. During co-evolution, prophages may adjust the sequence pattern according to their hosts to eliminate the hosts' restriction enzymes [39]. In terms of VirFinder, the ability of identifying prophages was significantly reduced and more than half of the prophages were missed, which may be because VirFinder ignored prophages that exist in the chromosomes

during training. In both the training and test set of VirFinder, all prophages were labelled as chromosomes, which led to the misjudgement of prophages. In microbial communities, temperate phages are dominant and a significant portion of temperate phages exist in the form of prophages [43]. For example, prophage have been shown to represent the main component of phages in healthy human guts [17]. In certain prokaryotes, prophages account for up to 20% of the host chromosome [27]. Thus, compared with VirFinder, PPR-Meta may be more adapted to real microbial communities since it can identify more prophages.

Table 5. Recognition rate of prophages

Evaluation in real metagenomic data

We also evaluated PPR-Meta and the related tools using real metagenomic data. We first evaluated whether PPR-Meta can identify MGEs using both phage metagenomic and plasmid metagenomic data of bovine rumens, in which either phages or plasmids were enriched before sequencing. The phage metagenomic data were downloaded as raw reads, and a total of 107,529 contigs were generated after assembly. VirSorter, VirFinder and PPR-Meta were run on the phage metagenome. Consistent with the results for artificial contigs, VirSorter missed nearly all the phages and only 0.02% of the contigs were identified. VirFinder and PPR-Meta were much better than VirSorter and

identified 68.86% and 76.90% of the contigs, respectively, showing that PPR-Meta had the highest coverage of this data set.

The plasmid metagenomic data were downloaded as assembled contigs containing 5771 sequences. It is worth noting that there are a certain number of phages survive as circular DNA [17], and when enriching plasmids, these circular phages will also be extracted together with the plasmids. Thus, the plasmid metagenome may contain a mixture of phages and plasmids in which the host chromosomes are filtered. From the RefSeq viral database, we collected the genes labelled as “portal”, “spike”, “major capsid protein”, “terminase large subunit”, “tail”, “coat”, or “virion formation”, which were more likely to exist in phages [14]. We found that one of the sequences contained a homologous region of the portal protein by applying the blastx search (e-value<=1e-4), indicating that phages are likely to co-exist with plasmids in this dataset. Therefore, all of PPR-Meta, VirSorter, VirFinder, cBar and PlasFlow were run on this dataset. Results showed that VirSorter did not identify any sequences as phages while VirFinder identified 49.90% as phages. In terms of cBar and PlasFlow, they identified 64.46% and 74.67% of the sequences as plasmids. For PPR-Meta, total of 81.96% of the sequences were identified as MGEs, in which 49.18% were phages and 32.78% were plasmids. More than half of the sequences (64.73%) predicted as phages by PPR-Meta were also predicted as phages by VirFinder, and most of the sequences (74.74%) predicted as plasmids by PPR-Meta were also predicted by PlasFlow.

Furthermore, the sequence containing the homologous region of the portal protein was identified as phages and 8 out of 10 sequences coding plasmid backbone functions listed in Figure 3 of [32] were also identified as plasmids by PPR-Meta. Thus, the prediction of PPR-Meta may be reliable. Because of the filtering of chromosomes from this dataset, PPR-Meta could identify most of the extrachromosomal elements with the fewest false negative predictions.

Since we lack samples in which only chromosomes are enriched and all the extrachromosomal elements are filtered, estimating whether related tools will misjudge chromosomes as MGEs directly is difficult using real data. Because 16S rRNA is more likely to occur in chromosomes, sequences containing the homologous region of 16S rRNA are likely chromosome-derived. We collected 20 metagenome samples from the human gut, which represented mixtures of phages, chromosomes and plasmids. All contigs of the samples were searched against the 16S rRNA database of Greengeen [44] using blastn, and the contigs containing the homologous region (e-value<=1e-4, hits length>=250) of 16S rRNA were collected. Hits longer than 250 bp could cover at least one conserved region of 16S rRNA so the alignments were reliable. In terms of phage identification, PPR-Meta, VirFinder and VirSorter predicted an average of 3.43%, 11.32% and 0% of the 16S-like contigs as phages, respectively, indicating that PPR-Meta likely generated fewer false positive predictions than VirFinder. Although VirSorter did not cover any of the 16S-like contigs, the low number of false positive predictions came at the cost of missing almost all

phages as shown above. In terms of plasmid identification, PPR-Meta, PlasFlow and cBar predicted an average of 26.69%, 52.57% and 63.36% of the 16S-like contigs as plasmids, respectively, indicating that the PPR-Meta may generate the lowest number of false positive predictions. Because individual extrachromosomal elements also contain ribosomal RNA, especially large plasmids [45], the coverage of 16S-like contigs may be higher than the real FPR. Overall, PPR-Meta can identify more MGEs with fewer false positives.

Considering that the third-generation sequencing technology is more and more widely used to analyse metagenomes, we also used real virome data generated by MinION [46] to test whether PPR-Meta and the related tools can identify phages from third-generation sequencing technology. The virome was downloaded as assembled sequences (accession: GCA_900491955.1), containing 1500 sequences. The results showed that PPR-Meta, VirFinder and VirSorter could identify 79.20%, 76.27% and 30.40% of viral sequences respectively, indicating that PPR-Meta has the highest performance. Therefore PPR-Meta can also handle data from the third-generation sequencing technology, although it is designed primarily for the second-generation sequencing technology.

Phages and plasmids in the human digestive tract

As an application example, we employed PPR-Meta to analyse the percentages of phages, bacterial chromosomes and plasmids in microbial communities from

the human digestive tract. We collected 10 samples from the gut (sampling from stools), 7 samples from the throat and 10 samples from the oral cavity (sampling from the tongue dorsum). All samples were downloaded from the Human Microbiome Project (HMP) [47] as assembled contigs. The accessions of all samples are provided in Additional File 1. PPR-Meta was run on all samples, and the percentages of sequences predicted as phages, chromosomes and plasmids were calculated. The results are shown in Figure 3. We found that in the positions closer to the outer end of the digestive tract, the percentages of phages and plasmids tended to be higher. For example, in the gut, the inner end of the digestive tract, the percentages of phages and plasmids were lower, while in the oral cavity, the outer end of the digestive tract, the percentages were higher. Especially, phage sequences occupied approximately 14.80% of all sequences in the gut, which was consistent with the viral proportion in the human gut (4~17%) estimated earlier [48]. In the oral cavity, the percentage of phage sequences was obviously higher, occupying approximately 26.23% of all sequences. It has been reported that the number of phages in the oral cavity is estimated to be 35 times more than that of bacteria [49], indicating that the high percentage of phages predicted by PPR-Meta may be reliable. Moreover, the high percentages of phages and plasmids means that HGT may be more frequent. Since the outer end of the digestive tract is closer to the changing external environment, HGT seems to be a way for microbial communities at the outer end of the digestive tract to adapt to the

external environment.

Figure 3. Percentages of phages, chromosomes and plasmids in the human digestive tract. PPR-Meta was used to predict the sequences of phages, chromosomes and plasmids in metagenomic assemblies, including samples from the gut, throat and oral cavity. The sequence percentages of phages, chromosomes and plasmids were calculated.

Usage of PPR-Meta

PPR-Meta takes the sequence file in fasta format as input and outputs a tabular file. The output file contains three scores between 0 and 1 that reflect the likelihood of obtaining phages, chromosomes and plasmids for each sequence. By default, the final prediction is the category with the highest score. To meet users' actual requirements, PPR-Meta is designed with the option to adjust the threshold to filter out the uncertain predictions so that the remaining predictions may be more reliable. Given a threshold, a sequence with a highest score lower than the threshold will be labelled as "uncertain". In this way, the outputs of PPR-Meta contain six categories: phage, uncertain phage, chromosome, uncertain chromosome, plasmid and uncertain plasmid. We evaluated the uncertain prediction rate, accuracy, AUC, TPR and FPR under different thresholds, and the results are shown in Additional File 3, Figure S5. In general, with a higher threshold, the accuracy, AUC, and TPR as well as the uncertain

prediction rate will be higher, while the FPR will be lower.

PPR-Meta is user friendly, and the program has been optimized in a virtual machine so that users can directly run PPR-Meta without installing any dependency package. We also provided a short video guide to show how to install the virtual machine. If users are analysing large-scale data, running the executable file on the physical host is more suitable. In this way, when the GPU is available, PPR-Meta will run on the GPU automatically to speed up the program. The memory requirements are dependent on the data size. We recommend at least 4~6 GB of available memory when running the virtual machine or at least 16 GB when handling large-scale data on the physical host. We tested the running time of PPR-Meta using 90,000 sequences from 100 to 10k bp and found that this tool can handle all sequences in approximately 45 minutes on a machine with the following configuration: CPU: Intel Core i7 6700; GPU: NVIDIA GTX1060; and Memory: 64G, DDR4.

Discussion and conclusions

In this paper, we proposed an ab initio method, PPR-Meta, to identify both phages and plasmids from metagenomic sequences. PPR-Meta employs a novel strategy to improve the MGE identification performance and avoids performing similarity searches to make judgments. Similarity search-based tools, such as VirSorter, provide good results for long sequences. However, such methods do not work effectively for short fragments due to the insufficient

number of genes for the statistical analysis. Compared with other reference-free tools, PPR-Meta employs a more detailed method of characterizing DNA sequences. We use a BOH matrix, which is beneficial to non-coding regions, and a COH matrix, which is beneficial to coding regions, to represent sequences. In contrast, traditional k-mer methods do not consider coding or non-coding regions. When the sequence is short, k-mer frequencies will be noisy. On the other hand, k-mer-based methods may also be more sensitive to the sequence length than the BiPathCNN method in the current work. The distribution of k-mer frequencies may be different between long sequences and short sequences, and the variance of the k-mer frequencies for short sequences may be much higher. Thus, the k-mer-based classifier constructed using short sequence data may not by applicable for long sequences, and vice versa. Among the k-mer-based tools, cBar was trained with complete genomes and PlasFlow was trained on 10k bp fragments, which might make them hard to adapt to metagenomic data with a wide range of lengths. Differently, our BiPathCNN directly extracts sequence features from the raw data represented by the one-hot matrix and may be less sensitive to the sequence length. Tests of each BiPathCNN on test datasets from different groups (Additional File 3, Figure S2) also showed that although the overall accuracy was slightly reduced when testing each group using a non-corresponding BiPathCNN from the other groups, the decrease was not obvious, indicating that our approach is not quite sensitive to the sequence length. Another shortcoming of k-mer-based tools

may be that mapping sequences of different length for k-mer feature vectors with the same dimension will also lose some information. PPR-Meta takes all bases and codons as inputs in the neural network, thereby exploiting all information in the fragments. In the design of the algorithm, we employed a deep learning network as the classifier. Deep learning has achieved great success in many fields, such as lncRNA identification [50] and the prediction of sequence specificities of nucleic acid binding proteins [51]. In the construction of PPR-Meta, we designed the BiPathCNN, which contains a “base path” and “codon path” to handle the BOH matrix and COH matrix, respectively. Testing showed that the performance of the CNN with double paths was better than that with single path.

Furthermore, we were surprised to find that PPR-Meta’s output scores were able to describe the interaction between phages or plasmids and their hosts. Specifically, the difference between the phage score and chromosome score reveals the lifestyle of the phages (virulent or temperate), while the difference between the plasmid score and chromosome score reveals the transmissibility of the plasmids (transmissible or non-transmissible). We collected both phage genomes with lifestyle annotations from McNair et al. [8] and plasmid genomes with transmissibility annotations from Shintani et al. [52] and then extracted artificial contigs. PPR-Meta was run on all the contigs and the correctly predicted contigs were retained. From the results, two normalized statistics were constructed:

$$life\_score=(phage\_score-chromosome\_score)/phage\_score$$

and

$$trans\_score=(plasmid\_score-chromosome\_score)/plasmid\_score$$

The receiver operating characteristic curve (ROC) showed that life_score could distinguish the lifestyle of phages while trans_score could distinguish the transmissibility of plasmids with AUC values higher than 0.5 (shown in Figure 4). Specifically, temperate phages tend to have lower life_score values and non-transmissible plasmids tend to have lower trans_score values. This phenomenon may be due to the genome amelioration of foreign DNA to the host. For example, research has shown that the comparison of the trinucleotide composition between a plasmid and bacterial chromosome can be used to predict the host range of plasmids [53]. Since temperate phages and non-transmissible plasmids experience longer residence times within the host cell, they may adjust the sequence pattern toward the host. Thus, the sequence pattern between temperate phages (or non-transmissible plasmids) and host chromosomes may be more similar than that between virulent phages (or transmissible plasmids) and host chromosomes, thereby resulting in a lower life_score (or trans_score) value. Although tools that can classify phage lifestyle and plasmid transmissibility on metagenomes are lacking as far as we know, the phenomena mentioned above may provide insights into the classification strategy for future studies.

Figure 4. ROC curve of classifying phage lifestyle and plasmid transmissibility. (a) Classify virulent phages and temperate phages using life_score. In order of sequence length, the AUC is 0.63, 0.69, 0.71 and 0.76. (b) Classify transmissible plasmid and non-transmissible plasmid using trans_score. In order of sequence length, the AUC is 0.58, 0.55, 0.60 and 0.62.

In general, bacteria contain genomic islands, regions of horizontal origin on chromosomes [54]. The formation mechanisms of some genomic islands are caused by phages or plasmids [55]. To see how PPR-Meta and related tools perform on DNA fragments from these regions, we collected genomic island sequences from the Islander database [56]. Upon testing on artificial contigs between 100 bp and 10k bp extracted from these genomic islands, the results showed that PPR-Meta could identify 65.25% of them as foreign DNA (either phage or plasmid), while VirFinder, VirSorter, PlasFlow and cBar could identify 20.46%, 6.72%, 53.11% and 51.62% of them, respectively, indicating that PPR-Meta has a better ability to recognize sequences from regions of horizontal origin on bacterial chromosomes.

PPR-Meta also has some limitations. In addition to prokaryote chromosomes, plasmids and phages, other organisms of low-abundance may exist in the microbial community, such as fungi and protozoan. Such organisms are not included in the training set of PPR-Meta and may have interfered with the judgment of PPR-Meta. To increase the suitability of PPR-Meta for real

scenes, we will retrain PPR-Meta regularly with expanded datasets. More organisms as well as the newly sequenced genomes will be added to the dataset so that PPR-Meta will be more powerful and reliable. In addition, due to sequence exchanges among phages, plasmids and chromosomes, there are a few chimeric sequences from two sources (e.g., a prophage and chromosome chimera). PPR-Meta cannot perform detailed judgments about these chimeras, and we are considering how to further identify such sequences. However, because these chimeras do not exist at a large scale, we believe that the presence of chimeras will not have a significant impact on the application of PPR-Meta.

In conclusion, the performance of PPR-Meta has shown much better than that of currently available similar tools, while none of these tools can function as PPR-Meta does. It is thus expected that the PPR-Meta tool would meet the demand of metagenomics analysis when considered the microbial community tangled with phages and plasmids, and certainly qualifies as a powerful tool for the research community.

Methods

PPR-Meta was trained and tested using artificial contigs. We downloaded the accession list of prokaryote chromosomes, prokaryote plasmids and phages from the NCBI genome database, and the corresponding genomes were downloaded according to the list. To ensure the quality of the data, we only

used the complete genomic molecule with the RefSeq accession prefix. Since chromosomes may contain prophages, we used ProphET to extract the prophage regions of all chromosomes. ProphET requires a genome sequence file (fasta format) and genome annotation file (gff format) as inputs. A few genomes do not contain the annotation information, and these genomes were removed from the dataset. We then used MetaSim to generate four groups of artificial contigs with different lengths as mentioned in the main text. To generate artificial contigs with no error for both training and test set, we used the “exact” preset to return fragments exactly matching reference sequences. In each group, the “DNA Clone Size Distribution Type” was set to “Uniform”. To generate artificial contigs modified with sequencing errors, we used the “Sanger” preset, which allowed users to modify sequences according to their settings. Note that as we were not going to generate sequences with technology-specific errors, the following settings do not reflect the real situation of the Sanger technology. For the generation of sequences with 1% base substitutions, the “Read Length Distribution Type” was set to “Uniform”, and the “Mate Pair Probability” was set to 0; both the “Error Rate at Read Start” and the “Error Rate at End of Read” were set to 0.01; and both the “Insertion Error Rate” and “Deletion Error Rate” were set to 0. For the generation of sequences with 1% base insertions or deletions, most settings were the same as mentioned above, except that both the “Insertion Error Rate” and “Deletion Error Rate” were set to 0.5. In general, the performance of the algorithm will be better as the amount

of training data increases. Considering the memory size, running time and accuracy, a total of 2,700,000 artificial contigs were generated to train PPR-Meta. The number of training contigs of each phage, chromosome and plasmid is 300,000 from Group A to C.

We also used real metagenomic data to evaluate PPR-Meta and the related tools. We used SPAdes to assemble the raw reads, as we mentioned in the main text. The phage metagenomic data of the bovine rumen were downloaded from MG-RAST, and we used the command "spades.py --meta -1 file1.fastq -2 file2.fastq -o out_folder" to assemble the pair-end raw reads. In the assembly, the contig number, N50, average length, maximum length and minimum length were 107529, 288, 312.06, 75508 and 56, respectively. To download the 20 samples of the healthy human gut, we used the command "prefetch SRRaccession" from the SRA Toolkit. All samples were downloaded as ".sra" files. We then used the command "fastq-dump --split-files accession.sra" from the SRA Toolkit to convert the sra file into two pair-end fastq files and used SPAdes with the same settings as mentioned above to assemble the raw reads. The information about the contig number, N50, average length, maximum length and minimum length is provided in Additional File 1.

The artificial contigs are stored at http://cqb.pku.edu.cn/ZhuLab/PPR_Meta/data/.

Availability of supporting source code and requirements

Project name: PPR-Meta.

Project home page: http://cqb.pku.edu.cn/ZhuLab/PPR_Meta or https://github.com/zhenchengfang/PPR-Meta.

Operating system: The code of PPR-Meta was written on Linux. We optimized the program in a virtual machine thus PPR-Meta is platform independent.

Programming language: python, matlab

Other requirements: no other requirements are needed if running in the virtual machine. If not, Python 2.7.12, TensorFlow 1.4.1, Keras 2.0.8 and MATLAB Component Runtime 2018a (for free) are needed. MATLAB is not necessary.

License: GPL-3.0.

RRID: SCR_016915

Availability of supporting data

The artificial contigs, related scripts and original results are available at http://cqb.pku.edu.cn/ZhuLab/PPR_Meta/data/. All the other data are available at corresponding references mentioned in the main text. Snapshots of our code and other supporting data are available in the GigaScience repository, GigaDB [57].

Additional file

Additional file 1: accession list of the data used to train and test PPR-Meta.

Additional file 2: prophage coordinate predicted by ProphET.

Additional file 3: Figure S1 to Figure S5.

List of abbreviations

MGEs: mobile genetic elements

HGT: horizontal gene transfer

WGS: Whole Genome Sequencing

SMO: sequential minimal optimization

BiPathCNN: Bi-path Convolutional Neural Network

BOH: base one-hot matrix

COH: codon one-hot matrix

TPR: true positive rate

FPR: false positive rate

AUC: area under curve

ROC: receiver operating characteristic

HMP: Human Microbiome Project

Ethics approved and consent to participate

Not applicable

Consent for publication

Not applicable

Competing interests

The authors declare that they have no competing interests

Funding

This work was supported by the National Key Research and Development Program of China (2017YFC1200205), the National Natural Science Foundation of China (31671366), and the Special Research Project of ‘Clinical Medicine + X’ by PKU.

Authors' contributions

HQZ and ZCF proposed and designed the study. ZCF, JT and SFW constructed the data sets, and wrote and optimized the code. ML, CMX and ZJX tested the program. ZCF and HQZ wrote and revised the manuscript and all authors proofread and improved the manuscript.

Acknowledgements

We thank Dr. Cheng Yang, Dr. Longshu Yang, Dr. Xiaoqing Jiang, Li Qu of Peking University for their helpful discussions. Part of the analysis was performed on the High Performance Computing Platform of the Center for Life Science of Peking University.

References

[1] Frost LS, Leplae R, Summers AO, Toussaint A. Mobile genetic elements: the agents of open source evolution. *Nat. Rev. Microbiol.*, 2005;3(9): 722.

[2] Brown-Jaque M, Calero-Cáceres W, Muniesa M. Transfer of antibiotic-resistance genes via phage-related mobile elements. *Plasmid*, 2015;*79*: 1-7.

[3] Paul JH, Sullivan MB. Marine phage genomics: what have we learned?. *Curr. Opin. Biotechnol.*, 2005:16(3): 299-307.

[4] Sobecky PA, Hazen TH. Horizontal gene transfer and mobile genetic elements in marine systems. In *Horizontal Gene Transfer*, 2009; 435-453, Humana Press.

[5] Hayes S, Mahony J, Nauta A, van Sinderen D. Metagenomic approaches to assess bacteriophages in various environmental niches. *Viruses*, 2017; 9(6): 127.

[6] Li LL., Norman A, Hansen LH, Sørensen SJ. Metamobilomics-expanding our knowledge on the pool of plasmid encoded traits in natural environments using high-throughput sequencing. *Clin. Microbiol. Infect.*, 2012: 18, 5-7.

[7] Rozov R, Brown Kav A, Bogumil D, Shterzer N, Halperin E, Mizrahi I, Shamir R. Recycler: an algorithm for detecting plasmids from de novo assembly graphs. *Bioinformatics*, 2017; 33(4): 475-482.

[8] McNair K, Bailey BA, Edwards RA. PHACTS, a computational approach to classifying the lifestyle of phages. *Bioinformatics*, 2012; 28(5): 614-618.

[9] Lima-Mendez G, Van Helden J, Toussaint A, Leplae R. Prophinder: a computational tool for prophage prediction in prokaryotic genomes.

Bioinformatics, 2008; 24(6): 863-865.
[10] Fouts DE. Phage_Finder: automated identification and classification of prophage regions in complete bacterial genome sequences. *Nucleic Acids Res.*, 2006; 34(20): 5839-5851.
[11] Akhter S, Aziz RK, Edwards RA. PhiSpy: a novel algorithm for finding prophages in bacterial genomes that combines similarity-and composition-based strategies. *Nucleic Acids Res.*, 2012; 40(16): e126-e126.
[12] Zhou Y, Liang Y, Lynch KH, Dennis JJ, Wishart DS. PHAST: a fast phage search tool. *Nucleic Acids Res.*, 2011; 39(suppl_2): W347-W352.
[13] Arndt D, Grant JR, Marcu A, Sajed T, Pon A, Liang Y, Wishart DS. PHASTER: a better, faster version of the PHAST phage search tool. *Nucleic Acids Res.*, 2016; 44(W1): W16-W21.
[14] Roux S, Enault F, Hurwitz B L, Sullivan MB. VirSorter: mining viral signal from microbial genomic data. *PeerJ*, 2015; 3: e985.
[15] Reis-Cunha JL., Bartholomeu DC, Earl AM, Birren BW, Cerqueira GC. ProphET, Prophage Estimation Tool: a standalone prophage sequence prediction tool with self-updating reference database. 2017; *bioRxiv*, 176750.
[16] Liu Y, Guo J, Hu G, Zhu H. Gene prediction in metagenomic fragments based on the SVM algorithm. *BMC Bioinformatics*, 2013; 14(5): S12.
[17] Mirzaei MK, Maurice CF. Ménage à trois in the human gut: interactions between host, bacteria and phages. *Nat. Rev. Microbiol.*, 2017; 15(7), 397.
[18] Amgarten DE, Braga LPP, Da Silva AM, Setubal JC. MARVEL, a Tool for

Prediction of Bacteriophage Sequences in Metagenomic Bins. *Front. Genet.*, 2018; 9: 304.

[19] Ren J, Ahlgren NA, Lu YY, Fuhrman JA, Sun F. VirFinder: a novel k-mer based tool for identifying viral sequences from assembled metagenomic data. *Microbiome*, 2017; 5(1): 69.

[20] Carattoli A, Zankari E, García-Fernández A, Larsen MV, Lund O, Villa L, Aarestrup FM, Hasman H. In silico detection and typing of plasmids using PlasmidFinder and plasmid multilocus sequence typing. *Antimicrob. Agents Chemother.*, 2014; 58(7): 3895-3903.

[21] Lanza VF, de Toro M, Garcillán-Barcia MP, Mora A, Blanco J, Coque TM, de la Cruz F. Plasmid flux in Escherichia coli ST131 sublineages, analyzed by plasmid constellation network (PLACNET), a new method for plasmid reconstruction from whole genome sequences. *PLoS Genet.*, 2014; 10(12): e1004766.

[22] Roosaare M, Puustusmaa M, Möls M, Vaher M, Remm M. PlasmidSeeker: identification of known plasmids from bacterial whole genome sequencing reads. 2018; *PeerJ*, 6: e4588.

[23] Arredondo-Alonso S, Rogers MRC, Braat JC, Verschuuren TD, Top J, Corander J, Willems RJL, Schürch AC. mlplasmids: a user-friendly tool to predict plasmid- and chromosome-derived sequences for single species. *Microb. Genom.*, 2018; 4(11).

[24] Zhou F, Xu Y. cBar: a computer program to distinguish plasmid-derived

from chromosome-derived sequence fragments in metagenomics data. *Bioinformatics*, 2010; 26(16): 2051-2052.

[25] Krawczyk PS, Lipinski L, Dziembowski, A. PlasFlow: predicting plasmid sequences in metagenomic data using genome signatures. *Nucleic Acids Res.*, 2018; 46(6): e35-e35.

[26] The NCBI genome database. ftp://ftp.ncbi.nlm.nih.gov/genomes/GENOME_REPORTS/. Accessed 12 July 2018.

[27] Casjens S. Prophages and bacterial genomics: what have we learned so far?. *Mol. Microbiol.*, 2003; 49(2): 277-300.

[28] Richter DC, Ott F, Auch AF, Schmid R, Huson DH. MetaSim—a sequencing simulator for genomics and metagenomics. *PLoS One*, 2008; 3(10): e3373.

[29] Ross EM, Petrovski S, Moate PJ, Hayes BJ. Metagenomics of rumen bacteriophage from thirteen lactating dairy cattle. BMC Microbiol., 2013; 13(1): 242.

[30] Meyer F, Paarmann D, D'Souza M, Olson R, Glass EM, Kubal M, Paczian T, Rodriguez A, Stevens R, Wilke A, Wilkening J. The metagenomics RAST server–a public resource for the automatic phylogenetic and functional analysis of metagenomes. *BMC Bioinformatics.* 2008; 9(1): 386.

[31] Bankevich A, Nurk S, Antipov D, Gurevich AA, Dvorkin M, Kulikov AS, Lesin VM, Nikolenko SI, Pham S, Prjibelski AD, Pyshkin AV. SPAdes: a new genome assembly algorithm and its applications to single-cell sequencing. *J.*

Comput. Biol., 2012; 19(5): 455-477.

[32] Kav AB, Sasson G, Jami E, Doron-Faigenboim A, Benhar I, Mizrahi I. Insights into the bovine rumen plasmidome. *Proc. Natl. Acad. Sci. U S A*, 2012; 109(14): 5452-5457.

[33] Qin J, et al. A metagenome-wide association study of gut microbiota in type 2 diabetes. *Nature*, 2012; 490(7418): 55-60.

[34] NCBI Short Read Archive. https://www.ncbi.nlm.nih.gov/sra/. Accessed 12 July 2018.

[35] Galiez C, Siebert M, Enault F, Vincent J, Söding J. WIsH: who is the host? Predicting prokaryotic hosts from metagenomic phage contigs. *Bioinformatics*, 2017; 33(19): 3113-3114.

[36] Braud C, Denis P. (2015). Comparing word representations for implicit discourse relation classification. *In Proceedings of the 2015 Conference on Empirical Methods in Natural Language Processing*, (2015); 2201-2211.

[37] LeCun Y, Bottou L, Bengio Y, Haffner P. Gradient-based learning applied to document recognition. *Proceedings of the IEEE*, 1998; 86(11): 2278-2324.

[38] Simonyan K, Zisserman A. Very deep convolutional networks for large-scale image recognition. *arXiv preprint arXiv*, 2014: 1409.1556.

[39] Edwards RA, McNair K, Faust K, Raes J, Dutilh BE. Computational approaches to predict bacteriophage–host relationships. *FEMS Microbiol. Rev.*, 2015; 40(2): 258-272.

[40] Rho M, Tang H, Ye Y. FragGeneScan: predicting genes in short and error-

prone reads. *Nucleic Acids Res.*, 2010; 38(20): e191-e191.

[41] Wick RR, Judd LM, Holt KE. Comparison of Oxford Nanopore Basecalling Tools. *URL https://doi.org/10.5281/zenodo.1082696*, 2017.

[42] Aziz, Ramy, Akhter, Sajia, Schmieder, Robert, Edwards, Robert A. PhAnToMe. Phage annotation, tools, and Methods. http://www.phantome.org/Downloads/Prophages/PhiSpy/. Accessed 9 January 2019.

[43] Hurwitz BL, Ponsero A, Thornton J, U'Ren JM. Phage Hunters: computational strategies for finding phages in large-scale 'omics datasets. *Virus Res.*, 2018; 244(15): 110-115.

[44] DeSantis TZ, Hugenholtz P, Larsen N, Rojas M, Brodie EL, Keller K, Huber T, Dalevi D, Hu P, Andersen GL. Greengenes, a chimera-checked 16S rRNA gene database and workbench compatible with ARB. *Appl. Environ. Microbiol.*, 2006; 72(7): 5069-5072.

[45] Smillie C, Garcillán-Barcia MP, Francia MV, Rocha EP, de la Cruz F. Mobility of plasmids. *Microbiol. Mol. Biol. R.*, 2010; 74(3): 434-452.

[46] Warwick-Dugdale J, Solonenko N, Moore K, Chittick L, Gregory AC, Allen MJ, Sullivan MB, Temperton B. Long-read metagenomics reveals cryptic and abundant marine viruses. *bioRxiv*, 2018; 345041.

[47] Turnbaugh PJ, Ley RE, Hamady M, Fraser-Liggett CM, Knight R, Gordon JI. The human microbiome project. *Nature*, 2007; 449(7164): 804-810.

[48] Minot S, Sinha R, Chen J, Li H, Keilbaugh SA, Wu GD, Lewis JD, Bushman

FD. The human gut virome: inter-individual variation and dynamic response to diet. *Genome Res.*, 2011; 21:1616–25.

[49] Edlund A, Santiago-Rodriguez TM, Boehm TK, Pride DT. Bacteriophage and their potential roles in the human oral cavity. *J. Oral Microbiol.*, 2015; 7(1): 27423.

[50] Yang C, Yang L, Zhou M, Xie H, Zhang C, Wang MD, Zhu H. LncADeep: An ab initio lncRNA identification and functional annotation tool based on deep learning. *Bioinformatics*, 2018; 34(22): 3825-3843.

[51] Alipanahi B, Delong A, Weirauch MT, Frey BJ. Predicting the sequence specificities of DNA-and RNA-binding proteins by deep learning. *Nat. Biotechnol.*, 2015; 33(8): 831-838.

[52] Shintani M, Sanchez ZK, Kimbara K. Genomics of microbial plasmids: classification and identification based on replication and transfer systems and host taxonomy. *Front. Microbiol.*, 2015; 6:242.

[53] Suzuki H, Yano H, Brown CJ, Top EM. Predicting plasmid promiscuity based on genomic signature. *J. Bacteriol.*, 2010; 192(22): 6045–6055.

[54] Bertelli C, Laird MR, Williams KP, Simon Fraser University Research Computing Group, Lau BY, Hoad G, Winsor GL, Brinkman FSL. IslandViewer 4: expanded prediction of genomic islands for larger-scale datasets. *Nucleic Acids Res.*, 2017; 45(W1): W30-W35.

[55] Juhas M, Van Der Meer JR, Gaillard M, Harding RM, Hood DW, Crook DW. Genomic islands: tools of bacterial horizontal gene transfer and evolution.

FEMS Microbiol. Rev., 2009; 33(2):376-93.

[56] Hudson CM, Lau BY, Williams KP. Islander: a database of precisely mapped genomic islands in tRNA and tmRNA genes. *Nucleic Acids Res.*, 2015; 43: D48-53.

[57] Fang Z; Tan J; Wu S; Li M; Xu C; Xie Z; Zhu H: Supporting data for "PPR-Meta: a tool for identifying phages and plasmids from metagenomic fragments using deep learning" GigaScience Database. 2019. http://dx.doi.org/10.5524/100605

Table

Table 1. Evaluation of the performance of PPR-Meta and comparison of the performance of PPR-Meta and related tools.

Group	Tool	Evaluation on phage			Evaluation on plasmid		
		TPR(%)	*FPR*(%)	*AUC*(%)	*TPR*(%)	*FPR*(%)	*AUC*(%)
Group A 100-400 bp	**PPR-Meta**	**84.96**	18.01	**91.82**	59.91	**14.14**	**83.05**
	VirFinder	73.77	25.45	81.30	NA	NA	NA
	VirSorter	0.00	**0.00**	50.00	NA	NA	NA
	PlasFlow	NA	NA	NA	**71.89**	62.59	56.30
	cBar	NA	NA	NA	52.68	46.07	53.31
Group B 400-800 bp	**PPR-Meta**	**90.75**	8.37	**97.21**	**74.56**	**13.37**	**89.64**
	VirFinder	79.27	18.15	88.64	NA	NA	NA
	VirSorter	0.05	**0.002**	50.02	NA	NA	NA
	PlasFlow	NA	NA	NA	72.61	55.01	62.50
	cBar	NA	NA	NA	55.00	43.59	55.70
Group C 800-1200 bp	**PPR-Meta**	**95.24**	7.75	**98.54**	**78.09**	**10.95**	**91.84**
	VirFinder	81.91	15.63	91.09	NA	NA	NA
	VirSorter	0.17	**0.002**	50.09	NA	NA	NA
	PlasFlow	NA	NA	NA	75.89	50.55	68.01
	cBar	NA	NA	NA	55.54	41.87	56.84
Group D 5000-10k bp	**PPR-Meta**	**99.20**	3.25	**99.77**	87.53	**6.45**	**96.02**
	VirFinder	89.26	8.13	97.12	NA	NA	NA
	VirSorter	66.80	**2.48**	82.66	NA	NA	NA
	PlasFlow	NA	NA	NA	**88.50**	30.22	88.42
	cBar	NA	NA	NA	63.79	32.61	65.59

NA: not applicable.

Table 2. Performance comparison among BiPathCNN, the base path-only CNN and codon path-only CNN.

Group	Tool	Evaluation on phage			Evaluation on plasmid		
		TPR(%)	*FPR*(%)	*AUC*(%)	*TPR*(%)	*FPR*(%)	*AUC*(%)
Group A 100-400 bp	**BiPathCNN**	84.96	18.01	91.82	59.91	14.14	**83.05**
	Base path-only	81.86	24.58	87.50	56.96	17.60	78.50
	Codon path-only	86.84	20.47	**91.85**	62.15	16.65	82.26
Group B 400-800 bp	**BiPathCNN**	90.75	8.37	**97.21**	74.56	13.37	**89.64**
	Base path-only	88.76	17.46	93.87	72.37	18.86	85.57
	Codon path-only	84.95	5.93	96.57	82.98	23.10	88.32
Group C 800-1200 bp	**BiPathCNN**	95.24	7.75	**98.54**	78.09	10.95	**91.84**
	Base path-only	92.09	17.71	95.47	73.31	15.12	88.02
	Codon path-only	94.60	12.44	97.55	73.17	12.41	89.22

Table 3. Identification performance of each tool with 1% base substitutions.

Group	Tool	Evaluation on phage			Evaluation on plasmid		
		TPR(%)	*FPR*(%)	*AUC*(%)	*TPR*(%)	*FPR*(%)	*AUC*(%)
Group A 100-400 bp	**PPR-Meta**	**84.42**	17.99	**91.57**	61.19	**15.27**	**82.76**
	VirFinder	72.55	26.20	80.42	NA	NA	NA
	VirSorter	0.00	**0.00**	50.00	NA	NA	NA
	PlasFlow	NA	NA	NA	**71.72**	62.82	55.86
	cBar	NA	NA	NA	52.98	46.18	53.40
Group B 400-800 bp	**PPR-Meta**	**90.05**	8.48	**97.02**	**75.07**	**14.03**	**89.39**
	VirFinder	78.50	18.75	87.95	NA	NA	NA
	VirSorter	0.02	**0.00**	50.01	NA	NA	NA
	PlasFlow	NA	NA	NA	72.31	55.61	61.87
	cBar	NA	NA	NA	54.83	44.63	55.10
Group C 800-1200 bp	**PPR-Meta**	**94.54**	7.72	**98.33**	**79.03**	**11.99**	**91.59**
	VirFinder	81.29	15.92	90.68	NA	NA	NA
	VirSorter	0.21	**0.00**	50.11	NA	NA	NA
	PlasFlow	NA	NA	NA	75.24	50.91	67.15
	cBar	NA	NA	NA	56.57	42.85	56.86
Group D 5000-10k bp	**PPR-Meta**	**98.97**	3.15	**99.75**	87.65	**7.20**	**95.83**
	VirFinder	88.90	8.19	97.01	NA	NA	NA
	VirSorter	60.30	**1.13**	79.80	NA	NA	NA
	PlasFlow	NA	NA	NA	**88.57**	31.42	87.86
	cBar	NA	NA	NA	64.31	34.63	64.84

NA: not applicable.

Table 4. Identification performance of each tool with 1% base insertions or deletions.

Group	Tool	Evaluation on phage			Evaluation on plasmid		
		TPR(%)	*FPR*(%)	*AUC*(%)	*TPR*(%)	*FPR*(%)	*AUC*(%)
Group A 100-400 bp	**PPR-Meta**	**80.26**	18.64	**89.28**	65.29	**19.93**	**81.62**
	VirFinder	72.62	25.96	80.57	NA	NA	NA
	VirSorter	0.00	**0.00**	50.00	NA	NA	NA
	PlasFlow	NA	NA	NA	**71.12**	62.83	55.81
	cBar	NA	NA	NA	53.63	46.43	53.60
Group B 400-800 bp	**PPR-Meta**	**85.50**	9.69	**95.26**	**77.44**	**17.57**	**88.48**
	VirFinder	79.00	18.76	88.28	NA	NA	NA
	VirSorter	0.24	**0.00**	50.12	NA	NA	NA
	PlasFlow	NA	NA	NA	72.74	55.44	62.31
	cBar	NA	NA	NA	55.38	45.22	55.08
Group C 800-1200 bp	**PPR-Meta**	**92.99**	9.12	**97.54**	**79.74**	**14.29**	**90.80**
	VirFinder	81.98	16.00	90.93	NA	NA	NA
	VirSorter	2.38	**0.02**	51.18	NA	NA	NA
	PlasFlow	NA	NA	NA	75.23	51.25	66.75
	cBar	NA	NA	NA	56.74	43.45	56.64
Group D 5000-10k bp	**PPR-Meta**	**98.90**	3.51	**99.73**	**89.23**	**8.48**	**95.84**
	VirFinder	88.93	8.40	96.98	NA	NA	NA
	VirSorter	47.25	**0.25**	73.51	NA	NA	NA
	PlasFlow	NA	NA	NA	88.74	31.70	88.08
	cBar	NA	NA	NA	64.62	35.40	64.61

NA: not applicable.

Table 5. Recognition rate of prophages

Group	Tool	Recognition rate (%)
Group A	**PPR-Meta**	**60.79**

100-400 bp	VirFinder	43.46
	VirSorter	0.00
Group B 400-800 bp	**PPR-Meta**	**60.59**
	VirFinder	40.77
	VirSorter	0.00
Group C 800-1200 bp	**PPR-Meta**	**68.09**
	VirFinder	41.94
	VirSorter	0.05
Group D 5000-10k bp	**PPR-Meta**	**75.58**
	VirFinder	48.62
	VirSorter	37.75

Figure 1

Click here to access/download;Figure;figure_1.pdf

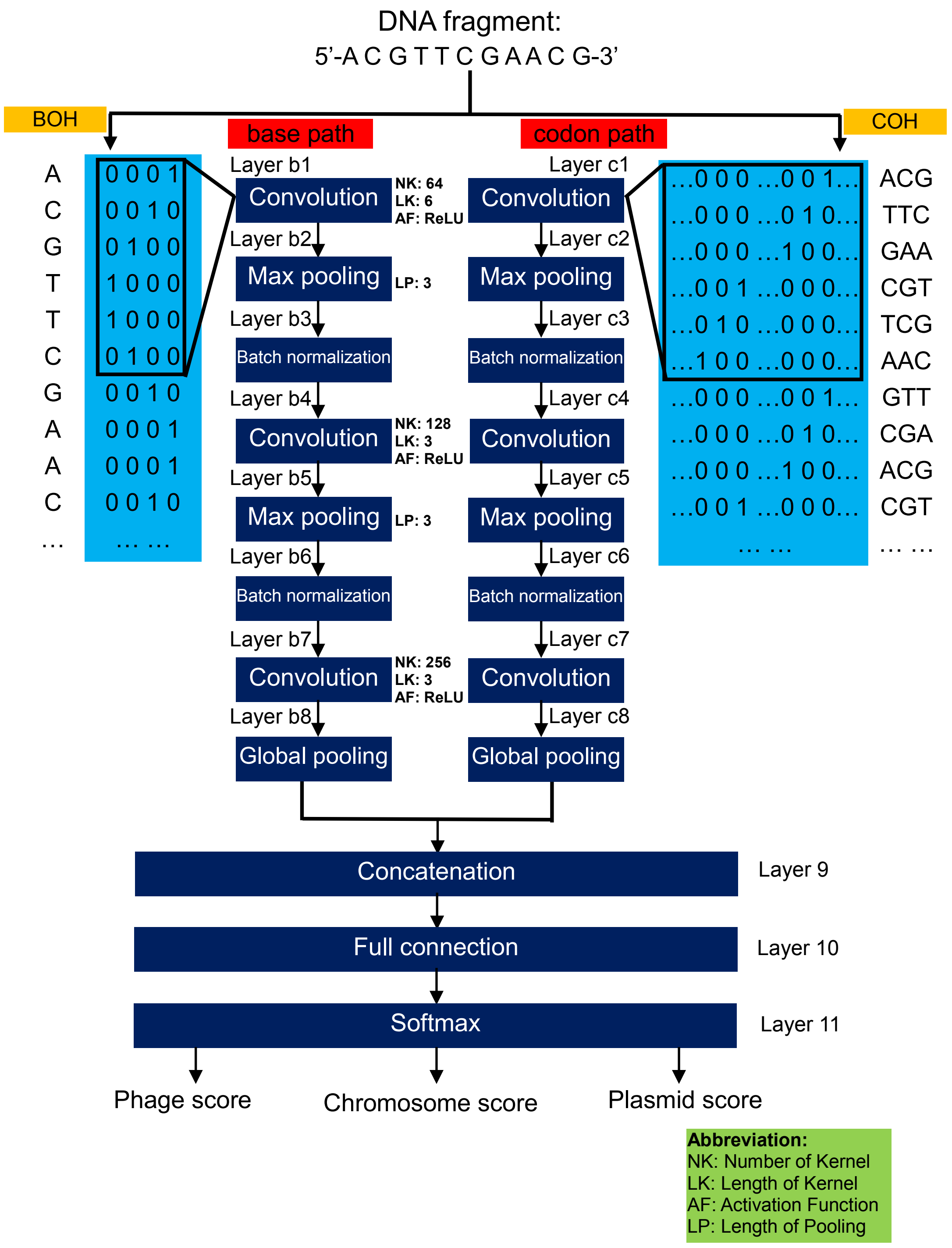

Figure 2

Click here to access/download;Figure;figure_2.pdf

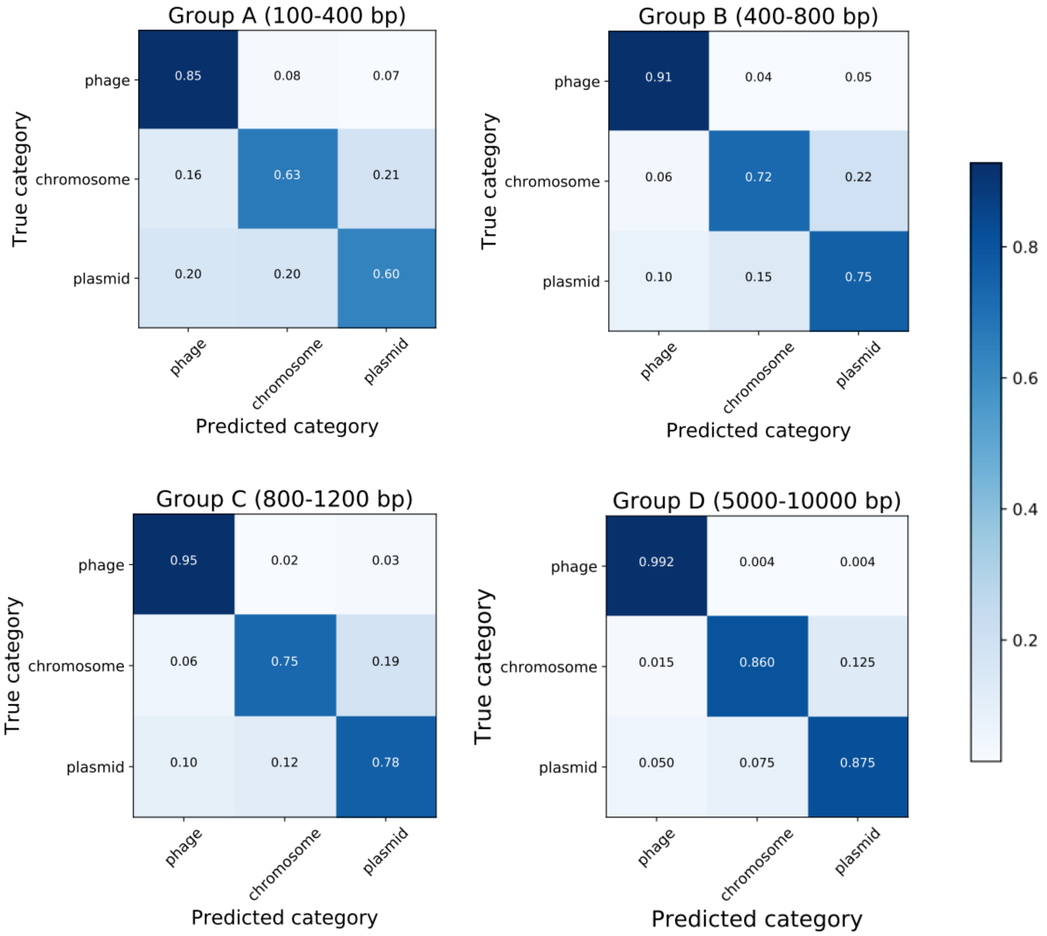

Figure 3

Click here to access/download;Figure;figure_3.pdf

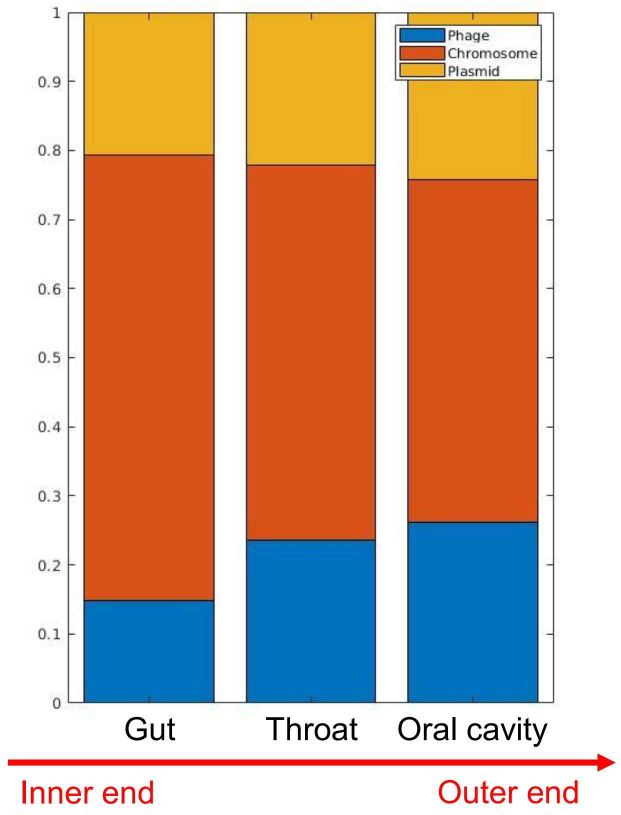

Figure 4

Click here to access/download;Figure;figure_4.jpg

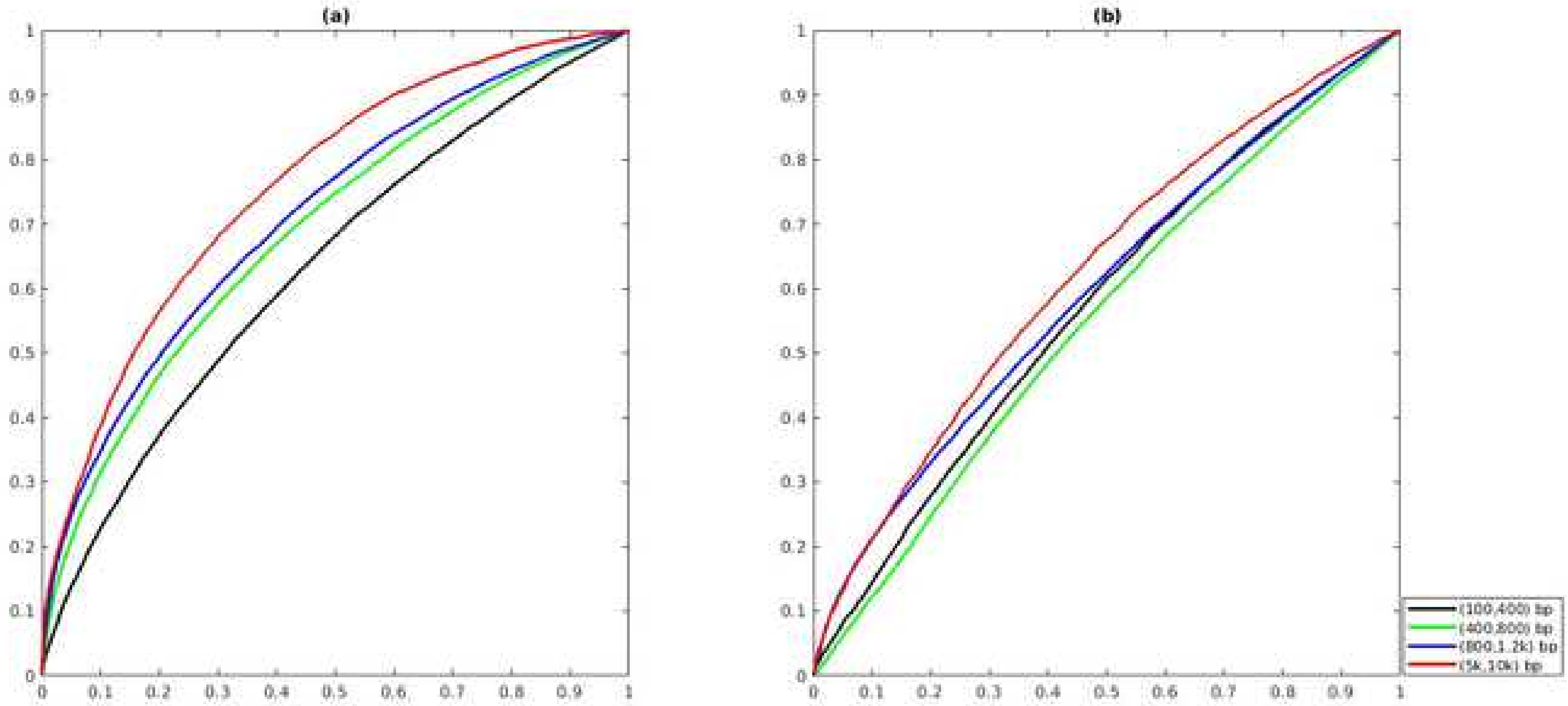

Additional File 1

Click here to access/download
Supplementary Material
Additional file 1.xlsx

Additional File 2

Click here to access/download
Supplementary Material
Additional file 2.xlsx

Additional File 3

Click here to access/download
Supplementary Material
Additional file 3.docx

Click here to access/download;Personal Cover;Cover+Letter+revision+2_revised.docx

Cover Letter

Dear Editor,

Thank you very much for your previous E-mail on April 6, 2019 regarding our manuscript "PPR-Meta: a tool for identifying phages and plasmids from metagenomic fragments using deep learning" (Manuscript ID: GIGA-D-18-00464R1). We are very pleased to know that our manuscript is potentially acceptable for publication in the journal, subject to the suggested further revision following the reviewers. Herein we would like to thank three reviewers and are pleased to know that our previous responses were addressed all of their concerns. Additionally, we are grateful for their careful reading of the previous version of our manuscript, and the further comments raised by three reviewers helped us make further improvements. In the revised manuscript, all the changed words, sentences and paragraphs are marked in red text.

Following your instruction and Reviewers' comments, this time we have made a conscious effort to revise the manuscript with the further improvement. Before we report the revisions and responses to Reviewers' comments, we would like to first present our responses to Editor's instructions. With respect to the website, because the server is regularly maintained, users might be occasionally unable to access it when the server is under maintenance. Therefore we have double-checked our website (http://cqb.pku.edu.cn/ZhuLab/PPR_Meta/), and we confirm that the website is working. Additionally, our tool is stored on our website, as well as GitHub and the GigaScience database, and thus, users can also download the software if our website is under maintenance. The links to our website and the GitHub page are provided in Subsection "Availability of supporting source code and requirements". (Please refer to Lines 2-3, Page 35 in the revised manuscript.) Also, we have already registered PPR-Meta in the SciCrunch.org database, and the RRID (SCR_016915) is provided in Subsection "Availability of supporting source code and requirements". (Please refer to Line 11, Page 35 in the revised manuscript.) In addition, Reviewer 1 pointed out that the resolution of Figure 4 was poor. However, we had uploaded Figure 4 to the submission system as a high-resolution "jpg" file, but it appears that the resolution decreased when the "jpg" file was converted into a "pdf" file by the submission system. So, we would like to ask for your help to solve this problem when the manuscript is published. If the file needs to be reformatted, please kindly let us know.

We then report our revisions and responses to three reviewers' all comments (*italic text*) one by one as follows:

To Reviewer #1:

General Comments:

In the revised version of their manuscript, the Authors have adequately addressed all points that I had raised on the previous version. The manuscript is now clearer and easier to read. The change to the algorithm, mostly removal of FNN part makes the software and idea clearer. Moving in scan window across longer sequences is also a great idea and I see great possibilities with that approach for identification of prophages or chromosome-derived fragments on plasmids and phage genomes. An interesting addition is also a part describing virome and plasmidome of the digestive tract, and such analysis can be further expanded into a separate manuscript.

We are glad to see that our previous responses were adequately addressed all the points raised by Reviewer 1. The positive comment "*Moving in scan window across longer sequences is also a great idea and I see great possibilities with that approach for identification of prophages or chromosome-derived fragments on plasmids and phage genomes*" is really encouraging. Additionally, the further comments raised by Reviewer 1 have helped us further improve the quality of the manuscript. We would like to greatly thank Reviewer 1 for his/her careful reading of our manuscript. Below, we itemize our revisions in response to Reviewer 1's points.

1. On page. 33, line 15: should be Error Rate at Read Start.

We thank Reviewer 1 for noting this spelling mistake. The phrase "Error Rate at Read Star" has been revised to "Error Rate at Read Start". (Please refer to the Section "Methods", Line 17, Page 33 in the revised manuscript.)

2. I think Figure 4 should be presented in better resolution.

Herein we apologize for the low resolution of Figure 4. We had uploaded Figure 4 to the submission system as a high-resolution "jpg" file, but it appears that the resolution decreased when the "jpg" file was converted into a "pdf" file by the submission system. We believe that Editor can help us solve this problem when the manuscript is published.

3. Please provide versions of software used, if possible (missing for most of the programs).

We thank Reviewer 1 for reminding us to add the versions of the software programs used in the manuscript. In the revised manuscript, all of this information has been provided now. Specifically, in Subsection "Dataset construction", Line 17, Page 7, we have added the version of ProphET to the sentence: "Here, we used ProphET (v0.5.1) to extract prophages from all the prokaryote chromosomes…"; in Subsection "Dataset construction", Line 7, Page 8, we have added the version of MetaSim to the sentence: "We used the MetaSim (v0.9.1) simulator to extract artificial contigs from the complete genomes"; in Subsection "Dataset construction", Line 18, Page 8, we have added the version of SPAdes to the sentence: "…which were downloaded from MG-RAST (Accessions: mgm4534202.3 and mgm4534203.3) as raw reads and assembled by SPAdes

(v3.11.1)"; and in Subsection "Performance comparison", Lines 18-19, Page 14, we have added the versions of VirFinder, VirSorter, PlasFlow and cBar to the sentence: "We then compare PPR-Meta with VirFinder (v1.1) and VirSorter (v1.0.3) regarding the ability to identify phages, and with PlasFlow (v1.1) and cBar (v1.2) regarding the ability to identify plasmids".

4. Unfortunately, I can't agree with the statement: "Recently, many basecalling tools for the third-generation sequencing technology have been developed to help improve the accuracy over 99% [41], therefore the extremely high error rate on the raw data will not affect the usage of PPR-Meta" (page 19). Authors correctly refer to work by Wick et al, recently published as a preprint (https://doi.org/10.1101/543439), but accuracy over 99% is not a raw read accuracy, but rather consensus accuracy, where reads are first assembled into contigs, then polished using dedicated software. This sentence should be changed to something like (this is only the suggestion): Recently, many dedicated tools have been developed to help improve the consensus accuracy for the third-generation sequencing technology over 99% [41], therefore the extremely high error rate on the raw data should not affect the usage of PPR-Meta on assembled 3rd generation sequences.

We are grateful to Reviewer 1 for noting this incorrect statement, which helped us greatly improve the rigour of the manuscript. We have revised this statement according to the suggestion of Reviewer 1 as follows: "Recently, many dedicated tools have been developed to help improve the consensus accuracy for the third-generation sequencing technology over 99%, therefore the extremely high error rate on the raw data should not affect the usage of PPR-Meta on assembled 3rd generation sequences." (Please refer to Subsection "Performance in the presence of sequencing errors", Lines 13-17, Page 19, in the revised manuscript.)

5. In the case of the sentence: "PPR-Meta can also handle data from the third-generation sequencing technology, although it is designed primarily for the next-generation sequencing technology" (page 24): Isn't 3rd generation also the next-generation? Authors should consider changing "next-generation" to "2nd generation" or 3rd generation to single-molecule

We appreciate Reviewer 1 for noting that we have confused certain definitions related to the sequencing technology. Following the suggestion of Reviewer 1, this sentence has been revised as follows: "Therefore, PPR-Meta can also handle data from the third-generation sequencing technology, although it is designed primarily for the second-generation sequencing technology." (Please refer to Subsection "Evaluation in real metagenomic data", Line 17, Page 24, in the revised manuscript.)

6. The sentence "In the other hand, in order to identify sequences from low-abundance phages, which may fall into binning, we also need tools that can directly judge each fragment." (page 5) needs rephrasing.

Herein, we thank Reviewer 1 for reminding us to rephrase the sentence in a more appropriate

way. To clarify the sentence, we have revised it as follows: "In the other hand, in order to identify sequences from low-abundance phages, which may not fall into bins, tools that can directly judge each fragment are also needed." (Please refer to Section "Introduction", Line 14-16, Page 5 in the revised manuscript.) We confirm that "**not** fall into bins" is the meaning that we want to express and we apologize for missing an important word in this sentence.

To Reviewer #2:

We are pleased to hear that we have properly addressed all of the concerns raised by Reviewer 2. We especially thank Reviewer 2 for the extremely careful reading of our manuscript, and the new comments raised by Reviewer 2 are worthy of attention. Our responses to Reviewer 2's comments are as follows.

General Comments:

1. Regarding this statement in authors' response and similar statements in the manuscript (pg 24 and 25): "We found that in the position closer to the outer end of the digestive tract, the percentages of phages and plasmids tended to be higher. For example, in the gut, the inner end of the digestive tract, the percentages of phages and plasmids were lower; in the oral cavity, the outer end of the digestive tract, the percentages of phages and plasmids were higher."

It seems that authors' conclusion that "percentage of phage and plasmids are higher in the oral cavity" is only based in counts and proportions of contigs found in each one of these environments. However, abundance estimates would be more appropriate if based on raw reads counts. There are several factors which may affect assembly efficiency, and some (if not most) are not dependent on the total abundance of an organism in the sample. Presence of repeats are one good example which may prevent assembly. All of that said to suggest an approach like mapping raw reads to contigs predicted as phages and plasmids and calculating proportions of the total. Or counts based in total number of bases instead of absolute sequence counts.

Herein, we understand the consideration of Reviewer 2 that estimating the abundance by contig counts may has bias. Indeed, in addition to organism abundance, other factors might also affect the assembly efficiency. When the assembly performance is poor, there may be a large number of short fragments, and therefore, the higher contig percentage of phages and plasmids in the throat and oral cavity might result from the poor assembly rather than the higher abundance of phages or plasmids. To test whether the higher contig percentage of phages and plasmids in the throat and oral cavity results from a poor assembly, we calculated the average length of phage and plasmid contigs predicted by PPR-Meta in each sample from the gut, throat and oral cavity. The results show that the average length of phage contigs from the gut, throat and oral cavity was 732.4, 701.1 and 747.0 bp, respectively, and the average length of plasmid contigs from the gut, throat and oral cavity was 838.7, 959.6 and 962.4 bp, respectively. As shown, the average length of phage and plasmid contigs

in the oral cavity was not shorter than that in the gut and throat, which indicated that the sequence assembly performance was comparable among the gut, throat and oral cavity samples. These results also show that the higher percentage of phage and plasmid contigs in the outer end of the digestive tract was likely caused by the higher abundance of phages and plasmids rather than a poor sequence assembly. Furthermore, the results mentioned above also show that in the outer end of the digestive tract, the average lengths of phage and plasmid contigs were generally longer than those in the inner end, and these results were particularly notable for plasmid contigs. This finding further supports our statement that "in the position closer to the outer end of the digestive tract, the percentage of phages and plasmids tended to be higher" because reads from highly abundant organisms are more easily assembled into long contigs. Moreover, all the data from the Human Microbiome Project (HMP) were generated using a similar protocol, such as a similar sequencing depth, which had significant influences on the sequence assembly; thus, the quality of the data from different samples might be similar. We believe that this uniformity can also ensure the reliability of our analysis and conclusion.

Of course, if we want to learn more about the functions of phages and plasmids in the human digestive tract, we need more detailed analyses that can be expanded into future work. In the current study, the main purpose of the analysis was to illustrate how PPR-Meta can promote research on phages and plasmids.

2. I tried to access the website provided in the manuscript and it did not work for me. Please, be careful about links: http://cqb.pku.edu.cn/ZhuLab/PPR_Meta/

We thank Reviewer 2 for visiting the website of our tool. We would like to apologize for that the website could not be accessed then, which might be the server maintenance at that time. We have double-checked the website and will keep trying our best to make it accessible. Besides, our tool is stored on our website, GitHub and the GigaScience database, and thus, users can also download the software if our website is under maintenance. The links to our website and GitHub page are provided in the Subsection "Availability of supporting data". (Please refer to Lines 2-3, Page 35 in the revised manuscript.)

3. I respectfully disagree with review #3 regarding virtual machines being an "odd choice and not at all standard in bioinformatics". I would say that this kind of approach has become more and more common in bioinformatics, specially for very complex software that require lots of dependencies. I work with keras and Tensor Flow, and as a matter of fact they are not easy to set up libraries. Virtual machines or docker containers are very practical way of running tools by people without specialized knowledge. Moreover, Virsorter (which is one of the tools that predict phage and prophages in contigs) also has a docker container, which is very useful. Nonetheless, I also appreciate the gold standard github availability, and that authors decreased VM size.

Herein we thank Reviewer 2 for this helpful discussion about how to release a bioinformatic

tool. Our PPR-Meta can run on both a physical host and a virtual machine. The advantage of running PPR-Meta on virtual machine is that the virtual machine is very easy to install by non-computer professionals because it does not require the installation of any dependent packages. The PPR-Meta manual provides detailed explanation on how both versions of the tool can be run. Therefore, users can conveniently choose which version to use according to their situation.

To Reviewer #3:

We are very pleased to note that Reviewer 3 recommends that this paper be accepted for publication. We think that the revisions made according to Reviewer 3's previous comments have made the manuscript more convincing. Below, we provide our responses to Reviewer 3's further comments.

General Comments:

1. I brought up that I thought the files should be distributed over a more conventional means, like GitHub. I still have concerns about the requirement to download an entire VM. However, given that the journal's author guidelines specify allowing a virtual machine, I think the authors have acted in the best possible way. I appreciate the authors willingness to further compress the download, from 30 to 2.5 Gb in size. While the program is still quite large, it is much less unwieldy.

Herein we understand the concern of Reviewer 3 regarding the downloading of an entire VM. Our PPR-Meta can run on both a physical host and a virtual machine. The advantage of running PPR-Meta on virtual machine is that the virtual machine is very easy to install by non-computer professionals because it does not require the installation of any dependent packages. If users choose to run PPR-Meta on a physical host, they do not need to download the virtual machine; instead, they only need to download a small package from our website or GitHub. The PPR-Meta manual provides detailed explanations on running both versions of the tool. Therefore, users can conveniently select which version to use according to their own situation.

With respect to the size of the virtual machine, it appears that 2.5 GB is the minimum size that we can achieve, and we apologize for being unable to further reduce the size. Although downloading the virtual machine might take some time, running PPR-Meta on a virtual machine might still be the best approach for non-computer professionals because it can save more time when installing the tool.

2. In my comments, I addressed issues with the detection of phage vs. prophage vs. plasmid - in particular the reliance on phage and plasmid databases and the lack of experimentally verified data, particularly, the lack of genomic islands. The authors updated their analysis, including more phage/plasmid manually curated data, and data on genomic islands. They also showed the performance of these in comparison. This reanalysis and discussion assuages my concerns about

the limitations in the analysis.

We are glad to see that our new analysis and discussion have been addressed Reviewer 3's concerns. We believe that this analysis indicates that PPR-Meta can also identify genomic islands, which might be caused by phages or plasmids, and this information makes our manuscript more convincing.

3. The justification for Figure 1 and the elimination of the original Figure 2 sufficiently addressed my concerns that the motivation and description of the FNN were insufficient. I appreciate that the authors show all the changes that resulted from these changes.

Again, we are pleased to hear that our new description sufficiently addressed Reviewer 3's concerns regarding the motivation of the design of the neural network. Additionally, we believe that the new description provides insights into the neural network design strategy for readers.

4. Many of the issues from this comment were taken into account in comment #2 and I appreciate the full description in the authors' responses.

We thank Reviewer 3 for this comment from the last report, and we are happy to note that the issues raised in this comment have been addressed.

5. The authors went above-and-beyond what was required by including the HMP data. This is a major improvement to the generalizability of the analysis. I still believe that the second part hasn't been sufficiently addressed in the manuscript, but has in the comments. Having a robust justification for why the authors believe PPR-Meta is keying on sequence signatures and what the implications of that are, may warrant subsequent work.

Herein, we are grateful for the positive comment provided by Reviewer 3 noting that the identification of phage and plasmid sequences from HMP data is a major improvement to the generalizability of the analysis. Additionally, we agree with Reviewer 3 that subsequent work is needed to prove why PPR-Meta is keying on sequence signatures. In the previous response, we explained this issue intuitively, primarily by comparing the performances of PPR-Meta and VirSorter, which is not a sequence-signatures-based tool. Related statements about tool keying on larger gene-level issues may not work well in metagenomic fragments were also emphasized in our manuscript. For example, in Section "Discussion and conclusions", Line 20, Page 27 to Line 1, Page 28, we mentioned that "**Similarity search-based tools, such as VirSorter, provide good results for long sequences. However, such methods do not work effectively for short fragments due to the insufficient number of genes for the statistical analysis.**" Although we cannot provide a more quantitative interpretation at this time, we would like to state that neural networks have been widely used to analyse biological sequence and that neural networks have shown a strong ability to extract sequence patterns or sequence motifs (for more details, see the following reviews: [1] Jones W. et al. (2017). Computational biology: deep learning. *Emerging Top. Life Sci.* [2] Min S. et al. (2017).

Deep learning in bioinformatics. *Briefings Bioinf.*). Therefore, we think that it is not strange that PPR-Meta can bypass the genetic information of larger gene-level issues and directly identify phage and plasmid sequences by observing the sequence signatures.

In hoping that the above revision has clarified all the points by three reviewers and given a point-by-point response to all the concerns, we hereby resubmit our manuscript to the journal. We thank you for your kind consideration.

Sincerely yours,
Huaiqiu Zhu, Ph. D., Professor
Peking University
