# Supplementary material for: PPR-Meta: a tool for identifying phages and plasmids from metagenomic fragments using deep learning
Source: Gigascience. 2019 Jun 20;8(6):giz066. doi: 10.1093/gigascience/giz066 (PMC6586199; doi:10.1093/gigascience/giz066)
Supplement: giz066_Supplemental_Files [file giz066_supplemental_files.zip › Additional file 3.docx]

**Figure S1 to Figure S5**

**Figure S1. Comparison of the performance of PPR-Meta and related tools using artificial contigs of 15k bp and 30k bp.**

**
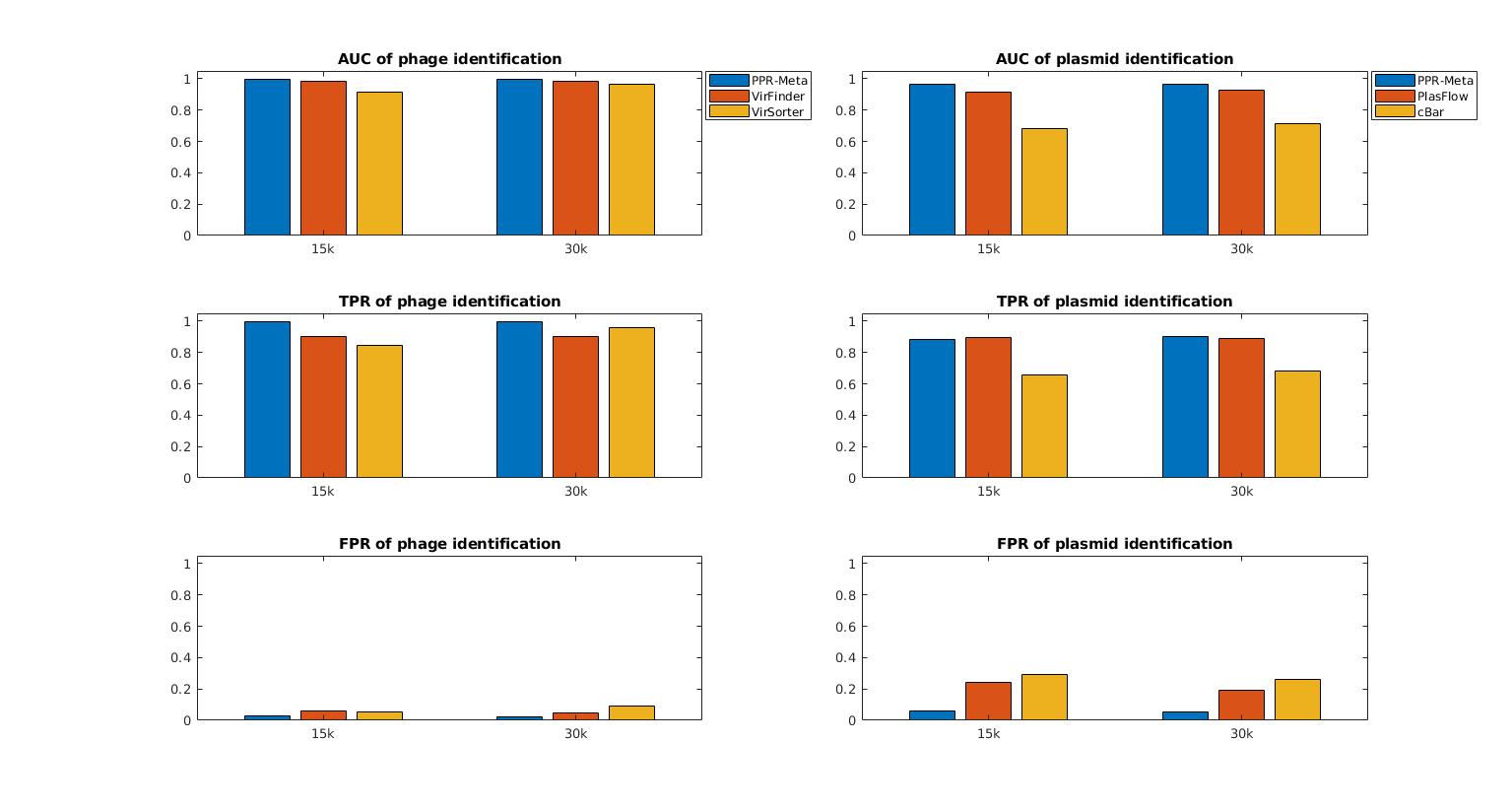
**

Although most fragments in the current metagenomic data are short, a few reads from high-abundance species can be assembled into long contigs, such as contigs longer than 10k bp. To further evaluate how PPR-Meta and related tools perform on these long sequences, we used artificial contigs of 15k bp and 30k bp generated from the test genomes. Compared with the performance on short sequences, as mentioned in the main text, most tools had better performance on these long sequences. PPR-Meta is still the best performing tool.

**Figure S2. Evaluation of the accuracy and running time of each group using BiPathCNNs from different groups.**


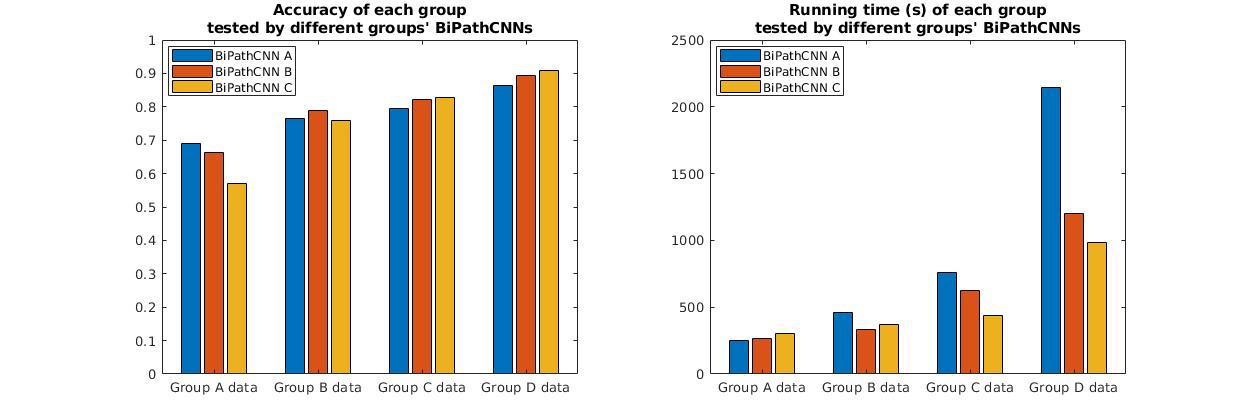


We evaluated the accuracy and running time of each group using BiPathCNNs from different groups (e.g., used BiPathCNN A to test sequences from Group A to Group D; used BiPathCNN B to test sequences from Group A to Group D). There were 90000 sequences in Group A, B and C while there were 30000 sequences in Group D. The result showed that the accuracy might be lower and the running time might be longer if a non-corresponding BiPathCNN was used to predict the sequences. Note that the input size of each BiPathCNN is fixed. The lengths of the input one-hot matrix for BiPathCNN A to C is 800, 1600 and 2400, respectively (twice the maximum single-strand length in each group). If the BiPathCNN for a long sequence is used to predict a shorter sequence, the input one-hot matrix should be padded with rows whose bits are all zeros to adapt the input size of the BiPathCNN, which will increase the unnecessary calculations, and therefore, the running time will be longer. If the BiPathCNN for a short sequence is used to predict a long sequence, the long sequence must be split into several short sequences whose lengths can adapt the input size of the BiPathCNN. Each subsequence will be predicted separately, and then an average score will be calculated for the whole sequence. Since the total number of sequences is increasing, the running time will also be longer. Therefore, constructing corresponding BiPathCNN for a specific group can both improve the accuracy and speed up the program.

**Figure S3. Identification performance of each tool with 10% base substitutions or 10% indels (insertions or deletions) using sequences in Group D.**

**
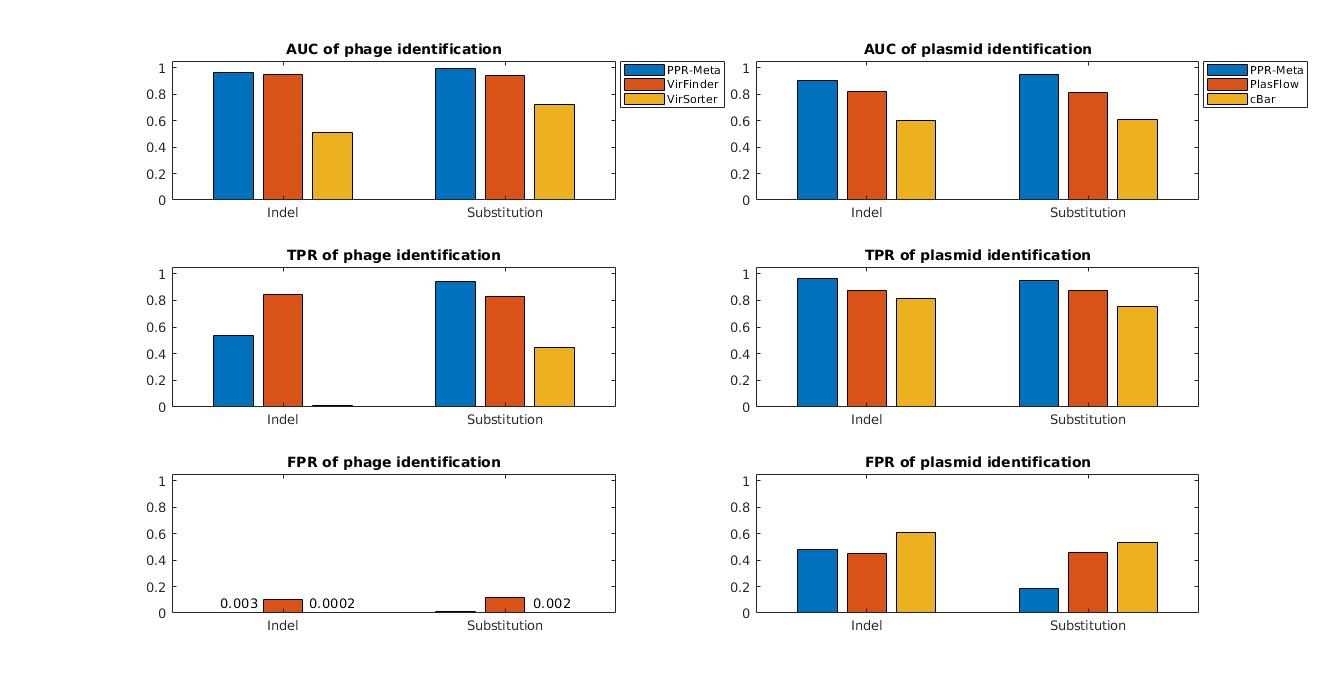
**

We evaluated PPR-Meta and the related tools using artificial contigs modified with 10% substitutions and indels. Although there were fluctuations in the performance of PPR-Meta under 10% indels, the AUCs of PPR-Meta were still the highest. Although the error rate of the third-generation sequencing technology can be as high as 10%, many basecalling tools have been developed to help improve the accuracy, as we mentioned in the main text. Therefore, we consider that PPR-Meta can handle data generated from the third-generation sequencing technology.

**Figure S4. Recognition rate of prophages from** **PHANTOME database.**


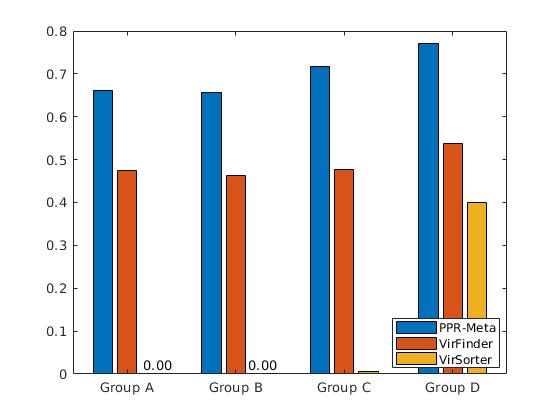


We evaluated the prophage recognition rate of PPR-Meta, VirFinder and VirSorter using artificial contigs from prophages in the PHANTOME database. These prophages were first predicted by PhiSpy and had been manually verified. The results showed that PPR-Meta had the best prophage recognition ability.

**Figure S5. Performance of PPR-Meta under different thresholds.**

**
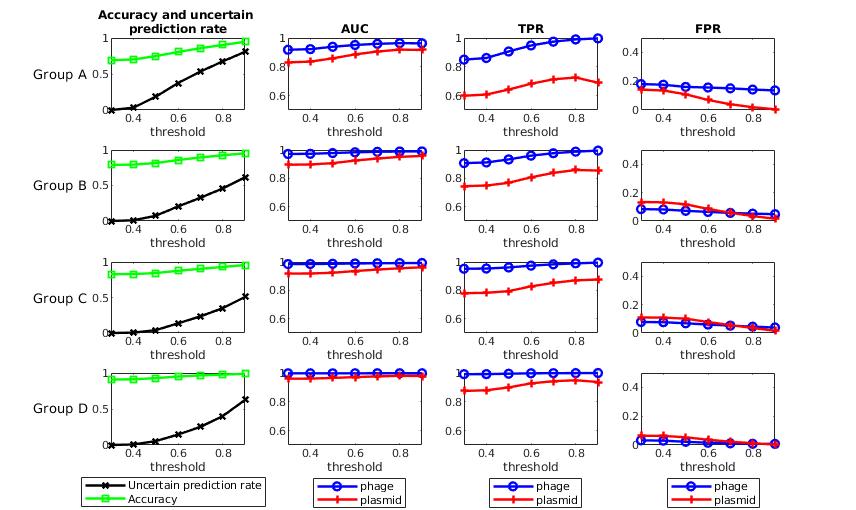
**

Given a threshold, the sequences with the highest scores (among phages, chromosomes and plasmids) that are lower than the threshold will be labelled as uncertain predictions by PPR-Meta. In general, with a higher threshold, the number of uncertain predictions will be higher, but the remaining predictions will be more reliable. We evaluate the uncertain prediction rate, accuracy, AUC, TPR and FPR of PPR-Meta under different thresholds. The accuracy is defined as the ratio of correct predictions to all test sequences, so that it reflects the overall performance of PPR-Meta. The accuracy, AUC, TPR and FPR were calculated only on the certain predictions.
