# Supplementary material for: PPR-Meta: a tool for identifying phages and plasmids from metagenomic fragments using deep learning
Source: Gigascience. 2019 Jun 20;8(6):giz066. doi: 10.1093/gigascience/giz066 (PMC6586199; doi:10.1093/gigascience/giz066)
Supplement: giz066_Response_to_Reviewer_Comments_Revision_1 [file giz066_response_to_reviewer_comments_revision_1.pdf]

Author's Response To Reviewer Comments

Close

Cover Letter

Dear Editor,

Thank you very much for your previous E-mail on April 6, 2019 regarding our manuscript ‵‵PPR-Meta: a tool for identifying phages and plasmids from metagenomic fragments using deep learning′′ (Manuscript ID: GIGA-D-18-00464R1). We are very pleased to know that our manuscript is potentially acceptable for publication in the journal, subject to the suggested further revision following the reviewers. Herein we would like to thank three reviewers and are pleased to know that our previous responses were addressed all of their concerns. Additionally, we are grateful for their careful reading of the previous version of our manuscript, and the further comments raised by three reviewers helped us make further improvements. In the revised manuscript, all the changed words, sentences and paragraphs are marked in red text.

Following your instruction and Reviewers' comments, this time we have made a conscious effort to revise the manuscript with the further improvement. Before we report the revisions and responses to Reviewers' comments, we would like to first present our responses to Editor's instructions. With respect to the website, because the server is regularly maintained, users might be occasionally unable to access it when the server is under maintenance. Therefore we have double-checked our website (http://cqb.pku.edu.cn/ZhuLab/PPR_Meta/), and we confirm that the website is working. Additionally, our tool is stored on our website, as well as GitHub and the GigaScience database, and thus, users can also download the software if our website is under maintenance. The links to our website and the GitHub page are provided in Subsection "Availability of supporting source code and requirements". (Please refer to Lines 2-3, Page 35 in the revised manuscript.) Also, we have already registered PPR-Meta in the SciCrunch.org database, and the RRID (SCR_016915) is provided in Subsection "Availability of supporting source code and requirements". (Please refer to Line 11, Page 35 in the revised manuscript.) In addition, Reviewer 1 pointed out that the resolution of Figure 4 was poor. However, we had uploaded Figure 4 to the submission system as a high-resolution "jpg" file, but it appears that the resolution decreased when the "jpg" file was converted into a "pdf" file by the submission system. So, we would like to ask for your help to solve this problem when the manuscript is published. If the file needs to be reformatted, please kindly let us know.

We then report our revisions and responses to three reviewers' all comments (italic text) one by one as follows:

To Reviewer #1:

General Comments:
In the revised version of their manuscript, the Authors have adequately addressed all points that I had raised on the previous version. The manuscript is now clearer and easier to read. The change to the algorithm, mostly removal of FNN part makes the software and idea clearer. Moving in scan window across longer sequences is also a great idea and I see great possibilities with that approach for identification of prophages or chromosome-derived fragments on plasmids and phage genomes.
An interesting addition is also a part describing virome and plasmidome of the digestive tract, and such analysis can be further expanded into a separate manuscript.
We are glad to see that our previous responses were adequately addressed all the points raised by Reviewer 1. The positive comment "Moving in scan window across longer sequences is also a great idea and I see great possibilities with that approach for identification of prophages or chromosome-derived fragments on plasmids and phage genomes" is really encouraging. Additionally, the further comments raised by Reviewer 1 have helped us further improve the quality of the manuscript. We would like to greatly thank Reviewer 1 for his/her careful reading of our manuscript. Below, we itemize our revisions in response to Reviewer 1's points.

1. On page. 33, line 15: should be Error Rate at Read Start.
We thank Reviewer 1 for noting this spelling mistake. The phrase “Error Rate at Read Star” has been revised to “Error Rate at Read Start”. (Please refer to the Section “Methods”, Line 17, Page 33 in the revised manuscript.)

2. I think Figure 4 should be presented in better resolution.
Herein we apologize for the low resolution of Figure 4. We had uploaded Figure 4 to the submission system as a high-resolution “jpg” file, but it appears that the resolution decreased when the “jpg” file was converted into a “pdf” file by the submission system. We believe that Editor can help us solve this problem when the manuscript is published.

3. Please provide versions of software used, if possible (missing for most of the programs).
We thank Reviewer 1 for reminding us to add the versions of the software programs used in the manuscript. In the revised manuscript, all of this information has been provided now. Specifically, in Subsection “Dataset construction”, Line 17, Page 7, we have added the version of ProphET to the sentence: “Here, we used ProphET (v0.5.1) to extract prophages from all the prokaryote chromosomes…”; in Subsection “Dataset construction”, Line 7, Page 8, we have added the version of MetaSim to the sentence: “We used the MetaSim (v0.9.1) simulator to extract artificial contigs from the complete genomes”; in Subsection “Dataset construction”, Line 18, Page 8, we have added the version of SPAdes to the sentence: “…which were downloaded from MG-RAST (Accessions: mgm4534202.3 and mgm4534203.3) as raw reads and assembled by SPAdes (v3.11.1)”; and in Subsection “Performance comparison”, Lines 18-19, Page 14, we have added the versions of VirFinder, VirSorter, PlasFlow and cBar to the sentence: “We then compare PPR-Meta with VirFinder (v1.1) and VirSorter (v1.0.3) regarding the ability to identify phages, and with PlasFlow (v1.1) and cBar (v1.2) regarding the ability to identify plasmids”.

4. Unfortunately, I can't agree with the statement: “Recently, many basecalling tools for the third-generation sequencing technology have been developed to help improve the accuracy over 99% [41], therefore the extremely high error rate on the raw data will not affect the usage of PPR-Meta" (page 19). Authors correctly refer to work by Wick et al, recently published as a preprint (https://doi.org/10.1101/543439), but accuracy over 99% is not a raw read accuracy, but rather consensus accuracy, where reads are first assembled into contigs, then polished using dedicated software. This sentence should be changed to something like (this is only the suggestion): Recently, many dedicated tools have been developed to help improve the consensus accuracy for the third-generation sequencing technology over 99% [41], therefore the extremely high error rate on the raw data should not affect the usage of PPR-Meta on assembled 3rd generation sequences.
We are grateful to Reviewer 1 for noting this incorrect statement, which helped us greatly improve the rigour of the manuscript. We have revised this statement according to the suggestion of Reviewer 1 as follows: “Recently, many dedicated tools have been developed to help improve the consensus accuracy for the third-generation sequencing technology over 99%, therefore the extremely high error rate on the raw data should not affect the usage of PPR-Meta on assembled 3rd generation sequences.” (Please refer to Subsection “Performance in the presence of sequencing errors”, Lines 13-17, Page 19, in the revised manuscript.)

5. In the case of the sentence: "PPR-Meta can also handle data from the third-generation sequencing technology, although it is designed primarily for the next-generation sequencing technology" (page 24): Isn't 3rd generation also the next-generation? Authors should consider changing “next-generation" to “2nd generation" or 3rd generation to single-molecule
We appreciate Reviewer 1 for noting that we have confused certain definitions related to the sequencing technology. Following the suggestion of Reviewer 1, this sentence has been revised as follows: “Therefore, PPR-Meta can also handle data from the third-generation sequencing technology, although it is designed primarily for the second-generation sequencing technology.” (Please refer to Subsection “Evaluation in real metagenomic data”, Line 17, Page 24, in the revised manuscript.)

6. The sentence "In the other hand, in order to identify sequences from low-abundance phages, which may fall into binning, we also need tools that can directly judge each fragment." (page 5) needs rephrasing.
Herein, we thank Reviewer 1 for reminding us to rephrase the sentence in a more appropriate way. To clarify the sentence, we have revised it as follows: “In the other hand, in order to identify sequences from low-abundance phages, which may not fall into bins, tools that can directly judge each fragment are also needed.” (Please refer to Section “Introduction”, Line 14-16, Page 5 in the revised manuscript.)
We confirm that “not fall into bins” is the meaning that we want to express and we apologize for missing

an important word in this sentence.

To Reviewer #2:

We are pleased to hear that we have properly addressed all of the concerns raised by Reviewer 2. We especially thank Reviewer 2 for the extremely careful reading of our manuscript, and the new comments raised by Reviewer 2 are worthy of attention. Our responses to Reviewer 2's comments are as follows.

General Comments:
1. Regarding this statement in authors' response and similar statements in the manuscript (pg 24 and 25): "We found that in the position closer to the outer end of the digestive tract, the percentages of phages and plasmids tended to be higher. For example, in the gut, the inner end of the digestive tract, the percentages of phages and plasmids were lower; in the oral cavity, the outer end of the digestive tract, the percentages of phages and plasmids were higher."
It seems that authors' conclusion that "percentage of phage and plasmids are higher in the oral cavity" is only based in counts and proportions of contigs found in each one of these environments. However, abundance estimates would be more appropriate if based on raw reads counts. There are several factors which may affect assembly efficiency, and some (if not most) are not dependent on the total abundance of an organism in the sample. Presence of repeats are one good example which may prevent assembly. All of that said to suggest an approach like mapping raw reads to contigs predicted as phages and plasmids and calculating proportions of the total. Or counts based in total number of bases instead of absolute sequence counts.
Herein, we understand the consideration of Reviewer 2 that estimating the abundance by contig counts may has bias. Indeed, in addition to organism abundance, other factors might also affect the assembly efficiency. When the assembly performance is poor, there may be a large number of short fragments, and therefore, the higher contig percentage of phages and plasmids in the throat and oral cavity might result from the poor assembly rather than the higher abundance of phages or plasmids. To test whether the higher contig percentage of phages and plasmids in the throat and oral cavity results from a poor assembly, we calculated the average length of phage and plasmid contigs predicted by PPR-Meta in each sample from the gut, throat and oral cavity. The results show that the average length of phage contigs from the gut, throat and oral cavity was 732.4, 701.1 and 747.0 bp, respectively, and the average length of plasmid contigs from the gut, throat and oral cavity was 838.7, 959.6 and 962.4 bp, respectively. As shown, the average length of phage and plasmid contigs in the oral cavity was not shorter than that in the gut and throat, which indicated that the sequence assembly performance was comparable among the gut, throat and oral cavity samples. These results also show that the higher percentage of phage and plasmid contigs in the outer end of the digestive tract was likely caused by the higher abundance of phages and plasmids rather than a poor sequence assembly. Furthermore, the results mentioned above also show that in the outer end of the digestive tract, the average lengths of phage and plasmid contigs were generally longer than those in the inner end, and these results were particularly notable for plasmid contigs. This finding further supports our statement that "in the position closer to the outer end of the digestive tract, the percentage of phages and plasmids tended to be higher" because reads from highly abundant organisms are more easily assembled into long contigs. Moreover, all the data from the Human Microbiome Project (HMP) were generated using a similar protocol, such as a similar sequencing depth, which had significant influences on the sequence assembly; thus, the quality of the data from different samples might be similar. We believe that this uniformity can also ensure the reliability of our analysis and conclusion.
Of course, if we want to learn more about the functions of phages and plasmids in the human digestive tract, we need more detailed analyses that can be expanded into future work. In the current study, the main purpose of the analysis was to illustrate how PPR-Meta can promote research on phages and plasmids.

2. I tried to access the website provided in the manuscript and it did not work for me. Please, be careful about links: http://cqb.pku.edu.cn/ZhuLab/PPR_Meta/
We thank Reviewer 2 for visiting the website of our tool. We would like to apologize for that the website could not be accessed then, which might be the server maintenance at that time. We have double-checked the website and will keep trying our best to make it accessible. Besides, our tool is stored on our website, GitHub and the GigaScience database, and thus, users can also download the software if our website is under maintenance. The links to our website and GitHub page are provided in the Subsection "Availability of supporting data". (Please refer to Lines 2-3, Page 35 in the revised manuscript.)

3. I respectfully disagree with review #3 regarding virtual machines being an "odd choice and not at all

standard in bioinformatics". I would say that this kind of approach has become more and more common in bioinformatics, specially for very complex software that require lots of dependencies. I work with keras and Tensor Flow, and as a matter of fact they are not easy to set up libraries. Virtual machines or docker containers are very practical way of running tools by people without specialized knowledge. Moreover, Virsorter (which is one of the tools that predict phage and prophages in contigs) also has a docker container, which is very useful. Nonetheless, I also appreciate the gold standard github availability, and that authors decreased VM size.
Herein we thank Reviewer 2 for this helpful discussion about how to release a bioinformatic tool. Our PPR-Meta can run on both a physical host and a virtual machine. The advantage of running PPR-Meta on virtual machine is that the virtual machine is very easy to install by non-computer professionals because it does not require the installation of any dependent packages. The PPR-Meta manual provides detailed explanation on how both versions of the tool can be run. Therefore, users can conveniently choose which version to use according to their situation.

To Reviewer #3:

We are very pleased to note that Reviewer 3 recommends that this paper be accepted for publication. We think that the revisions made according to Reviewer 3's previous comments have made the manuscript more convincing. Below, we provide our responses to Reviewer 3's further comments.

General Comments:
1. I brought up that I thought the files should be distributed over a more conventional means, like GitHub. I still have concerns about the requirement to download an entire VM. However, given that the journal's author guidelines specify allowing a virtual machine, I think the authors have acted in the best possible way. I appreciate the authors willingness to further compress the download, from 30 to 2.5 Gb in size. While the program is still quite large, it is much less unwieldy.
Herein we understand the concern of Reviewer 3 regarding the downloading of an entire VM. Our PPR-Meta can run on both a physical host and a virtual machine. The advantage of running PPR-Meta on virtual machine is that the virtual machine is very easy to install by non-computer professionals because it does not require the installation of any dependent packages. If users choose to run PPR-Meta on a physical host, they do not need to download the virtual machine; instead, they only need to download a small package from our website or GitHub. The PPR-Meta manual provides detailed explanations on running both versions of the tool. Therefore, users can conveniently select which version to use according to their own situation.
With respect to the size of the virtual machine, it appears that 2.5 GB is the minimum size that we can achieve, and we apologize for being unable to further reduce the size. Although downloading the virtual machine might take some time, running PPR-Meta on a virtual machine might still be the best approach for non-computer professionals because it can save more time when installing the tool.

2. In my comments, I addressed issues with the detection of phage vs. prophage vs. plasmid - in particular the reliance on phage and plasmid databases and the lack of experimentally verified data, particularly, the lack of genomic islands. The authors updated their analysis, including more phage/plasmid manually curated data, and data on genomic islands. They also showed the performance of these in comparison. This reanalysis and discussion assuages my concerns about the limitations in the analysis.
We are glad to see that our new analysis and discussion have been addressed Reviewer 3's concerns. We believe that this analysis indicates that PPR-Meta can also identify genomic islands, which might be caused by phages or plasmids, and this information makes our manuscript more convincing.

3. The justification for Figure 1 and the elimination of the original Figure 2 sufficiently addressed my concerns that the motivation and description of the FNN were insufficient. I appreciate that the authors show all the changes that resulted from these changes.
Again, we are pleased to hear that our new description sufficiently addressed Reviewer 3's concerns regarding the motivation of the design of the neural network. Additionally, we believe that the new description provides insights into the neural network design strategy for readers.

4. Many of the issues from this comment were taken into account in comment #2 and I appreciate the full description in the authors' responses.
We thank Reviewer 3 for this comment from the last report, and we are happy to note that the issues raised in this comment have been addressed.

5. The authors went above-and-beyond what was required by including the HMP data. This is a major

improvement to the generalizability of the analysis. I still believe that the second part hasn't been sufficiently addressed in the manuscript, but has in the comments. Having a robust justification for why the authors believe PPR-Meta is keying on sequence signatures and what the implications of that are, may warrant subsequent work.
Herein, we are grateful for the positive comment provided by Reviewer 3 noting that the identification of phage and plasmid sequences from HMP data is a major improvement to the generalizability of the analysis. Additionally, we agree with Reviewer 3 that subsequent work is needed to prove why PPR-Meta is keying on sequence signatures. In the previous response, we explained this issue intuitively, primarily by comparing the performances of PPR-Meta and VirSorter, which is not a sequence-signatures-based tool. Related statements about tool keying on larger gene-level issues may not work well in metagenomic fragments were also emphasized in our manuscript. For example, in Section "Discussion and conclusions", Line 20, Page 27 to Line 1, Page 28, we mentioned that "Similarity search-based tools, such as VirSorter, provide good results for long sequences. However, such methods do not work effectively for short fragments due to the insufficient number of genes for the statistical analysis." Although we cannot provide a more quantitative interpretation at this time, we would like to state that neural networks have been widely used to analyse biological sequence and that neural networks have shown a strong ability to extract sequence patterns or sequence motifs (for more details, see the following reviews: [1] Jones W. et al. (2017). Computational biology: deep learning. Emerging Top. Life Sci. [2] Min S. et al. (2017). Deep learning in bioinformatics. Briefings Bioinf.). Therefore, we think that it is not strange that PPR-Meta can bypass the genetic information of larger gene-level issues and directly identify phage and plasmid sequences by observing the sequence signatures.

In hoping that the above revision has clarified all the points by three reviewers and given a point-by-point response to all the concerns, we hereby resubmit our manuscript to the journal. We thank you for your kind consideration.

Sincerely yours,
Huaiqiu Zhu, Ph. D., Professor
Peking University

Close
