# Supplementary material for: PPR-Meta: a tool for identifying phages and plasmids from metagenomic fragments using deep learning
Source: Gigascience. 2019 Jun 20;8(6):giz066. doi: 10.1093/gigascience/giz066 (PMC6586199; doi:10.1093/gigascience/giz066)
Supplement: giz066_Reviewer_1_Report_Original_Submission — Pawel Krawczyk -- 12/13/2018 Reviewed [file giz066_reviewer_1_report_original_submission.pdf]

Reviewer Report

Title: PPR-Meta: a tool for identifying phages and plasmids from metagenomic fragments using deep learning

Version: Original Submission **Date:** 12/13/2018

Reviewer name: Pawel Krawczyk

Reviewer Comments to Author:

The article by Fang et al describes a novel tool aimed at simultaneous identification of viral and plasmid sequences in metagenomic datasets. Presented tool can be of great value to many researchers analyzing microbiome data, especially for those interested in viral communities and/or mobile elements involved in the AMR spread and may as such also be widely cited. Authors used the novel neural network approach, which improves sequence classification for shorter sequences over other existing tools. Especially, usage of both noncoding and coding information is an important improvement over other existing tools, what authors clearly show in their manuscript.

I find the manuscript well written and informative, the methods are appropriate and analysis is well conducted. However, I do have several not too difficult to accomplish, requests, as well as comments and questions, before I can recommend the manuscript for publication.

Authors do not state what is the exact target of their software. Should it be used mainly with the metagenomic assemblies or can it be also easily applied to raw sequencing reads (because one of the models used in the work was trained on relatively short sequences (100-400 bp))? How will it work with isolate genome sequences?

Although the training procedure and the algorithm itself are quite well described I missed some details regarding data preprocessing and preparation of models. What parameters were used in MetaSim to generate sequences used for training? Authors should note that MetaSim does not produce artificial contigs but synthetic sequencing reads, with technology-specific errors introduced. Did authors use 'exact' preset to return fragments perfectly matching reference sequences?

It would be also great if all the codes and scripts (e.g. those for preprocessing of sequences, and neural network training) are available online or as Supplemental materials.

Have authors tried to build model similar to BiPathCNN for longer sequences? As authors claim that codon path is beneficial for distinguishing plasmids, phages and chromosomes (p. 16, lines 7-10), this additional information should increase kmer-based approach, especially that longer fragments are more likely to contain coding sequences.

I also wonder why authors chose the hexamer frequencies and not any odd-number kmer?

I find really interesting part in which authors used likelihood scores generated by PPR-Meta to predict phage lifestyle or plasmid transmissability. Results shown are really encouraging and may make PPR-Meta an important tool in MGE studies. In the discussion of this phenomenon authors should cite the paper of Suzuki et al. (https://www.ncbi.nlm.nih.gov/pmc/articles/PMC2976448/), which discusses usage of genome signatures to predict the evolutionary host range of plasmids. Additionally, similarity between host and plasmid nucleotide composition is a known phenomenon, and so called genome

amelioration is a term properly describing convergence of plasmid and host sequence patterns. Therefore, the statement "Since temperate phages and non-transmissible plasmids experience longer residence times within the host cell, they may adjust the sequence pattern toward the host" seems correct.

For me it is really worth emphasizing that authors tested their software in the context of 3rd generation sequencing technologies like PacBio and Nanopore. As there is more and more reports showing applicability of single molecule sequencing technologies for describing microbial communities it would be really great to have tool which which is tested and works well on such datasets. Authors used 1% error rates for their simulated datasets, however, in my opinion they should test also higher error rates, as for aforementioned technologies they can reach up to 10%, error rate, especially for indels. Both technologies are sensitive to homopolymer sequences therefore one would expect more errors in such regions. Additionally I would also like to see if PPR-Meta is able to predict phage/plasmid sequences on real 3rd generation sequencing data. They can use for example recently published virome (https://www.biorxiv.org/content/early/2018/11/12/345041.full.pdf+html) or mock microbial community (https://www.biorxiv.org/content/early/2018/12/04/487033.full.pdf+html) nanopore data. The former one can also help with "Since we lack samples in which only chromosomes are enriched and all the extrachromosomal elements are filtered, estimating whether related tools will
misjudge chromosomes as MGEs directly is difficult using real data" (p. 21, l. 9-11)

It should be also noted that in case of plasmid prediction PPR-Meta presents different approach than previously published cBar and PlasFlow only for sequences shorter than 10kb, as neural network trained on Group D essentially use the 6-mer frequencies for prediction. Additionally in the PlasFlow manual it is said that such approach does not work well on short sequences (recommended length is > 1kb), therefore comparisons using test datasets from GroupA and GroupB should be done with having this in mind. In my opinion authors should also comment on different lengths of sequences used for training in cBar, PlasFlow and PPR-Meta: cBar was trained with whole chromosome/plasmid sequences whereas PlasFlow on 10kb fragments and PPR-Meta using 4 datasets with differing lengths, what may significantly influence their performance.

Specific comments:

Page 5 lines 2-4:

"extracted" should be "extract"

Page 5 line 21: Authors may also cite following tool: https://www.ncbi.nlm.nih.gov/pubmed/30383524,

Page 6 line 6 "this tool applies SMO" should be changed to: "this tool applies SOM" (Self Organizing Map)

page 8 lines 13-19 "phage metagenomic data of bovine rumen [19], which were downloaded from MG-RAST [29] (Accessions: mgm4534202.3 and mgm4534203.3) as raw reads and assembled by SPAdes"

and

"20 samples of healthy human gut [32], downloaded from the NCBI Short Read Archive [33] and assembled by SPAdes."

I miss details on SPAdes assembly. What settings were used and what was the quality of assembly (N50, number of contigs and so on).

page 12 lines 5-11

Which approach is used for sequences 5-10 kb? FNN or biPath-CNN (groupC model)? If biPath-CNN -

what is its performance on this dataset compared to FNN (group D)? It is not clear which approach the software will use for the real datasets of this length.
I would also like to see the comparison to other software regarding fragments longer than 10kb (which are easily achievable with current metagenomic sequencing techniques).
Additionally, please test accuracy of each model on testing datasets from other models, e.g. model for group A on test datasets for groups B, C and D, model for group B on test datasets for groups A, C and D, etc. It is not likely that in real datasets sequences will be distributed such uniformly. It is also interesting how biPath-CNN performs on long sequences, as coding information should significantly increase its performance (like it was shown for shorter fragments). And, maybe, any of the single models is good enough to be used on fragments of all lengths; for me this possibility can not be excluded by looking at presented data.
Page 15 line 12
I would remove the word "obviously". Please be less advertising and more informative.
page 16 lines 10-13
"Compared with other sequence representation methods that ignore the coding or non-coding region, such as method based on k-mer frequencies, PPR-Meta uses a more detailed method of describing a sequence and achieves a higher performance."
Authors should explicitly note that it relates only to sequences shorter than 5(or 10, see my note above) kb.
Page 22, lines 16-17
"PPR-Meta is designed with the option to adjust the default threshold of discriminant criteria"
It should be described more precisely. Although in the Manual it is noted, that "In this way, sequences with the phage (or plasmid) score higher than the other two categories and the threshold will be regarded as phage (or plasmid)", it is not mentioned that sequences not exceeding threshold for phage or plasmid category will fall into the "chromosome" category, what may not be the best option, increasing False Negative Rate. I also lack more information on the accuracy of PPR-Meta run with different thresholds (Table S1). Can you include also AUC in the table? And compare with PlasFlow, using the same thresholds?
This is only the suggestion, but all the data presenting performance of PPR-Meta in comparison to other tools can be also presented as graphs, what would allow for easy assessment of differences.
Specific comments regarding software usaibility:
Using output file extension other than .csv throws an error:
Error using writetable (line 124)
Unrecognized file extension '.tsv'. Use the 'FileType' parameter to specify the file type
Error in PPR-Meta(line 124)
MATLAB:table:write:UnrecognizedFileExtension
This should be better documented, and the user should be warned at the beginning of computation that using custom file extensions will cause that output file cannot be written.
Again, I find your work really interesting and solid and I hope I managed to help you improve the manuscript in some way. Good luck with other review!

Level of Interest

Please indicate how interesting you found the manuscript: Choose an item.

Quality of Written English

Please indicate the quality of language in the manuscript: Choose an item.

Declaration of Competing Interests

Please complete a declaration of competing interests, considering the following questions:

- Have you in the past five years received reimbursements, fees, funding, or salary from an organisation that may in any way gain or lose financially from the publication of this manuscript, either now or in the future?
- Do you hold any stocks or shares in an organisation that may in any way gain or lose financially from the publication of this manuscript, either now or in the future?
- Do you hold or are you currently applying for any patents relating to the content of the manuscript?
- Have you received reimbursements, fees, funding, or salary from an organization that holds or has applied for patents relating to the content of the manuscript?
- Do you have any other financial competing interests?
- Do you have any non-financial competing interests in relation to this paper?

If you can answer no to all of the above, write 'I declare that I have no competing interests' below. If your reply is yes to any, please give details below.

I'm the author of PlasFlow, one of the tools compared to PPR-Meta in the manuscript. I declare that I have no other competing interests.

I agree to the open peer review policy of the journal. I understand that my name will be included on my report to the authors and, if the manuscript is accepted for publication, my named report including any attachments I upload will be posted on the website along with the authors' responses. I agree for my report to be made available under an Open Access Creative Commons CC-BY license (http://creativecommons.org/licenses/by/4.0/). I understand that any comments which I do not wish to be included in my named report can be included as confidential comments to the editors, which will not be published.

I agree to the open peer review policy of the journal

To further support our reviewers, we have joined with Publons, where you can gain additional credit to further highlight your hard work (see: https://publons.com/journal/530/gigascience). On publication of this paper, your review will be automatically added to Publons, you can then choose whether or not to claim your Publons credit. I understand this statement.

Yes Choose an item.
