# Supplementary material for: PPR-Meta: a tool for identifying phages and plasmids from metagenomic fragments using deep learning
Source: Gigascience. 2019 Jun 20;8(6):giz066. doi: 10.1093/gigascience/giz066 (PMC6586199; doi:10.1093/gigascience/giz066)
Supplement: giz066_Reviewer_1_Report_Revision_1 — Pawel Krawczyk -- 4/1/2019 Reviewed [file giz066_reviewer_1_report_revision_1.pdf]

Reviewer Report

Title: PPR-Meta: a tool for identifying phages and plasmids from metagenomic fragments using deep learning

Version: Revision 1 **Date:** 4/1/2019

Reviewer name: Pawel Krawczyk

Reviewer Comments to Author:

In the revised version of their manuscript, the Authors have adequately addressed all points that I had raised on the previous version. The manuscript is now clearer and easier to read. The change to the algorithm, mostly removal of FNN part makes the software and idea clearer. Moving in scan window across longer sequences is also a great idea and I see great possibilities with that approach for identification of prophages or chromosome-derived fragments on plasmids and phage genomes.
An interesting addition is also a part describing virome and plasmidome of the digestive tract, and such analysis can be further expanded into a separate manuscript.
I still have some cosmetic comments regarding manuscript:
1. On page. 33, line 15: should be Error Rate at Read Start
2. I think Figure 4 should be presented in better resolution.
3. Please provide versions of software used, if possible (missing for most of the programs)
4. Unfortunately, I can't agree with the statement: â€žRecently, many basecalling tools for the third-generation sequencing technology have been developed to help improve the accuracy over 99% [41], therefore the extremely high error rate on the raw data will not affect the usage of PPR-Meta" (page 19). Authors correctly refer to work by Wick et al, recently published as a preprint (https://doi.org/10.1101/543439), but accuracy over 99% is not a raw read accuracy, but rather consensus accuracy, where reads are first assembled into contigs, then polished using dedicated software. This sentence should be changed to something like (this is only the suggestion): Recently, many dedicated tools have been developed to help improve the consensus accuracy for the third-generation sequencing technology over 99% [41], therefore the extremely high error rate on the raw data should not affect the usage of PPR-Meta on assembled 3rd generation sequences.
5. In the case of the sentence: "PPR-Meta can also handle data from the third-generation sequencing technology, although it is designed primarily for the next-generation sequencing technology" (page 24): Isn't 3rd generation also the next-generation? Authors should consider changing â€žnext-generation" to â€ž2nd generation" or 3rd generation to single-molecule
6. The sentence "In the other hand, in order to identify sequences from low-abundance phages, which may fall into binning, we also need tools that can directly judge each fragment." (page 5) needs rephrasing

Level of Interest

Please indicate how interesting you found the manuscript: Choose an item.

Quality of Written English

Please indicate the quality of language in the manuscript: Choose an item.

Declaration of Competing Interests

Please complete a declaration of competing interests, considering the following questions:

- Have you in the past five years received reimbursements, fees, funding, or salary from an organisation that may in any way gain or lose financially from the publication of this manuscript, either now or in the future?
- Do you hold any stocks or shares in an organisation that may in any way gain or lose financially from the publication of this manuscript, either now or in the future?
- Do you hold or are you currently applying for any patents relating to the content of the manuscript?
- Have you received reimbursements, fees, funding, or salary from an organization that holds or has applied for patents relating to the content of the manuscript?
- Do you have any other financial competing interests?
- Do you have any non-financial competing interests in relation to this paper?

If you can answer no to all of the above, write 'I declare that I have no competing interests' below. If your reply is yes to any, please give details below.

I'm the author of PlasFlow, one of the tools compared to PPR-Meta in the manuscript. I declare that I have no other competing interests.

I agree to the open peer review policy of the journal. I understand that my name will be included on my report to the authors and, if the manuscript is accepted for publication, my named report including any attachments I upload will be posted on the website along with the authors' responses. I agree for my report to be made available under an Open Access Creative Commons CC-BY license (http://creativecommons.org/licenses/by/4.0/). I understand that any comments which I do not wish to be included in my named report can be included as confidential comments to the editors, which will not be published.

I agree to the open peer review policy of the journal

To further support our reviewers, we have joined with Publons, where you can gain additional credit to further highlight your hard work (see: https://publons.com/journal/530/gigascience). On publication of this paper, your review will be automatically added to Publons, you can then choose whether or not to claim your Publons credit. I understand this statement.

Yes Choose an item.
