# Supplementary material for: PPR-Meta: a tool for identifying phages and plasmids from metagenomic fragments using deep learning
Source: Gigascience. 2019 Jun 20;8(6):giz066. doi: 10.1093/gigascience/giz066 (PMC6586199; doi:10.1093/gigascience/giz066)
Supplement: giz066_Reviewer_2_Report_Original_Submission — Deyvid Amgarten -- 12/23/2018 Reviewed [file giz066_reviewer_2_report_original_submission.pdf]

Reviewer Report

Title: PPR-Meta: a tool for identifying phages and plasmids from metagenomic fragments using deep learning

Version: Original Submission **Date:** 12/23/2018

Reviewer name: Deyvid Amgarten

Reviewer Comments to Author:

Authors present a tool which is able to perform multiclass prediction of phage, plasmid and chromosome sequences in metagenomic data. Using this deep learning approach along with its architecture and the weighted average of all windows is worthy of publication by itself. Really interesting.

Tool, source code, virtual machine (along with a video explaining how to use it) and supporting datasets are all publicly available.

Also, the discussion section is really interesting. Noting that the differences between chromosome and phage scores may reflect phage lifestyle may be an relevant finding.

I am somehow concerned by the biological support of trying to predict these three classes. My concern in mainly about the overlap characteristics that plasmid and chromosomes usually present. And the same thing may be said about phages/prophages, chromosomes and plasmids. An example of this is explored by authors in results reported in page 20.

As a suggestion, authors should state clearly that the tool is aimed to perform a three-class prediction. Perhaps adding sentences to the abstract or even to the title.

Some other minor comments:

Page 5, line 13: "However, research has shown that viral sequences are highly fragmented in the metagenome [19], which may prevent binning, thereby limiting the usage of MARVEL." This is an unfair affirmation, since its only supporting reference is a 2013 article and it is safe to say that much has been done to improve metagenomic assemblers since then. Recent publications have been reporting the retrieval of phage complete and/or almost complete genomes by only applying assembly and binning approaches. I refer specially to the IMG-VR database (versions 1.0 and 2.0), which is a repository of thousands of viral sequences retrieved from metagenomic datasets all around the world. Nonetheless, there are many other publications in this line such as:

Paez-Espino, David, et al. "IMG/VR: a database of cultured and uncultured DNA Viruses and retroviruses." Nucleic acids research (2016): gkw1030.

Paez-Espino, David, et al. "IMG/VR v. 2.0: an integrated data management and analysis system for cultivated and environmental viral genomes." Nucleic acids research (2018).

Paez-Espino, David, et al. "Uncovering Earth's virome." Nature 536.7617 (2016): 425.

Sangwan, Naseer, Fangfang Xia, and Jack A. Gilbert. "Recovering complete and draft population genomes from metagenome datasets." Microbiome 4.1 (2016): 8.

Page 27, line 12 and other parts of the article: It is not clear whether authors extracted prophages from chromosomes to train the algorithm with more phages or to remove noise from chromosome datasets.

Do the chromosome datasets still contain their prophages?
A more practical question: For each query given by the user, will the tool automatically decide which model to use for prediction? Page 12 states how authors have proceeded by each query size, but my question regards the tool's behavior.

Level of Interest

Please indicate how interesting you found the manuscript: Choose an item.

Quality of Written English

Please indicate the quality of language in the manuscript: Choose an item.

Declaration of Competing Interests

Please complete a declaration of competing interests, considering the following questions:

- Have you in the past five years received reimbursements, fees, funding, or salary from an organisation that may in any way gain or lose financially from the publication of this manuscript, either now or in the future?
- Do you hold any stocks or shares in an organisation that may in any way gain or lose financially from the publication of this manuscript, either now or in the future?
- Do you hold or are you currently applying for any patents relating to the content of the manuscript?
- Have you received reimbursements, fees, funding, or salary from an organization that holds or has applied for patents relating to the content of the manuscript?
- Do you have any other financial competing interests?
- Do you have any non-financial competing interests in relation to this paper?

If you can answer no to all of the above, write 'I declare that I have no competing interests' below. If your reply is yes to any, please give details below.

I declare that I have no competing interests

I agree to the open peer review policy of the journal. I understand that my name will be included on my report to the authors and, if the manuscript is accepted for publication, my named report including any attachments I upload will be posted on the website along with the authors' responses. I agree for my report to be made available under an Open Access Creative Commons CC-BY license (http://creativecommons.org/licenses/by/4.0/). I understand that any comments which I do not wish to be included in my named report can be included as confidential comments to the editors, which will not be published.

I agree to the open peer review policy of the journal

To further support our reviewers, we have joined with Publons, where you can gain additional credit to further highlight your hard work (see: https://publons.com/journal/530/gigascience). On publication of this paper, your review will be automatically added to Publons, you can then choose whether or not to claim your Publons credit. I understand this statement.

Yes Choose an item.
