# Supplementary material for: PPR-Meta: a tool for identifying phages and plasmids from metagenomic fragments using deep learning
Source: Gigascience. 2019 Jun 20;8(6):giz066. doi: 10.1093/gigascience/giz066 (PMC6586199; doi:10.1093/gigascience/giz066)
Supplement: giz066_Reviewer_2_Report_Revision_1 — Deyvid Amgarten -- 4/6/2019 Reviewed [file giz066_reviewer_2_report_revision_1.pdf]

**Reviewer Report**

**Title: PPR-Meta: a tool for identifying phages and plasmids from metagenomic fragments using deep learning**

**Version: Revision 1** **Date:** 4/6/2019

**Reviewer name: Deyvid Amgarten**

**Reviewer Comments to Author:**

All of my concerns were properly addressed by authors, thank you.
Three further comments:
1. Regarding this statement in authors' response and similar statements in the manuscript (pg 24 and 25): "We found that in the position closer to the outer end of the digestive tract, the percentages of phages and plasmids tended to be higher. For example, in the gut, the inner end of the digestive tract, the percentages of phages and plasmids were lower; in the oral cavity, the outer end of the digestive tract, the percentages of phages and plasmids were higher."
It seems that authors' conclusion that "percentage of phage and plasmids are higher in the oral cavity" is only based in counts and proportions of contigs found in each one of these environments. However, abundance estimates would be more appropriate if based on raw reads counts. There are several factors which may affect assembly efficiency, and some (if not most) are not dependent on the total abundance of an organism in the sample. Presence of repeats are one good example which may prevent assembly. All of that said to suggest an approach like mapping raw reads to contigs predicted as phages and plasmids and calculating proportions of the total. Or counts based in total number of bases instead of absolute sequence counts.
2. I tried to access the website provided in the manuscript and it did not work for me. Please, be careful about links:
http://cqb.pku.edu.cn/ZhuLab/PPR_Meta/
3. I respectfully disagree with review #3 regarding virtual machines being an "odd choice and not at all standard in bioinformatics". I would say that this kind of approach has become more and more common in bioinformatics, specially for very complex software that require lots of dependencies. I work with keras and Tensor Flow, and as a matter of fact they are not easy to set up libraries. Virtual machines or docker containers are very practical way of running tools by people without specialized knowledge. Moreover, Virsorter (which is one of the tools that predict phage and prophages in contigs) also has a docker container, which is very useful. Nonetheless, I also appreciate the gold standard github availability, and that authors decreased VM size.

**Level of Interest**

Please indicate how interesting you found the manuscript: Choose an item.

**Quality of Written English**

Please indicate the quality of language in the manuscript: Choose an item.

**Declaration of Competing Interests**

Please complete a declaration of competing interests, considering the following questions:

- Have you in the past five years received reimbursements, fees, funding, or salary from an organisation that may in any way gain or lose financially from the publication of this manuscript, either now or in the future?
- Do you hold any stocks or shares in an organisation that may in any way gain or lose financially from the publication of this manuscript, either now or in the future?
- Do you hold or are you currently applying for any patents relating to the content of the manuscript?
- Have you received reimbursements, fees, funding, or salary from an organization that holds or has applied for patents relating to the content of the manuscript?
- Do you have any other financial competing interests?
- Do you have any non-financial competing interests in relation to this paper?

If you can answer no to all of the above, write 'I declare that I have no competing interests' below. If your reply is yes to any, please give details below.

I declare that I have no competing interests

I agree to the open peer review policy of the journal. I understand that my name will be included on my report to the authors and, if the manuscript is accepted for publication, my named report including any attachments I upload will be posted on the website along with the authors' responses. I agree for my report to be made available under an Open Access Creative Commons CC-BY license (http://creativecommons.org/licenses/by/4.0/). I understand that any comments which I do not wish to be included in my named report can be included as confidential comments to the editors, which will not be published.

I agree to the open peer review policy of the journal

To further support our reviewers, we have joined with Publons, where you can gain additional credit to further highlight your hard work (see: https://publons.com/journal/530/gigascience). On publication of this paper, your review will be automatically added to Publons, you can then choose whether or not to claim your Publons credit. I understand this statement.

Yes Choose an item.
