# Supplementary material for: PPR-Meta: a tool for identifying phages and plasmids from metagenomic fragments using deep learning
Source: Gigascience. 2019 Jun 20;8(6):giz066. doi: 10.1093/gigascience/giz066 (PMC6586199; doi:10.1093/gigascience/giz066)
Supplement: giz066_Reviewer_3_Report_Original_Submission — Corey Hudson -- 12/25/2018 Reviewed [file giz066_reviewer_3_report_original_submission.pdf]

**Reviewer Report**

**Title: PPR-Meta: a tool for identifying phages and plasmids from metagenomic fragments using deep learning**

**Version: Original Submission** **Date:** 12/25/2018

**Reviewer name: Corey Hudson**

**Reviewer Comments to Author:**

This paper describes PPR-Meta - a tool for identifying phages and plasmids from metagenomic fragments using deep learning. The authors distribute the software through their own site as Virtual Machine and through Github as a set of Matlab and h5 model files. The distribution is very nonintuitive and unconventional. It would be far better for the authors to distribute as a standard git package, rather than as a .zip file. Furthermore, the requirement that the use download a 30gb Virtual Machine image is odd and not at all standard in bioinformatics. The installation of PPR-Meta should be possible without having to instantiate an entire VM. I would greatly prefer a makefile and configure script with the requirement of a Ubuntu OS, over downloading a 30gb over http. This may qualify as a major revision, because I assume switching to this would require a lot of additional work.
There are some basic issues in the paper in detecting phage vs. prophage vs. plasmid. I think the paper leans heavily on phage and plasmid databases, over which it excels in a metagenomic context, but doesn't address the large corpus of prophage and genomic island databases in the literature. I would prefer these to be addressed and compared. The entire literature on genomic islands, including experimental, feature-based and machine-learning based methods for identification are largely missing. The authors use ProphET to identify the prophages. This is a machine learning based approach, and not a structure or experimental-based gold standard dataset.
Figures 1 and 2, describing the bi-path convolutional network could be considerably clearer. Specifically the whole process would be clearer if the "base one-hot matrix" and "codon one-hot matrix" were better understood. Furthermore, the description of layers should be clear enough in the figure that they are unnecessary in the text. Finally, justification for the NN architecture is not offered. Is this a standard architecture? Was it randomly created? Was it optimized?
Training on simulated data, rather than on a known set of MGEs was a strange choice, I would like to see this more sufficiently explained. In the prophage identification section, only 267 manually annotated prophages are included, when there are in fact thousands of annotated prophages and genomic islands. Finally, I would like to see the analysis of this software performed on a large metagenomic dataset. What percentage of the an ocean, fecal or soil sample is made up of phages/plasmids? Does the tool simply key in on odd codon/base pair coding, or is it keying in on larger gene-level issues? With a neural network, this is not always easy to tell, but could have fundamental impacts on how we view the metagenomics of mobile elements.

**Level of Interest**

Please indicate how interesting you found the manuscript: Choose an item.

**Quality of Written English**

Please indicate the quality of language in the manuscript: Choose an item.

**Declaration of Competing Interests**

Please complete a declaration of competing interests, considering the following questions:

- Have you in the past five years received reimbursements, fees, funding, or salary from an organisation that may in any way gain or lose financially from the publication of this manuscript, either now or in the future?
- Do you hold any stocks or shares in an organisation that may in any way gain or lose financially from the publication of this manuscript, either now or in the future?
- Do you hold or are you currently applying for any patents relating to the content of the manuscript?
- Have you received reimbursements, fees, funding, or salary from an organization that holds or has applied for patents relating to the content of the manuscript?
- Do you have any other financial competing interests?
- Do you have any non-financial competing interests in relation to this paper?

If you can answer no to all of the above, write 'I declare that I have no competing interests' below. If your reply is yes to any, please give details below.

None

I agree to the open peer review policy of the journal. I understand that my name will be included on my report to the authors and, if the manuscript is accepted for publication, my named report including any attachments I upload will be posted on the website along with the authors' responses. I agree for my report to be made available under an Open Access Creative Commons CC-BY license (http://creativecommons.org/licenses/by/4.0/). I understand that any comments which I do not wish to be included in my named report can be included as confidential comments to the editors, which will not be published.

I agree to the open peer review policy of the journal

To further support our reviewers, we have joined with Publons, where you can gain additional credit to further highlight your hard work (see: https://publons.com/journal/530/gigascience). On publication of this paper, your review will be automatically added to Publons, you can then choose whether or not to claim your Publons credit. I understand this statement.

Yes Choose an item.
