# Supplementary material for: PPR-Meta: a tool for identifying phages and plasmids from metagenomic fragments using deep learning
Source: Gigascience. 2019 Jun 20;8(6):giz066. doi: 10.1093/gigascience/giz066 (PMC6586199; doi:10.1093/gigascience/giz066)
Supplement: giz066_Reviewer_3_Report_Revision_1 — Corey Hudson -- 4/5/2019 Reviewed [file giz066_reviewer_3_report_revision_1.pdf]

Reviewer Report

Title: PPR-Meta: a tool for identifying phages and plasmids from metagenomic fragments using deep learning

Version: Revision 1 **Date:** 4/5/2019

Reviewer name: Corey Hudson

Reviewer Comments to Author:

The authors have addressed many of my issues. In detail:
1. I brought up that I thought the files should be distributed over a more conventional means, like GitHub. I still have concerns about the requirement to download an entire VM. However, given that the journal's author guidelines specify allowing a virtual machine, I think the authors have acted in the best possible way. I appreciate the authors willingness to further compress the download, from 30 to 2.5 Gb in size. While the program is still quite large, it is much less unwieldy.
2. In my comments, I addressed issues with the detection of phage vs. prophage vs. plasmid - in particular the reliance on phage and plasmid databases and the lack of experimentally verified data, particularly, the lack of genomic islands. The authors updated their analysis, including more phage/plasmid manually curated data, and data on genomic islands. They also showed the performance of these in comparison. This reanalysis and discussion assuages my concerns about the limitations in the analysis.
3. The justification for Figure 1 and the elimination of the original Figure 2 sufficiently addressed my concerns that the motivation and description of the FNN were insufficient. I appreciate that the authors show all the changes that resulted from these changes.
4. Many of the issues from this comment were taken into account in comment #2 and I appreciate the full description in the authors' responses.
5. The authors went above-and-beyond what was required by including the HMP data. This is a major improvement to the generalizability of the analysis. I still believe that the second part hasn't been sufficiently addressed in the manuscript, but has in the comments. Having a robust justification for why the authors believe PPR-Meta is keying on sequence signatures and what the implications of that are, may warrant subsequent work.
Given that all of my comments have been addressed, either in the papers or in the response to reviewer and that the primary concerns are generally addressed, I recommend that this paper be accepted for publication.

Level of Interest

Please indicate how interesting you found the manuscript: Choose an item.

Quality of Written English

Please indicate the quality of language in the manuscript: Choose an item.

Declaration of Competing Interests

Please complete a declaration of competing interests, considering the following questions:

- Have you in the past five years received reimbursements, fees, funding, or salary from an organisation that may in any way gain or lose financially from the publication of this manuscript, either now or in the future?
- Do you hold any stocks or shares in an organisation that may in any way gain or lose financially from the publication of this manuscript, either now or in the future?
- Do you hold or are you currently applying for any patents relating to the content of the manuscript?
- Have you received reimbursements, fees, funding, or salary from an organization that holds or has applied for patents relating to the content of the manuscript?
- Do you have any other financial competing interests?
- Do you have any non-financial competing interests in relation to this paper?

If you can answer no to all of the above, write 'I declare that I have no competing interests' below. If your reply is yes to any, please give details below.

I declare I have no competing interests

I agree to the open peer review policy of the journal. I understand that my name will be included on my report to the authors and, if the manuscript is accepted for publication, my named report including any attachments I upload will be posted on the website along with the authors' responses. I agree for my report to be made available under an Open Access Creative Commons CC-BY license (http://creativecommons.org/licenses/by/4.0/). I understand that any comments which I do not wish to be included in my named report can be included as confidential comments to the editors, which will not be published.

I agree to the open peer review policy of the journal

To further support our reviewers, we have joined with Publons, where you can gain additional credit to further highlight your hard work (see: https://publons.com/journal/530/gigascience). On publication of this paper, your review will be automatically added to Publons, you can then choose whether or not to claim your Publons credit. I understand this statement.

Yes Choose an item.
